# Organocatalytic Enantioselective α-Nitrogenation of α,α-Disubstituted Aldehydes in the Absence of a Solvent

**DOI:** 10.1021/acs.joc.2c01919

**Published:** 2022-10-25

**Authors:** Alejandro Torregrosa-Chinillach, Asier Carral-Menoyo, Enrique Gómez-Bengoa, Rafael Chinchilla

**Affiliations:** † Department of Organic Chemistry and Institute of Organic Synthesis (ISO), University of Alicante, PO Box 99, Alicante 03080, Spain; ‡ Department of Organic Chemistry I, University of the Basque Country UPV/EHU, Manuel Lardizabal 3, Donostia-San Sebastián 20018, Spain

## Abstract

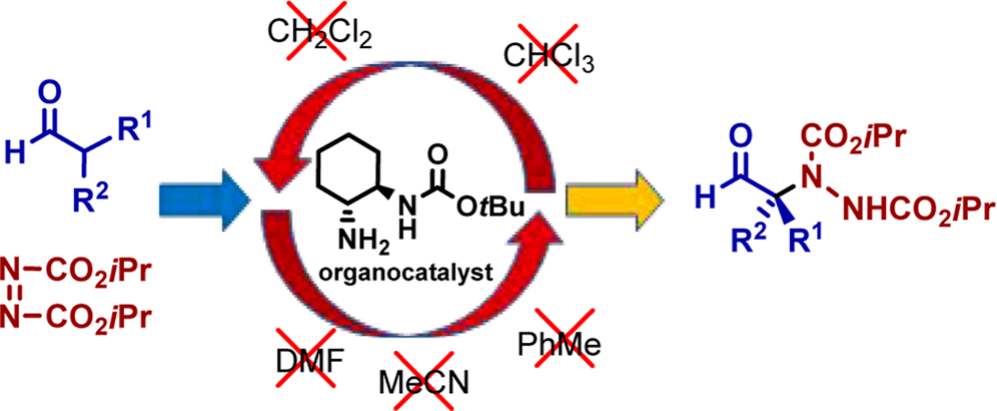

A highly efficient enantioselective α-nitrogenation method of α,α-disubstituted aldehydes with azodicarboxylates promoted by a chiral carbamate-monoprotected cyclohexa-1,2-diamine as organocatalyst has been developed. The process was carried out without any solvent, and the corresponding α,α-disubstituted α-nitrogenated aldehydes were obtained with excellent yields and enantioselectivities up to 99% *ee*. The sustainability of the procedure was established through the calculation of green metrics, such as EcoScale and E-factor. In addition, theoretical calculations have been used to justify the obtained enantioselectivity sense.

## Introduction

Enantioenriched α-nitrogenated aldehydes are important building blocks in chemical synthesis, with many applications in medicinal chemistry and the pharmaceutical industry.^1−5^ The aldehyde functionality can be transformed into various functional groups, leading to substituted chiral amines in natural products and bioactive substances.^6^ Particularly interesting is the synthesis of quaternary α-amino aldehydes as they can be transformed into quaternary α-amino acids, which are building blocks for constructing peptidomimetics or valuable as pharmaceuticals.^7−11^ An example of the latter is α-aryl−α-alkyl α-amino acid derivatives, which have shown strong inhibitory effects on aldose reductases, a potential target for treating various diabetes-related diseases.^12^

**Figure 1:**
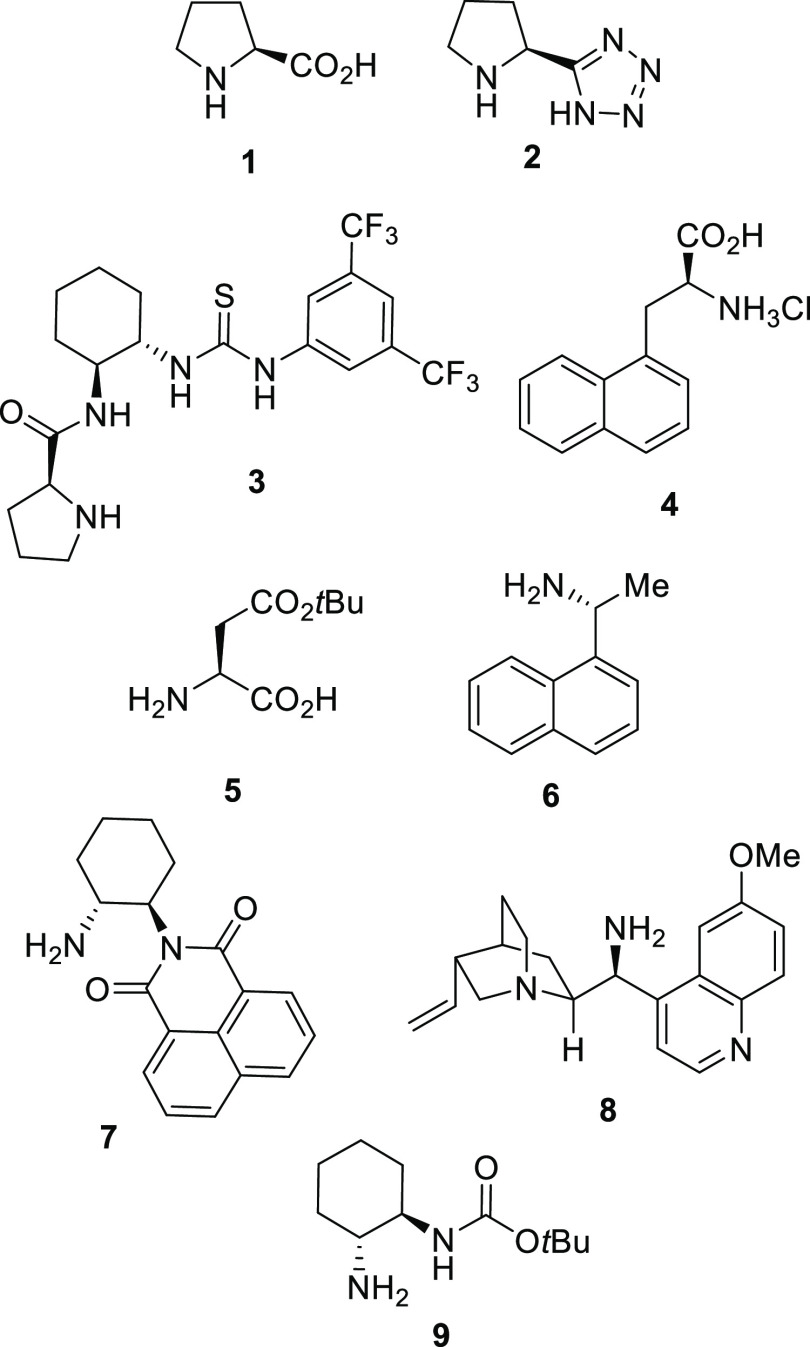
Organocatalysts **1**–**8** employed previously in the enantioselective addition of α,α-disubstituted aldehydes to azodicarboxylates and organocatalyst **9** used in this study.

Although not too extensively explored, organocatalysis has been found as a direct and convenient method for the asymmetric synthesis of quaternary α-amino aldehydes through the conjugate addition reaction of α,α-disubstituted aldehydes with azodicarboxylates as the electrophilic nitrogen source. Chiral amine-containing species, suitable to generate an enamine nucleophile and, at the same time, coordinate with the electrophile to get a close transition state, have been used as organocatalysts (Figure 1).

Thus, l-proline (**1**) has been the pioneering and mainly employed organocatalyst in this reaction.^13−18^ In addition, the secondary amine in l-proline has also been the enamine-forming moiety in the case of the l-proline-derived tetrazole **2**, employed in the synthesis of cell adhesion inhibitor BIRT-377^19^ or prolinamide-derived thiourea **3**.^20^ Moreover, chiral primary amines have also been used as organocatalysts for this enamine-driven transformation, as is the case of the amino acids 3-(1-naphthyl) alanine hydrochloride (**4**)^21^ and l-β-*tert*-butyl aspartate (**5**).^22^ Other organocatalysts containing primary amines have been naphthylethanamine **6**,^23^ chiral benzoisoquinoline-1,3-dione **7**,^24^ and 9-amino-(9-deoxy)-*epi*-quinine (**8**), alone^25−27^ or combined with (−)-camphorsulfonic acid as a chiral counteranion^28^ or even magnetically supported.^29^

These mentioned organocatalysts **1–8** only afforded good enantioselectivities starting from α-alkyl−α-aryl aldehydes. In addition, as is usual, an organic solvent is always present. Thus, environmentally unfriendly halogenated media^13,15,20,24,25,28^ or highly volatile and flammable^22,23^ or toxic^14,16−19,21^ solvents have been employed. As the solvent is one of the key elements for naming a chemical process as sustainable, we have taken seriously into consideration the aphorism “the best solvent is no solvent”^30−34^ and have developed a highly efficient and greener enantioselective solvent-free α-amination of α,α-disubstituted aldehydes with azodicarboxylates, using a simple mono-*N*-Boc-protected cyclohexa-1,2-diamine **9**(35) as a chiral organocatalyst.

## Results and Discussion

The conjugate addition reaction between 2-phenylpropanal (**10a**) and diisopropyI azodicarboxylate (DIAD, **11a**), in the presence of the monocarbamate derived from (1*R*,2*R*)-cyclohexa-1,2-diamine **9**(35) as an organocatalyst (20 mol%), was chosen as a model reaction to optimize the reaction conditions (see Table 1). Initially, we were interested in the behavior of organocatalyst **9** in conventional, solvent-present reaction conditions. Thus, the reaction of **10a** (2 equiv) with **11a** organocatalyzed by **9** (20 mol%) in several organic solvents at room temperature for 48 h afforded the α-nitrogenated α,α-disubstituted aldehyde **12aa** in up to 71% *ee* (Table 1, entries 1–6). The absolute stereochemistry of **12aa** was determined according to the order of elution of the corresponding enantiomers in chiral HPLC reported in the literature (see the Supporting Information).

**Table 1:**
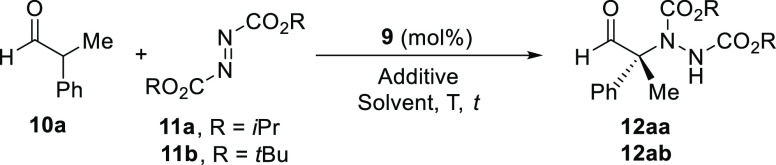
Enantioselective Organocatalytic α-Amination of Aldehyde **10a** with Azodicarboxylates: Optimization Experiments

entry	R	9 (mol %)	**10a**/**11** molar ratio	additive (mol %)^a^	solvent	*T* (°C)	*t* (h)	% conv.^b^	% *ee*^c^
1	*i*Pr	20	2/1		PhMe	25	48	89	52
2	*i*Pr	20	2/1		CH_2_Cl_2_	25	48	93	54
3	*i*Pr	20	2/1		DMF/H_2_O^d^	25	48	100	57
4	*i*Pr	20	2/1		THF	25	48	57	60
5	*i*Pr	20	2/1		TBME	25	48	73	71
6	*i*Pr	20	2/1		Et_2_O	25	48	51	71
7	*i*Pr	20	2/1			25	24	100	69
8	*i*Pr	20	1/1.2			25	24	100	75
9	*i*Pr	20	1/1.2	DABCO (10)		25	8	100	0
10	*i*Pr	20	1/1.2	HDA (10)		25	8	100	77
11	*i*Pr	20	1/1.2	PhCO_2_H (10)		25	8	100	71
12	*i*Pr	20	1/1.2	3,4-DMBA (10)		25	8	100	73
13	*i*Pr	20	1/1.2	NBA (10)		25	8	100	81
14	*i*Pr	20	1/1.2	AcOH (10)		25	8	100	83
15	*i*Pr	10	1/1.2	AcOH (10)		25	8	100	81
16	*i*Pr	20	1/1.2	AcOH (20)		25	8	100	87
17	*i*Pr	20	1/1.2	AcOH (30)		25	8	100	76
18	*t*Bu	20	1/1.2	AcOH (20)		25	8	100 (98)	84
19	*i*Pr	20	1/1.2	AcOH (20)		–10	24	100	88
20	*i*Pr	20	1/1.2	AcOH (20)		–20	24	100 (99)	94
21	*i*Pr	20	1/1.2	AcOH (20)		–30	24	100	89
22	*i*Pr	10	1/1.2	AcOH (20)		–20	24	100	70
23	*i*Pr		1/1.2	AcOH (20)		–20	24	3	

a Abbreviations: DABCO: 1,4-diazabicyclo[2.2.2]octane; 3,4-DMBA: 3,4-dimethoxybenzoic acid; HDA: hexanodioic acid; NBA: 4-nitrobenzoic acid; TBME: *tert*-butyl methyl ether.

b Determined by ^1^H NMR from the remaining aldehyde; isolated yield after flash chromatography in parenthesis.

c Enantioselectivities and absolute stereochemistry determined by chiral HPLC on the reaction crude.

d 2:1 (v/v).

However, when the same reaction was carried out under solvent-free conditions, the reaction was completed in 24 h, observing similar enantioselectivity for **12aa** to the one obtained when the best solvent was used (Table 1, compare entry 7 with entries 5 and 6). In addition, when the molar ratio of the reagents was modified and 1.2 equiv of **11a** were used under solvent-free conditions, the conversion remained unaltered in 24 h, and the enantioselectivity for **12aa** increased slightly to 75% (Table 1, entry 8). This change in the molar ratio is important as the usual excess of the most expensive or not commercially available aldehyde is avoided. Therefore, as solvent-free conditions resulted in a superior methodology, we further optimize this environmentally friendlier procedure.

We explored if the presence of additives was beneficial for the reaction. The addition of an organic base such as 1,4-diazabicyclo[2.2.2]octane (DABCO, 10 mol%) gave full conversion in only 8 h, although it was disastrous for the enantioselectivity, obtaining **12aa** as a racemic mixture (Table 1, entry 9). However, the use of acids as additives (10 mol%) also gave full conversions in 8 h while keeping the enantioselectivity (Table 1, entries 10–14), with the best *ee* (83%) being achieved using the economic acetic acid as an additive (Table 1, entry 14). Lowering the loading of the organocatalyst **9** down to 10 mol% diminished the enantioselectivity slightly for **12aa** (Table 1, entry 15), while keeping the loading of **9** in 20 mol% and increasing the loading of the acid additive up to 20 mol% raised the enantioselectivity of **12aa** (87%) (Table 1, entry 16). A further increase in the amount of acetic acid up to 30 mol% was detrimental to the enantioselectivity (Table 1, entry 17).

Using these conditions, we employed di-*tert*-butyl azodicarboxylate (**11b**) as a bulkier electrophilic nitrogen source, although the corresponding nitrogenated aldehyde **12ab** was obtained in lower enantioselectivity (Table 1, entry 18). The use of diethyl azodicarboxylate (DEAD) was discarded because it is commercially available in solution due to its explosive potential.

Finally, we lowered the reaction temperature. Thus, when the reaction was carried out at −10 °C, the *ee* observed for **12aa** raised slightly (Table 1, entry 19), increasing to 94% when working at −20 °C (Table 1, entry 20). A lower reaction temperature (−30 °C) did not increase the enantioselectivity (Table 1, entry 21). Diminishing the catalyst loading to 10 mol% at −20 °C gave only 70% *ee* of **12aa** (Table 1, entry 22). This change in enantioselectivity with catalyst loading has been previously observed when using this organocatalyst in other enantioselective reactions.^25^ This could suggest that when the catalyst concentration reaches a certain value, other species (even combined with the cocatalyst), such as aggregates, could also be present, modifying the enantioselectivity of the process upward. Moreover, in the absence of organocatalyst **9** and in the presence of 20 mol% of AcOH as a cocatalyst, almost no reaction was observed (Table 1, entry 23). In the absence of a background reaction, enantioselectivity should not vary with catalyst loading unless some other processes, such as described above, are occurring.

Once the optimized reaction conditions were established [**9** (20 mol%), AcOH (20 mol%), **11a** (1.2 equiv), solvent-free, −20 °C, 24 h], we proceeded to extend the application of this organocatalytic methodology to other α,α-disubstituted aldehydes **10** (see Table 2). Thus, we explored a series of α-arylated propanals **10b–k**, bearing different substituents on the aromatic ring (Table 2, entries 2–11).

**Table 2:**
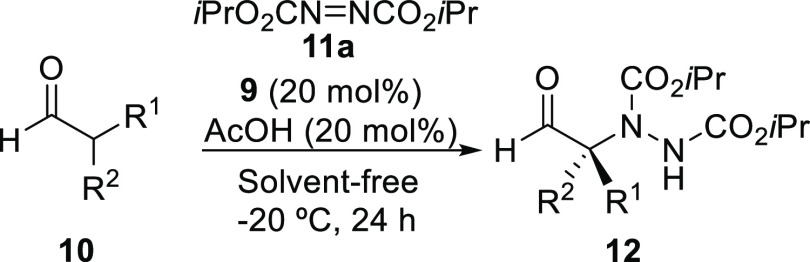

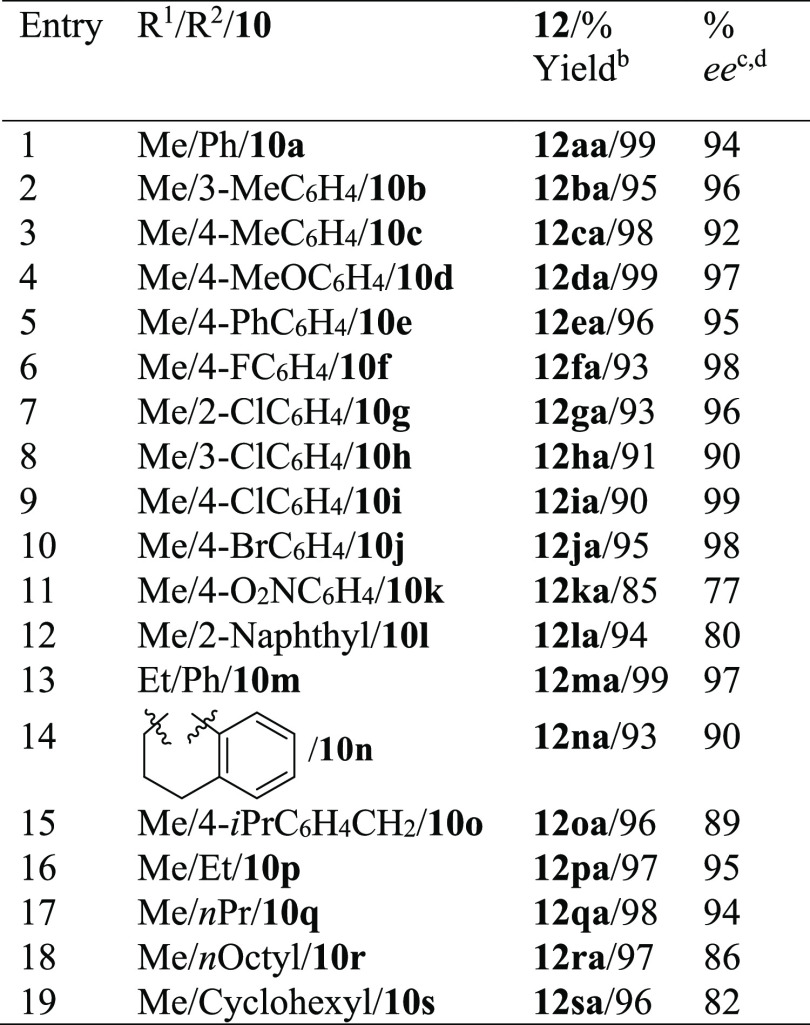
Enantioselective Organocatalytic α-Amination of α,α-Disubstituted Aldehydes **10** under Solvent-Free Conditions^a^

a Reactions were carried out by mixing **10** (0.2 mmol), **11a** (0.24 mmol), catalyst **9** (0.04 mmol), and AcOH (0.04 mmol).

b Isolated yield after flash chromatography.

c Determined by chiral HPLC on the reaction crude (see the Supporting Information).

d Absolute stereochemistry of known compounds was determined according to the elution order of the corresponding enantiomers (chiral HPLC) in the literature (see the Supporting Information). Absolute stereochemistry of unknown compounds was assigned by analogy.

In all cases, high isolated yields and high enantioselectivities of the corresponding α-aminated aldehydes **12ba**-**ja** were obtained, although in the case of the 4-nitro-containing aldehyde **10k**, the corresponding adduct **12ka** was obtained in a lower 77% *ee* (Table 2, entry 11). In addition, a 2-naphthyl in the aldehyde **10l** gave the corresponding adduct **12la** in an 80% *ee* (Table 2, entry 12). Moreover, changing the α-methyl in the aldehyde **10a** by ethyl (**10m**) allowed us to obtain the corresponding final nitrogenated aldehyde **12ma** in 97% *ee* (Table 2, entry 13). Furthermore, when 1,2,3,4-tetrahydronaphthalene-1-carbaldehyde (**10n**) was employed as the starting aldehyde, the final adduct **12na** was obtained in a 90% *ee* (Table 2, entry 14).

When using an α-benzylated-α-methyl aldehyde, such as cyclamen aldehyde (**10o**), the reaction gave the corresponding nitrogenated adduct **12oa** with an enantioselectivity of 89% (Table 2, entry 15). Interestingly, when several α,α-dialkylated aldehydes **10p–s** were used as starting materials, the final adducts **12pa**-**sa** were obtained with excellent yields and enantioselectivities ranging from 82 to 95% (Table 2, entries 16–19). These high enantioselections are remarkable as low or quite moderate enantioselectivities have been obtained from α,α-dialkylated aldehydes using other organocatalysts and solvent-including reaction conditions.^13,15,20−25,28^ These last results demonstrated the unusual applicability of this methodology to all kinds of starting disubstituted aldehydes.

We also carried out the synthesis of compound **12qa** under the optimal reaction conditions, but using 20 mol% of proline (**1**) as a commonly used organocatalyst, achieving an enantioselectivity of 59% *ee*. In addition, the use of 20 mol% of chiral naphthylethanamine **6** gave **12aa** in 63% *ee* and quinine-derived amine **8** afforded 71% *ee*. These enantioselections are noticeably higher than those reported for similar adducts from **10q** or other dialkylated aldehydes when using these catalysts under conventional solvent-including reaction conditions.^15,23,25^

To evaluate the “greenness” of our protocol, we calculate the EcoScale and E-factor of the reactions, leading to adducts **12** (see Table S1 in the Supporting Information). Thus, the obtained EcoScale^36^ values ranged from 63 to 72, which means, according to the established definitions, that the reaction conditions cannot be ranked as “excellent” (EcoScale > 75) but can be situated in the upper part of “acceptable” (EcoScale > 50). No “inadequate” reaction conditions were observed (EcoScale < 50). Expectedly, the EcoScale values from this solvent-free methodology are higher than the calculated for previously reported solvent-including reaction conditions (see Table S1 in the Supporting Information). In addition, the E-factor^37^ for the preparation of all compounds **12** was initially calculated excluding purification materials (no workup is employed), giving values in the range 0.27–0.47 (see Table S1 in the Supporting Information). However, more realistic values (244–346) were obtained when considering the purification by column chromatography, a method present in all previously reported procedures, which also include workup.

We also proved the easy scalability of this solvent-free process by carrying out the conjugate addition of aldehyde **10a** with **11a** under the conditions of Table 2 but on a 3 mmol scale of **10a**. This scaled-up reaction allowed us to isolate 0.96 g (95% yield) of the adduct **12aa** in a 93% enantioselectivity, which are almost identical results to those obtained when working on a 0.2 mmol scale (Table 2, entry 1).

**Scheme 1:**
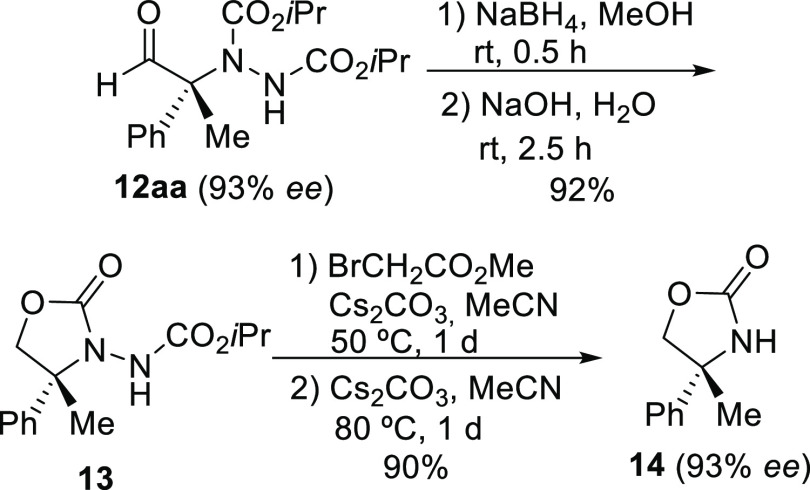
Preparation of Enantioenriched Oxazolidinone **14** from α-Nitrogenated Aldehyde **12aa**

The synthetic usefulness of these enantioenriched nitrogenated adducts **12** was exemplified by preparing the hydrazide-containing oxazolidin-2-one **13** in a 92% yield after reduction of aldehyde **12aa**, obtained in the scaled-up reaction (93% *ee*), and further cyclization (Scheme 1). The subsequent reaction of **13** with methyl bromoacetate in the presence of cesium carbonate, and additional treatment with this base, gave the (*R*)-oxazolidinone **14** in a 90% yield and the expected 93% *ee*.

**Figure 2:**
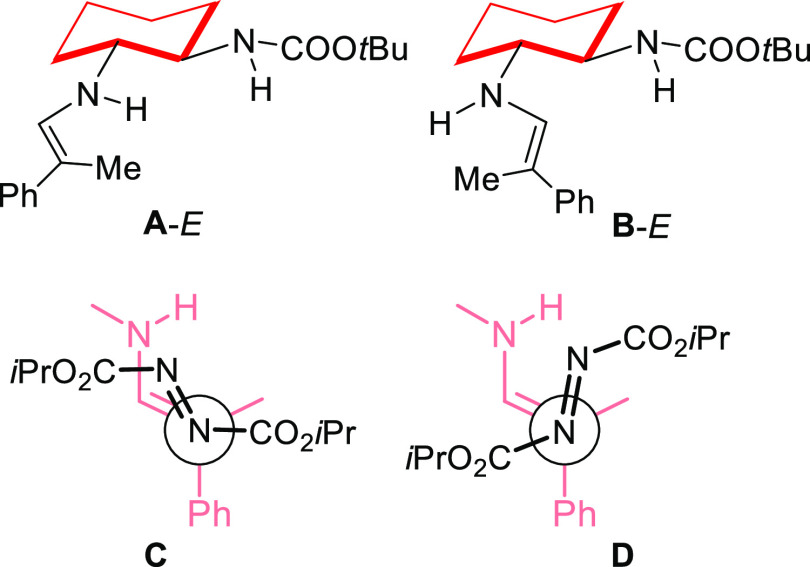
Computational models of the nucleophilic *E*-enamine (A,B) and the endo (C) and exo (D) transition states.

To get further insight into the behavior of organocatalyst **9** when leading to the observed sense of enantioselectivity, we performed DFT theoretical calculations (see the Supporting Information) on substrates **10a** and **11a**. Thus, the initial enamine formed between aldehyde **10a** and catalyst **9** can adopt two different configurations, and the most stable one corresponds to the phenyl group trans to the NH moiety (**A**-*E* isomer, Figure 2). The **A**-*Z* enamine was found to be 1.4 kcal/mol higher in energy, affording a predicted 10:1 *E*/*Z* enamine ratio. This difference is significant but not enough to discard the participation of both enamines in the reaction (see below).

From this initial point, the nucleophilic attack on the azodicarboxylate looks quite straightforward to compute. However, as previously described by our groups in related reactions,^35^ the calculations showed a complex mixture of possible conformations and reacting faces of the two substrates. For example, just to indicate some of the varying factors, the enamine can adopt different conformations, that is, **A** (*E* and *Z*) or **B** (*E* and *Z*) (Figure 2). The approach between the enamine and azodicarboxylate can proceed through diastereomeric *endo* (**C**) or *exo* (**D**) transition states, and both the nucleophile and electrophile present two reacting Si and Re faces. In the case of the azodicarboxylate, these faces do not generate a new stereogenic center but still strongly affect the approach’s selectivity to the enamine. Meanwhile, the two faces of the enamine lead to the *R* and *S* enantiomers of product **12aa** after forming the corresponding diastereoselective transition states.

**Figure 3:**
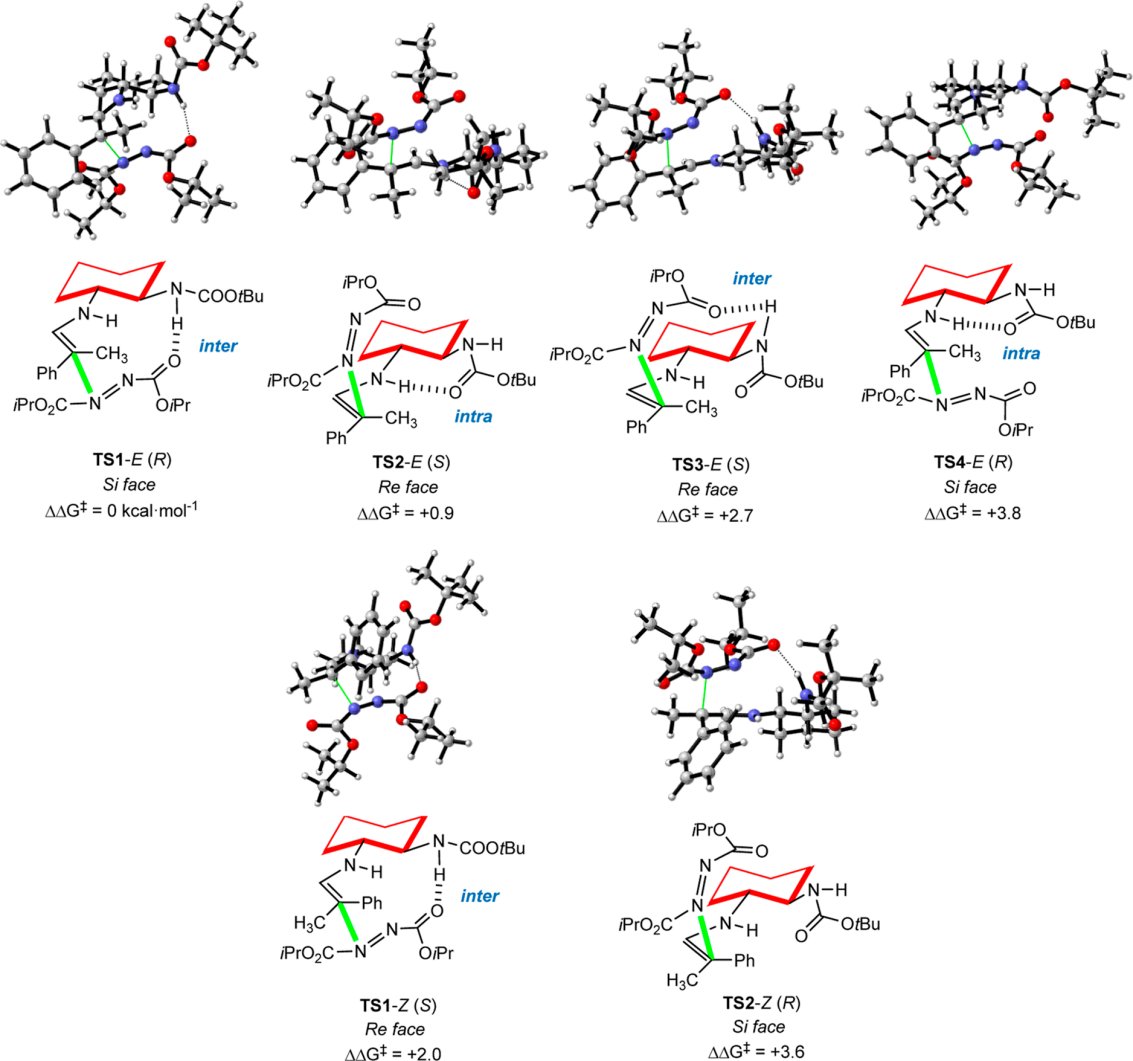
Computed transition states for the reaction between the enamine from **9** and **10a** and **11a**. Energies are given in kcal/mol (see the Supporting Information).

Following extensive calculations of all possibilities, structure **A**-*E* was found to be the most reactive enamine, where two main factors affect the electrophile’s approach. First, the *Re* face (upper face in **A**-*E* representation), which leads to the minor *S* enantiomer of the product, is less sterically congested, as computed by the difference between **TS2** and **TS4** (Figure 3). Meanwhile, an intramolecular hydrogen bond can be formed between the carbamate NH of the catalyst and one of the carbonyl groups in the azodicarboxylate. This H-bond is easier to create and stronger in the lower *Si* face of the enamine (**TS1** vs **TS3**). Thus, two opposite factors are competing, with the steric one favoring the *Re* approach and the H-bond leading to the *Si* face activation. The computed energies of the most preferred structures (**TS1–TS4**) show the stronger stabilizing effect of the H-bond, which outcompetes the steric destabilization, making **TS1**-*E* the lowest transition state in energy and explaining the formation of the experimental *R* major enantiomer. Related transition states were found for the minor *Z* enamines, but their activation energies are at least 2.0 kcal/mol higher, indicating that they do not participate in the reaction. It is worth noting that, in the absence of the intermolecular H-bond between the electrophile and enamine, a weaker intramolecular H-bond forms between the NH of the enamine and the carbonyl group of the carbamate, both in the catalyst. Finally, the calculations were repeated with aldehyde **10p**, containing methyl and ethyl substituents, and the results were almost identical to the previous ones, reinforcing the agreement between calculations and experiments (see Figure S106 in the Supporting Information).

## Conclusions

α,α-Disubstituted aldehydes have been enantioselectively α-nitrogenated employing diisopropyl azodicarboxylate as the electrophilic nitrogen source and a simple chiral mono-*N*-Boc-protected cyclohexa-1,2-diamine as the organocatalyst under solvent-free conditions, the presence of acetic acid as an additive improving the results. Contrary to other reported methodologies, high yields and enantioselectivities were obtained from alkyl-α-aryl aldehydes and their α,α-dialkylated counterparts. The obtained adducts can be transformed into valuable compounds, such as enantioenriched oxazolidine-2-ones. Theoretical calculations confirmed the bifunctional behavior of the employed organocatalyst, responsible for the formation of the nucleophilic enamine and the activation of the electrophilic azodicarboxylate through the creation of an intermolecular hydrogen bond with the NH of the carbamate. The stabilizing effect of the H-bond is also responsible for the enantioselective formation of the major *R* isomer. This procedure is efficient, easily scalable, and environmentally convenient for the enantioselective preparation of synthetically useful α,α-disubstituted α-nitrogenated aldehydes.

## Experimental Section

### General Procedure for the Organocatalytic Enantioselective α-Nitrogenation

A glass vial (ø 16 mm) was charged with **9** (8.6 mg, 0.04 mmol, 0.2 equiv), acetic acid (2.3 μL, 0.04 mmol, 0.2 equiv), aldehyde **10** (0.2 mmol, 1 equiv), and azodicarboxylate **11** (0.24 mmol, 1.2 equiv). The mixture was gently stirred at −20 °C under an argon atmosphere for 24 h. After this time, the reaction crude was purified by column chromatography [silica gel, hexanes/ethyl acetate (85/15, v/v)] to afford the product **12**. All characterization data of compounds **12** are available in the Supporting Information.

### Scaled-Up Synthesis of **12aa**

A glass vial (ø 16 mm) was charged with **9** (0.6 mmol, 129 mg), acetic acid (0.6 mmol, 36 mg, 34.5 μL), aldehyde **10a** (3 mmol, 402 mg, 0.40 mL), and azodicarboxylate **11a** (3.6 mmol, 727 mg, 0.73 mL). The mixture was gently stirred at −20 °C under an argon atmosphere for 24 h. After this time, the reaction crude was purified by column chromatography [silica gel, hexanes/ethyl acetate (85/15, v/v)] to afford product **12aa** (0.95 g, 95%, 93% *ee*).

## Supporting Information Available

Materials, methods, experimental procedures, characterization data, ^1^H NMR and ^13^C NMR spectra, HPLC chromatograms, calculation of sustainability metrics, and computational data (PDF)
