# Supplementary material for: Organocatalytic Enantioselective α-Nitrogenation of α,α-Disubstituted Aldehydes in the Absence of a Solvent
Source: J Org Chem. 2022 Oct 25;87(21):14507–13. doi: 10.1021/acs.joc.2c01919 (PMC9639010; doi:10.1021/acs.joc.2c01919)
Supplement: Supplementary file 1 — jo2c01919_si_001.pdf [file jo2c01919_si_001.pdf]

# Supporting Information

# Organocatalytic Enantioselective α-Nitrogenation of α,α-Disubstituted Aldehydes in the Absence of Solvent

*Alejandro Torregrosa-Chinillach,[a] Asier Carral-Menoyo,[b] Enrique Gómez-Bengoa*[b] and Rafael Chinchilla*[a]*

[a] Department of Organic Chemistry and Institute of Organic Synthesis (ISO), University of Alicante, PO Box 99, 03080 Alicante (Spain)

[b] Department of Organic Chemistry I, University of the Basque Country UPV/EHU, Manuel Lardizabal 3, 20018 Donostia-San Sebastián (Spain)

*Corresponding authors

enrique.gomez@ehu.es; chinchilla@ua.es

**Number of pages:** 105

**Number of figures:** 106

**Number of tables:** 53

## Table of contents


II. General experimental procedures ........ S5

**Figure S1.** Enantioselective α-nitrogenation of aldehydes organocatalyzed by **8**. ........ S5

**Figure S2.** Synthesis of oxazolidin-2-one **14** from α-nitrogenated aldehyde **12aa**. ........ S6

III. Characterization data of compounds **12**, **13** and **14** ........ S7

IV. $^{1}$H NMR spectrum of **9** ........ S13

**Figure S3.** $^{1}$H NMR (300 MHz, $CDCl_3$) of organocatalyst **9**. ........ S13

V. $^{1}$H NMR spectra of **10b-n** and **10s** ........ S13

**Figure S4.** $^{1}$H NMR (300 MHz, $CDCl_3$) of aldehyde **10b**. ........ S13

**Figure S5.** $^{1}$H NMR (300 MHz, $CDCl_3$) of aldehyde **10c**. ........ S14

**Figure S6.** $^{1}$H NMR (300 MHz, $CDCl_3$) of aldehyde **10d**. ........ S14

**Figure S7.** $^{1}$H NMR (300 MHz, $CDCl_3$) of aldehyde **10e**. ........ S15

**Figure S8.** $^{1}$H NMR (400 MHz, $CDCl_3$) of aldehyde **10f**. ........ S15

**Figure S9.** $^{1}$H NMR (400 MHz, $CDCl_3$) of aldehyde **10g**. ........ S16

**Figure S10.** $^{1}$H NMR (300 MHz, $CDCl_3$) of aldehyde **10h**. ........ S16

**Figure S11.** $^{1}$H NMR (300 MHz, $CDCl_3$) of aldehyde **10i**. ........ S17

**Figure S12.** $^{1}$H NMR (400 MHz, $CDCl_3$) of aldehyde **10j**. ........ S17

**Figure S13.** $^{1}$H NMR (300 MHz, $CDCl_3$) of aldehyde **10k**. ........ S18

**Figure S14.** $^{1}$H NMR (400 MHz, $CDCl_3$) of aldehyde **10l**. ........ S18

**Figure S15.** $^{1}$H NMR (300 MHz, $CDCl_3$) of aldehyde **10m**. ........ S19

**Figure S16.** $^{1}$H NMR (300 MHz, $CDCl_3$) of aldehyde **10n**. ........ S19

**Figure S17.** $^{1}$H NMR (300 MHz, $CDCl_3$) of aldehyde **10s**. ........ S20

VI. $^{1}$H NMR and $^{13}$C{$^{1}$H} NMR spectra of compounds **12**, **13** and **14** ........ S21

**Figure S18.** $^{1}$H NMR (300 MHz, $CDCl_3$) of compound **12aa**. ........ S21

**Figure S19.** $^{13}$C{$^{1}$H} NMR (75 MHz, $CDCl_3$) of compound **12aa**. ........ S21

**Figure S20.** $^{1}$H NMR (400 MHz, $CDCl_3$) of compound **12ab**. ........ S22

**Figure S21.** $^{13}$C{$^{1}$H} NMR (101 MHz, $CDCl_3$) of compound **12ab**. ........ S22

**Figure S22.** $^{1}$H NMR (400 MHz, $CDCl_3$) of compound **12ba**. ........ S23

**Figure S23.** $^{13}$C{$^{1}$H} NMR (101 MHz, $CDCl_3$) of compound **12ba**. ........ S23

**Figure S24.** $^{1}$H NMR (300 MHz, $CDCl_3$) of compound **12ca**. ........ S24

**Figure S25.** $^{13}$C{$^{1}$H} NMR (75 MHz, $CDCl_3$) of compound **12ca**. ........ S24

**Figure S26.** $^{1}$H NMR (300 MHz, $CDCl_3$) of compound **12da**. ........ S25

**Figure S27.** $^{13}$C{$^{1}$H} NMR (75 MHz, $CDCl_3$) of compound **12da**. ........ S25

**Figure S28.** $^{1}$H NMR (300 MHz, $CDCl_3$) of compound **12ea**. ........ S26

**Figure S29.** $^{13}$C{$^{1}$H} NMR (75 MHz, $CDCl_3$) of compound **12ea**. ........ S26

**Figure S30.** $^{1}$H NMR (400 MHz, $CDCl_3$) of compound **12fa**. ........ S27

**Figure S31.** $^{13}$C{$^{1}$H} NMR (101 MHz, $CDCl_3$) of compound **12fa**. ........ S27

**Figure S32.** $^{1}$H NMR (400 MHz, $CDCl_3$) of compound **12ga**. ........ S28

**Figure S33.** $^{13}$C{$^{1}$H} NMR (101 MHz, $CDCl_3$) of compound **12ga**. ........ S28

**Figure S34.** $^{1}H$ NMR (400 MHz, $CDCl_3$) of compound **12ha**. ................................................ S29
**Figure S35.** $^{13}C\{^{1}H\}$ NMR (101 MHz, $CDCl_3$) of compound **12ha**. ........................................ S29
**Figure S36.** $^{1}H$ NMR (300 MHz, $CDCl_3$) of compound **12ia**. ................................................ S30
**Figure S37.** $^{13}C\{^{1}H\}$ NMR (75 MHz, $CDCl_3$) of compound **12ia**. ........................................... S30
**Figure S38.** $^{1}H$ NMR (400 MHz, $CDCl_3$) of compound **12ja**. ................................................ S31
**Figure S39.** $^{13}C\{^{1}H\}$ NMR (101 MHz, $CDCl_3$) of compound **12ja**. ........................................ S31
**Figure S40.** $^{1}H$ NMR (400 MHz, $CDCl_3$) of compound **12ka**. ................................................ S32
**Figure S41.** $^{13}C\{^{1}H\}$ NMR (101 MHz, $CDCl_3$) of compound **12ka**. ........................................ S32
**Figure S42.** $^{1}H$ NMR (300 MHz, $CDCl_3$) of compound **12la**. ................................................ S33
**Figure S43.** $^{13}C\{^{1}H\}$ NMR (75 MHz, $CDCl_3$) of compound **12la**. ........................................... S33
**Figure S44.** $^{1}H$ NMR (400 MHz, $CDCl_3$) of compound **12ma**. .............................................. S34
**Figure S45.** $^{13}C\{^{1}H\}$ NMR (101 MHz, $CDCl_3$) of compound **12ma**. ...................................... S34
**Figure S46.** $^{1}H$ NMR (300 MHz, $CDCl_3$) of compound **12na**. ................................................ S35
**Figure S47.** $^{13}C\{^{1}H\}$ NMR (75 MHz, $CDCl_3$) of compound **12na**. ........................................... S35
**Figure S48.** $^{1}H$ NMR (400 MHz, $CDCl_3$) of compound **12oa**. ................................................ S36
**Figure S49.** $^{13}C\{^{1}H\}$ NMR (101 MHz, $CDCl_3$) of compound **12oa**. ........................................ S36
**Figure S50.** $^{1}H$ NMR (400 MHz, $CDCl_3$) of compound **12pa**. ................................................ S37
**Figure S51.** $^{13}C\{^{1}H\}$ NMR (101 MHz, $CDCl_3$) of compound **12pa**. ........................................ S37
**Figure S52.** $^{1}H$ NMR (500 MHz, $CDCl_3$) of compound **12qa**. ................................................ S38
**Figure S53.** $^{13}C\{^{1}H\}$ NMR (125 MHz, $CDCl_3$) of compound **12qa**. ........................................ S38
**Figure S54.** $^{1}H$ NMR (400 MHz, $CDCl_3$) of compound **12ra**. ................................................ S39
**Figure S55.** $^{13}C\{^{1}H\}$ NMR (101 MHz, $CDCl_3$) of compound **12ra**. ........................................ S39
**Figure S56.** $^{1}H$ NMR (400 MHz, $CDCl_3$) of compound **12sa**. ................................................ S40
**Figure S57.** $^{13}C\{^{1}H\}$ NMR (101 MHz, $CDCl_3$) of compound **12sa**. ........................................ S40
**Figure S58.** $^{1}H$ NMR (400 MHz, $CDCl_3$) of compound **13**. ................................................... S41
**Figure S59.** $^{13}C\{^{1}H\}$ NMR (101 MHz, $CDCl_3$) of compound **13**. ........................................... S41
**Figure S60.** $^{1}H$ NMR (300 MHz, $CDCl_3$) of compound **14**. ................................................... S42
**Figure S61.** $^{13}C\{^{1}H\}$ NMR (75 MHz, $CDCl_3$) of compound **14**. .............................................. S42
VII. HPLC chromatograms of compounds **12**, **13** and **14** ......................................................... S43
**Figure S62.** HPLC chromatogram of compound *rac*-**12aa**. .................................................. S43
**Figure S63.** HPLC chromatogram of compound **12aa**. ........................................................ S43
**Figure S64.** HPLC chromatogram of compound *rac*-**12ab**. .................................................. S44
**Figure S65.** HPLC chromatogram of compound **12ab**. ........................................................ S44
**Figure S66.** HPLC chromatogram of compound *rac*-**12ba**. .................................................. S45
**Figure S67.** HPLC chromatogram of compound **12ba**. ........................................................ S45
**Figure S68.** HPLC chromatogram of compound *rac*-**12ca**. .................................................. S46
**Figure S69.** HPLC chromatogram of compound **12ca**. ........................................................ S46
**Figure S70.** HPLC chromatogram of compound *rac*-**12da**. .................................................. S47
**Figure S71.** HPLC chromatogram of compound **12da**. ........................................................ S47
**Figure S72.** HPLC chromatogram of compound *rac*-**12ea**. .................................................. S48

**Figure S73.** HPLC chromatogram of compound **12ea**. ........ S48
**Figure S74.** HPLC chromatogram of compound *rac*-**12fa**. ........ S49
**Figure S75.** HPLC chromatogram of compound **12fa**. ........ S49
**Figure S76.** HPLC chromatogram of compound *rac*-**12ga**. ........ S50
**Figure S77.** HPLC chromatogram of compound **12ga**. ........ S50
**Figure S78.** HPLC chromatogram of compound *rac*-**12ha**. ........ S51
**Figure S79.** HPLC chromatogram of compound **12ha**. ........ S51
**Figure S80.** HPLC chromatogram of compound *rac*-**12ia**. ........ S52
**Figure S81.** HPLC chromatogram of compound **12ia**. ........ S52
**Figure S82.** HPLC chromatogram of compound *rac*-**12ja**. ........ S53
**Figure S83.** HPLC chromatogram of compound **12ja**. ........ S53
**Figure S84.** HPLC chromatogram of compound *rac*-**12ka**. ........ S54
**Figure S85.** HPLC chromatogram of compound **12ka**. ........ S54
**Figure S86.** HPLC chromatogram of compound *rac*-**12la**. ........ S55
**Figure S87.** HPLC chromatogram of compound **12la**. ........ S55
**Figure S88.** HPLC chromatogram of compound *rac*-**12ma**. ........ S56
**Figure S89.** HPLC chromatogram of compound **12ma**. ........ S56
**Figure S90.** HPLC chromatogram of compound *rac*-**12na**. ........ S57
**Figure S91.** HPLC chromatogram of compound **12na**. ........ S57
**Figure S92.** HPLC chromatogram of compound *rac*-**12oa**. ........ S58
**Figure S93.** HPLC chromatogram of compound **12oa**. ........ S58
**Figure S94.** HPLC chromatogram of compound *rac*-**12pa**. ........ S59
**Figure S95.** HPLC chromatogram of compound **12pa**. ........ S59
**Figure S96.** HPLC chromatogram of compound *rac*-**12qa**. ........ S60
**Figure S97.** HPLC chromatogram of compound **12qa**. ........ S60
**Figure S98.** HPLC chromatogram of compound *rac*-**12ra**. ........ S61
**Figure S99.** HPLC chromatogram of compound **12ra**. ........ S61
**Figure S100.** HPLC chromatogram of compound *rac*-**12sa**. ........ S62
**Figure S101.** HPLC chromatogram of compound **12sa**. ........ S62
**Figure S102.** HPLC chromatogram of compound *rac*-**13**. ........ S63
**Figure S103.** HPLC chromatogram of compound **13**. ........ S63
**Figure S104.** HPLC chromatogram of compound *rac*-**14**. ........ S64
**Figure S105.** HPLC chromatogram of compound **14**. ........ S64
VIII. Green chemistry metrics ........ S65
**Table S1.** E-Factor and EcoScale values for the different organocatalytic enantioselective α-nitrogenations of aldehydes and comparison with literature. ........ S65
IX. Computational details ........ S83
**Figure S106.** Computed transition states for the reaction between the enamine from **9** and **10p** and **11a**. ........ S83
X. References ........ S104

## I. General information

All solvents and reagents commercially available (Acros Organics, Alfa Aesar, Fluka, Fluorochem, Sigma Aldrich, TCI Chemicals) were used without further purification. Compounds **11** were purchased (Fluorochem). Compounds **1** (Apollo Scientific), **6** (Alfa Aesar) and **8** (trichlorohydrate, Strem Chemicals) were purchased. Optical rotations were measured in a JASCO P-1030 polarimeter at λ = 589 nm (sodium D-line) using a 5 cm cell at 20 ºC. The concentrations (*c*) are given in g/100 mL. $^{1}$H NMR spectra were recorded on Bruker AV300 (300 MHz), Bruker AV400 (400 MHz) and Bruker Avance DRX500 (500 MHz) spectrometers in proton-coupled mode at room temperature. $^{13}$C NMR spectra were recorded on Bruker AV300 (75 MHz), Bruker AV400 (101 MHz) and Bruker Avance DRX500 (125 MHz) spectrometers in proton decoupled mode at room temperature. Chemical shifts ($\delta$) are given in parts per million (ppm) using $CDCl_3$ as solvent and tetramethylsilane (TMS) as internal standard. Coupling constants (*J*) are given in Hz. Infrared (IR) spectra were obtained from neat samples with an ATR Jasco FT/IR-4100. Wavenumbers ($\tilde{\nu}$) are given in $cm^{-1}$ and the intensity is provided as very strong (vs), strong (s), weak (w) or broad (br). High-resolution mass spectrometry (HRMS-ESI-TOF) data were obtained on an Agilent 6530 LC/Q-TOF provided by the Servicio Central de Análisis of Álava (SGIker, UPV/EHU). A 50/50 mixture of 0.1% formic acid/acetonitrile with 0.1% formic acid was used as the mobile phase and an ESI Agilent Jet Stream as the ionization source. High resolution mass spectrometry (HRMS-QTOF) data were obtained on an Agilent 7200 Q-TOF in the Servicios Técnicos de Investigación (SSTTI, University of Alicante). The *ee*'s were determined on an Agilent 1100 Series HPLC equipped with an Agilent G1311A quaternary pump and an Agilent G1315B diode array detector (DAD). The employed conditions (column, mobile phase, flow rate, wavelength) are shown in each case (See 3. Characterization data). Thin layer chromatography (TLC) was carried out on Macherey-Nagel Alugram Sil G UV254 aluminum sheets coated with a 0.2 mm layer of silica gel, employing a UV light lamp (254 nm) for the detection. Flash column chromatography was performed using silica gel 60 of 40-63 µm (230-400 mesh).

## II. General experimental procedures

**Synthesis of the organocatalyst 9**. The organocatalyst **9** was synthesized by direct mono-Boc protection of (*R*,*R*)-cyclohexa-1,2-diamine using di-*tert*-butyl dicarbonate following the literature procedure.[1]

**Synthesis of aldehydes 10b-n and 10s**. Aldehydes **10a** and **10o-r** were purchased (**10a**: TCI Chemicals; **10p-q**: Alfa Aesar; **10o** and **10r**: BLDpharm). Aldehydes **10b**-**n** and **10s** were synthesized according to the literature procedure.[2] All synthesized aldehydes are described in the literature (**10b-c**,[3] **10d-f**,[2] **10g-h**,[4] **10i-m**,[2], **10n**,[5] **10s**[2]).

**General procedure for the organocatalytic enantioselective α-nitrogenation.**

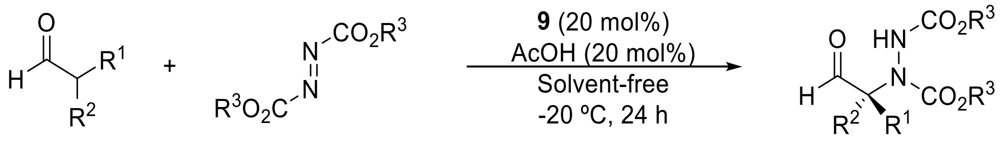


**10** **11** **12**

**Figure S1.** Enantioselective α-nitrogenation of aldehydes organocatalyzed by **8**.

A glass vial (ø 16 mm) was charged with **9** (8.6 mg, 0.04 mmol, 0.2 equiv.), acetic acid (2.3 µL, 0.04 mmol, 0.2 equiv.), azodicarboxylate **11** (0.24 mmol, 1.2 equiv.) and aldehyde **10** (0.20 mmol, 1 equiv.). The mixture was gently stirred at -20 °C under an argon atmosphere for 24 h. After this time, the reaction crude was purified by column chromatography [silica gel, ethyl acetate/hexanes (15/85, v/v)] to afford the product **12**.

Racemic samples of **12** as references for the HPLC determination of the *ee*'s, were prepared following the same experimental procedure but using an equimolar mixture of **9** and *ent*-**9**, and performing the reaction at room temperature for 8 h.

**Scaled-up synthesis of 12aa.** A glass vial (ø 16 mm) was charged with **9** (0.6 mmol, 129 mg), acetic acid (0.6 mmol, 36 mg, 34.5 µL), aldehyde **10a** (3 mmol, 402 mg, 0.40 mL) and azodicarboxylate **11a** (3.6 mmol, 727 mg, 0.73 mL). The mixture was gently stirred at -20 °C under an argon atmosphere for 24 h. After this time, the reaction crude was purified by column chromatography [silica gel, hexanes/ethyl acetate (85/15, v/v)] to afford the product **12aa** (0.95 g, 95%, 93% *ee*).

**Synthesis of oxazolidin-2-one 14**

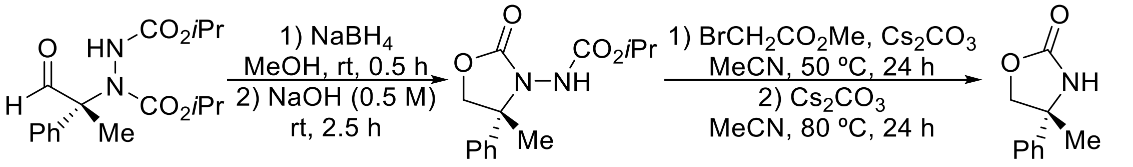


**Figure S2.** Synthesis of oxazolidin-2-one **14** from α-nitrogenated aldehyde **12aa**.

Aldehyde **12aa** (150 mg, 0.45 mmol) was placed in a round-bottom flask equipped with a magnetic bar and was dissolved in MeOH (3 mL). Then, $NaBH_4$ (22.3 mg, 0.59 mmol, 1.32 equiv.) was carefully added, and the mixture was stirred at room temperature for 0.5 h. After this time, a solution of NaOH (0.5 M in $H_2O$, 1.5 mL) was added, and the mixture was stirred at room temperature for 2.5 h. The reaction mixture was concentrated, diluted with water (3 mL) and extracted with ethyl acetate (3 x 3 mL). The combined organic layers were dried, filtered over $MgSO_4$ and evaporated under reduced pressure to give **13** (114 mg, 92%) without purification.

In a round-bottom flask, oxazolidin-2-one **13** (120 mg, 0.43 mmol) was dissolved in dry acetonitrile (5 mL) under argon. Methyl bromoacetate (82 µL, 0.86 mmol, 2 equiv.) was added, followed by $Cs_2CO_3$ (350.3 mg, 1.08 mmol, 2.5 equiv.), and the mixture was stirred at 50 ºC (heating mantle) for 24 h under argon. After this time, the reaction was quenched with saturated aqueous ammonium chloride (5 mL) and extracted with ethyl acetate (3 x 5 mL). The combined organic layers were dried, filtered over $MgSO_4$ and evaporated under reduced pressure. The crude was dissolved in dry acetonitrile (5 mL) and additional $Cs_2CO_3$ (3 equiv.) was added, stirring the mixture at 80 ºC (heating mantle) for 24 h under argon. After this time, the reaction was quenched with saturated aqueous ammonium chloride (5 mL) and extracted with ethyl acetate (3 x 5 mL). The combined organic layers were dried, filtered over $MgSO_4$ and evaporated under reduced pressure, purifying the crude by column chromatography (ethyl acetate/hexanes, 60/40) to obtain **14** (65 mg, 90%).

## III. Characterization data of compounds **12**, **13** and **14**

**(*R*)-2-[*N*,*N'*-Bis(isopropyloxycarbonyl)hydrazino]-2-phenylpropionaldehyde (12aa)**:[6]

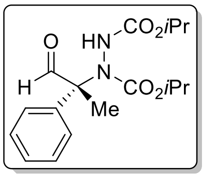

Yellow oil, 67 mg, 99% yield, 94% *ee*. $R_f$ = 0.43 (ethyl acetate/hexanes, 15/85); $\alpha_D^{20}$ = +46.7 (*c* = 1.0 in $CHCl_3$); $^1$H NMR (300 MHz, $CDCl_3$): $\delta$ = 9.75, 9.60 (2 br s, 1H), 7.62-7.28 (m, 5H), 6.41, 6.31 (2 br s, 1H), 5.04-4.85 (m, 2H), 1.79, 1.74 (2 br s, 3H), 1.33-1.10 (m, 12H) ppm; $^{13}$C{$^1$H} NMR (75 MHz, $CDCl_3$): $\delta$ = 194.2, 192.8, 156.2, 155.8, 137.1, 129.1, 128.7, 128.3, 126.9, 73.2, 71.6, 70.8, 70.3, 22.1, 21.9, 18.1, 17.6 ppm; HPLC: Chiralpak AS-H column, 2-propanol/hexanes (10/90), flow rate = 0.6 mL/min, λ = 254 nm, $t_r$ (major) = 9.90 min, $t_r$ (minor) = 10.74 min.

**(*R*)-2-[*N*,*N'*-Bis(*tert*-butoxycarbonyl)hydrazino]-2-phenylpropionaldehyde (12ab)**:[6]

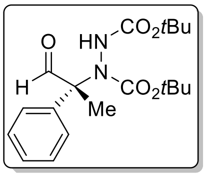

Yellow oil, 71 mg, 98% yield, 84% *ee*. $R_f$ = 0.45 (ethyl acetate/hexanes, 15/85); $\alpha_D^{20}$ = +35.0 (*c* = 1.0 in $CHCl_3$); $^1$H NMR (400 MHz, $CDCl_3$): $\delta$ = 9.71, 9.57, 9.54 (3 br s, 1H), 7.64-7.27 (m, 5H), 6.26, 6.07 (2 br s, 1H), 1.76, 1.73 (2 br s, 3H), 1.57-1.28 (m, 18H) ppm; $^{13}$C{$^1$H} NMR (101 MHz, $CDCl_3$): $\delta$ = 193.0, 155.5, 155.2, 137.6, 128.9, 128.6, 128.0, 127.1, 126.8, 83.2, 82.4, 81.6, 81.3, 72.9, 72.5, 28.2, 28.0, 18.4, 17.7 ppm; HPLC: Chiralpak AS-H column, ethanol/hexanes (10/90), flow rate = 0.6 mL/min, λ = 280 nm, $t_r$ (major) = 16.64 min, $t_r$ (minor) = 18.82 min.

**(*R*)-2-[*N*,*N'*-Bis(isopropyloxycarbonyl)hydrazino]-2-(3'methylphenyl)propionaldehyde (12ba)**:

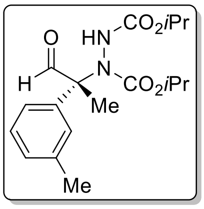

Colorless oil, 67 mg, 95% yield, 96% *ee*. $R_f$ = 0.44 (ethyl acetate/hexanes, 15/85); $\alpha_D^{20}$ = +77.8 (*c* = 1.0 in $CHCl_3$); $^1$H NMR (400 MHz, $CDCl_3$): $\delta$ = 9.74, 9.57 (2 br s, 1H), 7.41-7.22 (m, 2H), 7.20-7.08 (m, 2H), 6.30, 6.11 (2 br s, 1H), 5.03-4.85 (m, 2H), 2.36, 2.35 (2 br s, 3H), 1.78, 1.74 (2 br s, 3H), 1.32-1.12 (m, 12H) ppm; $^{13}$C{$^1$H} NMR (101 MHz, $CDCl_3$): $\delta$ = 192.9, 192.6, 156.6, 155.8, 138.9, 138.8, 129.1, 127.6, 124.1, 73.3, 71.6, 70.3, 22.4, 22.0, 21.9, 21.8, 17.9, 17.6 ppm; IR (Infrared film) $\tilde{\nu}$ = 3290 (br), 2981 (w), 2935 (w), 2870 (w), 1720 (vs), 1608 (w), 1508 (w), 1462 (w), 1377 (s), 1319 (w), 1242 (s), 1180 (w), 1146 (w), 1103 (s), 1053 (w), 1034 (w), 933 (w), 914 (w), 775 (w), 706 (w) cm$^{-1}$; HRMS (ESI-TOF) *m/z* calcd. for $C_{18}H_{27}N_2O_5$ ([*M*+H]$^+$): 351.1920, found: 351.1921; HPLC: Chiralpak AS-H column, ethanol/hexanes (5/95), flow rate = 1.0 mL/min, λ = 280 nm, $t_r$ (major) = 9.19 min, $t_r$ (minor) = 10.53 min.

**(*R*)-2-[*N*,*N'*-Bis(isopropyloxycarbonyl)hydrazino]-2-(4'methylphenyl)propionaldehyde (12ca)**:[6]

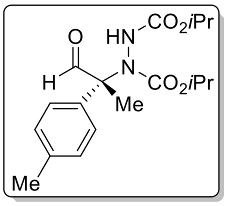

Orange oil, 69 mg, 98% yield, 92% *ee*. $R_f$ = 0.44 (ethyl acetate/hexanes, 15/85); $\alpha_D^{20}$ = +51.2 (*c* = 1.0 in $CHCl_3$); $^1$H NMR (300 MHz, $CDCl_3$): $\delta$ = 9.72, 9.56 (2 br s, 1H), 7.49-7.29 (m, 1H), 7.26-7.13 (m, 3H), 6.35, 6.23 (2 br s, 1H), 5.01-4.85 (m, 2H), 2.34, 2.33 (2 br s, 3H), 1.78, 1.72 (2 br s, 3H), 1.29-1.16 (m, 12H) ppm; $^{13}$C{$^1$H} NMR (75 MHz, $CDCl_3$): $\delta$ = 192.7, 192.6, 156.2, 155.9, 138.2, 133.9, 129.8, 129.5, 126.9, 73.1, 71.6, 70.3, 22.1, 21.9, 21.8, 21.2, 17.7, 17.4 ppm; HPLC: Chiralpak IB column, ethanol/hexanes (5/95), flow rate = 1.0 mL/min, λ = 280 nm, $t_r$ (major) = 7.75 min, $t_r$ (minor) = 8.21 min.

**(*R*)-2-[*N*,*N*'-Bis(isopropyloxycarbonyl)hydrazino]-2-(4'methoxyphenyl)propionaldehyde (12da)**:

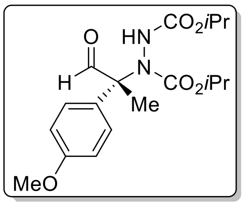

Yellow oil, 72 mg, 99% yield, 97% *ee*. $R_f$ = 0.23 (ethyl acetate/hexanes, 15/85); $\alpha_D^{20}$ = +50.7 (*c* = 1.0 in $CHCl_3$); $^1H$ NMR (300 MHz, $CDCl_3$): $\delta$ = 9.69, 9.55 (2 br s), 7.54-7.22 (m, 2H), 6.97-6.82 (m, 2H), 6.35, 6.25 (2 br s, 1H), 5.03-4.84 (m, 2H), 3.80 (s, 3H), 1.78, 1.72 (2 br s, 3H), 1.30-1.17 (m, 12H) ppm; $^{13}C\{^1H\}$ NMR (75 MHz, $CDCl_3$): $\delta$ = 193.5, 192.6, 159.5, 156.1, 155.8, 128.9, 128.3, 114.5, 114.1, 72.8, 71.5, 70.3, 70.2, 55.4, 22.1, 21.9, 17.7, 17.4 ppm; IR (infrared film): $\tilde{\nu}$ = 3309 (br), 2981 (w), 2881 (w), 2835 (w), 1724 (vs), 1608 (w), 1512 (w), 1462 (w), 1377 (w), 1311 (w), 1246 (s), 1180 (w), 1107 (w), 1030 (w), 910 (w), 829 (w), 768 (w) $cm^{-1}$; HRMS (ESI-TOF) *m/z* calcd. for $C_{18}H_{26}N_2O_6Na$ ([*M*+Na]$^+$): 389.1689, found: 389.1688; HPLC: Chiralpak AS-H column, EtOH/hexanes (10/90), flow rate = 1.0 mL/min, λ = 280 nm, $t_r$ (major) = 8.44 min, $t_r$ (minor) = 9.72 min.

**(*R*)-2-[*N*,*N*'-Bis(isopropyloxycarbonyl)hydrazino]-2-biphenylpropionaldehyde (12ea)**:

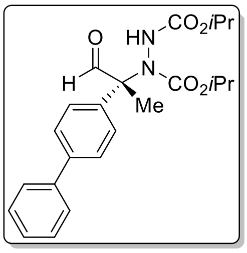

Colorless oil, 79 mg, 96% yield, 95% *ee*. $R_f$ = 0.51 (ethyl acetate/hexanes, 15/85); $\alpha_D^{20}$ = +30.3 (*c* = 1.0 in $CHCl_3$); $^1H$ NMR (300 MHz, $CDCl_3$): $\delta$ = 9.78, 9.63 (2 br s, 1H), 7.68-7.54 (m, 5H), 7.49-7.41 (m, 3H), 7.39-7.32 (m, 1H), 6.41, 6.26 (2 br s, 1H), 5.02-4.88 (m, 2H), 1.83, 1.80, 1.77 (3 br s, 3H), 1.32-1.12 (m, 12H) ppm; $^{13}C\{^1H\}$ NMR (75 MHz, $CDCl_3$): $\delta$ = 193.9, 192.7, 156.1, 155.9, 141.1, 140.4, 136.2, 136.1, 129.0, 127.7, 127.4, 127.3, 127.2, 73.1, 71.7, 70.9, 70.4, 22.1, 22.0, 21.9, 21.8, 18.2, 17.7 ppm; IR (infrared film): $\tilde{\nu}$ = 3302 (br), 2985 (w), 2943 (w), 2885 (w), 2827 (w), 1724 (vs), 1512 (w), 1493 (w), 1462 (w), 1377 (s), 1315 (w), 1242 (s), 1180 (w), 1103 (s), 1057 (w), 1034 (w), 910 (w), 837 (w), 764 (w), 733 (w), 694 (w) $cm^{-1}$; HRMS (ESI-TOF) *m/z* calcd. for $C_{23}H_{29}N_2O_5$ ([*M*+H]$^+$): 413.2076, found: 413.2079; HPLC: Chiralpak IB column, 2-propanol/hexanes (5/95), flow rate = 0.7 mL/min, λ = 280 nm, $t_r$ (major) = 10.32 min, $t_r$ (minor) = 13.61 min.

**(*R*)-2-[*N*,*N*'-Bis(isopropyloxycarbonyl)hydrazino]-2-(4'fluorophenyl)propionaldehyde (12fa)**:[6]

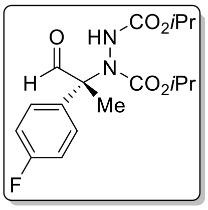

Colorless oil, 66 mg, 93% yield, 98% *ee*. $R_f$ = 0.53 (ethyl acetate/hexanes, 15/85); $\alpha_D^{20}$ = +26.4 (*c* = 1.0 in $CHCl_3$); $^1H$ NMR (400 MHz, $CDCl_3$): $\delta$ = 9.70, 9.58 (2 br s, 1H), 7.55-7.27 (m, 4H), 6.35 (br s, 1H), 5.03-4.89 (m, 2H), 1.75, 1.69 (2 br s, 3H), 1.31-1.16 (m, 12H) ppm; $^{13}C\{^1H\}$ NMR (101 MHz, $CDCl_3$): $\delta$ = 192.9, 162.6 (d, *J* = 248.0 Hz), 156.1, 155.7, 133.0, 130.9 (d, *J* = 7.8 Hz), 128.8, 128.7 (d, *J* = 6.9 Hz), 116.0, 115.8, 115.3 (d, *J* = 21.9 Hz), 72.5, 71.8, 70.5, 22.1, 22.0, 21.9, 18.2 ppm; HPLC: Chiralpak AS-H column, ethanol/hexanes (5/95), flow rate = 0.8 mL/min, λ = 210 nm, $t_r$ (major) = 5.92 min, $t_r$ (minor) = 7.27 min.

**(*R*)-2-[*N*,*N*'-Bis(isopropyloxycarbonyl)hydrazino]-2-(2'chlorophenyl)propionaldehyde (12ga)**:[6]

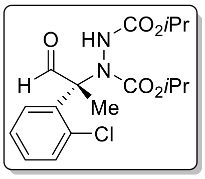

Colorless oil, 69 mg, 93% yield, 96% *ee*. $R_f$ = 0.50 (ethyl acetate/hexanes, 15/85); $\alpha_D^{20}$ = -8.6 (*c* = 1.0 in $CHCl_3$); $^1H$ NMR (400 MHz, $CDCl_3$): $\delta$ = 10.14, 10.08 (2 br s, 1H), 7.48-7.34 (m, 2H), 7.32-7.25 (m, 2H), 6.71, 6.59, 6.48 (3 br s, 1H), 5.04-4.84 (m, 2H), 1.84, 1.78 (2 br s, 3H), 1.35-1.12 (m, 12H) ppm; $^{13}C\{^1H\}$ NMR (101 MHz, $CDCl_3$): $\delta$ = 195.6, 156.2, 154.9, 135.8, 131.9, 131.7, 129.6, 129.3, 127.6, 73.1, 71.3, 70.8, 70.3, 22.1, 22.0, 21.9, 19.5 ppm; HPLC: Chiralpak AS-H column, ethanol/hexanes (5/95), flow rate = 0.8 mL/min, λ = 210 nm, $t_r$ (major) = 9.95 min, $t_r$ (minor) = 12.26 min.

**(*R*)-2-[*N*,*N'*-Bis(isopropyloxycarbonyl)hydrazino]-2-(3'chlorophenyl)propionaldehyde (12ha)**:[6]

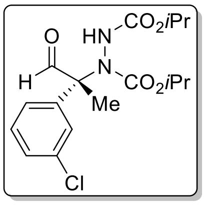

Colorless oil, 67 mg, 91% yield, 90% *ee*. $R_f$ = 0.50 (ethyl acetate/hexanes, 15/85); $\alpha_D^{20}$ = +10.9 (*c* = 1.0 in $CHCl_3$); $^1$H NMR (400 MHz, $CDCl_3$): $\delta$ = 9.73, 9.60 (2 br s, 1H), 7.63-7.27 (m, 4H), 6.40 (br s, 1H), 5.04-4.88 (m, 2H), 1.75 (br s, 3H), 1.33-1.14 (m, 12H) ppm; $^{13}$C{$^1$H} NMR (101 MHz, $CDCl_3$): $\delta$ = 193.8, 192.7, 156.2, 155.7, 139.6, 135.0, 130.2, 128.4, 127.1, 125.2, 72.7, 71.9, 70.6, 22.1, 21.9, 18.7, 18.3 ppm; HPLC: Chiralpak AS-H column, ethanol/hexanes (5/95), flow rate = 0.6 mL/min, λ = 280 nm, $t_r$ (major) = 10.01 min, $t_r$ (minor) = 12.56 min.

**(*R*)-2-[*N*,*N'*-Bis(isopropyloxycarbonyl)hydrazino]-2-(4'chlorophenyl)propionaldehyde (12ia)**:

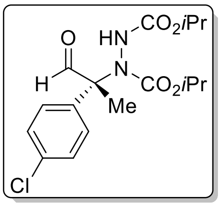

Colorless oil, 67 mg, 90% yield, 99% *ee*. $R_f$ = 0.50 (ethyl acetate/hexanes, 15/85); $\alpha_D^{20}$ = +12.0 (*c* = 1.0 in $CHCl_3$); $^1$H NMR (300 MHz, $CDCl_3$): $\delta$ = 9.71, 9.58 (2 br s, 1H), 7.61-7.28 (m, 4H), 6.34, 6.23 (2 br s, 1H), 5.01-4.87 (m, 2H), 1.75, 1.70 (2 br s, 3H), 1.32-1.12 (m, 12H) ppm; $^{13}$C{$^1$H} NMR (75 MHz, $CDCl_3$): $\delta$ = 193.8, 193.3, 156.3, 155.8, 136.1, 135.9, 134.3, 129.2, 128.8, 128.4, 72.6, 71.8, 70.6, 70.3, 22.1, 21.9, 18.5 ppm; IR (infrared film): $\tilde{\nu}$ = 3313 (br), 2981 (w), 2935 (w), 2870 (w), 1720 (vs), 1593 (w), 1493 (w), 1462 (w), 1381 (s), 1319 (w), 1246 (s), 1176 (w), 1146 (w), 1099 (s), 1061 (w), 1014 (w), 910 (w), 825 (w), 768 (w), 733 (w) cm$^{-1}$; HRMS (ESI-TOF) *m/z* calcd. for $C_{17}H_{24}ClN_2O_5$ ([*M*+H]$^+$): 371.1373, found: 371.1377; HPLC: Chiralpak AS-H column, 2-propanol/hexanes (5/95), flow rate = 0.6 mL/min, λ = 280 nm, $t_r$ (major) = 28.83 min, $t_r$ (minor) = 31.24 min.

**(*R*)-2-[*N*,*N'*-Bis(isopropyloxycarbonyl)hydrazino]-2-(4'bromophenyl)propionaldehyde (12ja)**:[6]

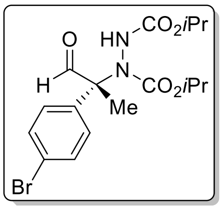

Colorless oil, 79 mg, 95% yield, 98% *ee*. $R_f$ = 0.39 (ethyl acetate/hexanes, 15/85); $\alpha_D^{20}$ = +31.6 (*c* = 1.0 in $CHCl_3$); $^1$H NMR (400 MHz, $CDCl_3$): $\delta$ = 9.70, 9.58 (2 br s, 1H), 7.56-7.43 (m, 3H), 7.42-7.28 (m, 1H), 6.36, 6.24 (2 br s, 1H), 5.02-4.88 (m, 2H), 1.75, 1.69 (2 br s, 3H), 1.31-1.17 (m, 12H) ppm; $^{13}$C{$^1$H} NMR (101 MHz, $CDCl_3$): $\delta$ = 192.8, 155.9, 155.8, 136.6, 132.1, 131.8, 131.6, 128.7, 128.0, 122.5, 72.7, 71.9, 71.2, 70.6, 22.1, 21.9, 18.6 ppm; HPLC: Chiralpak AD-H column, ethanol/hexanes (5/95), flow rate = 0.7 mL/min, λ = 230 nm, $t_r$ (major) = 13.22 min, $t_r$ (minor) = 15.66 min.

**(*R*)-2-[*N*,*N'*-Bis(isopropyloxycarbonyl)hydrazino]-2-(4'nitrophenyl)propionaldehyde (12ka)**:[4]

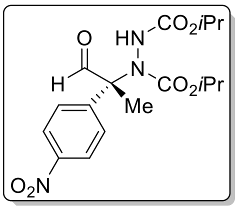

Yellow oil, 65 mg, 85% yield, 77% *ee*. $R_f$ = 0.15 (ethyl acetate/hexanes, 15/85); $\alpha_D^{20}$ = +28.1 (*c* = 1.0 in $CHCl_3$); $^1$H NMR (400 MHz, $CDCl_3$): $\delta$ = 9.76, 9.67 (2 br s, 1H), 8.31-8.16 (m, 2H), 7.98-7.48 (m, 2H), 6.76, 6.67 (2 br s, 1H), 5.12-4.84 (m, 2H), 1.75, 1.72 (2 br s, 3H), 1.38-1.00 (m, 12H) ppm; $^{13}$C{$^1$H} NMR (101 MHz, $CDCl_3$): $\delta$ = 194.3, 193.4, 156.7, 155.4, 147.5, 145.4, 128.4, 127.8, 123.8, 72.4, 72.1, 70.8, 22.1, 21.9, 21.8, 20.2, 19.5 ppm; HPLC: Chiralpak AD-H column, 2-propanol/hexanes (5/95), flow rate = 1.0 mL/min, λ = 230 nm, $t_r$ (minor) = 37.21 min, $t_r$ (major) = 41.08 min.

**(*R*)-2-[*N*,*N'*-Bis(isopropyloxycarbonyl)hydrazino]-2-(2'naphthyl)propionaldehyde (12la)**:[6]

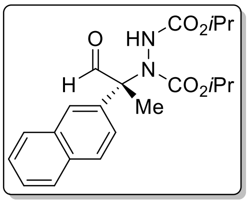


Colorless oil, 73 mg, 94% yield, 80% *ee*. $R_f$ = 0.51 (ethyl acetate/hexanes, 15/85); $\alpha_D^{20}$ = +27.2 (*c* = 1.0 in $CHCl_3$); $^1H$ NMR (300 MHz, $CDCl_3$): *δ* = 9.86, 9.70 (2 br s, 1H), 8.05-7.67 (m, 4H), 7.66-7.36 (m, 3H), 6.40 (br s, 1H), 5.04-4.84 (m, 2H), 1.92, 1.87, 1.85 (3 br s, 3H), 1.33-1.12 (m, 12H) ppm; $^{13}C\{^1H\}$ NMR (75 MHz, $CDCl_3$): *δ* = 194.0, 192.9, 156.3, 155.9, 134.6, 133.4, 132.9, 128.9, 128.4, 127.7, 126.8, 126.6, 124.5, 73.4, 71.7, 70.4, 22.1, 22.0, 21.9, 18.3, 17.8 ppm; HPLC: Chiralpak AD-H column, 2-propanol/hexanes (10/90), flow rate = 0.8 mL/min, λ = 280 nm, $t_r$ (major) = 16.64 min, $t_r$ (minor) = 22.45 min.

**(*R*)-2-[*N*,*N'*-Bis(isopropyloxycarbonyl)hydrazino]-2-phenylbutyraldehyde (12ma)**:

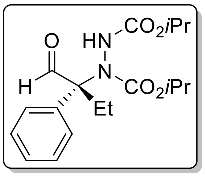


Yellow oil, 69 mg, 99% yield, 97% *ee*. $R_f$ = 0.43 (ethyl acetate/hexanes, 15/85); $\alpha_D^{20}$ = +84.1 (*c* = 1.0 in $CHCl_3$); $^1H$ NMR (400 MHz, $CDCl_3$): *δ* = 9.81, 9.59 (2 br s, 1H), 7.67-7.28 (m, 5H), 6.31, 6.13 (2 br s, 1H), 5.08-4.79 (m, 2H), 2.66-1.92 (2 m, 2H), 1.38-1.09 (m, 12H), 0.97-0.86 (m, 3H) ppm; $^{13}C\{^1H\}$ NMR (101 MHz, $CDCl_3$): *δ* = 192.9, 156.3, 155.6, 136.5, 136.3, 128.8, 128.0, 127.5, 127.1, 125.8, 71.4, 70.6, 70.1, 25.0, 24.8, 22.1, 22.0, 8.8 ppm; IR (infrared film): $\tilde{\nu}$ = 3305 (br), 2981 (w), 2942 (w), 2881 (w), 1709 (vs), 1508 (w), 1462 (w), 1377 (s), 1315 (w), 1242 (s), 1180 (w), 1146 (w), 1103 (s), 1034 (w), 941 (w), 918 (w), 845 (w), 760 (s), 698 (s) $cm^{-1}$; HRMS (ESI-TOF) *m/z* calcd. for $C_{18}H_{27}N_2O_5$ ([*M*+H]$^+$): 351.1920, found: 351.1922; HPLC: Chiralpak AS-H column, ethanol/hexanes (5/95), flow rate = 0.9 mL/min, λ = 230 nm, $t_r$ (major) = 8.08 min, $t_r$ (minor) = 9.10 min.

**(*R*)-1-[*N*,*N'*-Bis(isopropyloxycarbonyl)hydrazino]-1,2,3,4-tetrahydronaphthalen-1-carbaldehyde (12na)**:

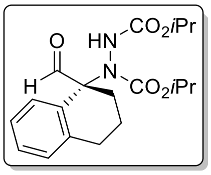


Yellow oil, 67 mg, 93% yield, 90% *ee*. $R_f$ = 0.47 (ethyl acetate/hexanes, 15/85); $\alpha_D^{20}$ = +40.2 (*c* = 1.0 in $CHCl_3$); $^1H$ NMR (300 MHz, $CDCl_3$): *δ* = 9.81, 9.54 (2 br s, 1H), 7.51, 7.33 (2 br s, 1H), 7.26-7.10 (m, 3H), 6.24, 6.01 (2 br s, 1H), 5.03-4.84 (m, 2H), 2.88-2.51 (m, 3H), 2.00, 1.88 (2 br s, 3H), 1.30-1.12 (m, 12H) ppm; $^{13}C\{^1H\}$ NMR (75 MHz, $CDCl_3$): *δ* = 192.1, 191.6, 155.7, 140.1, 139.1, 130.2, 129.9, 128.9, 128.2, 126.7, 126.2, 71.8, 71.4, 70.3, 29.8, 29.6, 27.6, 21.9, 20.2 ppm; IR (infrared film): $\tilde{\nu}$ = 3294 (br), 2981 (w), 2943 (w), 2881 (w), 2831 (w), 1724 (vs), 1512 (w), 1458 (w), 1377 (s), 1319 (w), 1242 (s), 1180 (w), 1107 (s), 1038 (w), 914 (w), 837 (w), 733 (vs) $cm^{-1}$; HRMS (ESI-TOF) *m/z* calcd. for $C_{19}H_{27}N_2O_5$ ([*M*+H]$^+$): 363.1920, found: 363.1925; HPLC: Chiralpak AS-H column, 2-propanol/hexanes (5/95), flow rate = 1.0 mL/min, λ = 254 nm, $t_r$ (major) = 17.06 min, $t_r$ (minor) = 24.43 min.

**(*R*)-2-[*N*,*N'*-Bis(isopropyloxycarbonyl)hydrazino]-cyclamen aldehyde (12oa)**:

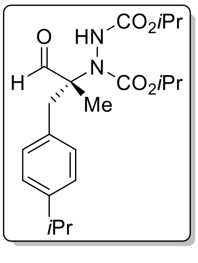


Colorless oil, 75 mg, 96% yield, 89% *ee*. $R_f$ = 0.35 (ethyl acetate/hexanes, 15/85); $\alpha_D^{20}$ = +49.6 (*c* = 1.0 in $CHCl_3$); $^1H$ NMR (400 MHz, $CDCl_3$): *δ* = 9.69, 9.58 (2 br s, 1H), 7.25-7.13 (m, 2H), 7.10-6.89 (m, 2H), 5.45, 5.22 (2 br s, 1H), 5.10-4.83 (m, 2H), 3.53-2.73 (m, 3H), 1.35-1.16 (m, 21H) ppm; $^{13}C\{^1H\}$ NMR (101 MHz, $CDCl_3$): *δ* = 199.1, 198.4, 156.0, 155.7, 147.9, 147.7, 134.0, 133.6, 130.2, 130.1, 127.0, 126.8, 71.1, 70.4, 70.1, 37.4, 33.8, 24.1, 24.0, 22.0, 18.3 ppm; IR (infrared film): $\tilde{\nu}$ = 3317 (br), 2978 (w), 2877 (w), 2816 (w), 1732 (vs), 1705 (vs), 1512 (w), 1466 (w), 1381 (s), 1315 (w), 1234 (s), 1180 (w), 1139 (w), 1057 (w), 1030 (w), 918 (w), 829 (w), 768 (w), 733 (w) $cm^{-1}$; HRMS (ESI-TOF) *m/z* calcd. for $C_{21}H_{33}N_2O_5$ ([*M*+H]$^+$): 393.2389, found: 393.2392; HPLC: Chiralpak AS-H column, ethanol/hexanes (5/95), flow rate = 0.8 mL/min, λ = 230 nm, $t_r$ (major) = 8.00 min, $t_r$ (minor) = 8.86 min.

**(*R*)-2-[*N*,*N'*-Bis(isopropyloxycarbonyl)hydrazino]-2-ethylpropionaldehyde (12pa)**:

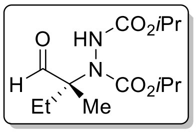

Yellow oil, 56 mg, 97% yield, 95% *ee*. $R_f$ = 0.48 (ethyl acetate/hexanes, 15/85); $\alpha_D^{20}$ = +29.2 (*c* = 1.0 in $CHCl_3$); $^1H$ NMR (400 MHz, $CDCl_3$): $\delta$ = 9.55, 9.50, 9.48 (3 br s, 1H), 6.49 (br s, 1H), 5.08-4.87 (m, 2H), 1.98-1.64 (m, 2H), 1.31-1.21 (m, 15H), 0.92-0.82 (m, 3H) ppm; $^{13}C\{^1H\}$ NMR (101 MHz, $CDCl_3$): $\delta$ = 199.1, 156.6, 155.4, 71.3, 70.6, 70.3, 28.5, 26.7, 26.3, 22.1, 17.8, 17.3, 8.1 ppm; IR (infrared film): $\tilde{\nu}$ = 3313 (br), 2978 (w), 2877 (w), 2819 (w), 1728 (vs), 1705 (vs), 1512 (w), 1462 (w), 1381 (s), 1319 (w), 1242 (s), 1180 (w), 1103 (vs), 1030 (w), 914 (w), 837 (w), 764 (w), 660 (w) $cm^{-1}$; HRMS (ESI-TOF) *m/z* calcd. for $C_{13}H_{25}N_2O_5$ ([*M*+H]$^+$): 289.1763, found: 289.1766; HPLC: Chiralpak AD-H column, 2-propanol/hexanes (5/95), flow rate = 1.0 mL/min, λ = 280 nm, $t_r$ (major) = 9.13 min, $t_r$ (minor) = 10.50 min.

**(*R*)-2-[*N*,*N'*-Bis(isopropyloxycarbonyl)hydrazino]-2-(*n*-propyl)-propionaldehyde (12qa)**:[6]

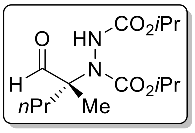

Yellow oil, 59 mg, 98% yield, 94% *ee*. $R_f$ = 0.48 (ethyl acetate/hexanes, 15/85); $\alpha_D^{20}$ = +28.1 (*c* = 1.0 in $CHCl_3$); $^1H$ NMR (500 MHz, $CDCl_3$): $\delta$ = 9.54, 9.52, 9.47 (3 br s, 1H), 6.41 (br s, 1H), 5.06-4.87 (m, 2H), 1.93-1.59 (m, 3H), 1.32-1.22 (m, 16H), 0.97-0.85 (m, 3H) ppm; $^{13}C\{^1H\}$ NMR (125 MHz, $CDCl_3$): $\delta$ = 199.0, 156.6, 155.7, 71.3, 70.3, 69.9, 35.3, 35.9, 22.1, 18.4, 18.0, 17.0, 16.8, 14.6 ppm; HPLC: Chiralpak AD-H column, *i*-PrOH/hexanes (5/95), flow rate = 0.6 mL/min, λ = 210 nm, $t_r$ (major) = 20.65 min, $t_r$ (minor) = 21.54 min.

**(*R*)-2-[*N*,*N'*-Bis(isopropyloxycarbonyl)hydrazino]-2-(*n*-octyl)-propionaldehyde (12ra)**:

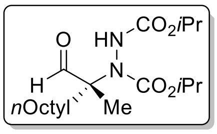

Yellow oil, 72 mg, 97% yield, 86% *ee*. $R_f$ = 0.61 (ethyl acetate/hexanes, 15/85); $\alpha_D^{20}$ = +22.0 (*c* = 1.0 in $CHCl_3$); $^1H$ NMR (400 MHz, $CDCl_3$): $\delta$ = 9.55, 9.52, 9.47, 9.43 (4 br s, 1H), 6.43 (br s, 1H), 5.07-4.84 (m, 2H), 1.94-1.56 (m, 3H), 1.36-1.19 (m, 26H), 0.92-0.84 (m, 3H) ppm; $^{13}C\{^1H\}$ NMR (101 MHz, $CDCl_3$): $\delta$ = 199.1, 198.3, 156.6, 155.7, 71.2, 70.3, 69.8, 34.0, 33.7, 31.9, 30.1, 29.5, 29.3, 23.6, 22.7, 22.0, 18.3, 17.9, 14.2 ppm; IR (infrared film): $\tilde{\nu}$ = 3313 (br), 2981 (w), 2931 (w), 2861 (w), 1728 (vs), 1705 (vs), 1512 (w), 1462 (w), 1381 (s), 1315 (w), 1242 (s), 1180 (w), 1107 (s), 1030 (w), 914 (w), 837 (w), 764 (w), 733 (w) $cm^{-1}$; HRMS (ESI-TOF) *m/z* calcd. for $C_{19}H_{37}N_2O_5$ ([*M*+H]$^+$): 373.2702, found: 373.2705; HPLC: Chiralpak AD-H column, 2-propanol/hexanes (5/95), flow rate = 1.0 mL/min, λ = 230 nm, $t_r$ (major) = 11.83 min, $t_r$ (minor) = 13.22 min.

**(*R*)-2-[*N*,*N'*-Bis(isopropyloxycarbonyl)hydrazino]-2-cyclohexylpropionaldehyde (12sa)**:

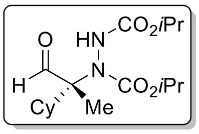

Yellow oil, 66 mg, 96% yield, 82% *ee*. $R_f$ = 0.57 (ethyl acetate/hexanes, 15/85); $\alpha_D^{20}$ = +25.5 (*c* = 1.0 in $CHCl_3$); $^1H$ NMR (400 MHz, $CDCl_3$): $\delta$ = 9.61, 9.51, 9.47 (3 br s, 1H), 6.32, 6.26 (2 br s, 1H), 5.06-4.88 (m, 2H), 2.21-1.78 (m, 3H), 1.73-1.54 (m, 2H), 1.32-1.16 (m, 18H), 1.14-0.94 (m, 3H) ppm; $^{13}C\{^1H\}$ NMR (101 MHz, $CDCl_3$): $\delta$ = 197.9, 156.5, 156.0, 72.5, 72.2, 71.3, 70.7, 70.3, 43.5, 42.8, 29.8, 28.8, 27.7, 26.9, 26.7, 22.1, 16.3 ppm; IR (infrared film): $\tilde{\nu}$ = 3305 (br), 2981 (w), 2931 (w), 2858 (w), 1720 (vs), 1701 (vs), 1516 (w), 1458 (w), 1377 (s), 1311 (w), 1238 (s), 1176 (w), 1107 (s), 1030 (w), 914 (w), 845 (w), 764 (w), 733 (w) $cm^{-1}$; HRMS (ESI-TOF) *m/z* calcd. for $C_{17}H_{31}N_2O_5$ ([*M*+H]$^+$): 343.2233, found: 343.2237; HPLC: Chiralpak IB column, ethanol/hexanes (10/90), flow rate = 1.0 mL/min, λ = 210 nm, $t_r$ (minor) = 9.00 min, $t_r$ (major) = 24.49 min.

**(*R*)-3-Isopropoxycarbonylamino-4-methyl-4-phenyl-oxazolidin-2-one (13)**:

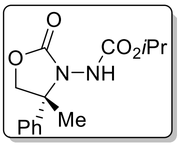


Colorless oil, 114 mg, 92% yield, 92% *ee*. $R_f$ = 0.48 (ethyl acetate/hexanes, 30/70); $\alpha_D^{20}$ = -82.3 (*c* = 1.0 in $CHCl_3$); $^1$H NMR (400 MHz, $CDCl_3$): $\delta$ = 7.45-7.34 (m, 5H), 6.15 (br s, 1H), 5.02-4.89 (m, 1H), 4.40 (d, *J* = 8.7 Hz, 1H), 4.37 (d, *J* = 8.7 Hz, 1H), 1.82 (s, 3H), 1.27-1.21 (m, 6H) ppm; $^{13}C\{^1H\}$ NMR (101 MHz, $CDCl_3$): $\delta$ = 156.2, 155.5, 140.6, 129.2, 128.6, 125.9, 125.8, 76.2, 70.6, 70.1, 63.9, 22.0, 21.9, 21.8 ppm; IR (infrared film): $\tilde{\nu}$ = 3271 (br), 2981 (w), 2939 (w), 1774 (vs), 1732 (vs), 1500 (w), 1454 (w), 1408 (w), 1381 (w), 1242 (vs), 1146 (w), 1107 (s), 1014 (s), 930 (w), 764 (s), 698 (s) $cm^{-1}$; HRMS (QTOF) *m/z* calcd. for $C_{14}H_{19}N_2O_4$ ([*M*+H]$^+$): 279.1345, found: 279.1329; HPLC: ChiralCel OD-H column, 2-propanol/hexanes (10/90), flow rate = 1.0 mL/min, λ = 210 nm, $t_r$ (minor) = 14.42 min, $t_r$ (major) = 16.64 min.

**(*R*)-(–)-4-Methyl-4-phenyl-oxazolidin-2-one (14)**:[2]

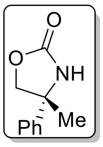


Yellow oil, 65 mg, 90% yield, 93% *ee*. $R_f$ = 0.31 (ethyl acetate/hexanes, 60/40); $\alpha_D^{20}$ = -89.8 (*c* = 1.0 in $CHCl_3$); $^1$H NMR (300 MHz, $CDCl_3$): $\delta$ = 7.44-7.27 (m, 5H), 6.47 (br s, 1H), 4.38 (d, *J* = 8.3 Hz, 1H), 4.34 (d, *J* = 8.4 Hz, 1H), 1.75 (s, 3H) ppm; $^{13}C\{^1H\}$ NMR (75 MHz, $CDCl_3$): $\delta$ = 159.4, 143.6, 129.1, 128.1, 124.7, 78.2, 60.6, 27.9 ppm; HPLC: ChiralCel OD-H column, 2-propanol/hexanes (10/90), flow rate = 1.0 mL/min, λ = 210 nm, $t_r$ (minor) = 13.18 min, $t_r$ (major) = 16.71 min.

## IV. $^{1}$H NMR spectrum of **9**

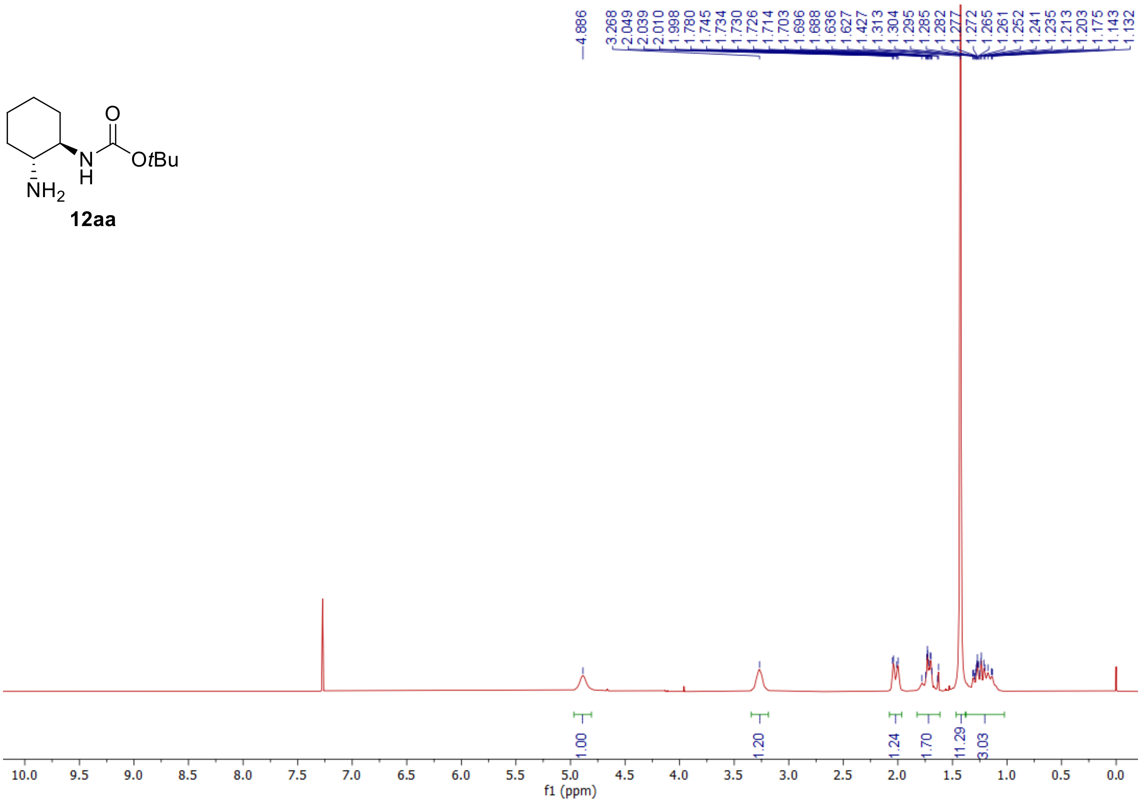


**Figure S3.** $^{1}$H NMR (300 MHz, $CDCl_3$) of organocatalyst **9**.

## V. $^{1}$H NMR spectra of **10b-n** and **10s**

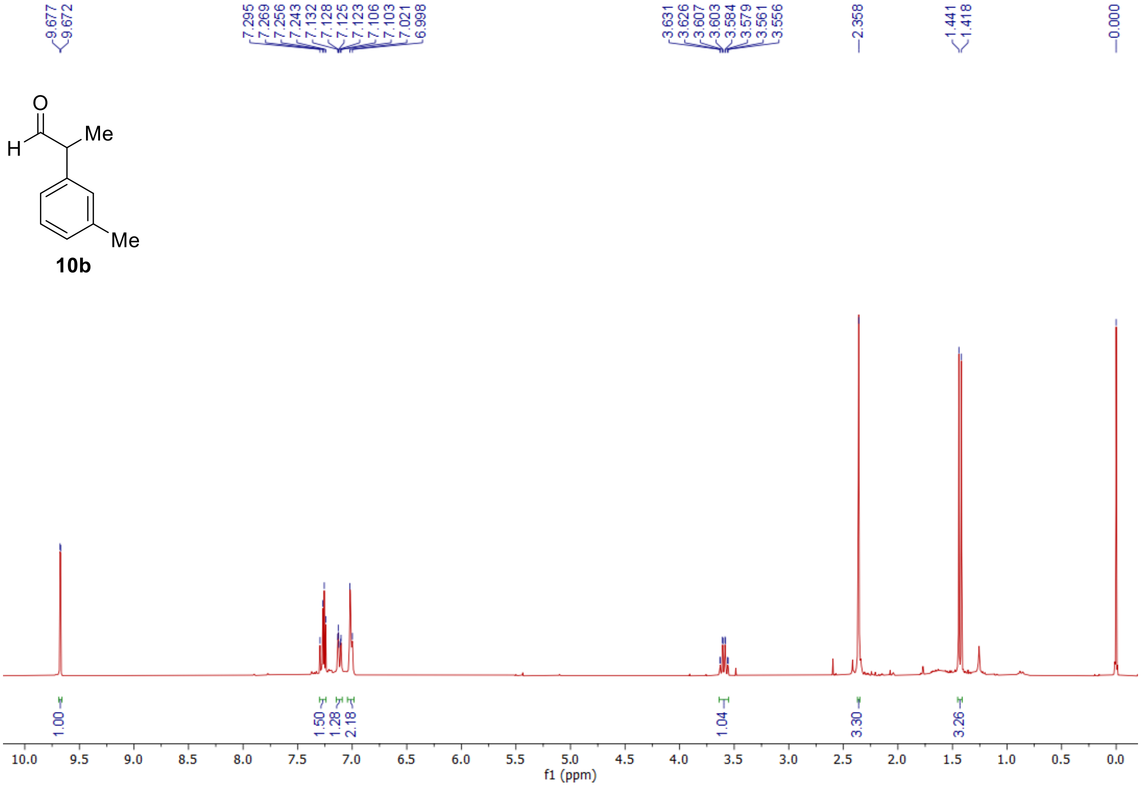


**Figure S4.** $^{1}$H NMR (300 MHz, $CDCl_3$) of aldehyde **10b**.

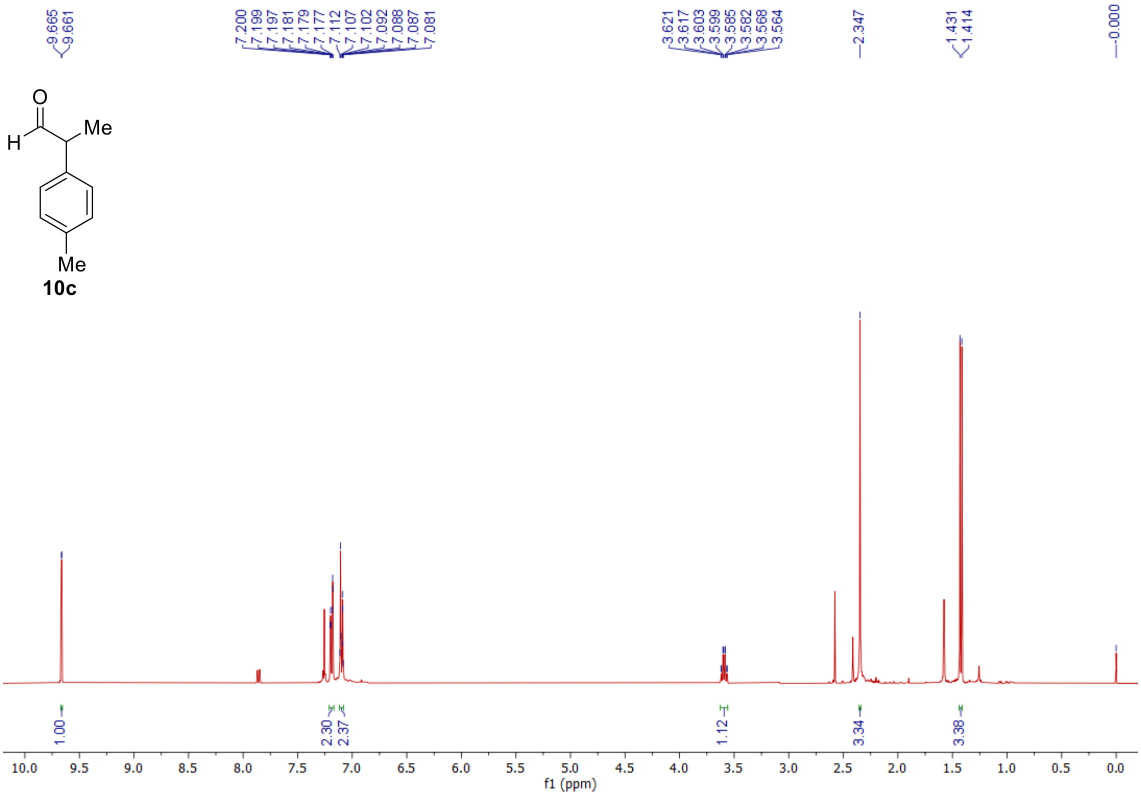

**Figure S5.** $^{1}H$ NMR (300 MHz, $CDCl_3$) of aldehyde **10c**.

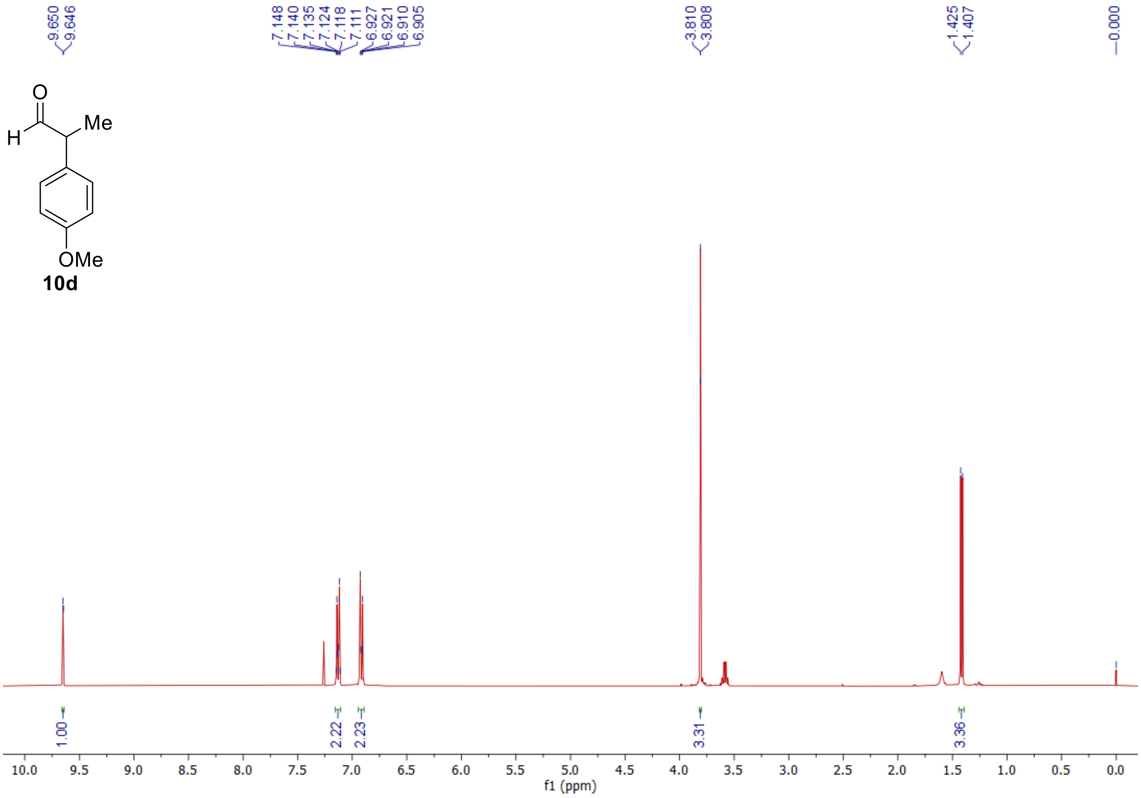

**Figure S6.** $^{1}H$ NMR (300 MHz, $CDCl_3$) of aldehyde **10d**.

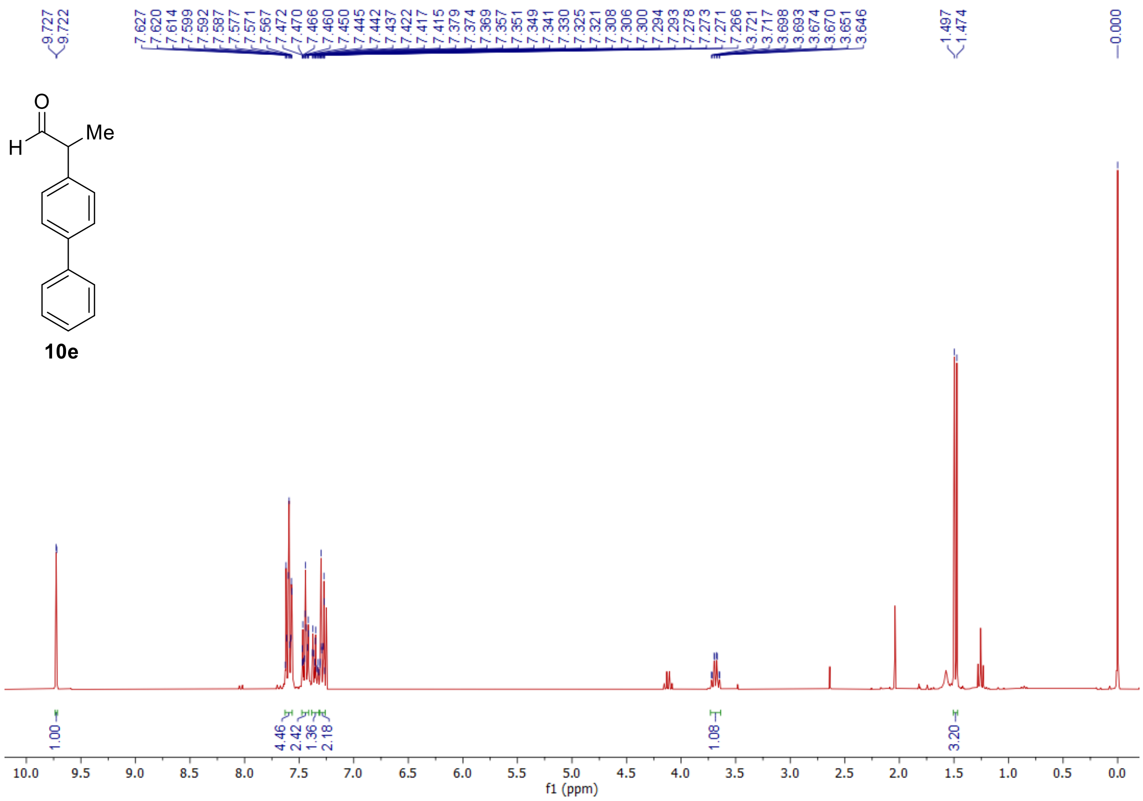

**Figure S7.** $^{1}$H NMR (300 MHz, $CDCl_3$) of aldehyde **10e**.

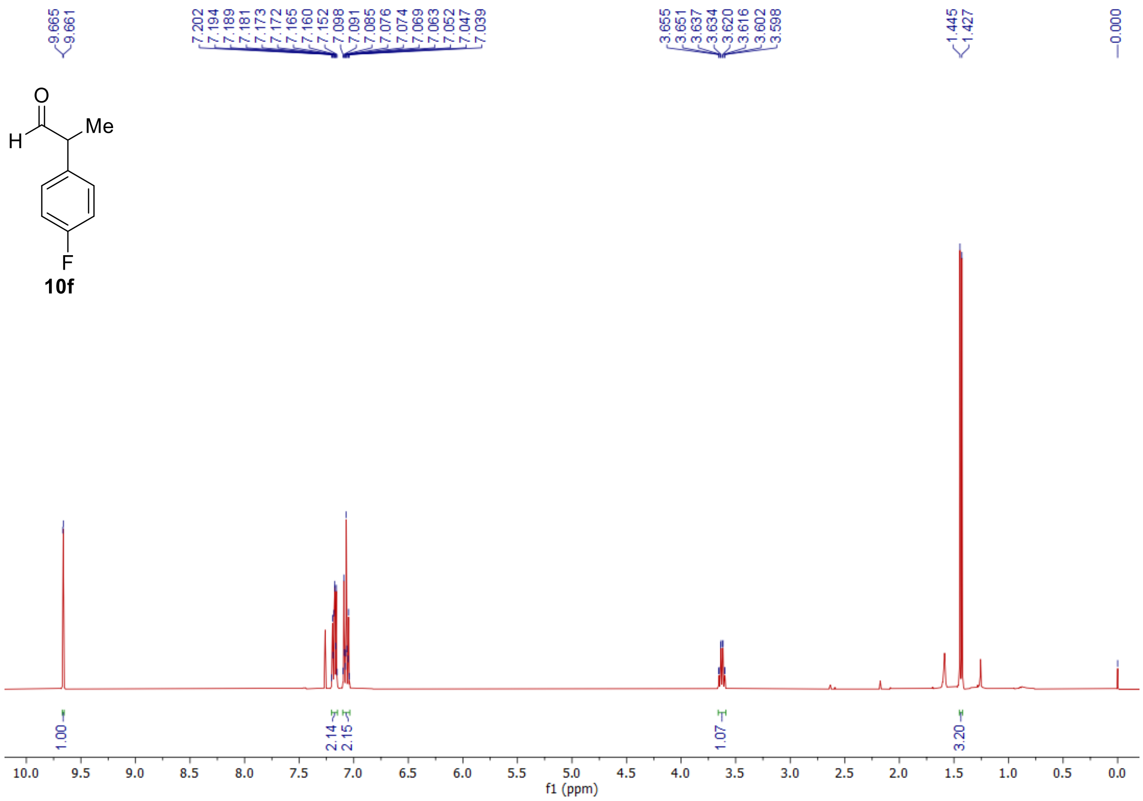

**Figure S8.** $^{1}$H NMR (400 MHz, $CDCl_3$) of aldehyde **10f**.

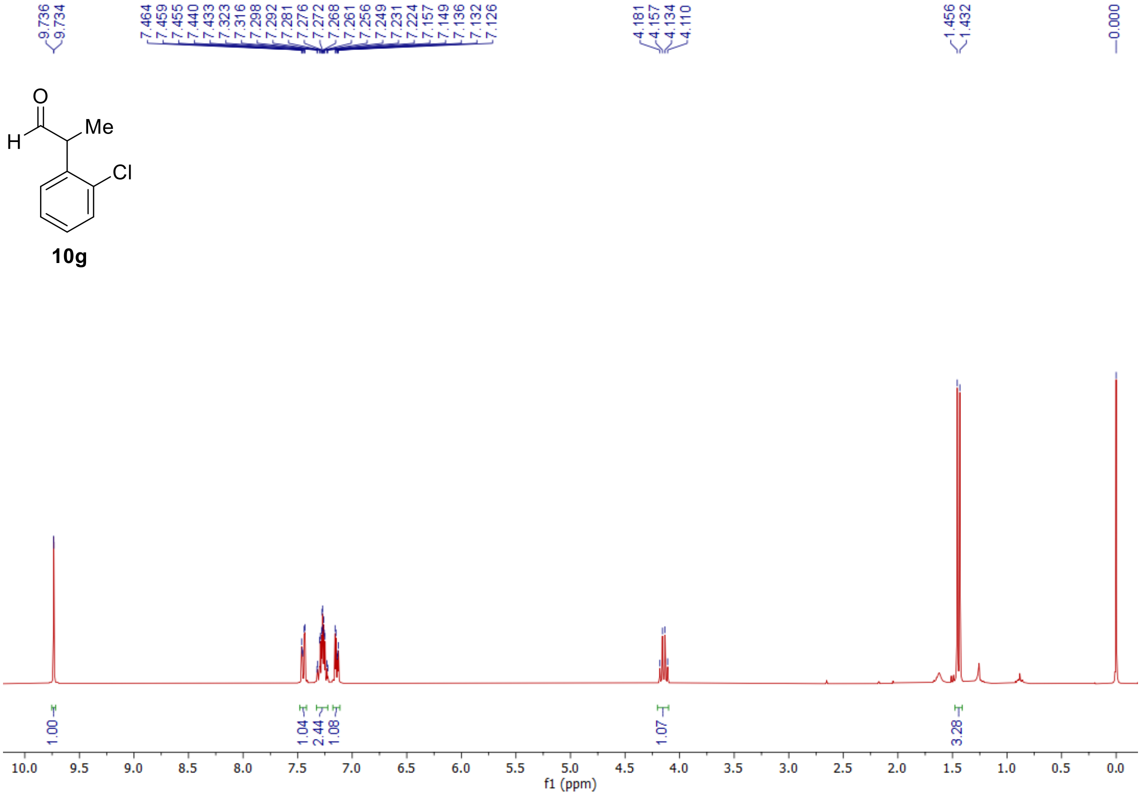


**Figure S9.** $^1$H NMR (400 MHz, $CDCl_3$) of aldehyde **10g**.

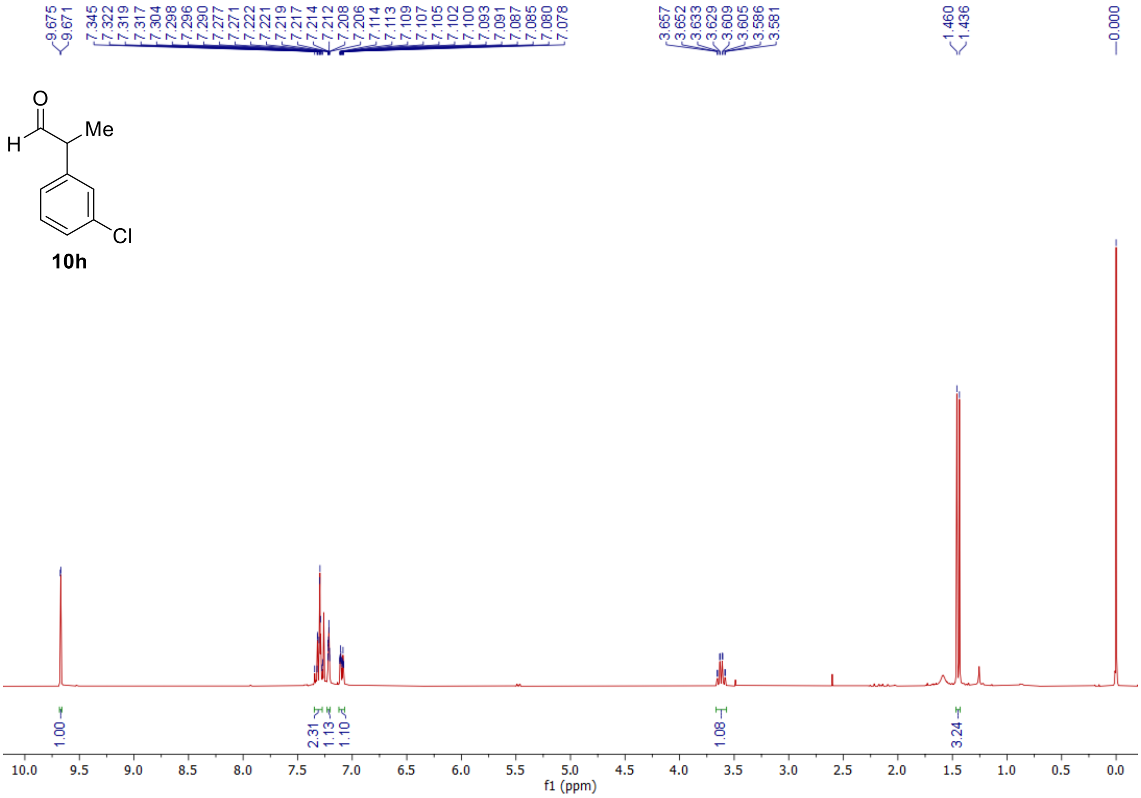


**Figure S10.** $^1$H NMR (300 MHz, $CDCl_3$) of aldehyde **10h**.

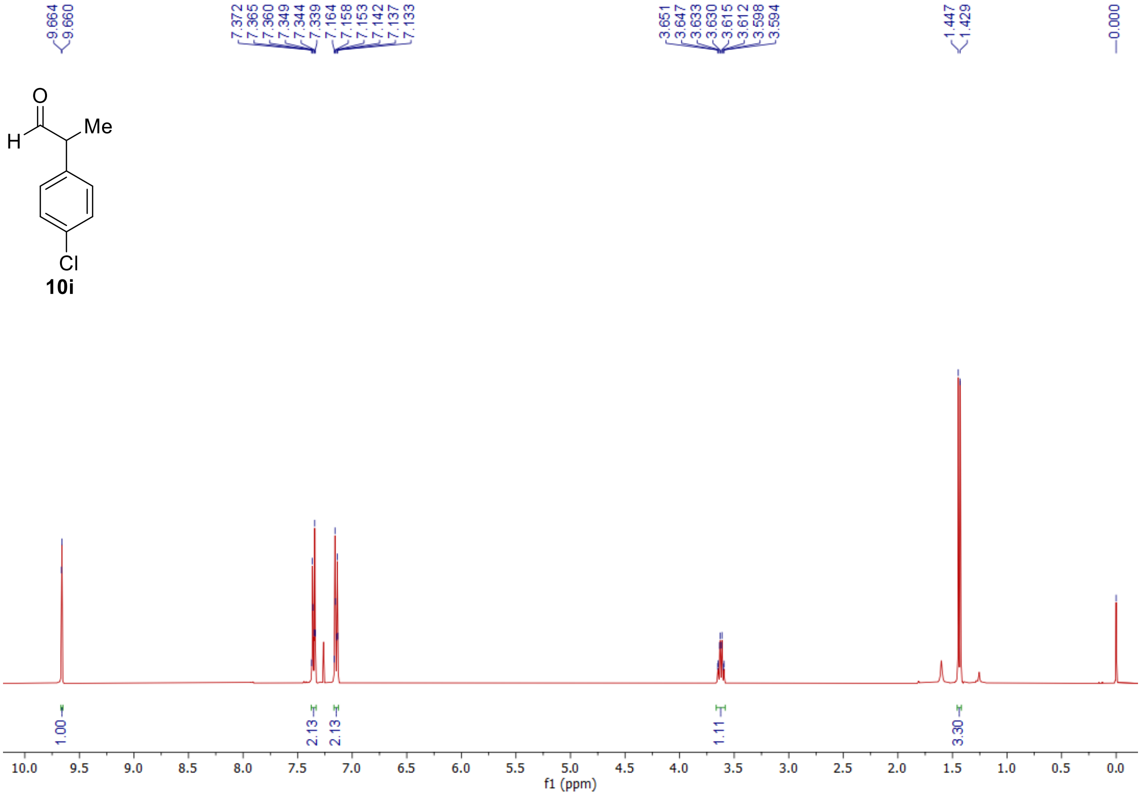

**Figure S11.** $^{1}H$ NMR (300 MHz, $CDCl_3$) of aldehyde **10i**.

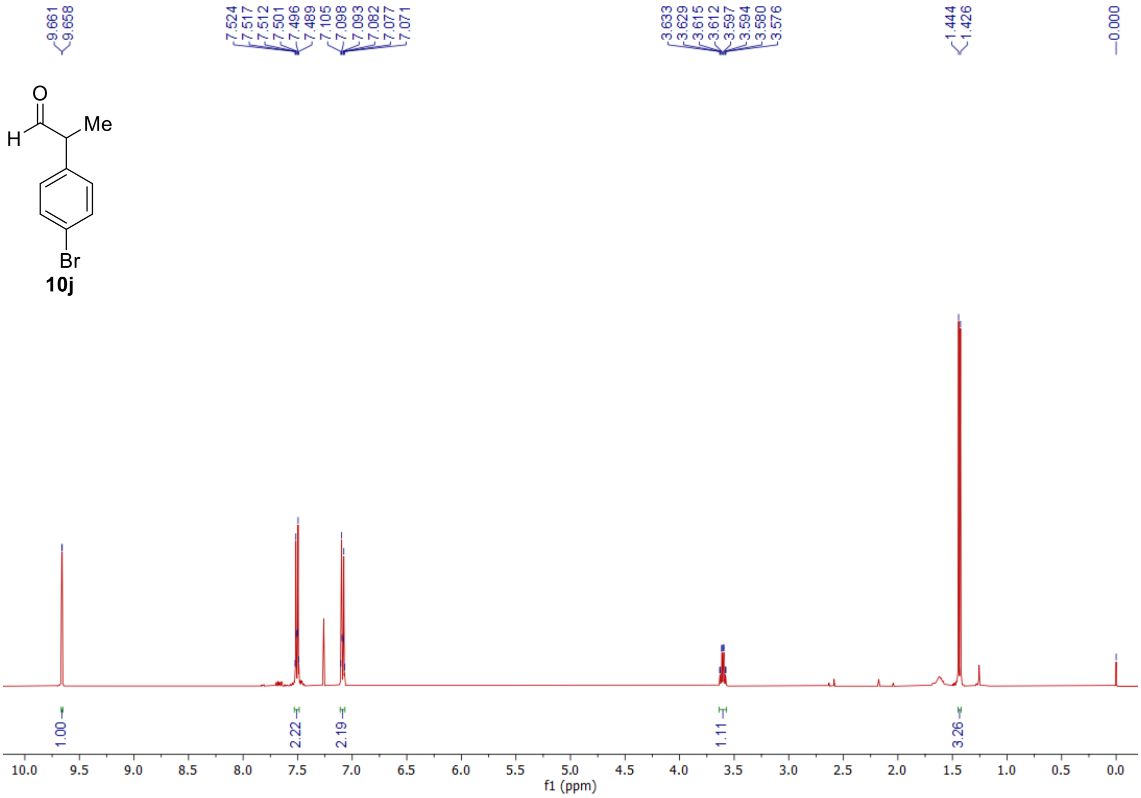

**Figure S12.** $^{1}H$ NMR (400 MHz, $CDCl_3$) of aldehyde **10j**.

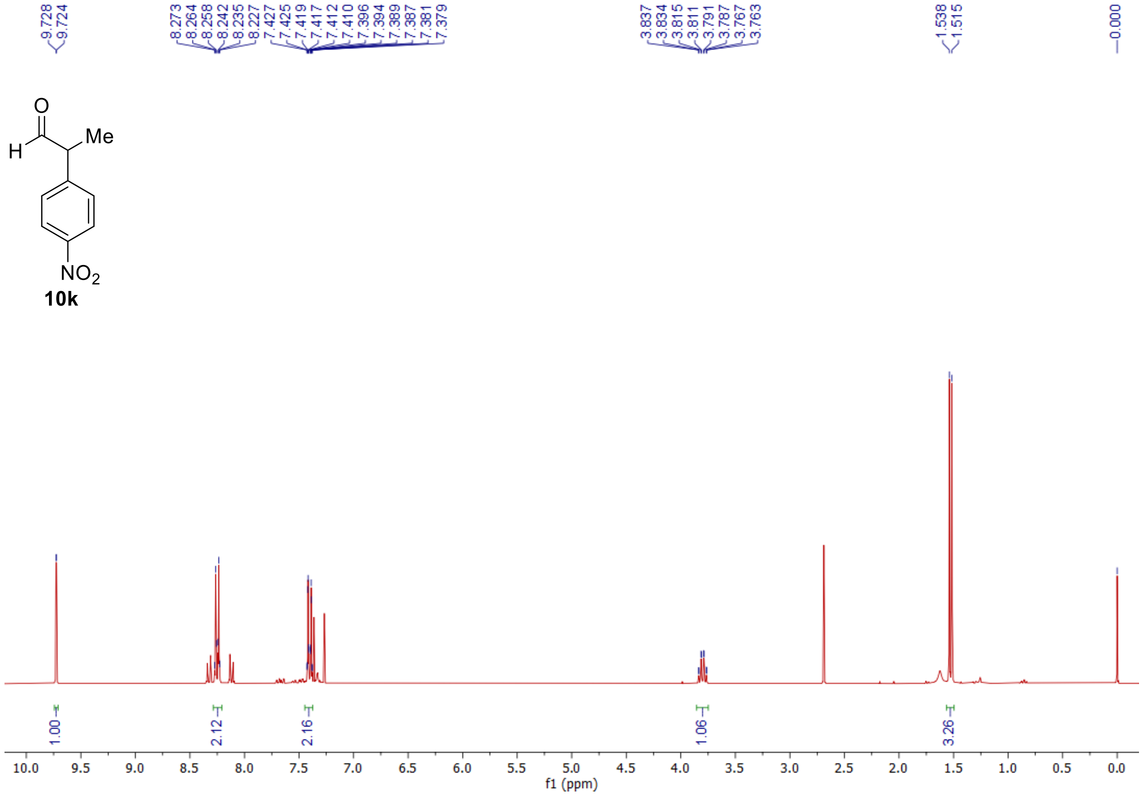

**Figure S13.** $^1$H NMR (300 MHz, $CDCl_3$) of aldehyde **10k**.

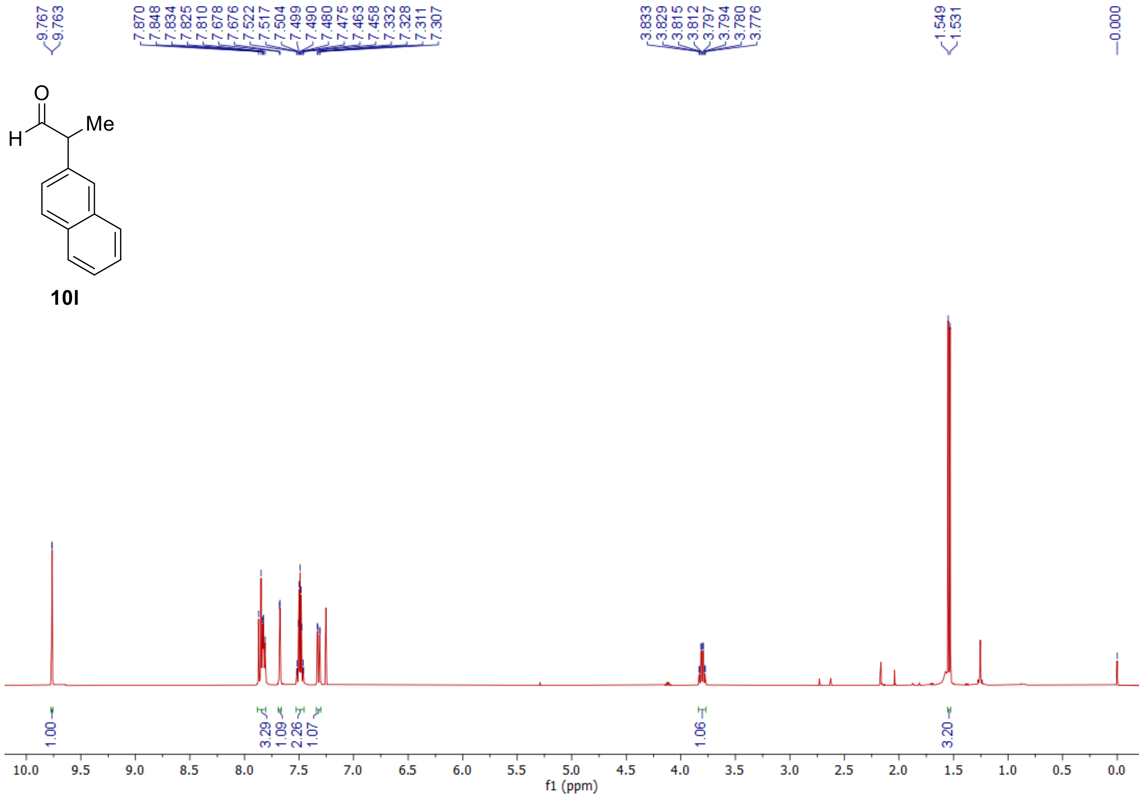

**Figure S14.** $^1$H NMR (400 MHz, $CDCl_3$) of aldehyde **10l**.

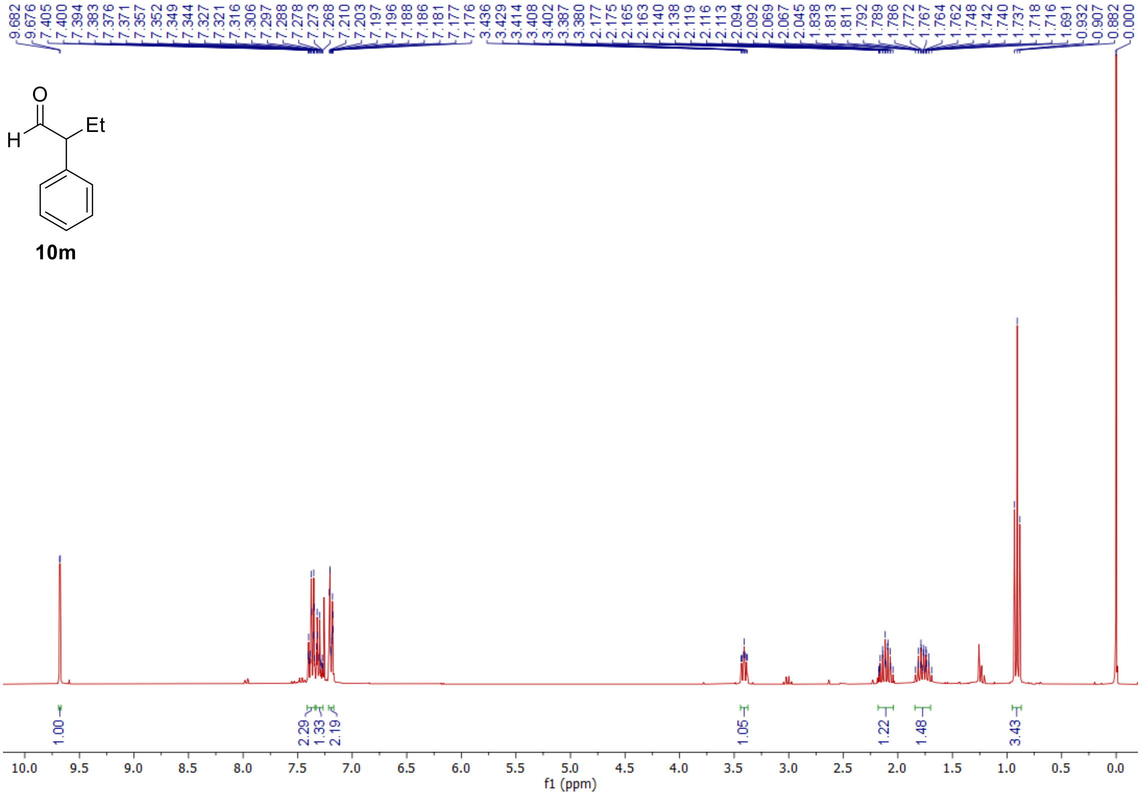


**Figure S15.** $^{1}$H NMR (300 MHz, $CDCl_3$) of aldehyde **10m**.

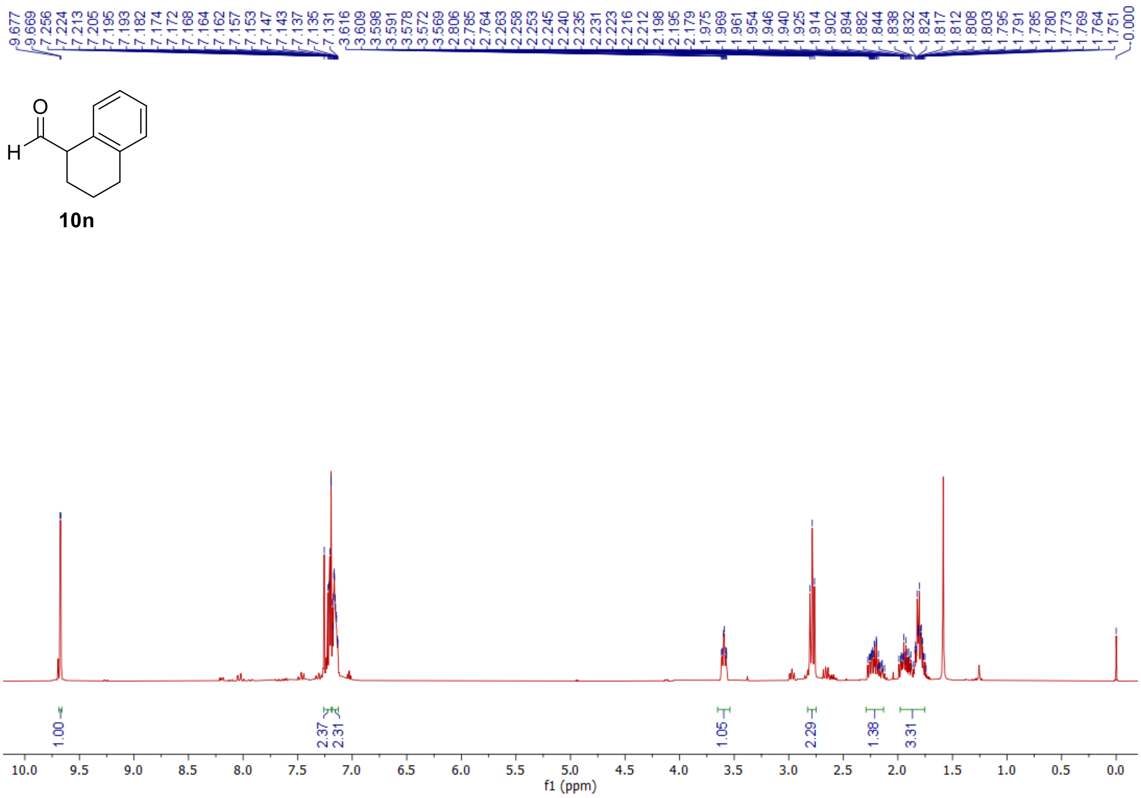


**Figure S16.** $^{1}$H NMR (300 MHz, $CDCl_3$) of aldehyde **10n**.

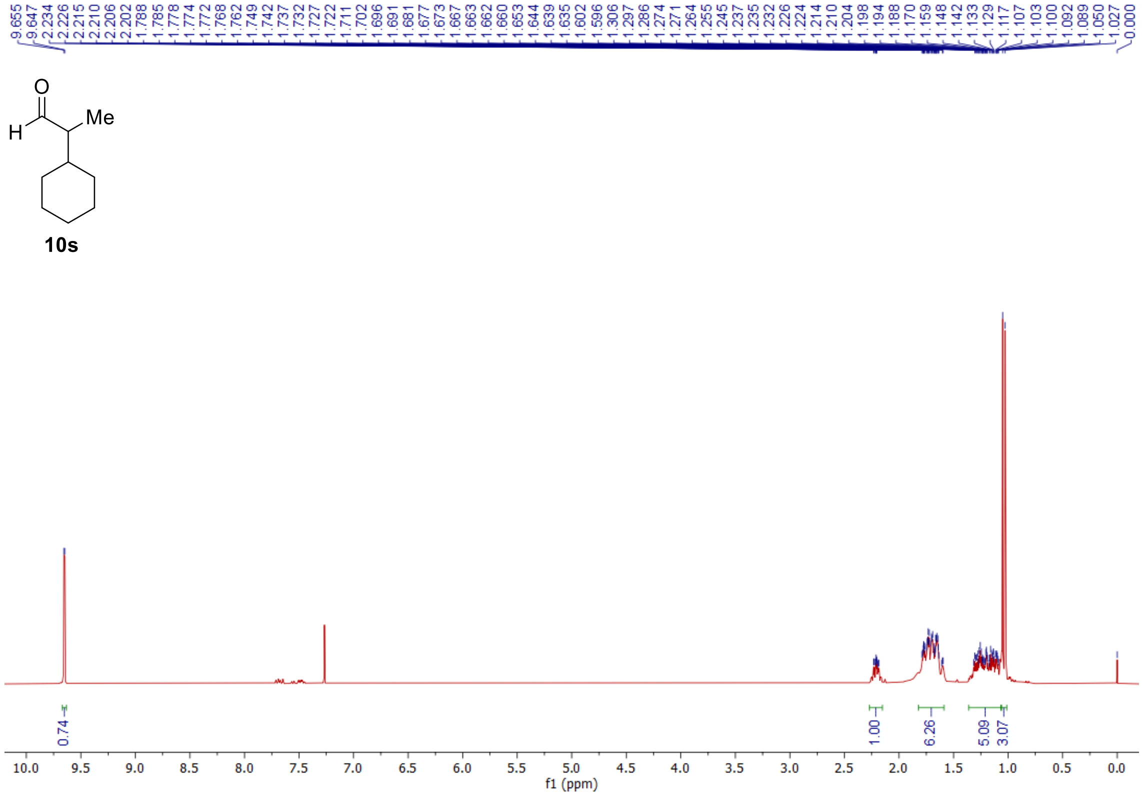

**Figure S17.** $^1$H NMR (300 MHz, $CDCl_3$) of aldehyde **10s**.

VI. $^{1}H$ NMR and $^{13}C\{^{1}H\}$ NMR spectra of compounds **12**, **13** and **14**

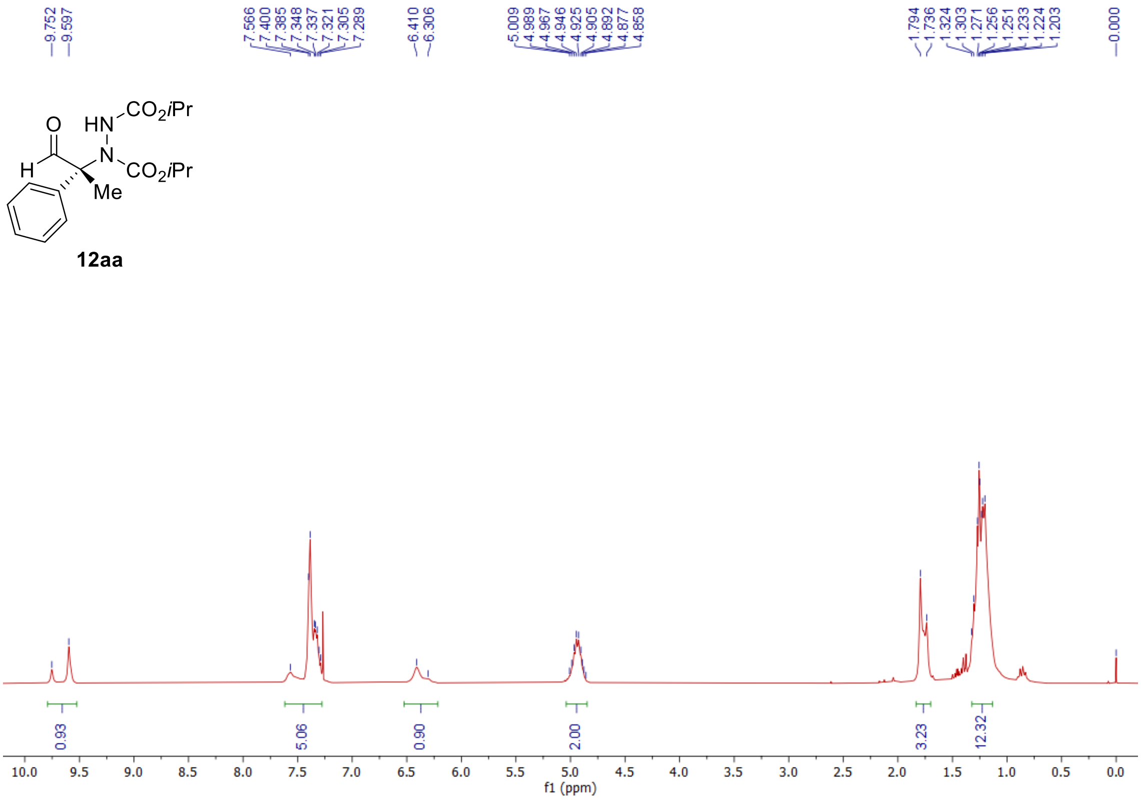

Figure S18. $^{1}H$ NMR (300 MHz, $CDCl_3$) of compound **12aa**.

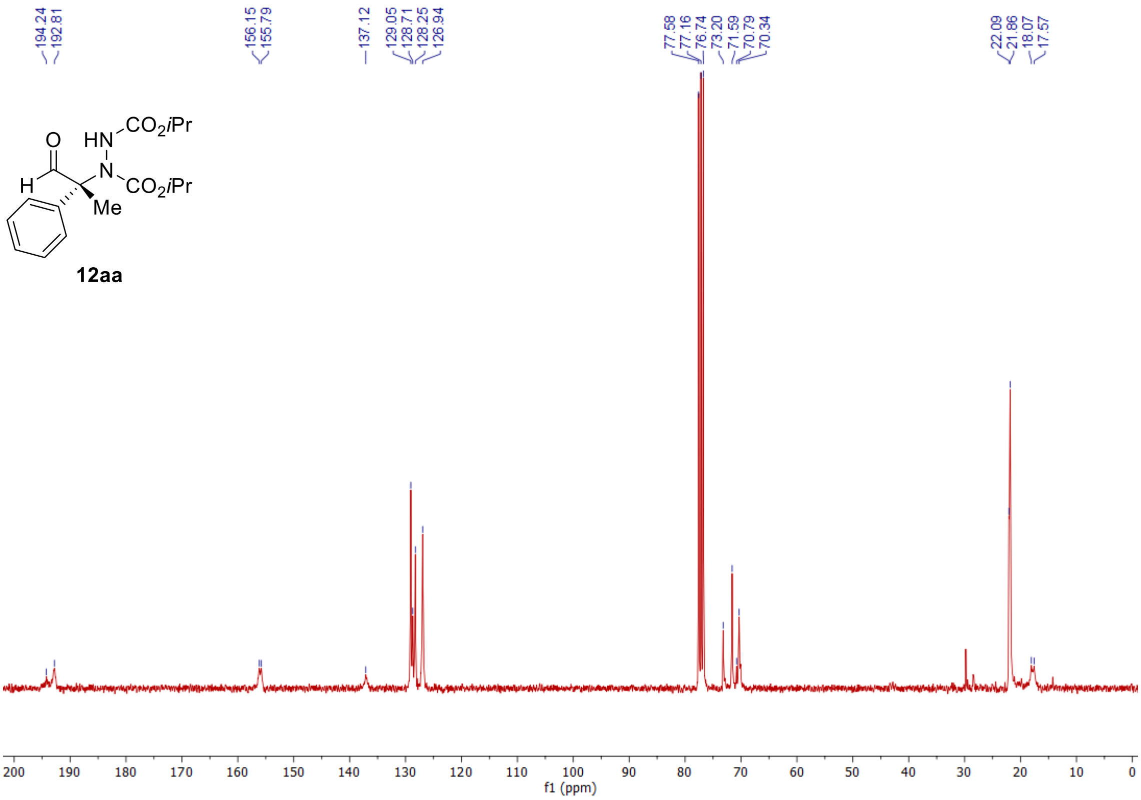

Figure S19. $^{13}C\{^{1}H\}$ NMR (75 MHz, $CDCl_3$) of compound **12aa**.

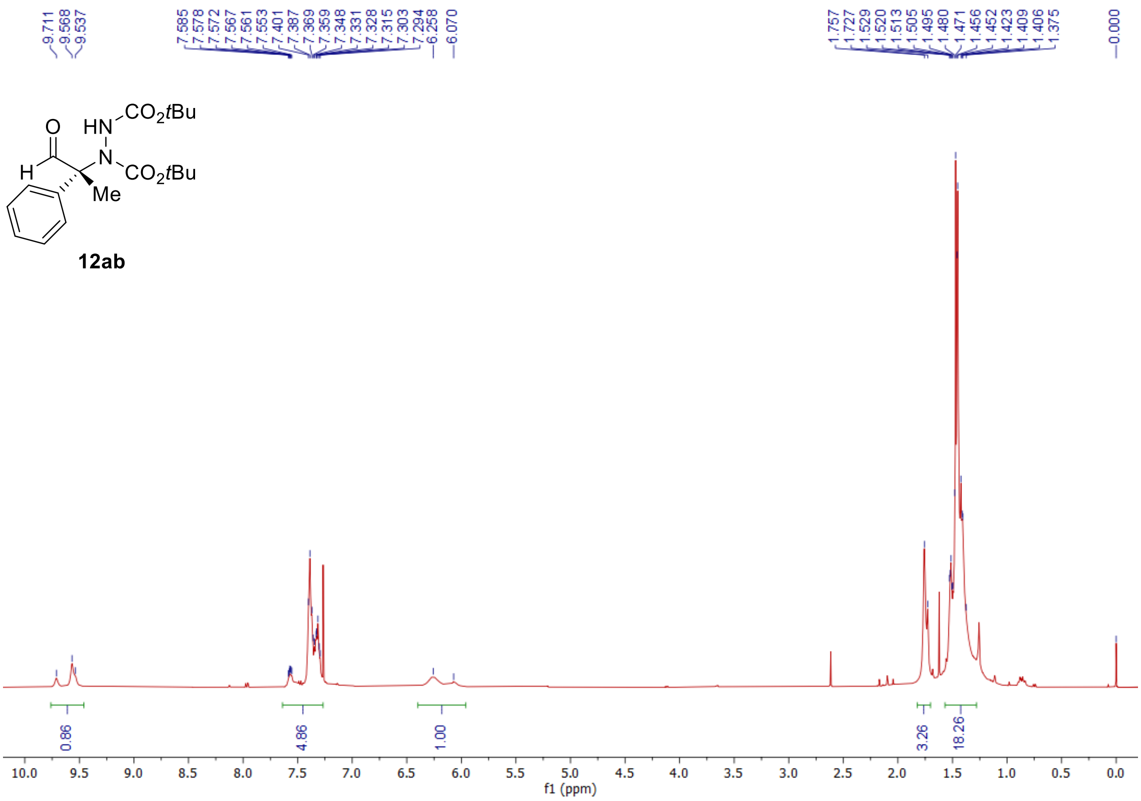


**Figure S20.** $^{1}$H NMR (400 MHz, $CDCl_3$) of compound **12ab**.

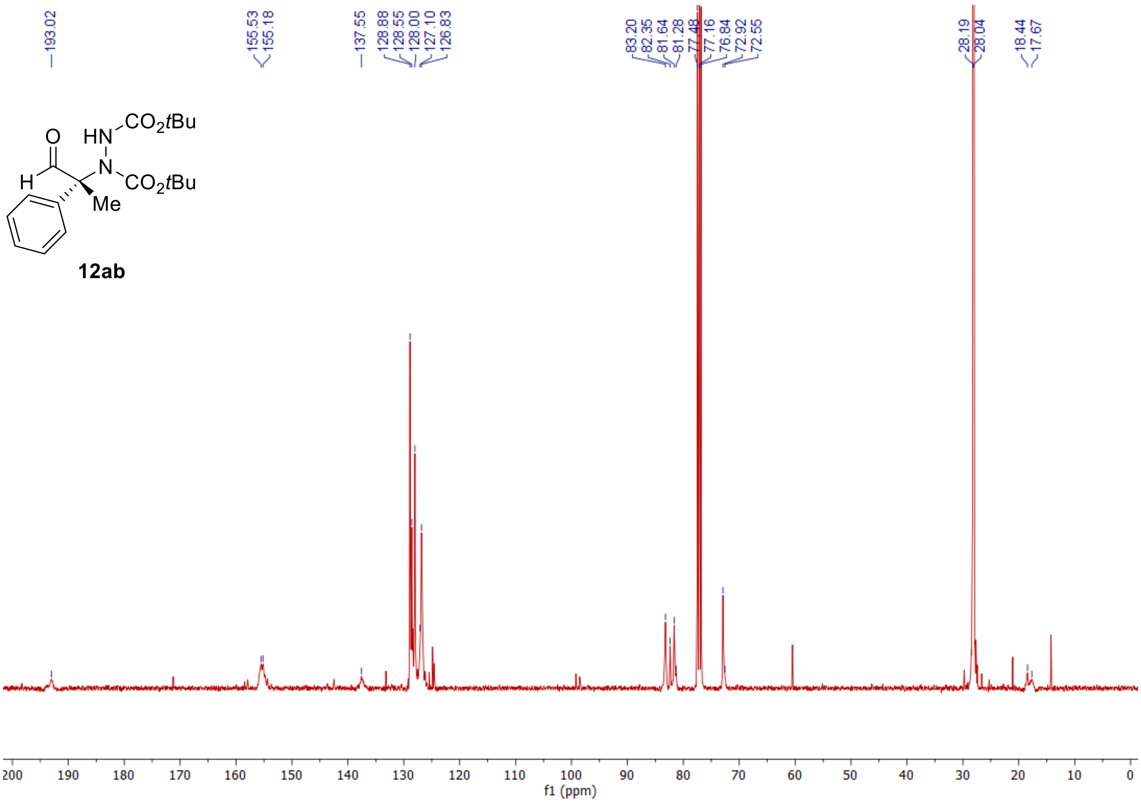


**Figure S21.** $^{13}C\{^{1}H\}$ NMR (101 MHz, $CDCl_3$) of compound **12ab**.

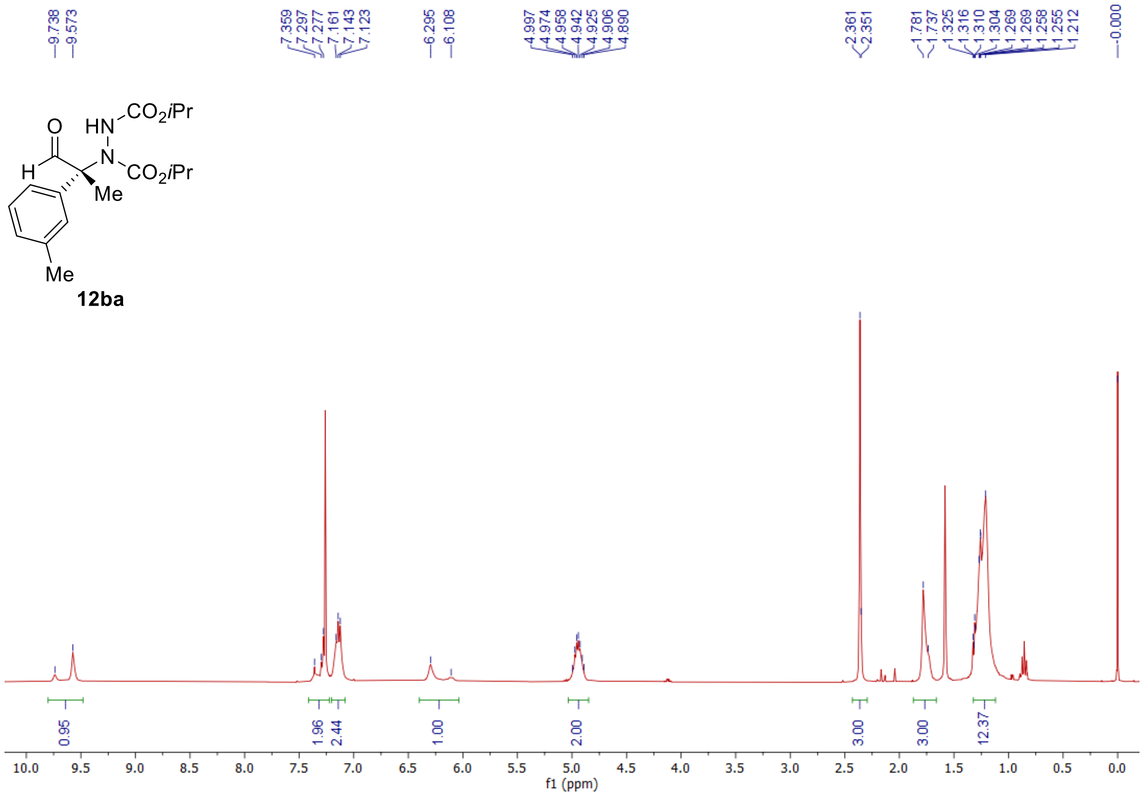

**Figure S22.** $^{1}H$ NMR (400 MHz, $CDCl_3$) of compound **12ba**.

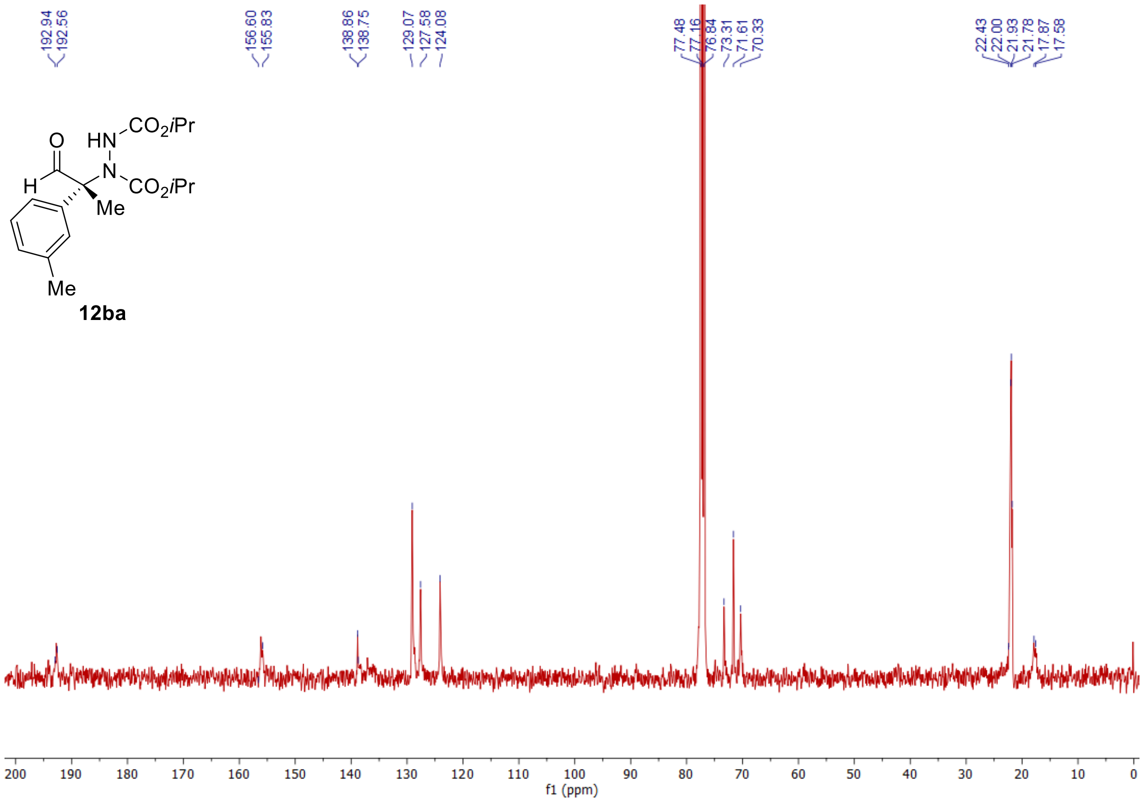

**Figure S23.** $^{13}C\{^{1}H\}$ NMR (101 MHz, $CDCl_3$) of compound **12ba**.

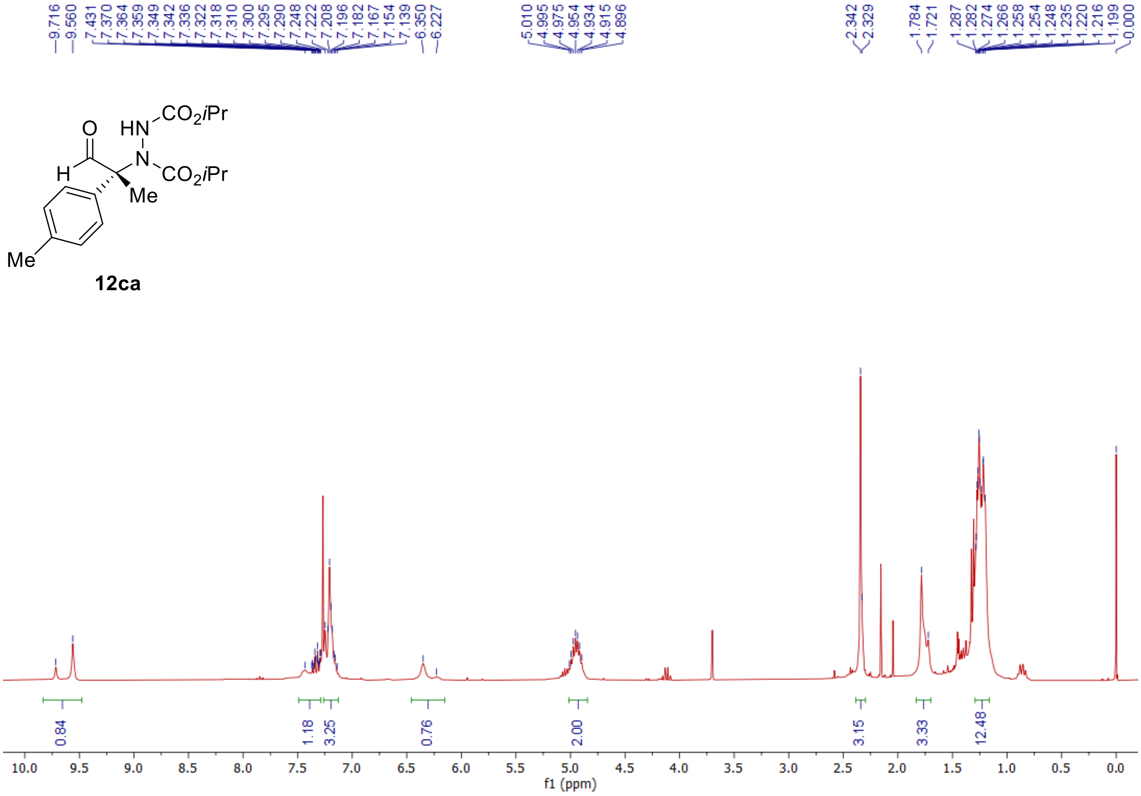


**Figure S24.** $^{1}$H NMR (300 MHz, $CDCl_3$) of compound **12ca**.

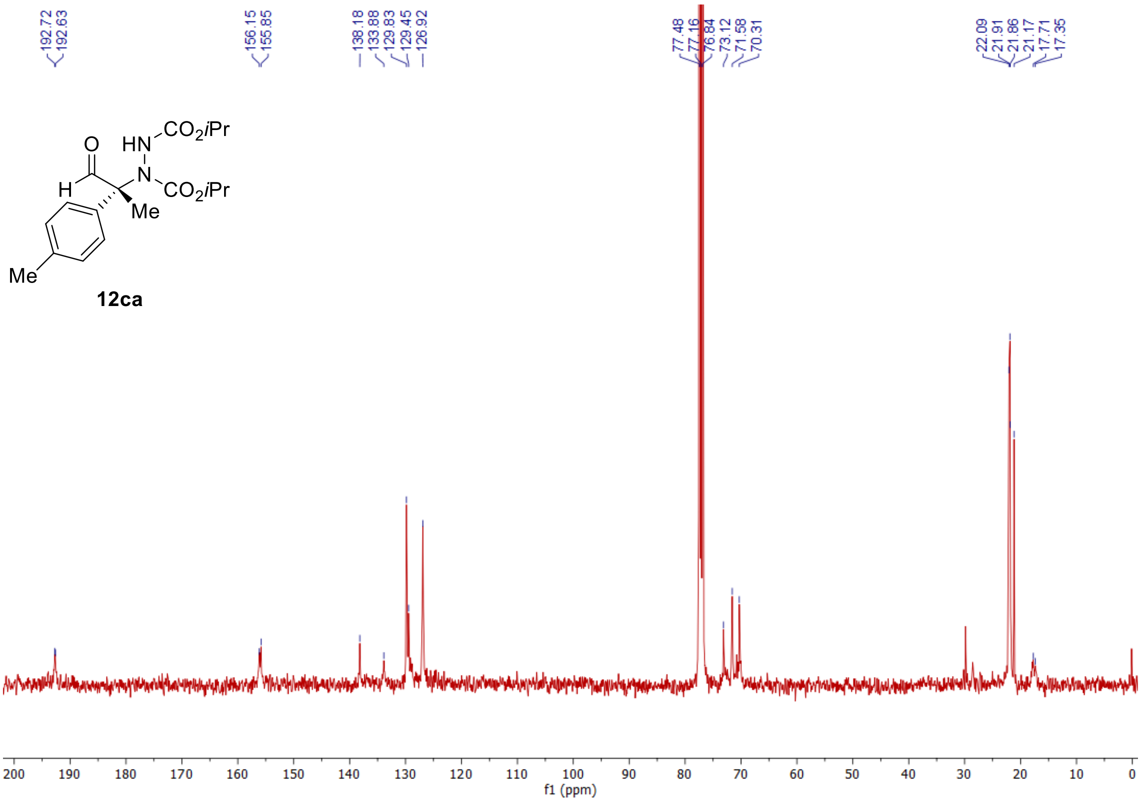


**Figure S25.** $^{13}C\{^{1}H\}$ NMR (75 MHz, $CDCl_3$) of compound **12ca**.

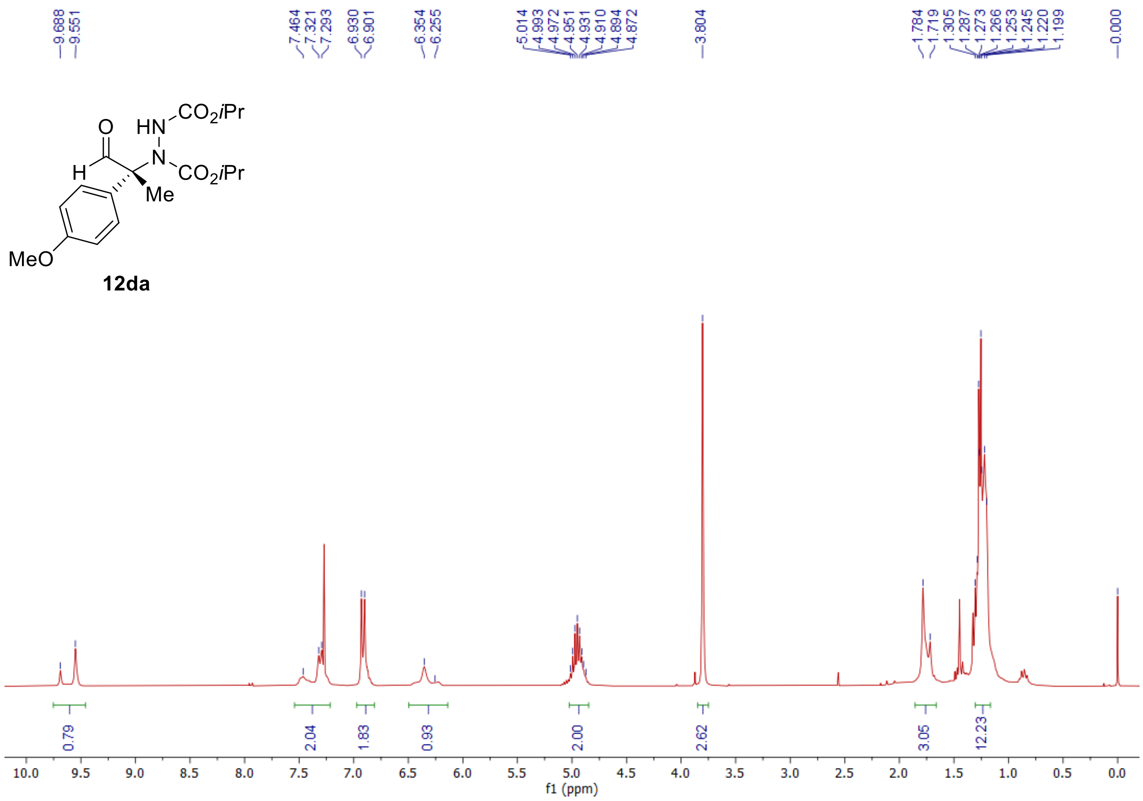

Figure S26. $^{1}$H NMR (300 MHz, $CDCl_3$) of compound **12da**.

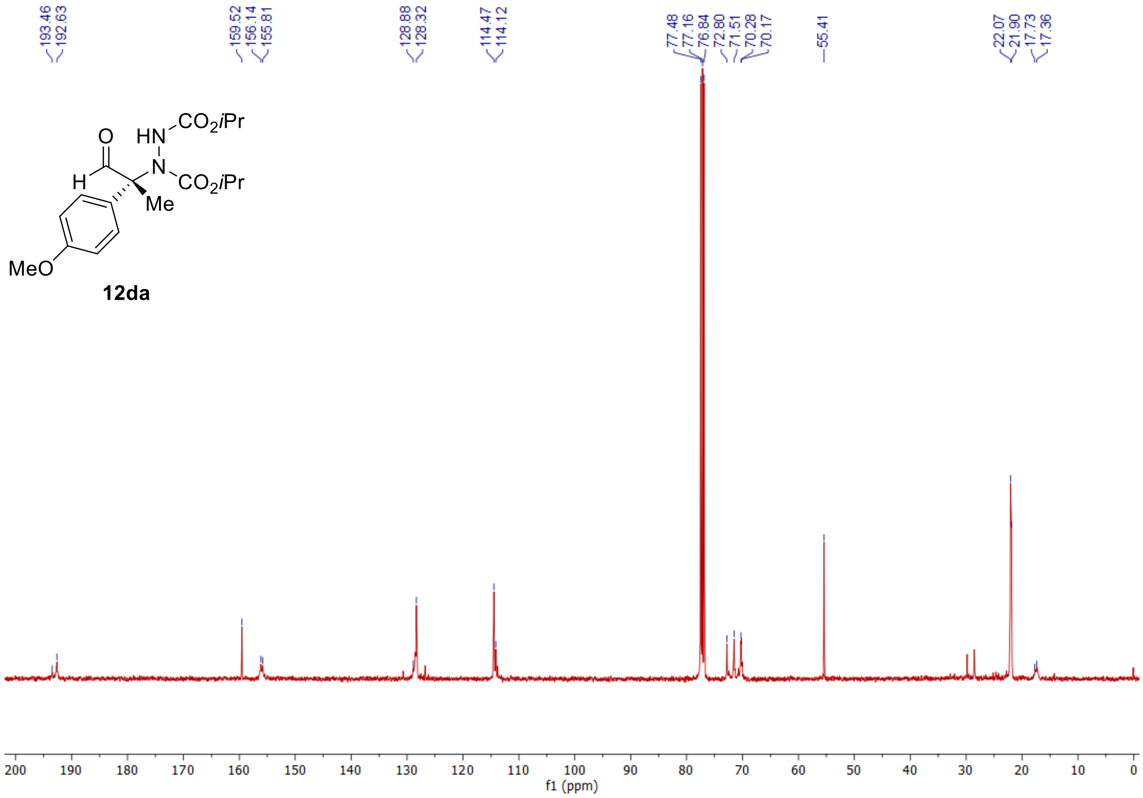

Figure S27. $^{13}C\{^1H\}$ NMR (75 MHz, $CDCl_3$) of compound **12da**.

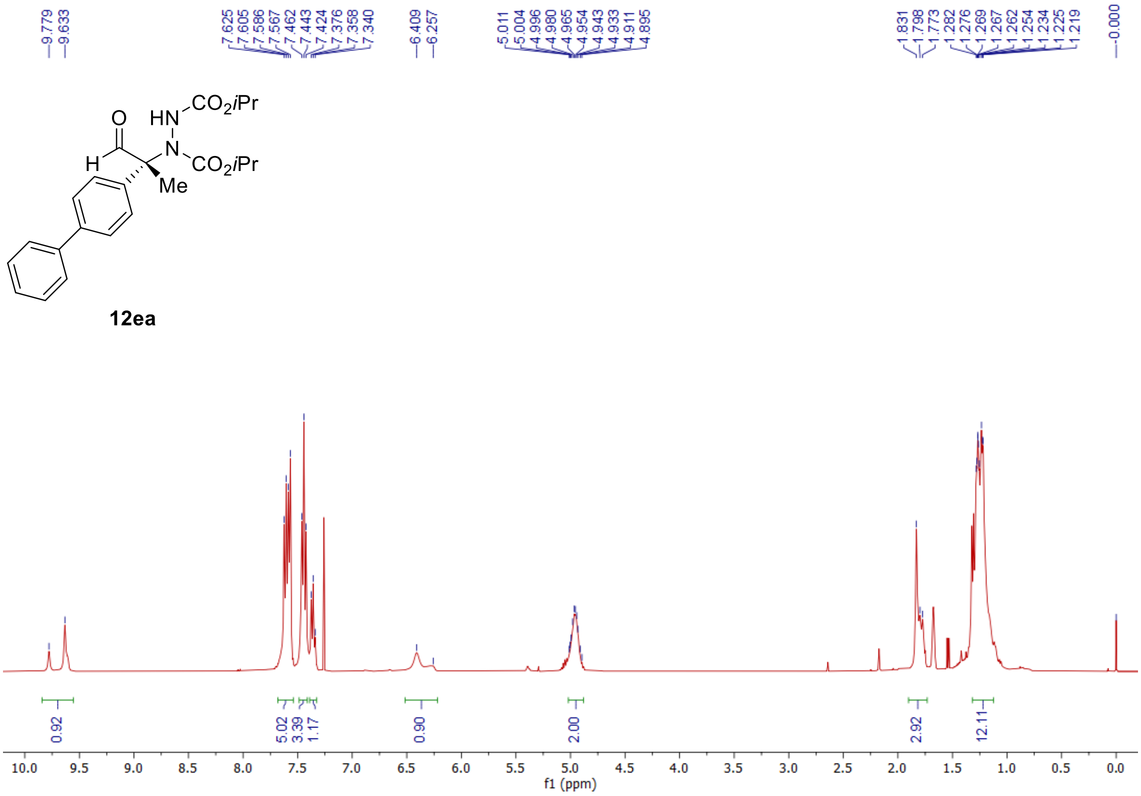


**Figure S28.** $^1$H NMR (300 MHz, CDCl$_3$) of compound **12ea**.

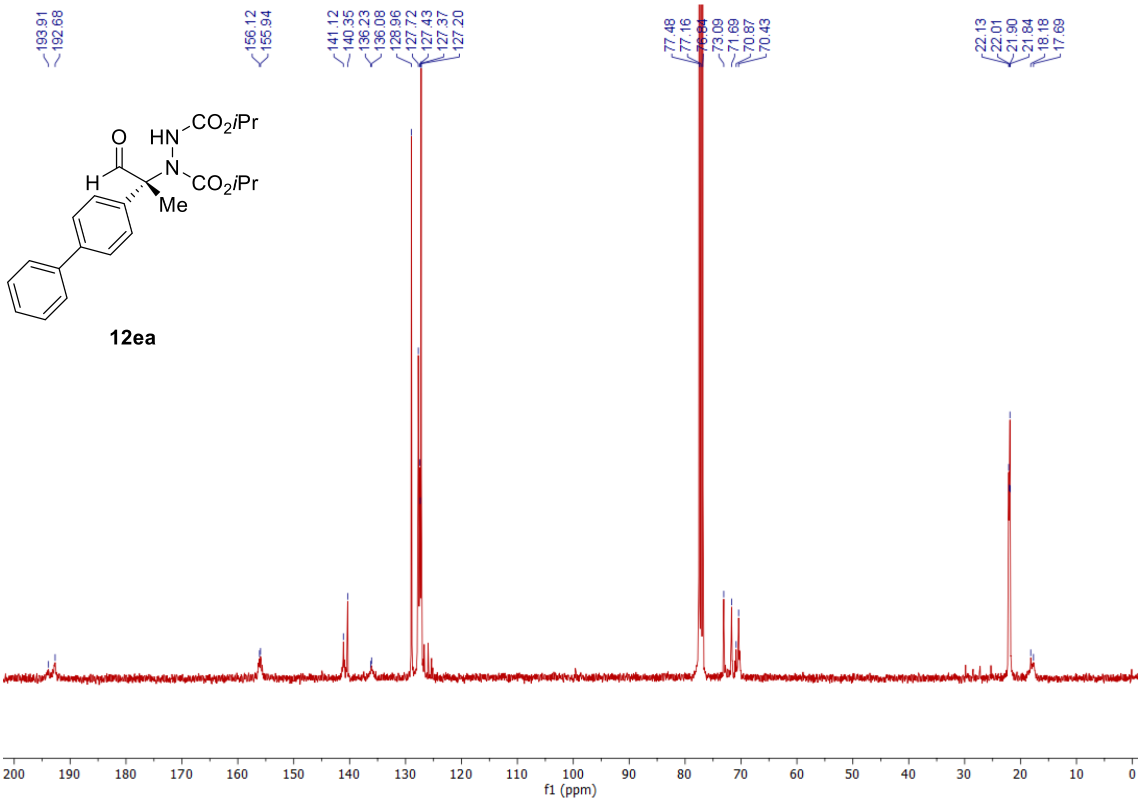


**Figure S29.** $^{13}$C{$^1$H} NMR (75 MHz, CDCl$_3$) of compound **12ea**.

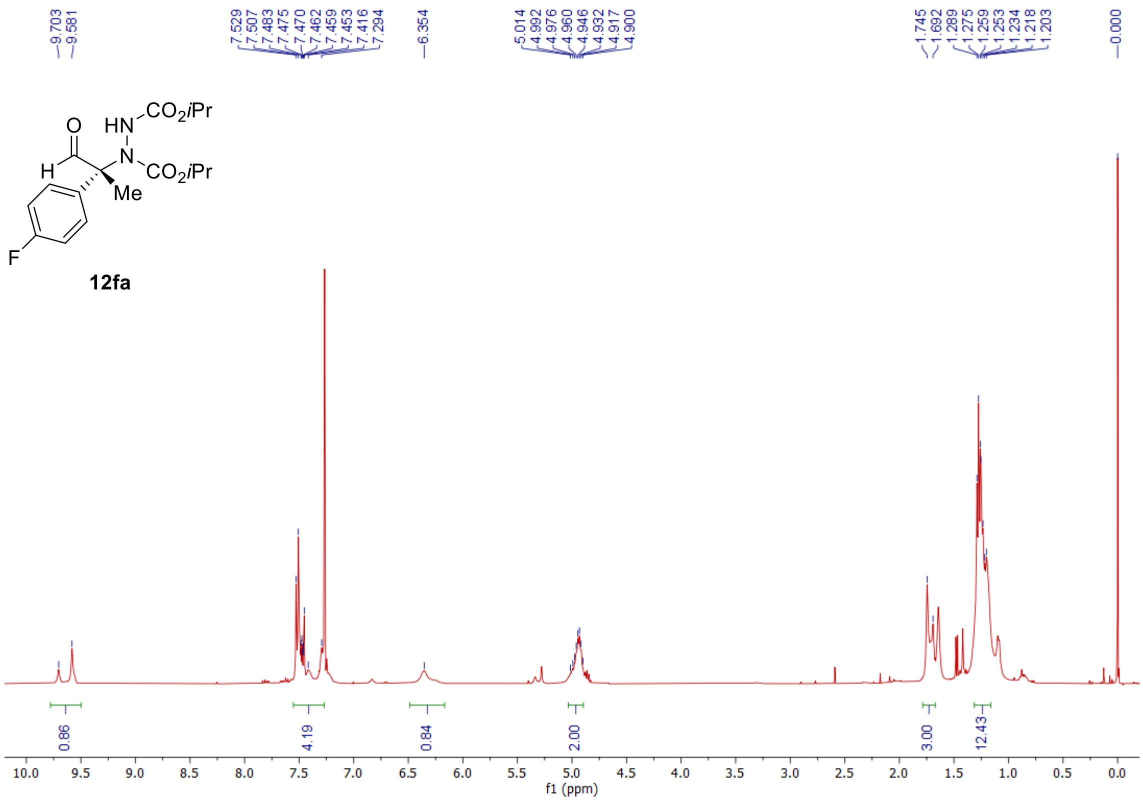

**Figure S30.** $^{1}H$ NMR (400 MHz, $CDCl_3$) of compound **12fa**.

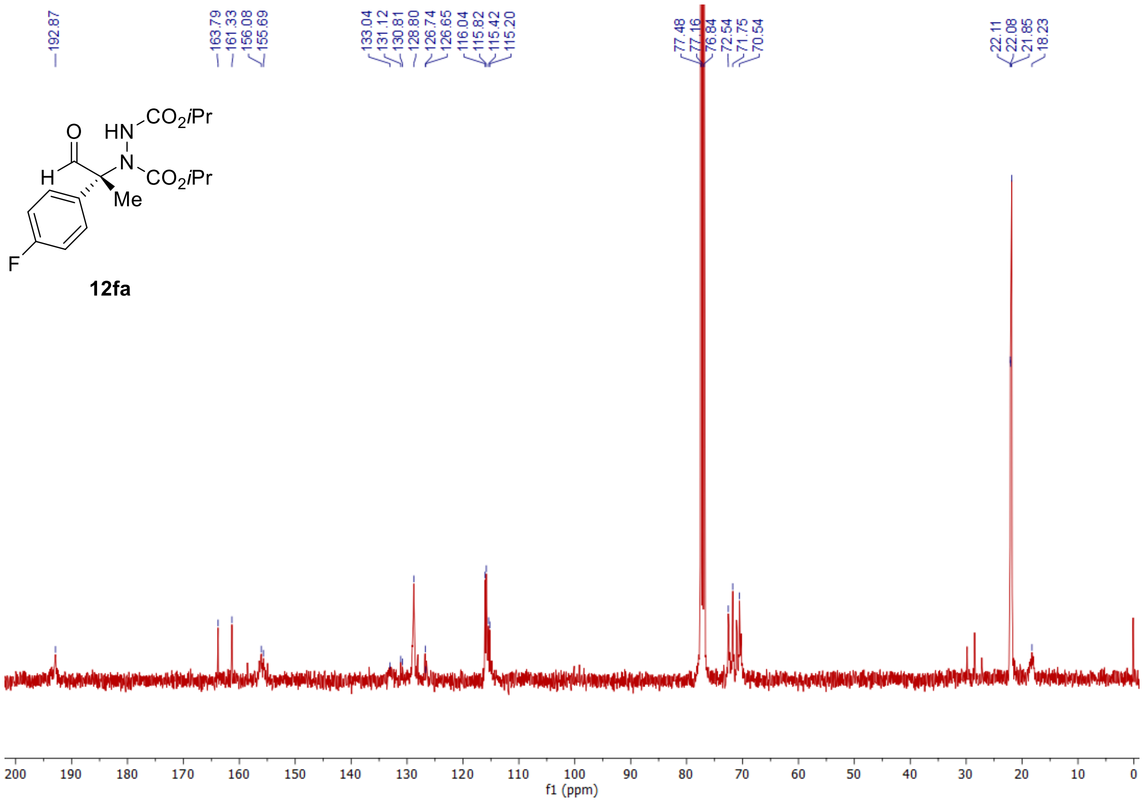

**Figure S31.** $^{13}C\{^{1}H\}$ NMR (101 MHz, $CDCl_3$) of compound **12fa**.

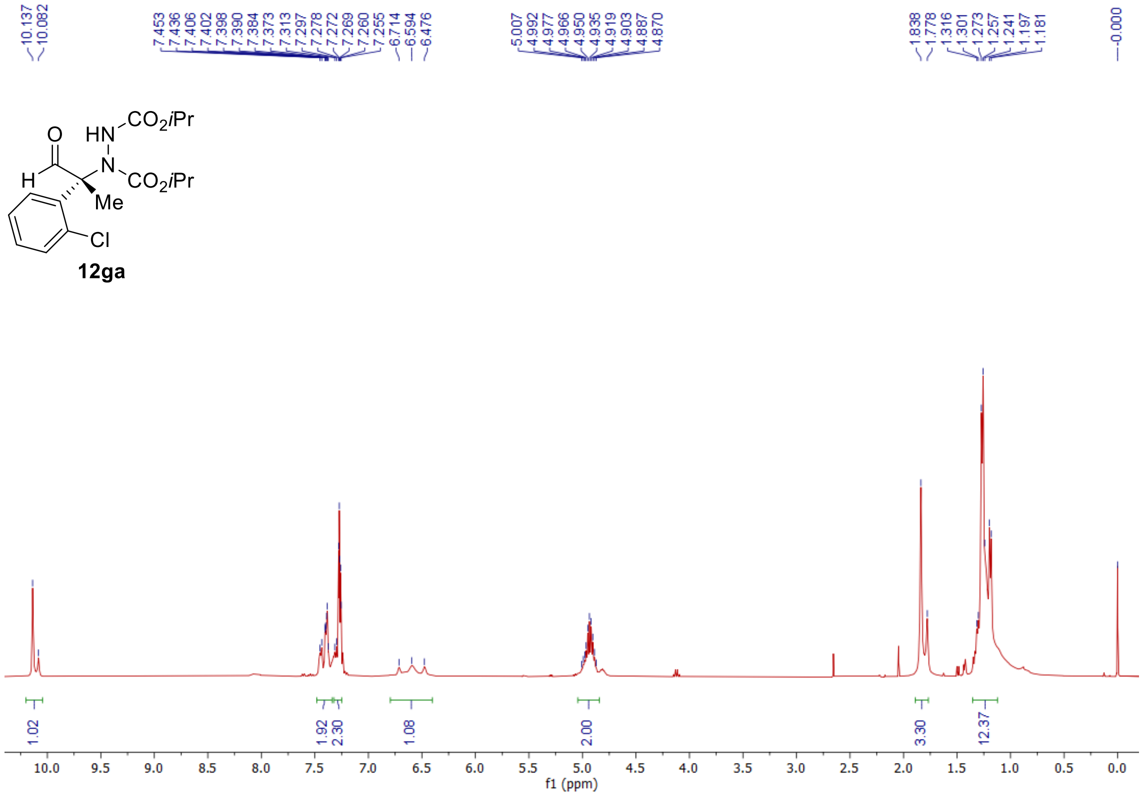


**Figure S32.** $^{1}$H NMR (400 MHz, CDCl$_3$) of compound **12ga**.

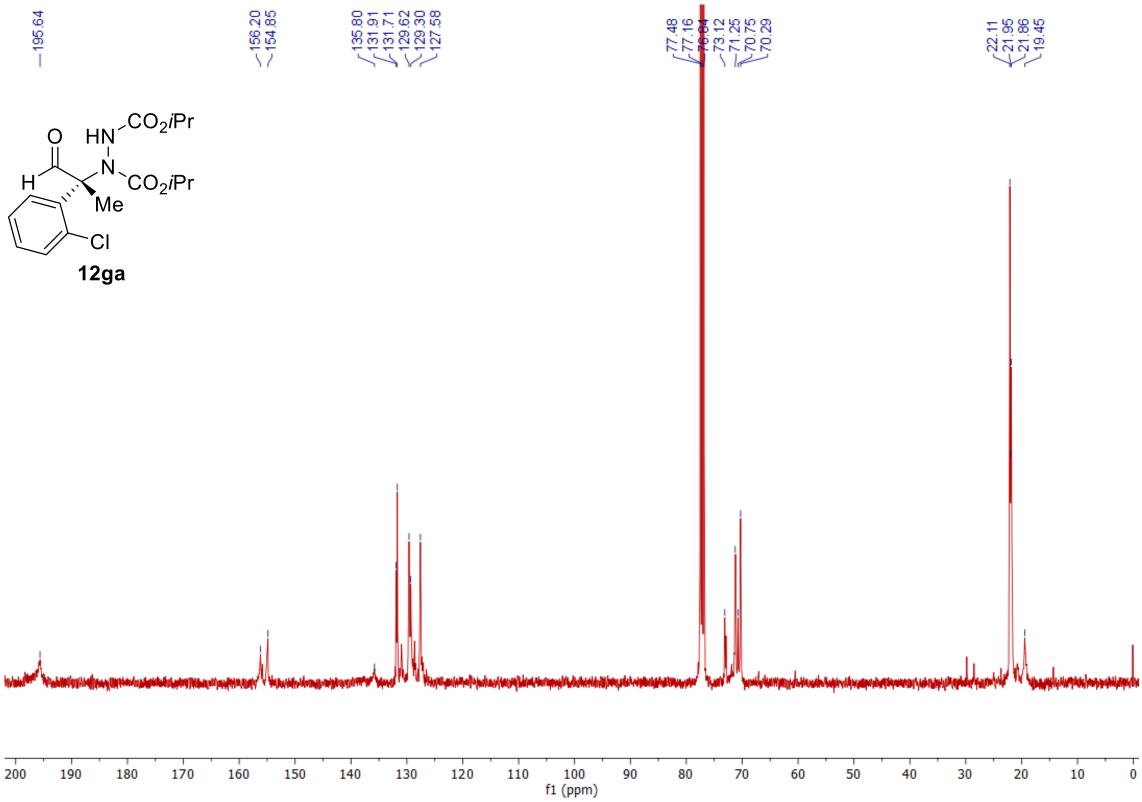


**Figure S33.** $^{13}$C{$^{1}$H} NMR (101 MHz, CDCl$_3$) of compound **12ga**.

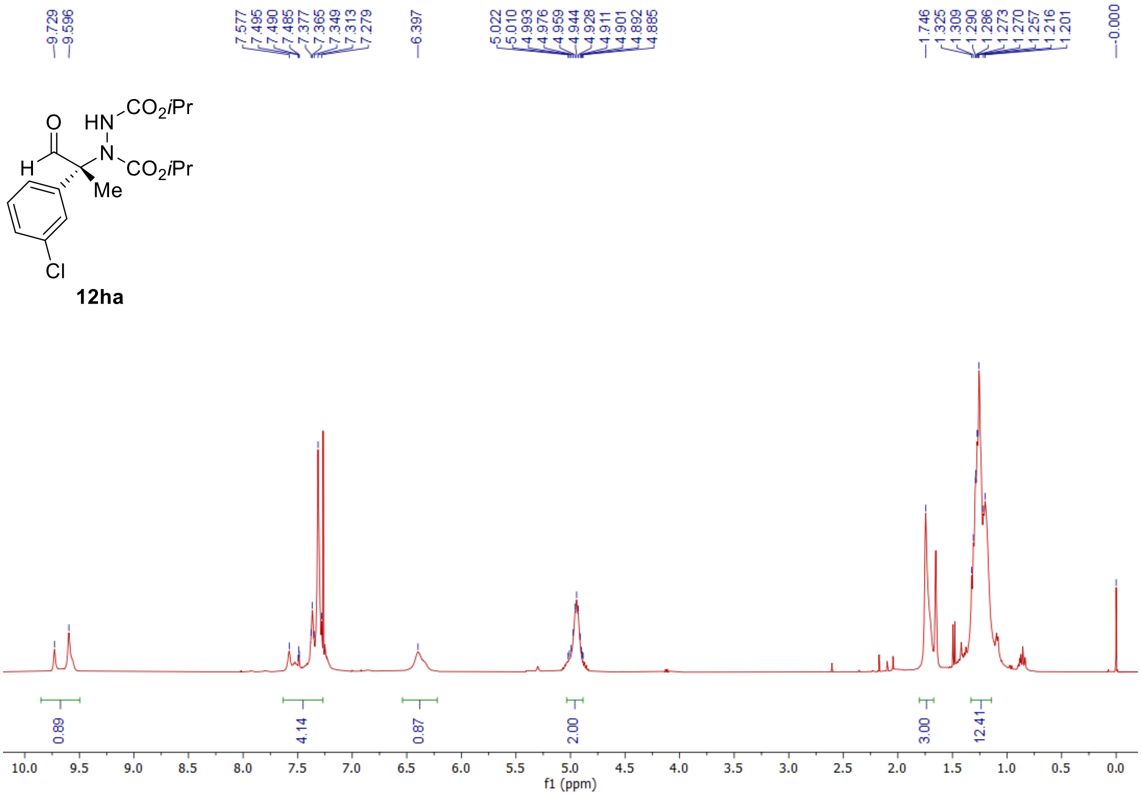


**Figure S34.** $^{1}H$ NMR (400 MHz, $CDCl_3$) of compound **12ha**.

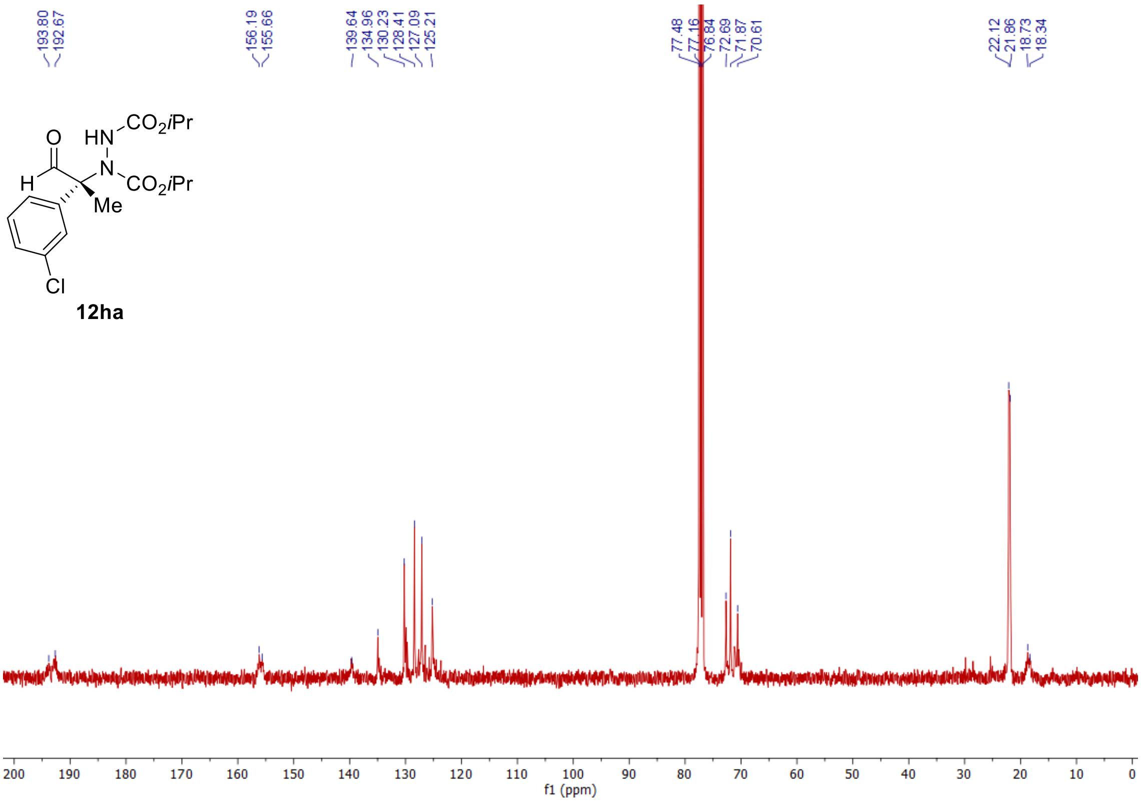


**Figure S35.** $^{13}C\{^{1}H\}$ NMR (101 MHz, $CDCl_3$) of compound **12ha**.

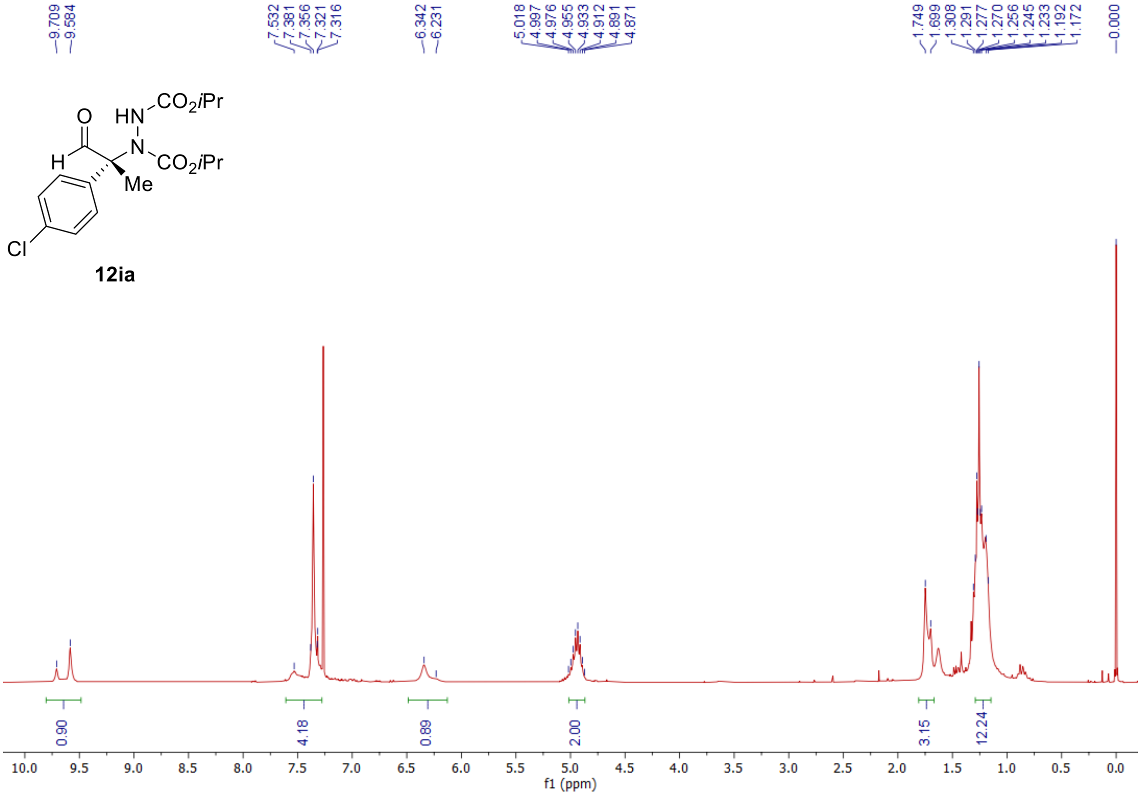


**Figure S36.** $^{1}$H NMR (300 MHz, CDCl$_3$) of compound **12ia**.

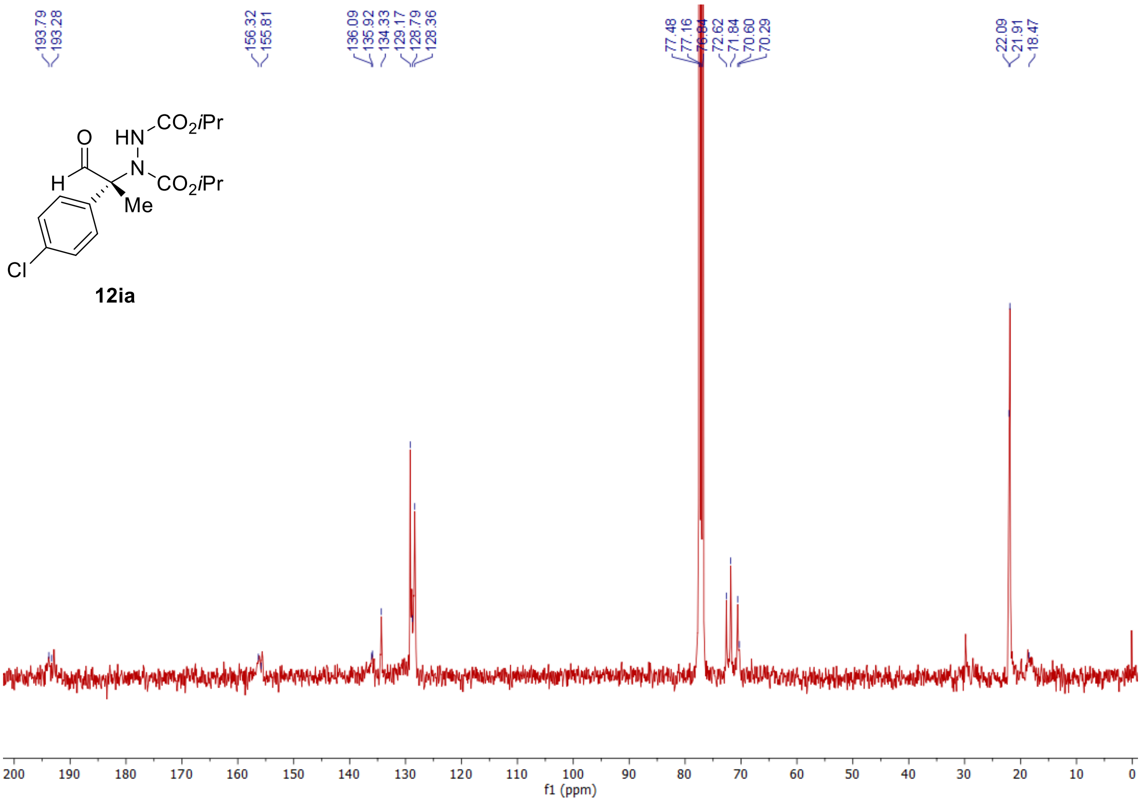


**Figure S37.** $^{13}$C{$^{1}$H} NMR (75 MHz, CDCl$_3$) of compound **12ia**.

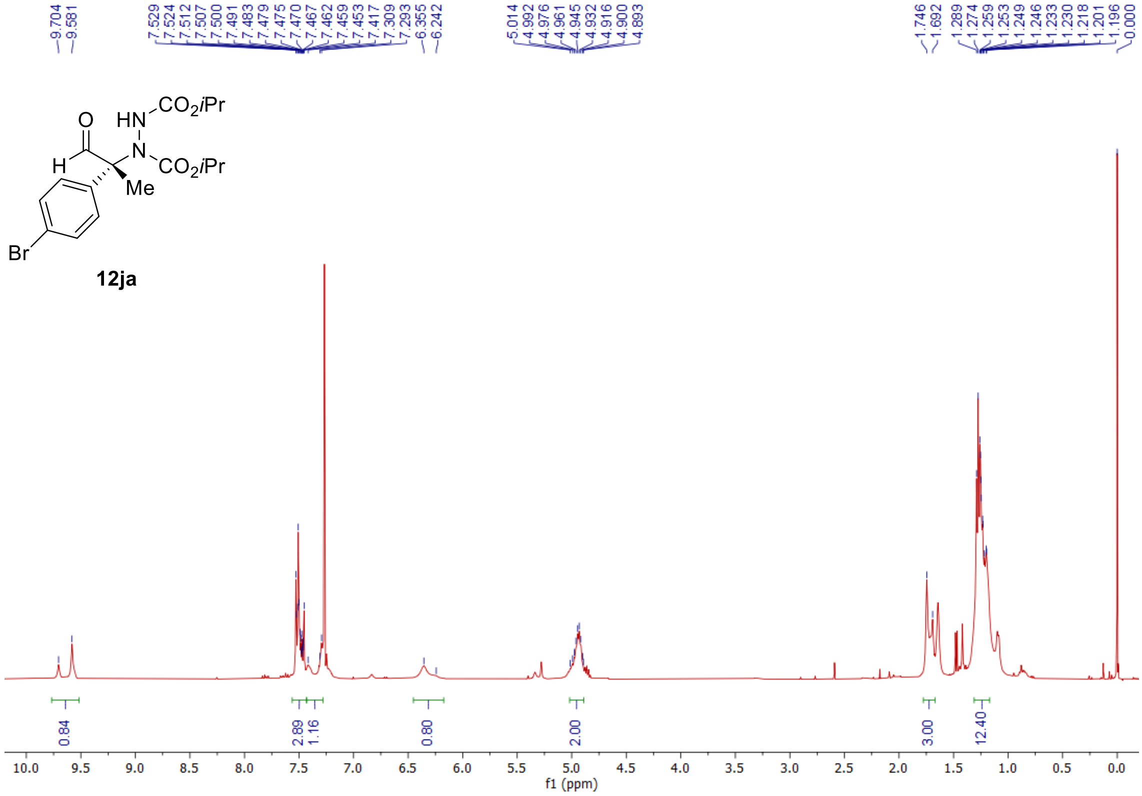

**Figure S38.** $^{1}$H NMR (400 MHz, $CDCl_3$) of compound **12ja**.

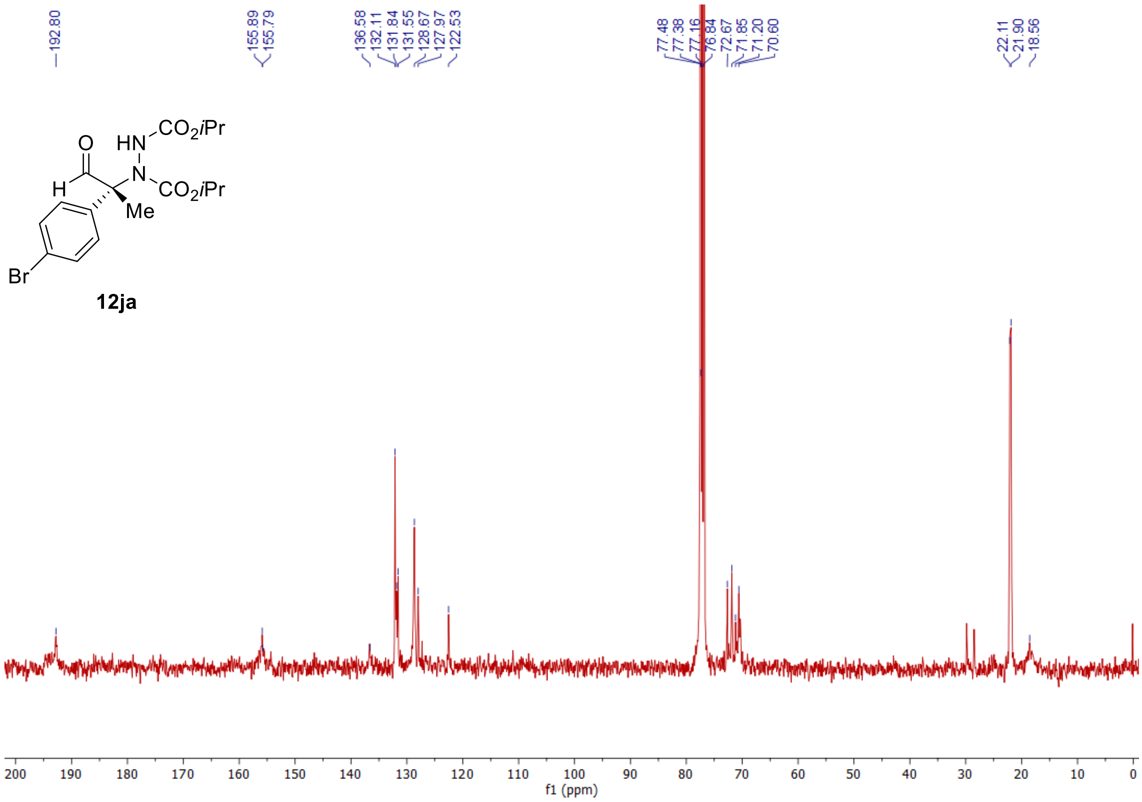

**Figure S39.** $^{13}C\{^{1}H\}$ NMR (101 MHz, $CDCl_3$) of compound **12ja**.

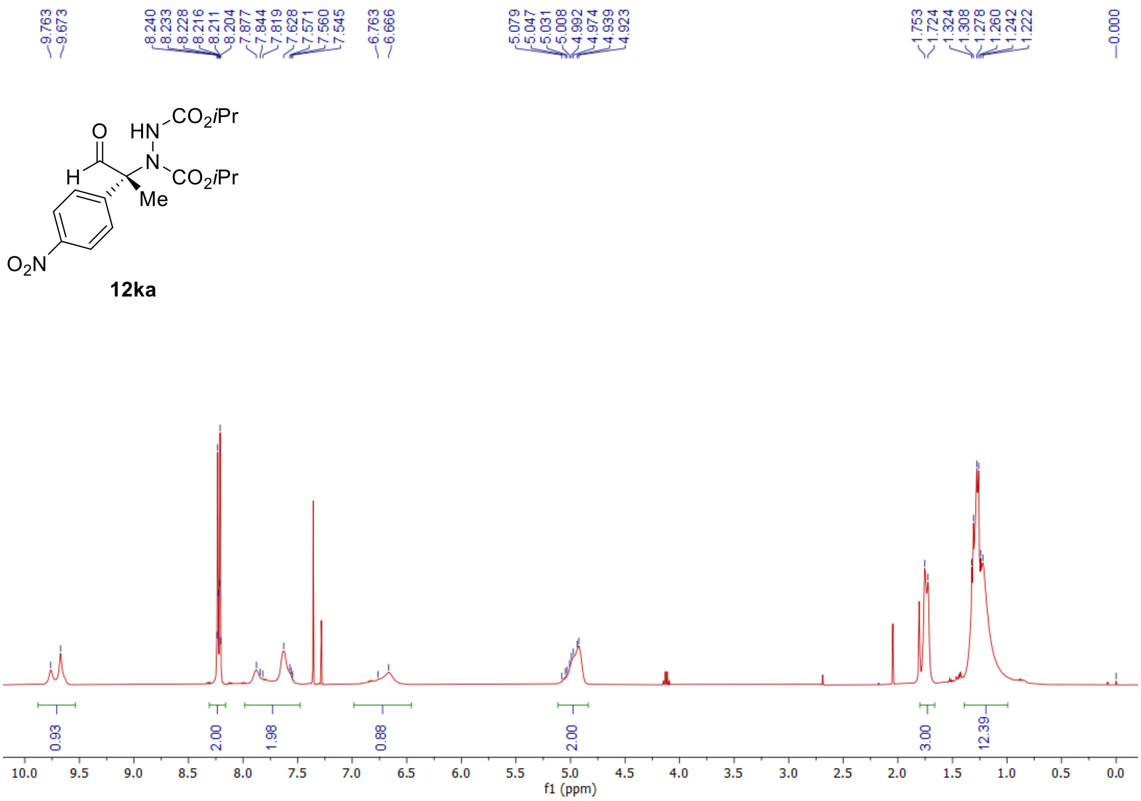


**Figure S40.** $^{1}$H NMR (400 MHz, $CDCl_3$) of compound **12ka**.

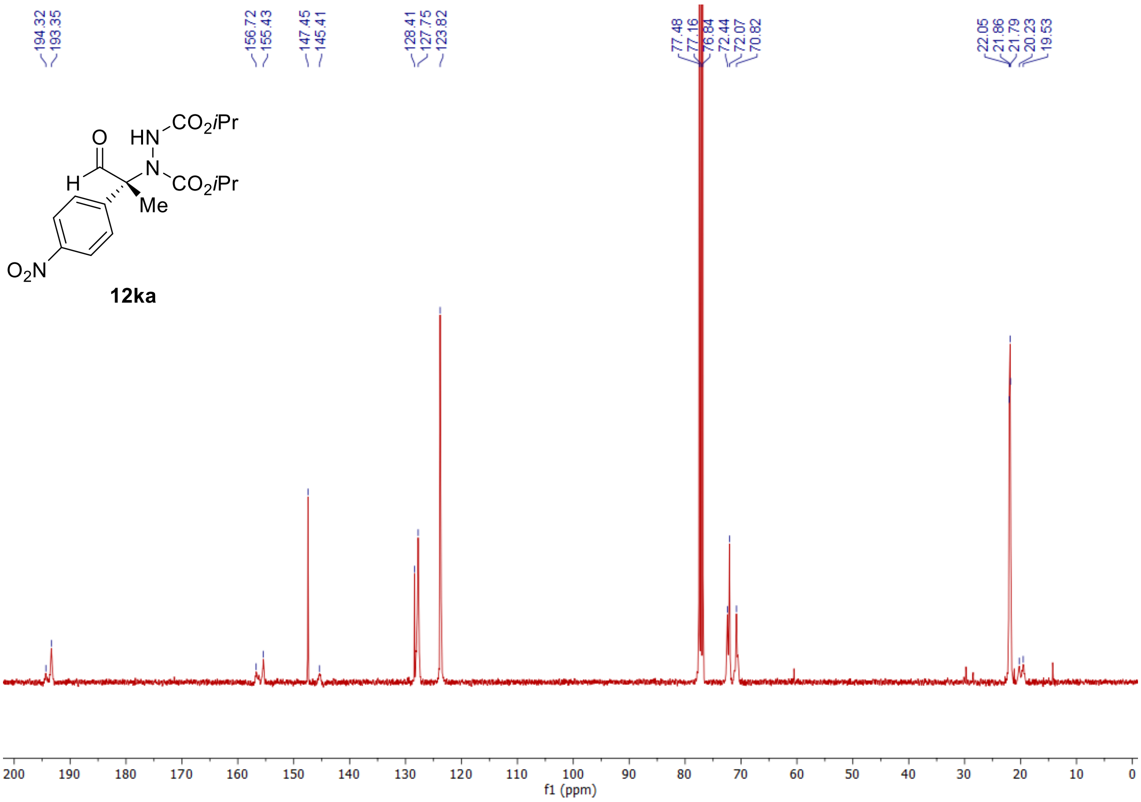


**Figure S41.** $^{13}C\{^{1}H\}$ NMR (101 MHz, $CDCl_3$) of compound **12ka**.

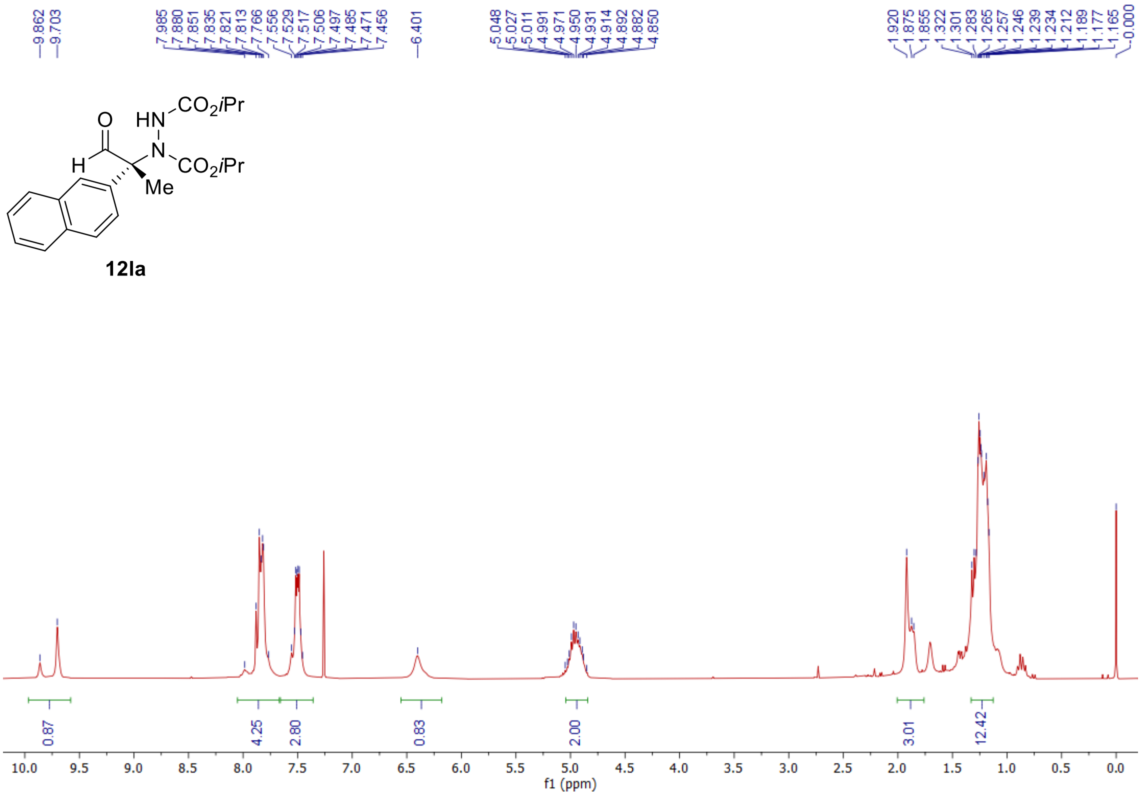


**Figure S42.** $^{1}H$ NMR (300 MHz, $CDCl_3$) of compound **12la**.

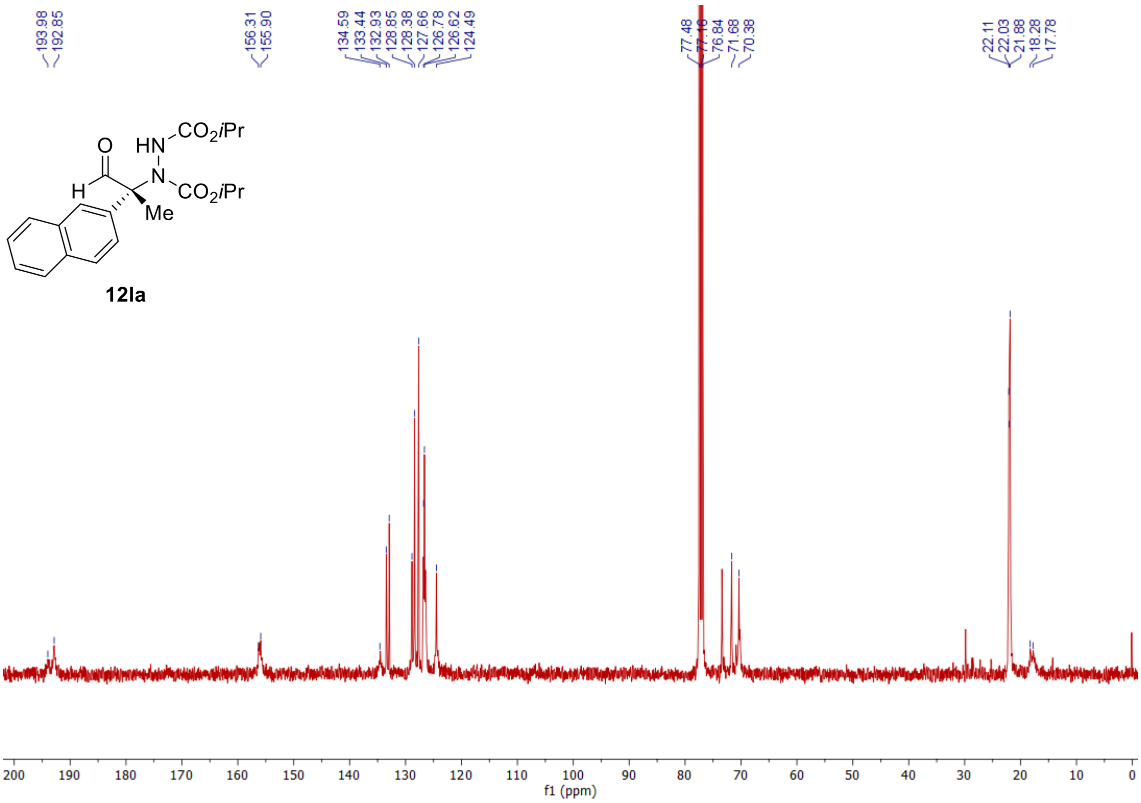


**Figure S43.** $^{13}C\{^{1}H\}$ NMR (75 MHz, $CDCl_3$) of compound **12la**.

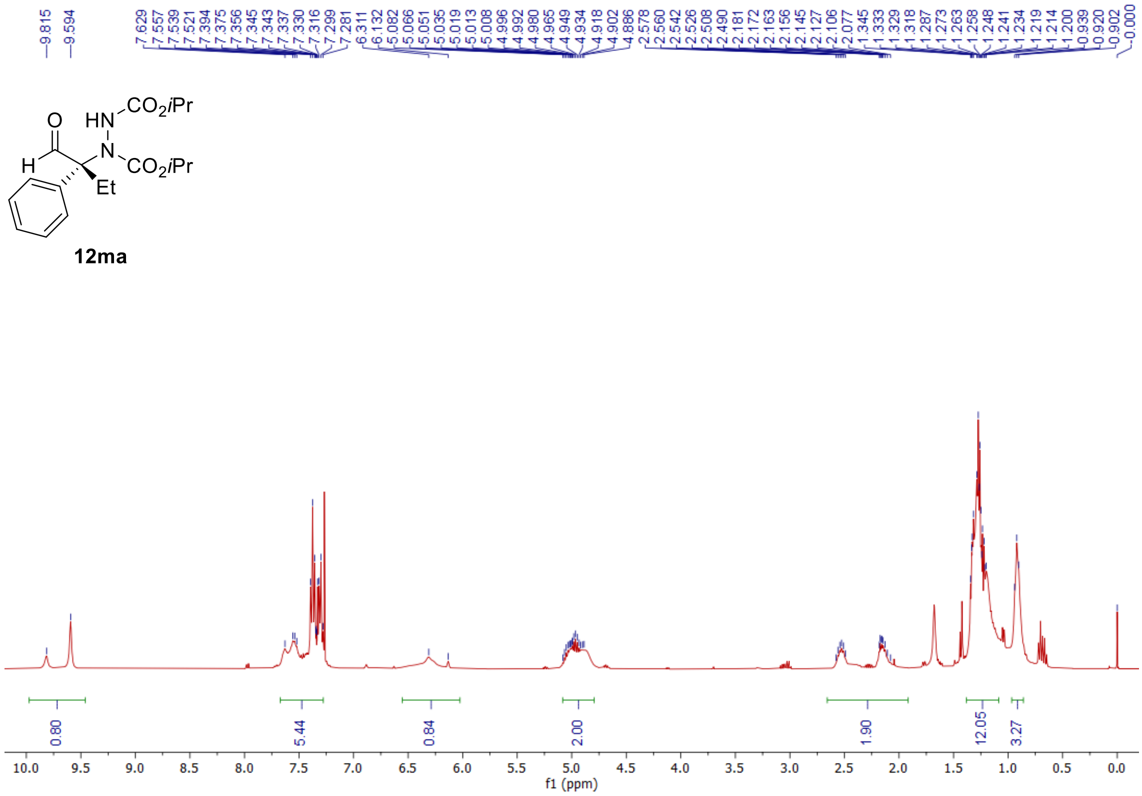


**Figure S44.** $^{1}H$ NMR (400 MHz, $CDCl_3$) of compound **12ma**.

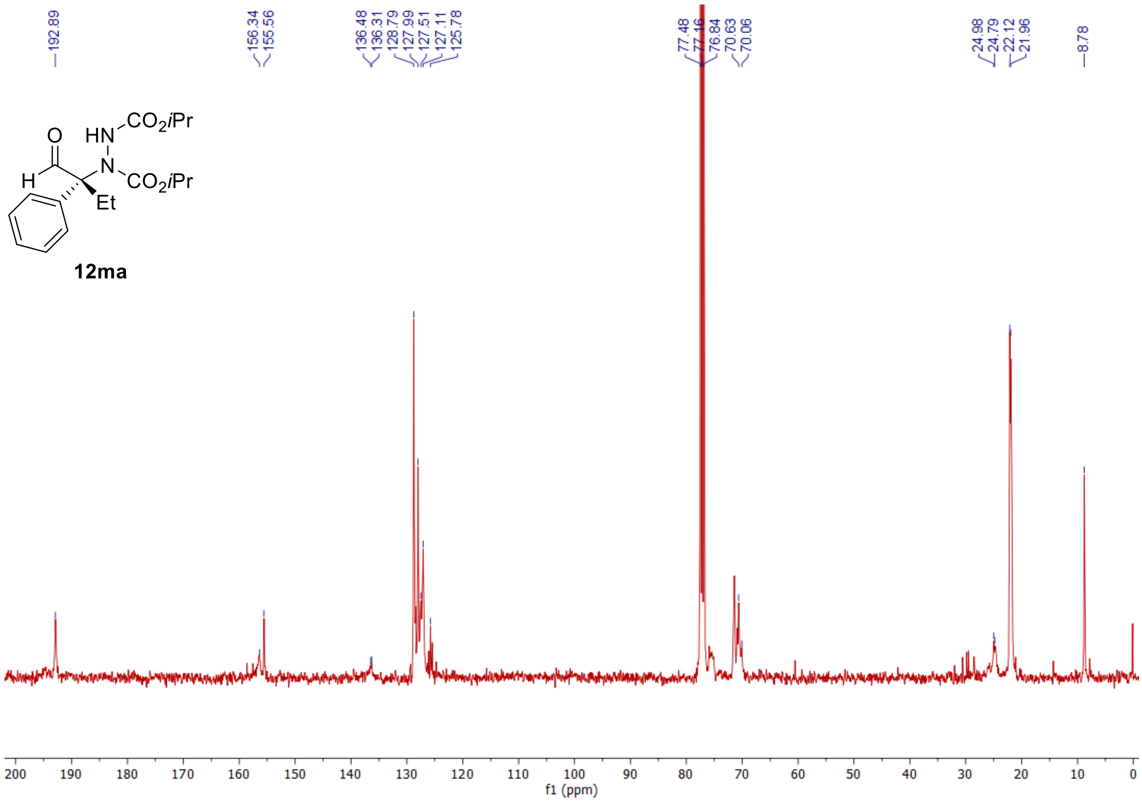


**Figure S45.** $^{13}C\{^{1}H\}$ NMR (101 MHz, $CDCl_3$) of compound **12ma**.

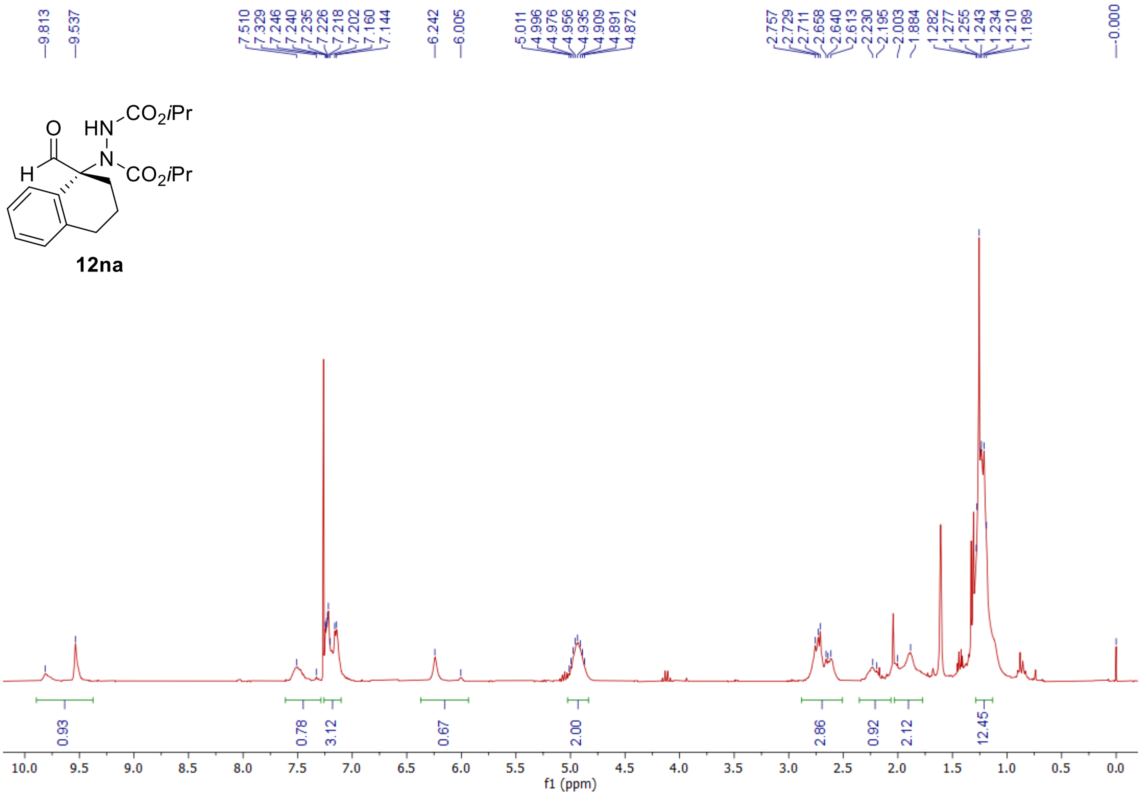


**Figure S46.** $^1$H NMR (300 MHz, $CDCl_3$) of compound **12na**.

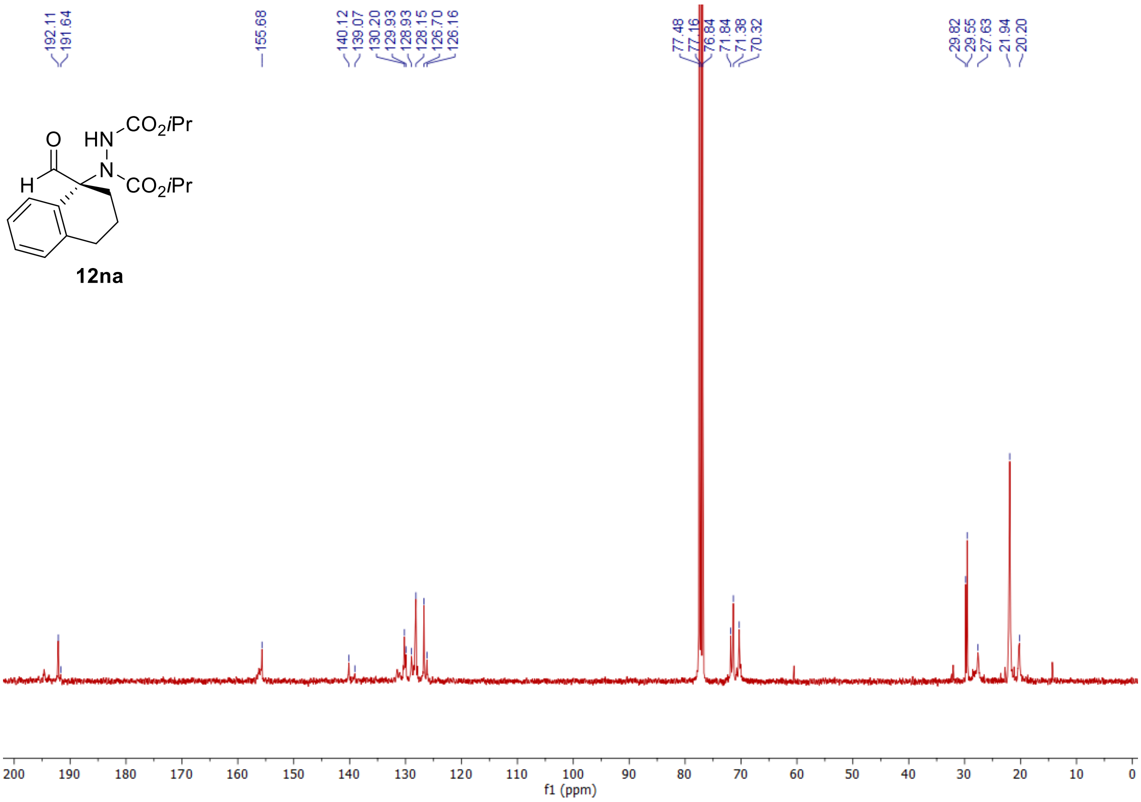


**Figure S47.** $^{13}C\{^1H\}$ NMR (75 MHz, $CDCl_3$) of compound **12na**.

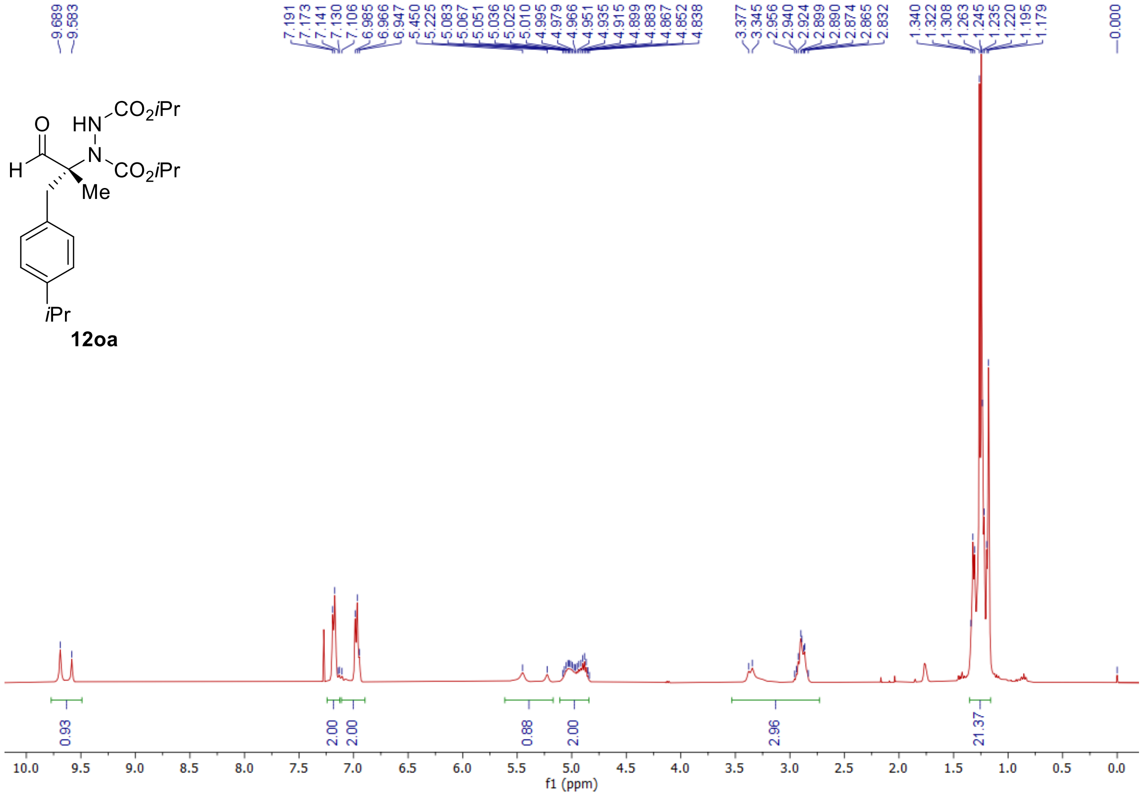

**Figure S48.** $^{1}$H NMR (400 MHz, $CDCl_3$) of compound **12oa**.

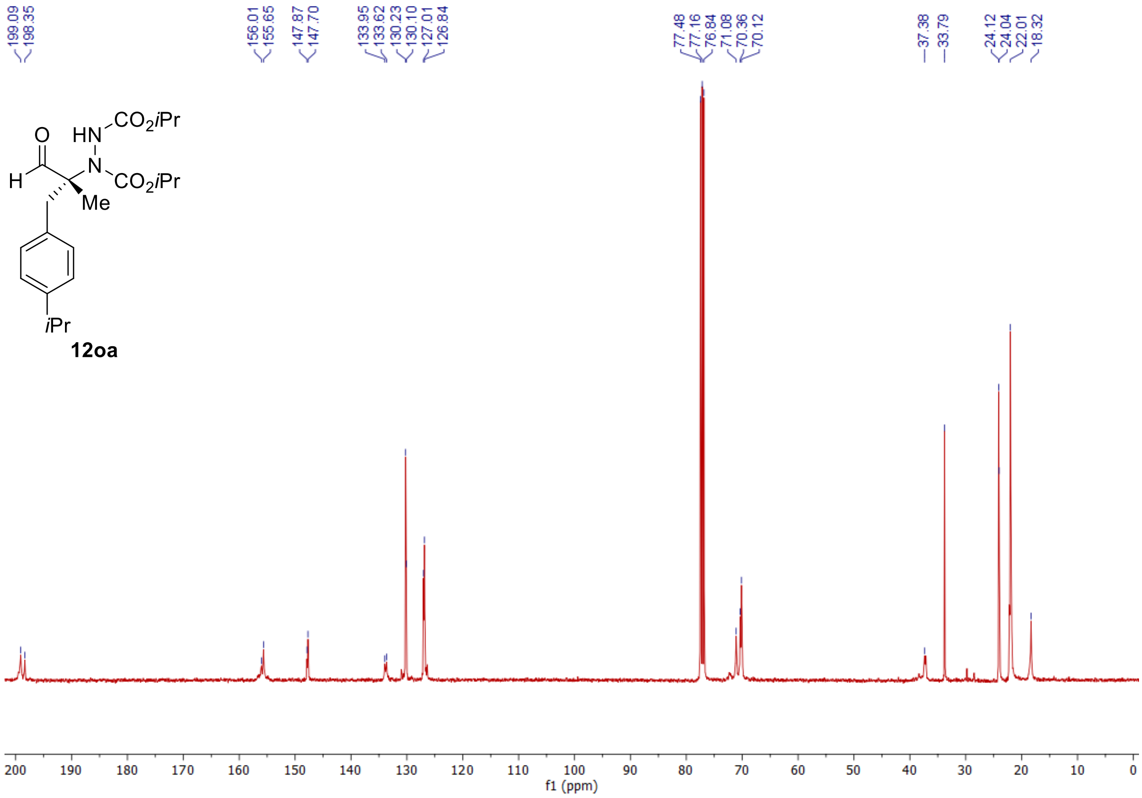

**Figure S49.** $^{13}C\{^{1}H\}$ NMR (101 MHz, $CDCl_3$) of compound **12oa**.

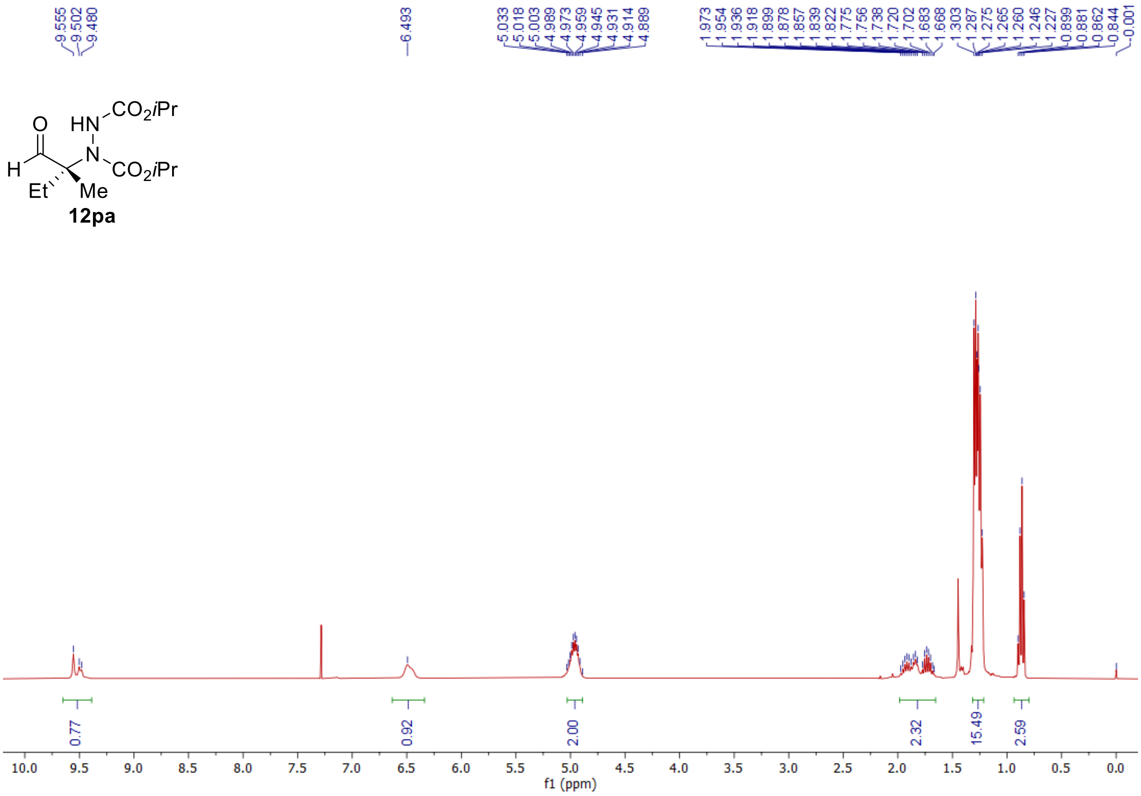


**Figure S50.** $^{1}H$ NMR (400 MHz, $CDCl_3$) of compound **12pa**.

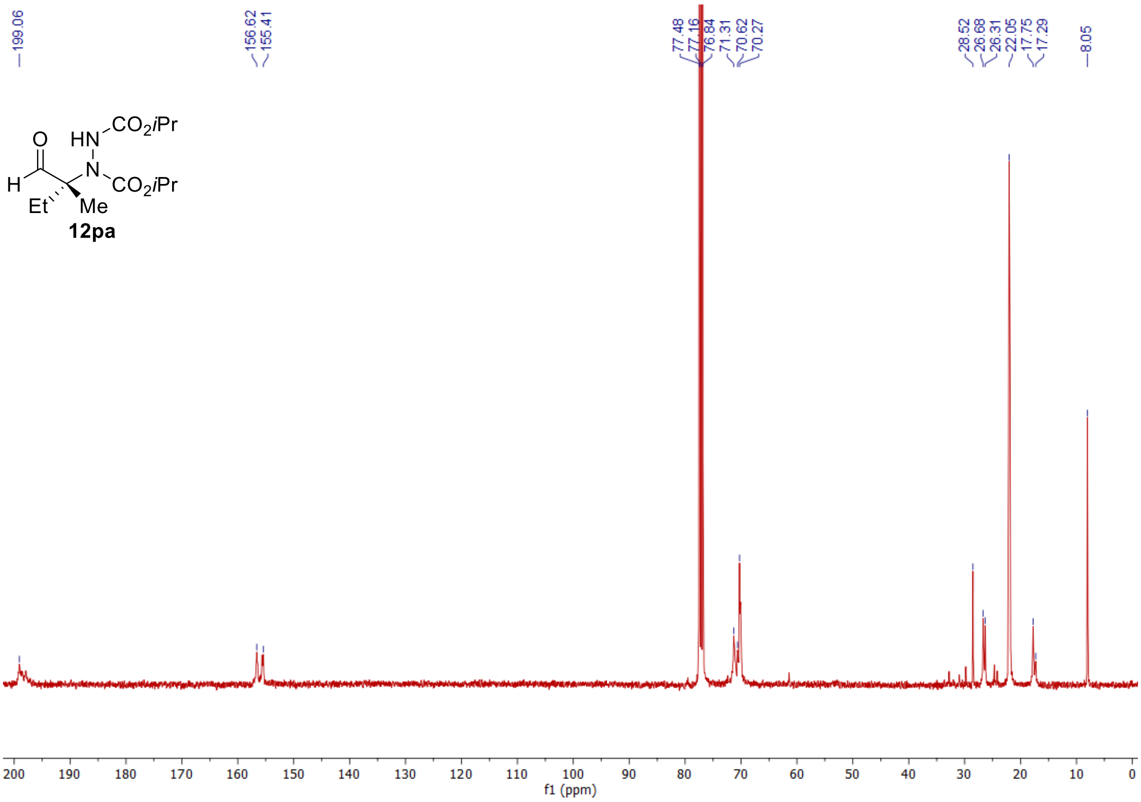


**Figure S51.** $^{13}C\{^{1}H\}$ NMR (101 MHz, $CDCl_3$) of compound **12pa**.

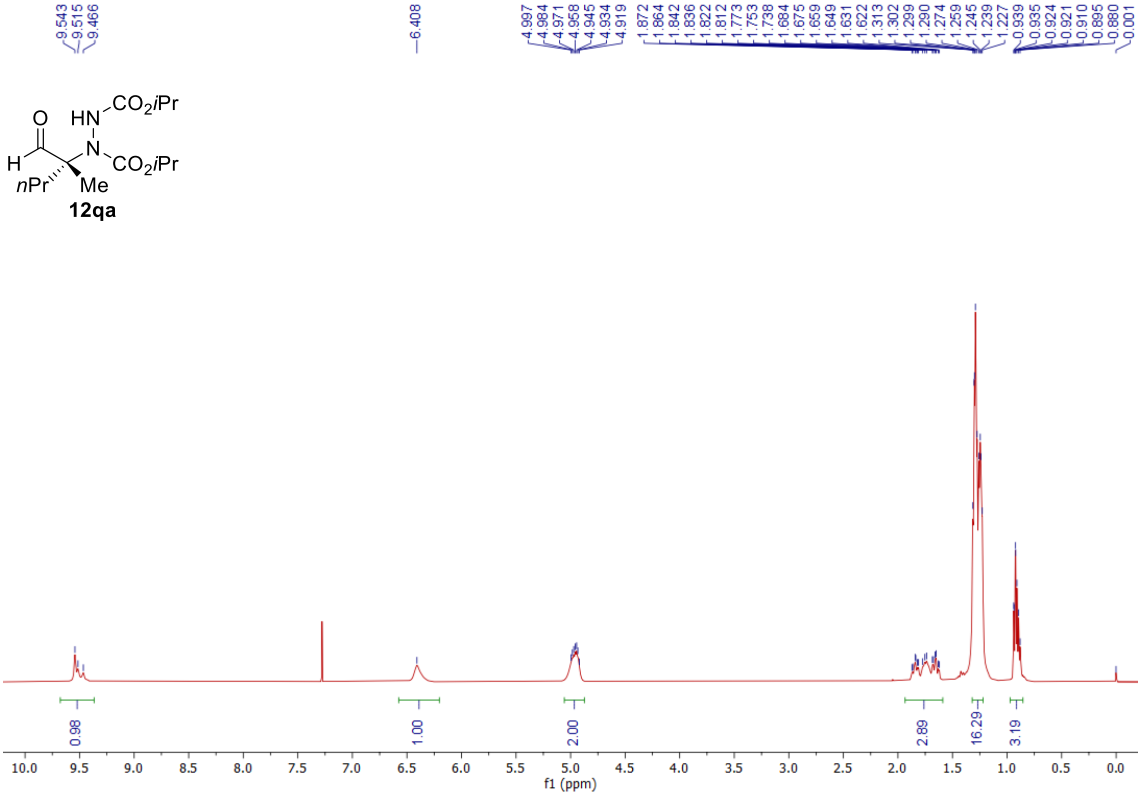


**Figure S52.** $^{1}H$ NMR (500 MHz, $CDCl_3$) of compound **12qa**.

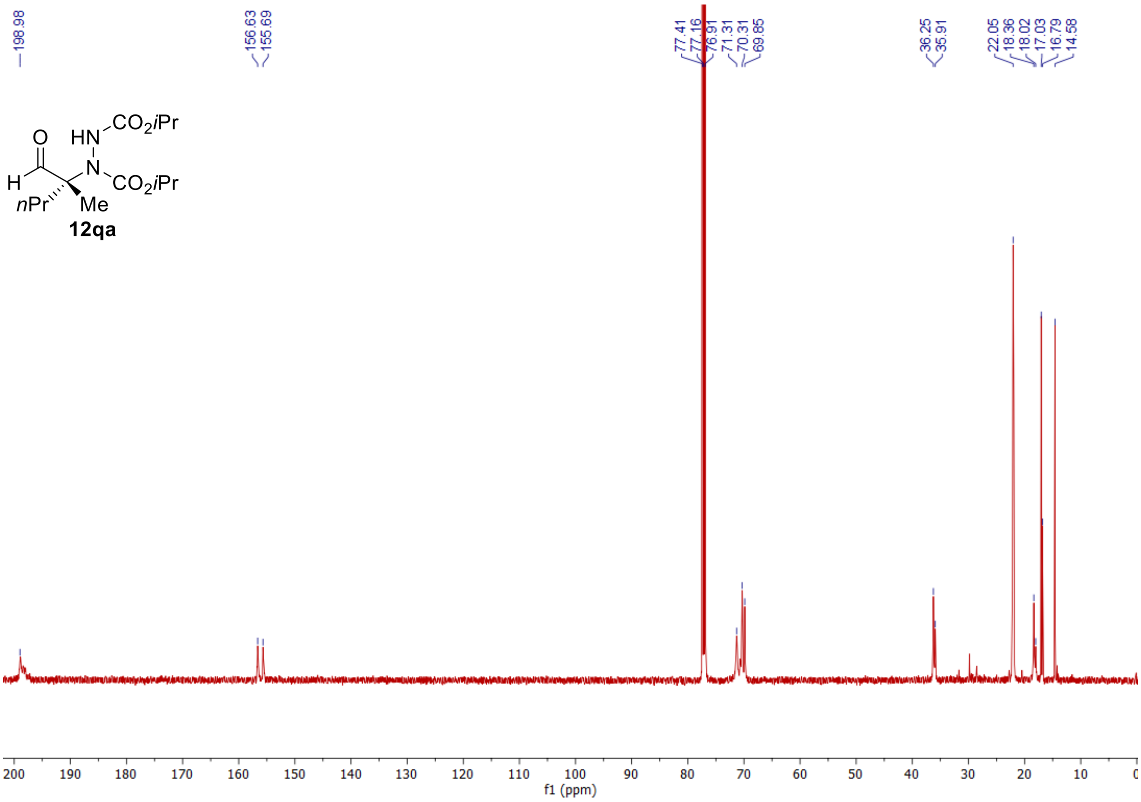


**Figure S53.** $^{13}C\{^{1}H\}$ NMR (125 MHz, $CDCl_3$) of compound **12qa**.

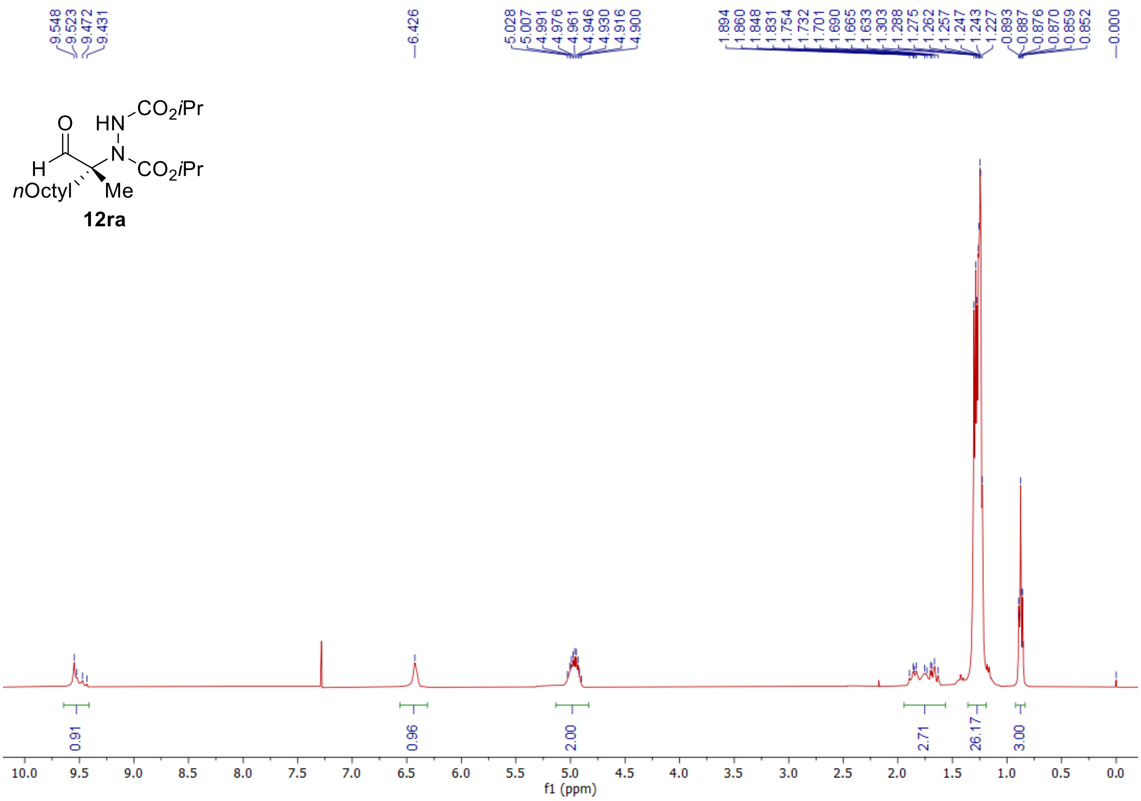

**Figure S54.** $^{1}H$ NMR (400 MHz, $CDCl_3$) of compound **12ra**.

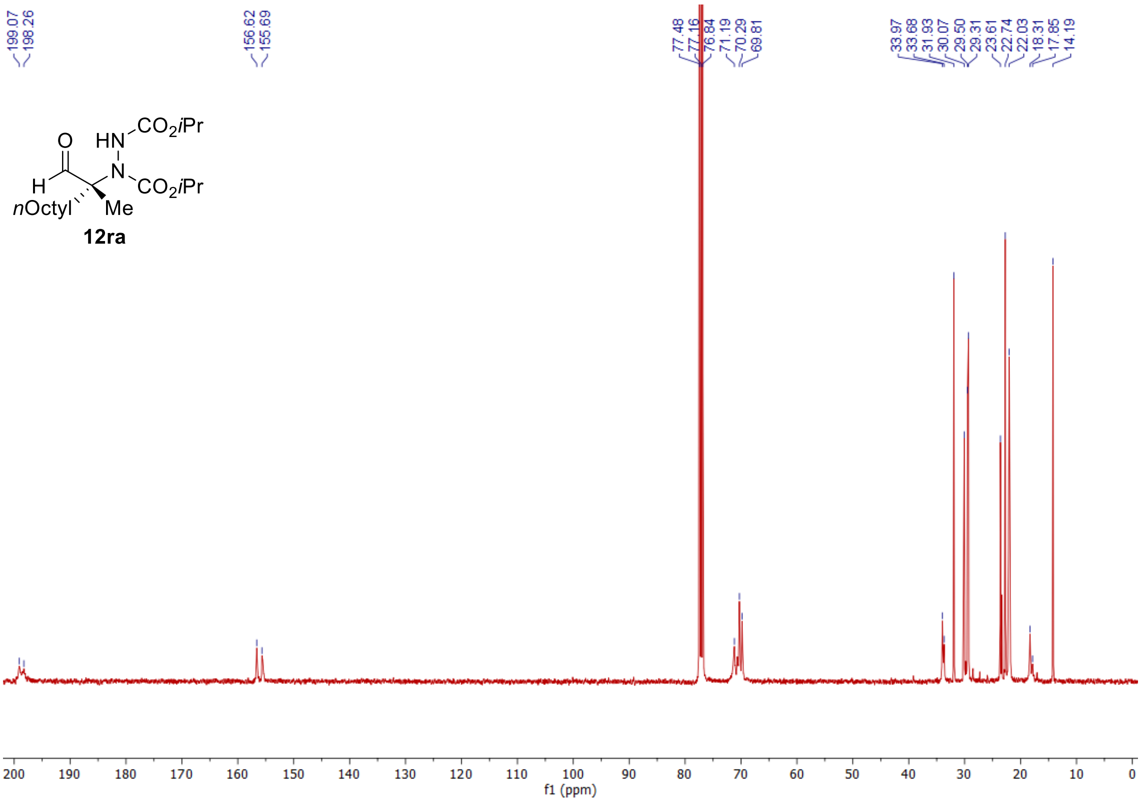

**Figure S55.** $^{13}C\{^{1}H\}$ NMR (101 MHz, $CDCl_3$) of compound **12ra**.

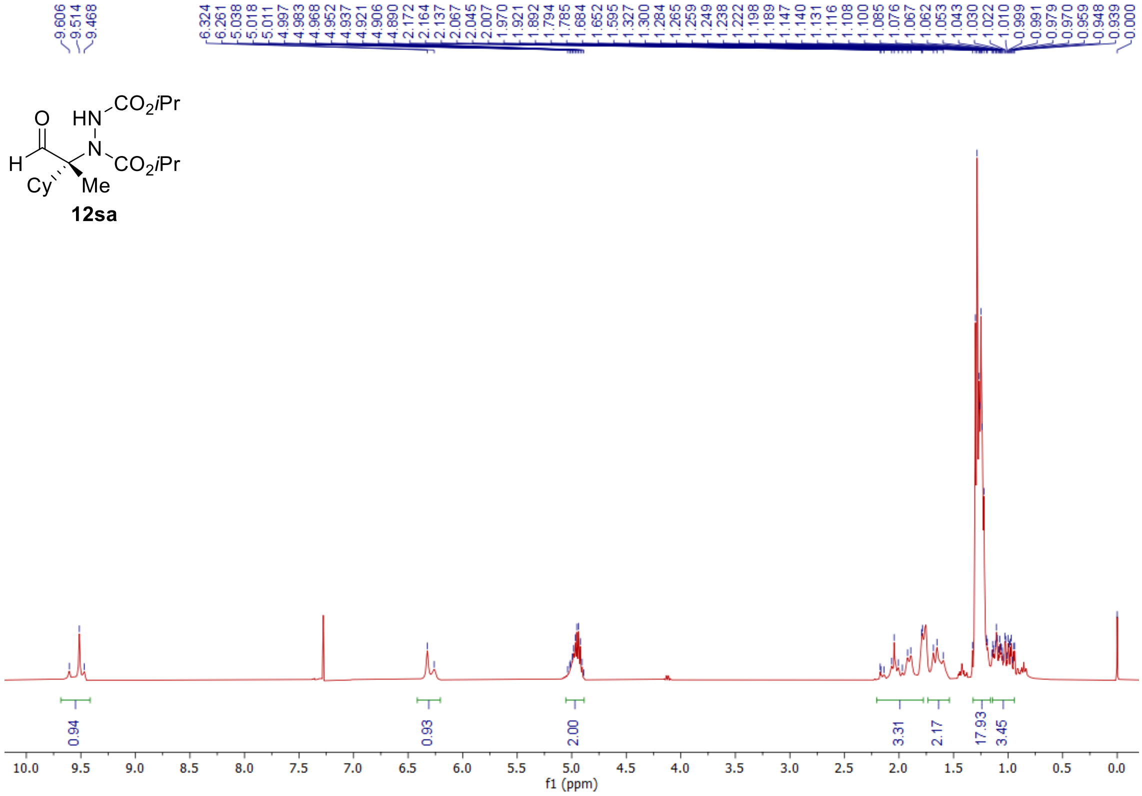

**Figure S56.** $^{1}H$ NMR (400 MHz, $CDCl_3$) of compound **12sa**.

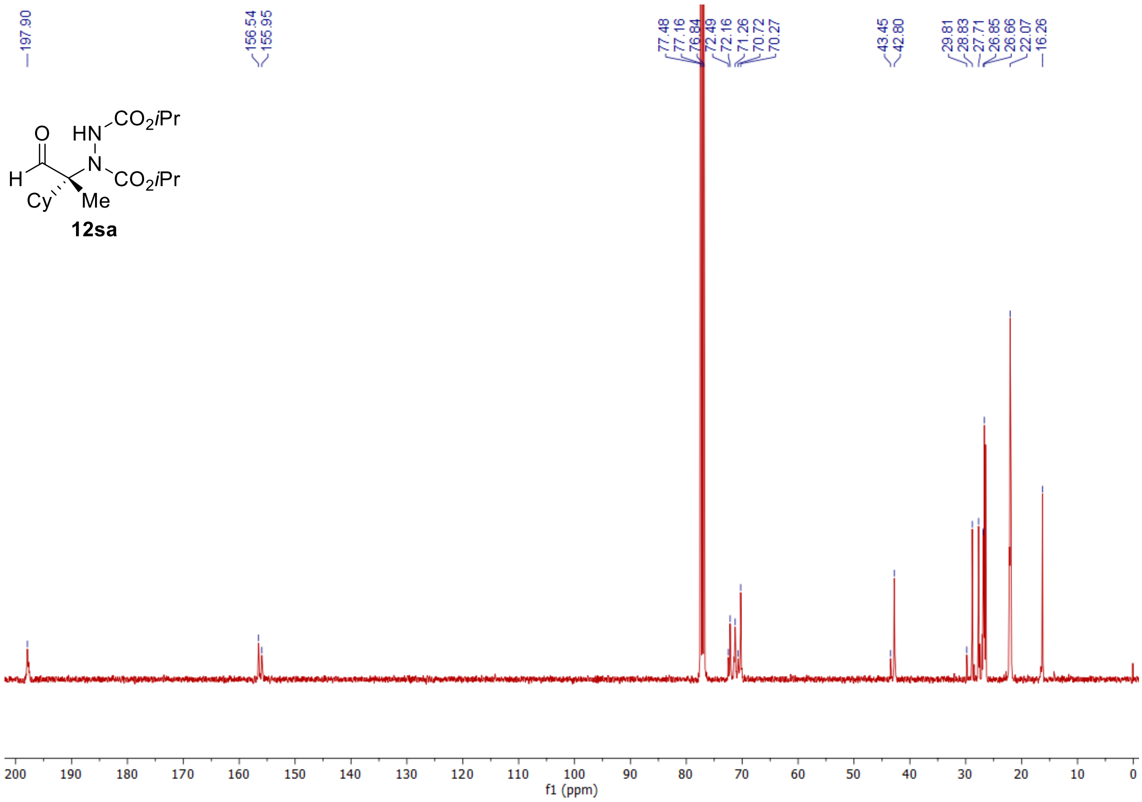

**Figure S57.** $^{13}C\{^{1}H\}$ NMR (101 MHz, $CDCl_3$) of compound **12sa**.

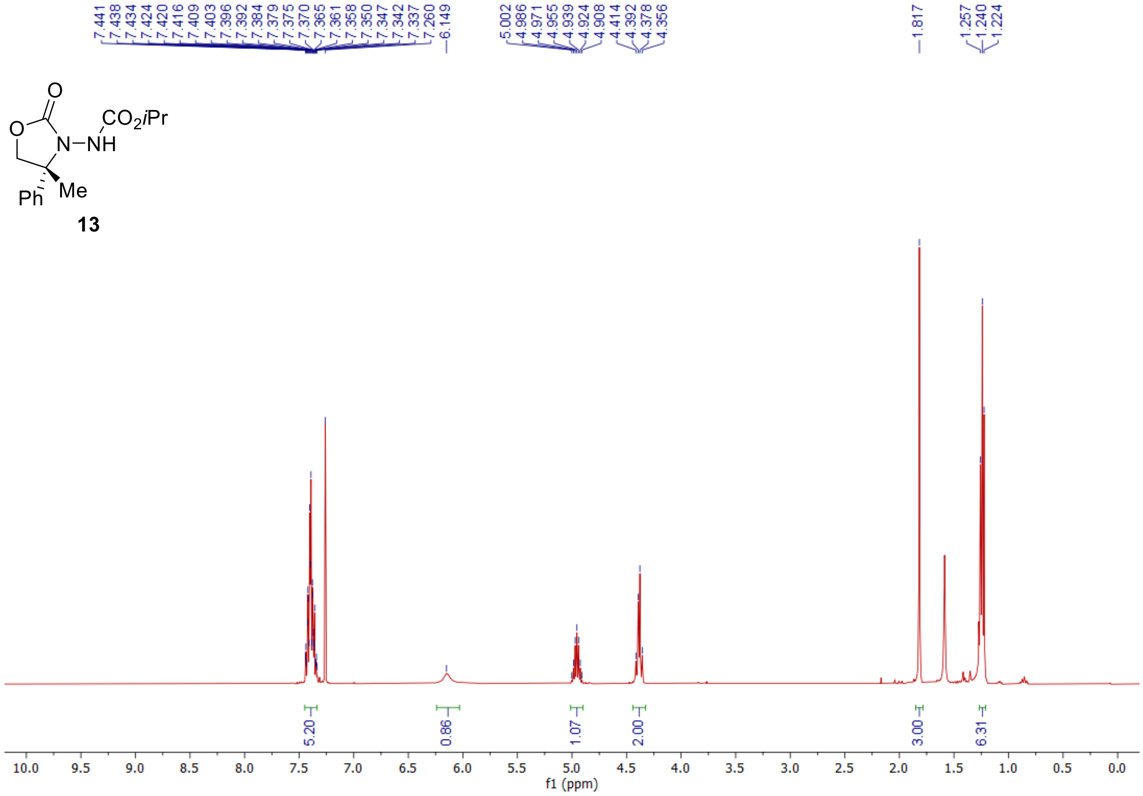


**Figure S58.** $^{1}H$ NMR (400 MHz, $CDCl_3$) of compound **13**.

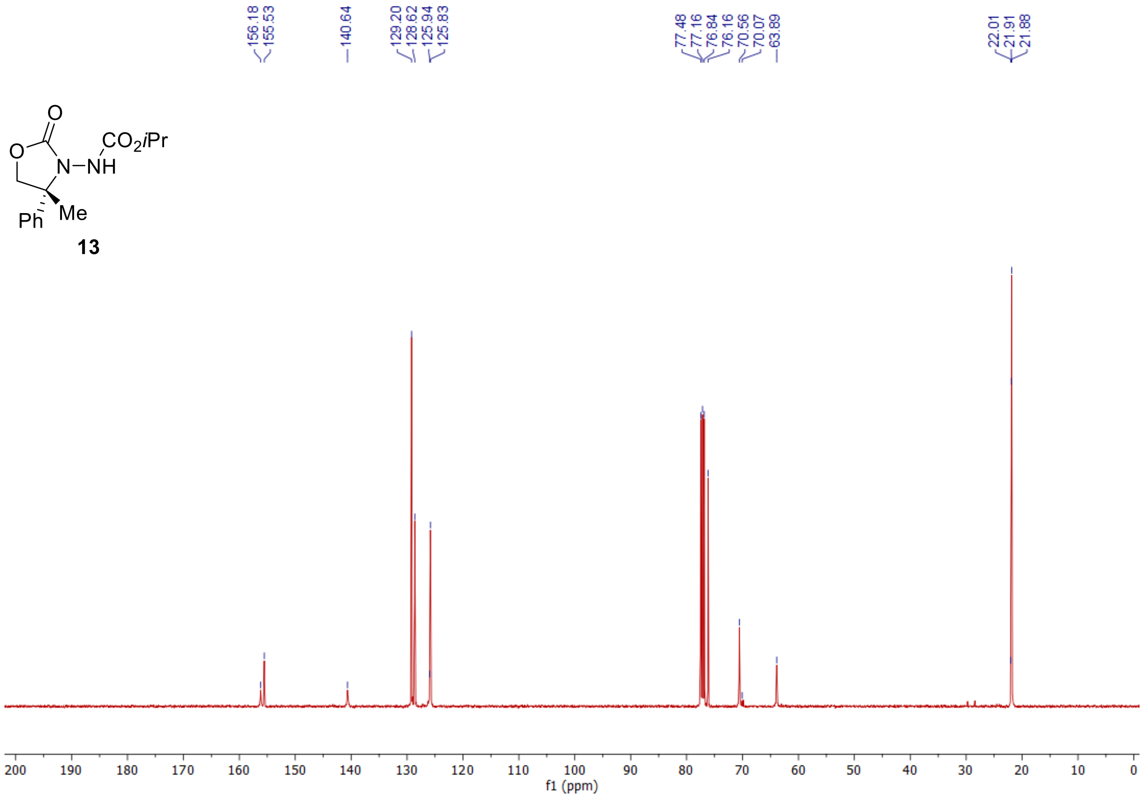


**Figure S59.** $^{13}C\{^{1}H\}$ NMR (101 MHz, $CDCl_3$) of compound **13**.

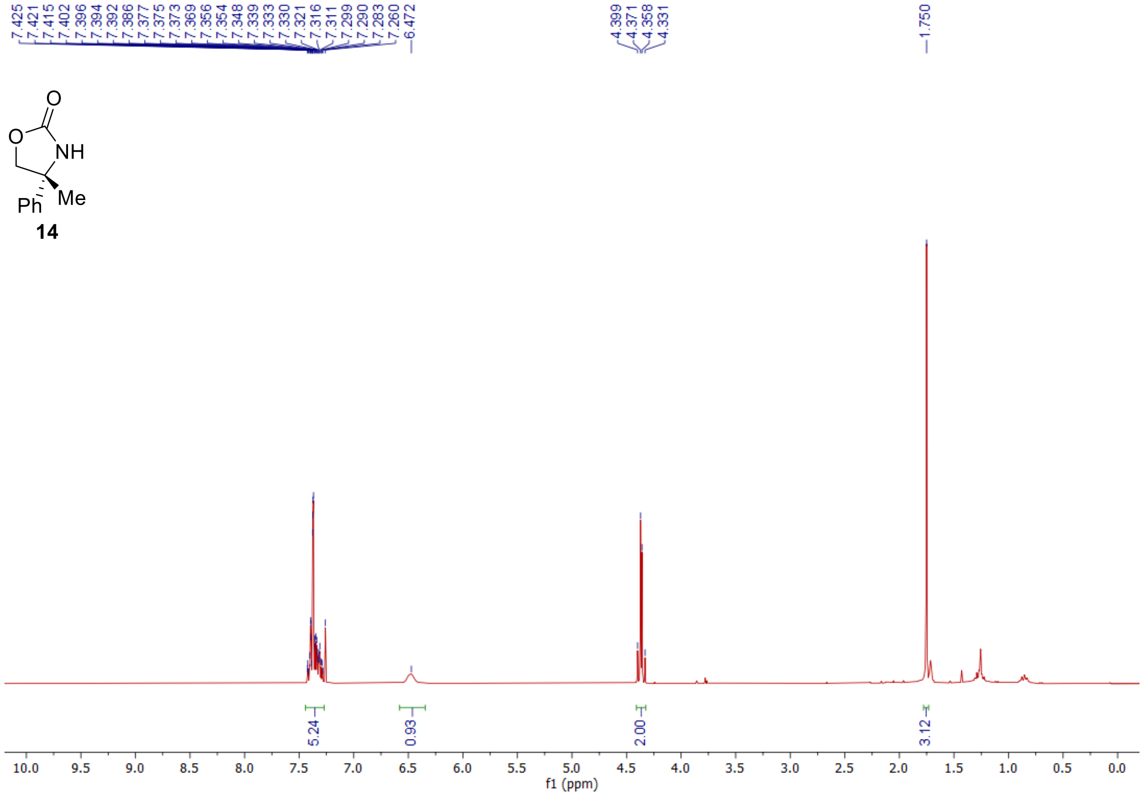


**Figure S60.** $^{1}$H NMR (300 MHz, $CDCl_3$) of compound **14**.

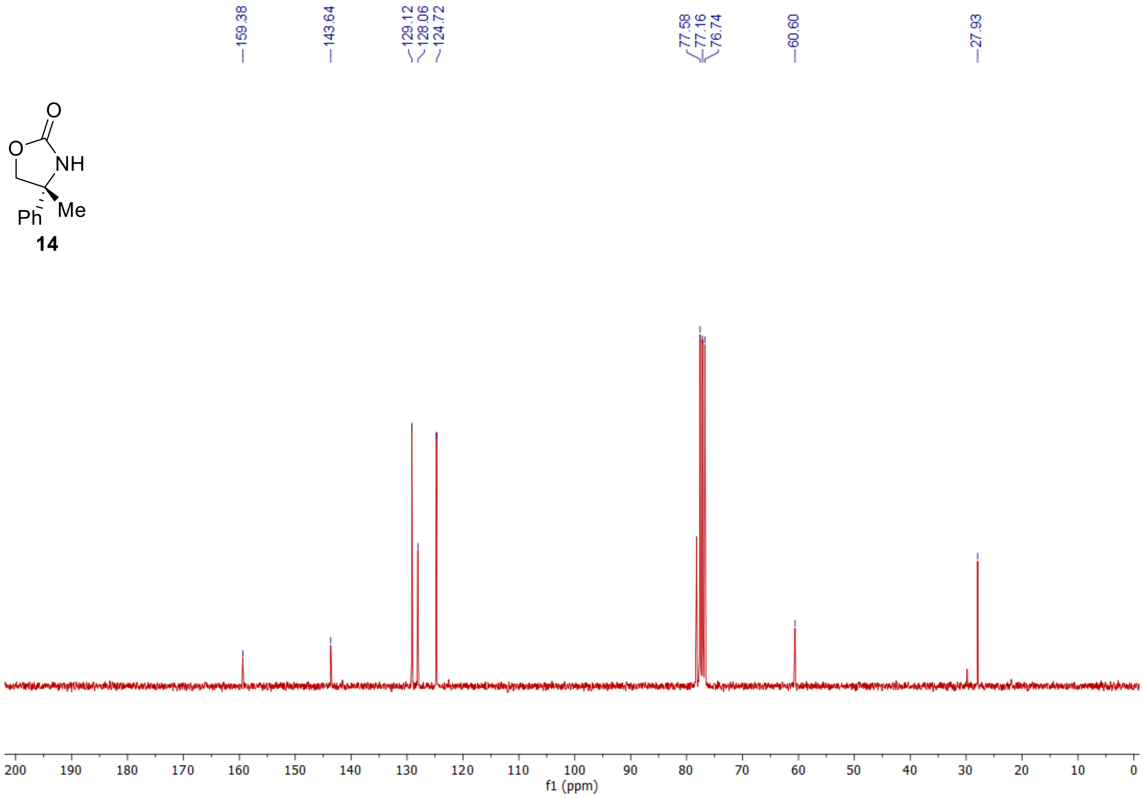


**Figure S61.** $^{13}C\{^{1}H\}$ NMR (75 MHz, $CDCl_3$) of compound **14**.

VII. HPLC chromatograms of compounds **12**, **13** and **14**

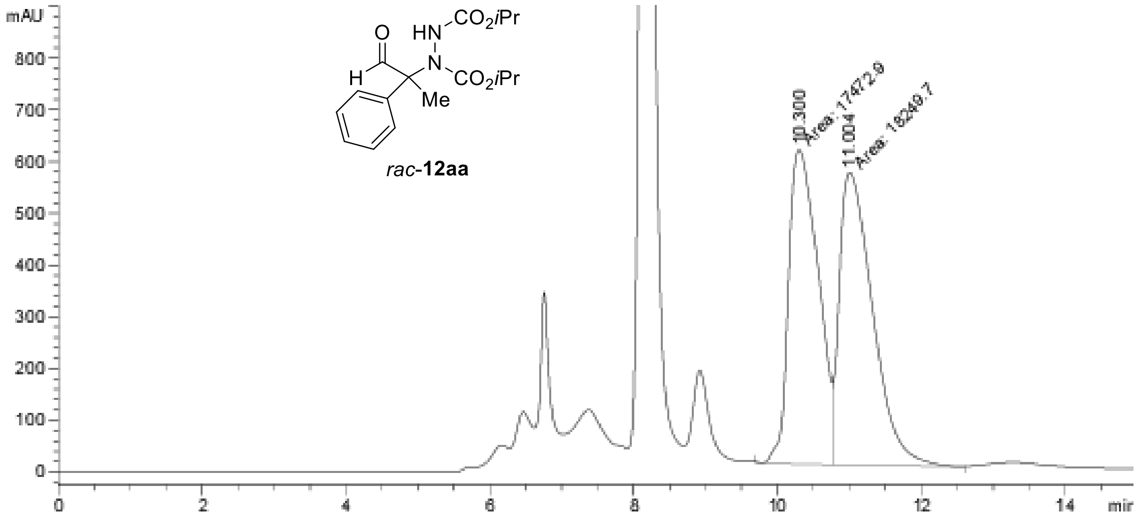


| Peak # | RetTime [min] | Type | Width [min] | Area [mAU*s] | Height [mAU] | Area % |
|---|---|---|---|---|---|---|
| 1 | 10.300 | MF | 0.4794 | 1.74729e4 | 607.49402 | 48.9127 |
| 2 | 11.004 | FM | 0.5383 | 1.82497e4 | 565.04242 | 51.0873 |

**Figure S62.** HPLC chromatogram of compound *rac*-**12aa**.

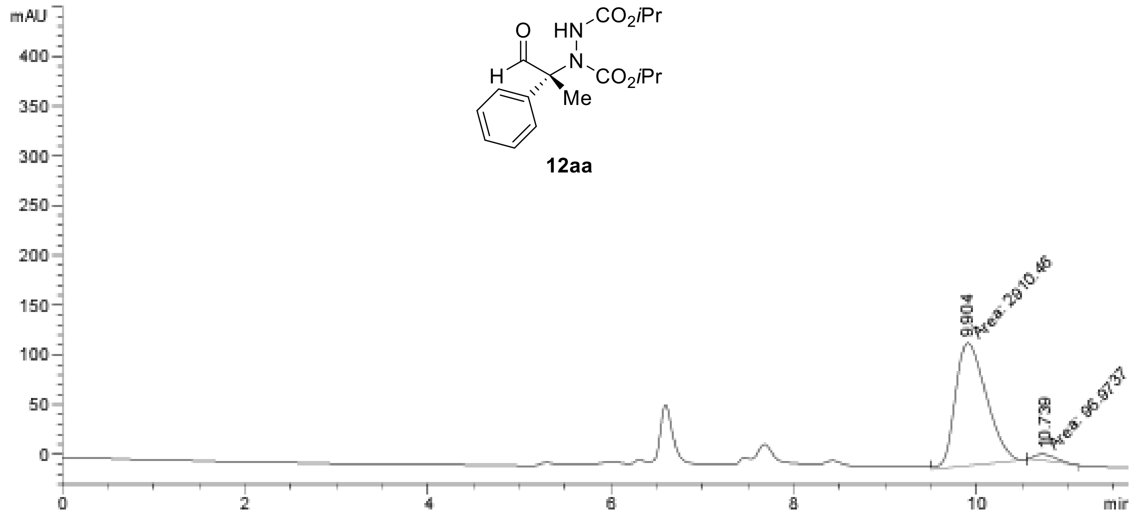


| Peak # | RetTime [min] | Type | Width [min] | Area [mAU*s] | Height [mAU] | Area % |
|---|---|---|---|---|---|---|
| 1 | 9.904 | MM | 0.3932 | 2910.46240 | 123.35352 | 96.7755 |
| 2 | 10.739 | MM | 0.2585 | 96.97370 | 6.25138 | 3.2245 |

**Figure S63.** HPLC chromatogram of compound **12aa**.

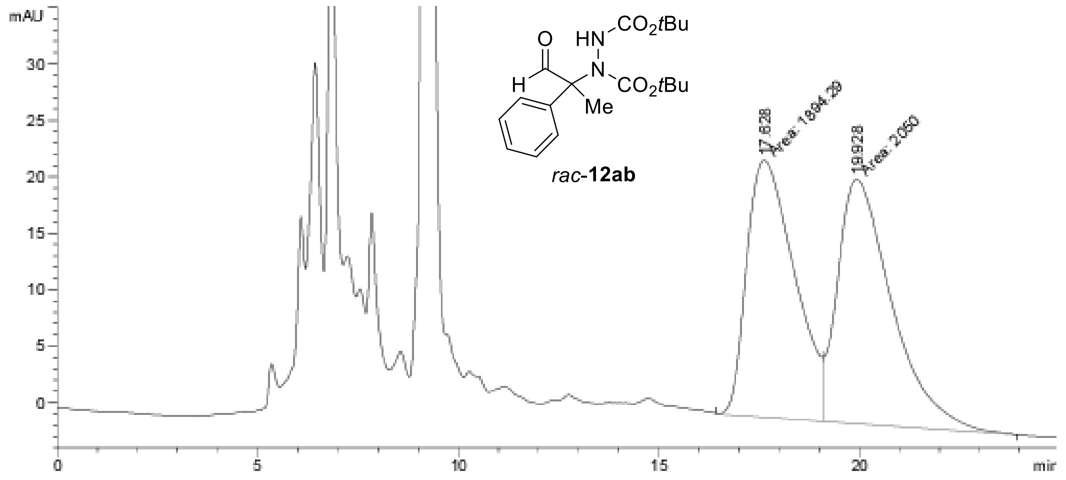


| Peak # | RetTime [min] | Type | Width [min] | Area [mAU*s] | Height [mAU] | Area % |
|---|---|---|---|---|---|---|
| 1 | 17.628 | MF | 1.3883 | 1894.29016 | 22.74179 | 48.0262 |
| 2 | 19.928 | FM | 1.5781 | 2049.99561 | 21.65088 | 51.9738 |

**Figure S64.** HPLC chromatogram of compound *rac*-**12ab**.

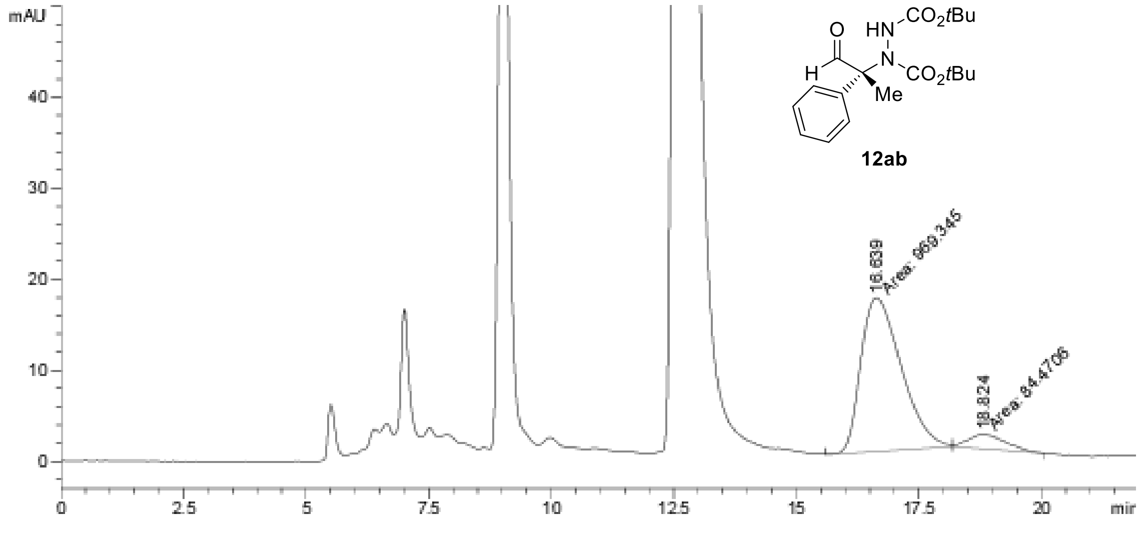


| Peak # | RetTime [min] | Type | Width [min] | Area [mAU*s] | Height [mAU] | Area % |
|---|---|---|---|---|---|---|
| 1 | 16.639 | MM | 0.9564 | 969.34534 | 16.89276 | 91.9843 |
| 2 | 18.824 | MM | 0.8894 | 84.47057 | 1.58290 | 8.0157 |

**Figure S65.** HPLC chromatogram of compound **12ab**.

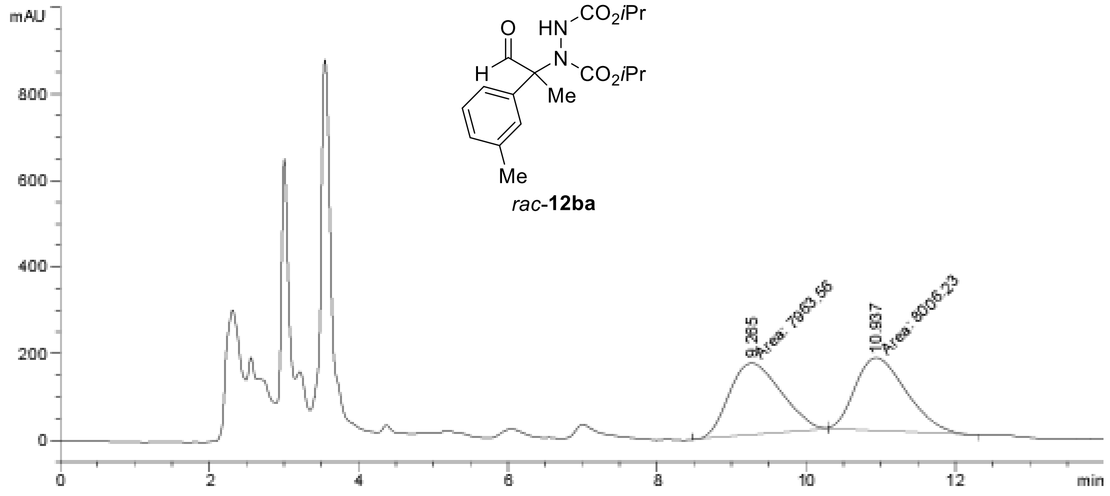


| Peak # | RetTime [min] | Type | Width [min] | Area [mAU*s] | Height [mAU] | Area % |
|---|---|---|---|---|---|---|
| 1 | 9.265 | MM | 0.8048 | 7963.55615 | 164.91283 | 49.8664 |
| 2 | 10.937 | MM | 0.7948 | 8006.22705 | 167.89563 | 50.1336 |

**Figure S66.** HPLC chromatogram of compound *rac*-**12ba**.

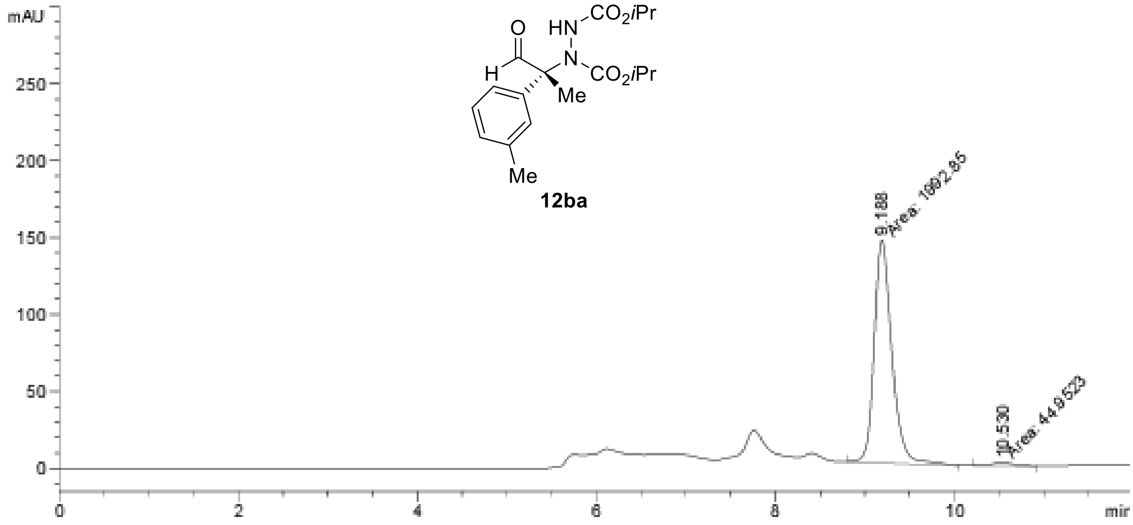


| Peak # | RetTime [min] | Type | Width [min] | Area [mAU*s] | Height [mAU] | Area % |
|---|---|---|---|---|---|---|
| 1 | 9.188 | MM | 0.2293 | 1992.85461 | 144.83578 | 97.7941 |
| 2 | 10.530 | MM | 0.3634 | 44.95228 | 2.06173 | 2.2059 |

**Figure S67.** HPLC chromatogram of compound **12ba**.

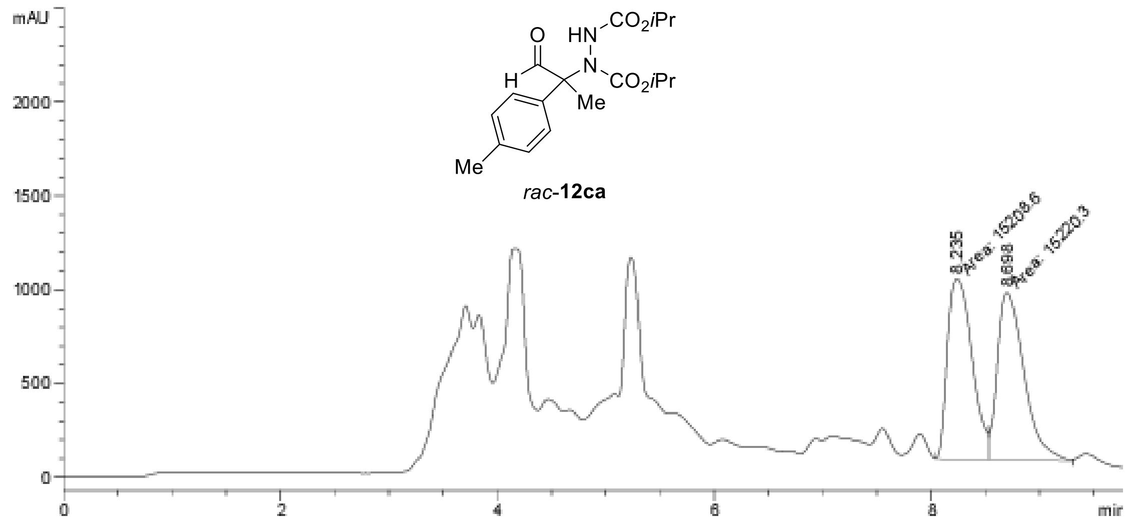


| Peak # | RetTime [min] | Type | Width [min] | Area [mAU*s] | Height [mAU] | Area % |
|---|---|---|---|---|---|---|
| 1 | 8.235 | MF | 0.2635 | 1.52086e4 | 961.94586 | 49.9807 |
| 2 | 8.698 | FM | 0.2845 | 1.52203e4 | 891.73889 | 50.0193 |

**Figure S68.** HPLC chromatogram of compound *rac*-**12ca**.

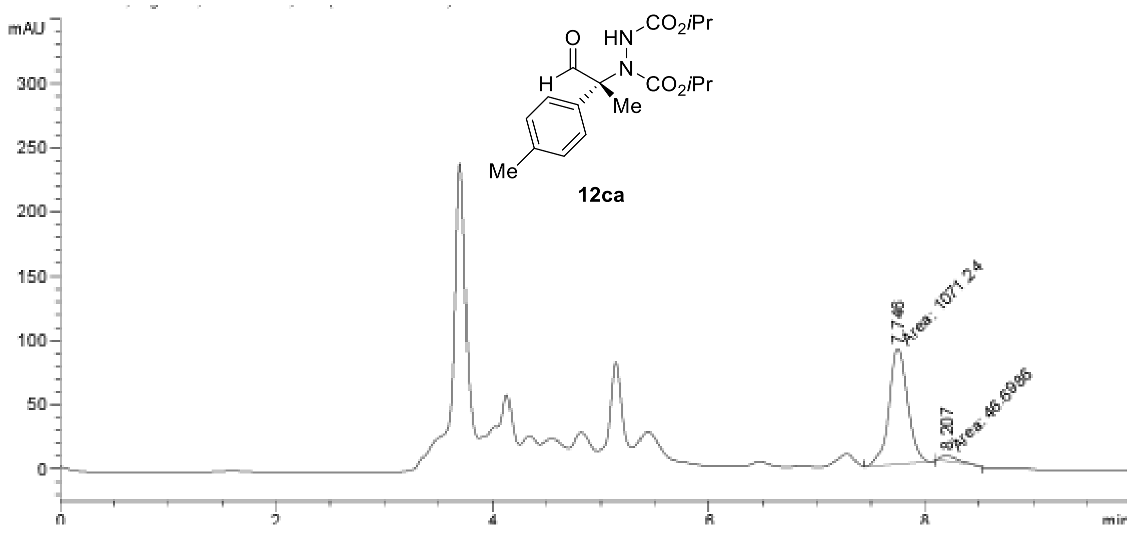


| Peak # | RetTime [min] | Type | Width [min] | Area [mAU*s] | Height [mAU] | Area % |
|---|---|---|---|---|---|---|
| 1 | 7.746 | MM | 0.1983 | 1071.24219 | 90.03523 | 95.8228 |
| 2 | 8.207 | MM | 0.1820 | 46.69865 | 4.27711 | 4.1772 |

**Figure S69.** HPLC chromatogram of compound **12ca**.

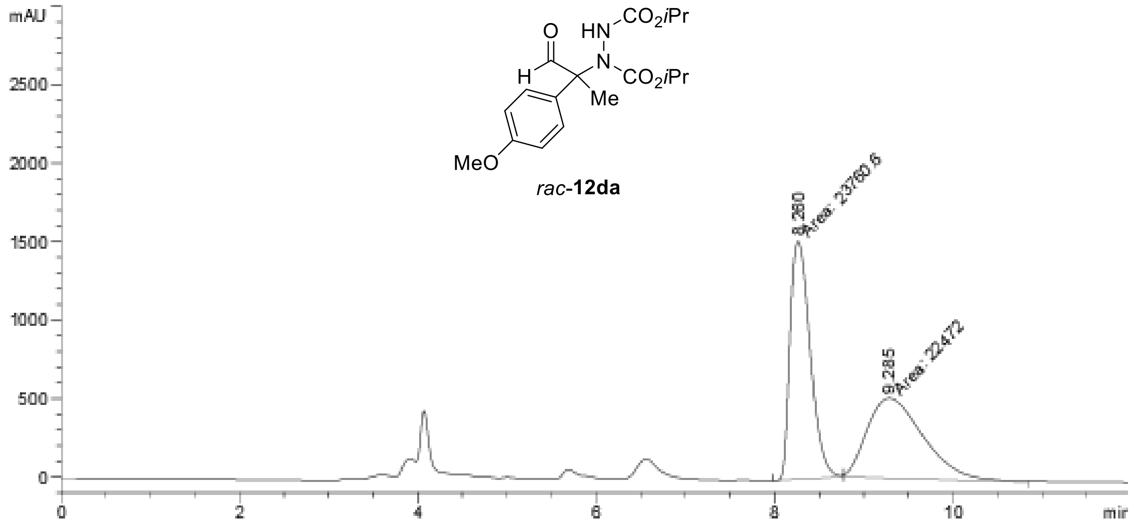


| Peak # | RetTime [min] | Type | Width [min] | Area [mAU*s] | Height [mAU] | Area % |
|---|---|---|---|---|---|---|
| 1 | 8.260 | MM | 0.2611 | 2.37606e4 | 1516.42358 | 51.3936 |
| 2 | 9.285 | MM | 0.7336 | 2.24720e4 | 510.52960 | 48.6064 |

**Figure S70.** HPLC chromatogram of compound *rac*-**12da**.

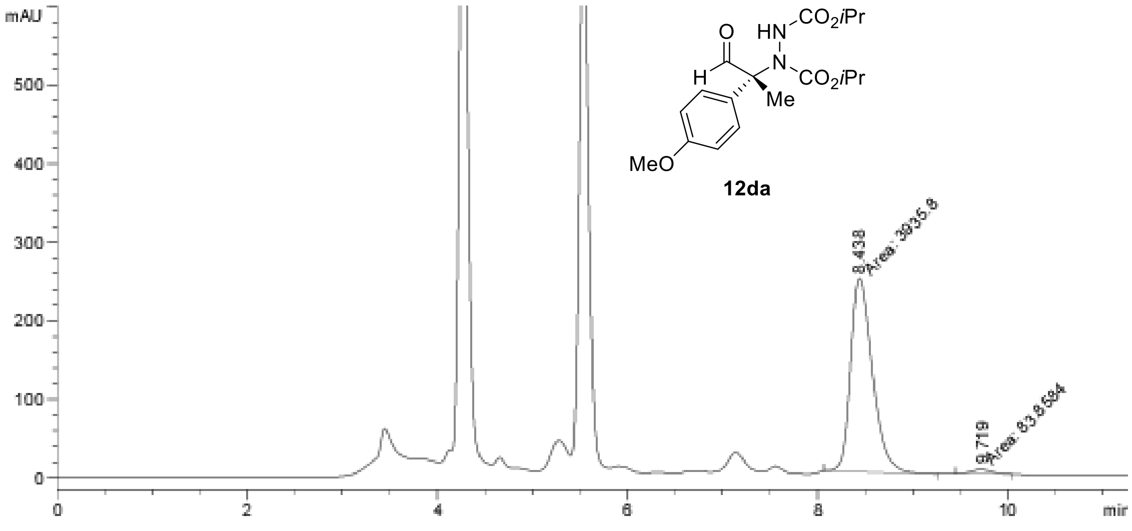


| Peak # | RetTime [min] | Type | Width [min] | Area [mAU*s] | Height [mAU] | Area % |
|---|---|---|---|---|---|---|
| 1 | 8.438 | MM | 0.2661 | 3935.79517 | 246.50224 | 97.9138 |
| 2 | 9.719 | MM | 0.2514 | 83.85837 | 5.55849 | 2.0862 |

**Figure S71.** HPLC chromatogram of compound **12da**.

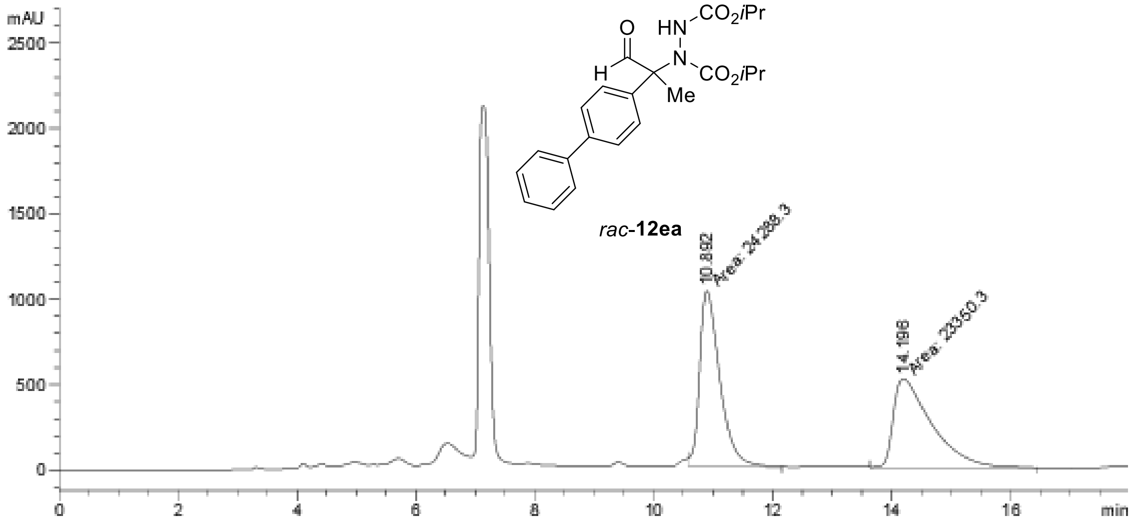


| Peak # | RetTime [min] | Type | Width [min] | Area [mAU*s] | Height [mAU] | Area % |
|---|---|---|---|---|---|---|
| 1 | 10.892 | MM | 0.3940 | 2.42883e4 | 1027.34741 | 50.9845 |
| 2 | 14.196 | MM | 0.7457 | 2.33503e4 | 521.87360 | 49.0155 |

**Figure S72.** HPLC chromatogram of compound *rac*-**12ea**.

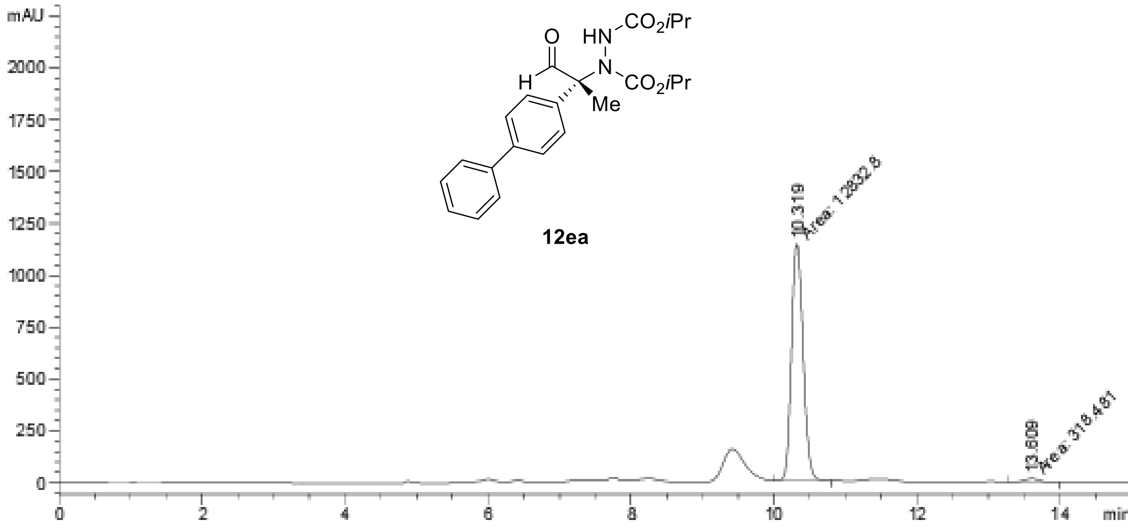


| Peak # | RetTime [min] | Type | Width [min] | Area [mAU*s] | Height [mAU] | Area % |
|---|---|---|---|---|---|---|
| 1 | 10.319 | MM | 0.1872 | 1.28328e4 | 1142.55042 | 97.5783 |
| 2 | 13.609 | MM | 0.2837 | 318.48068 | 18.71139 | 2.4217 |

**Figure S73.** HPLC chromatogram of compound **12ea**.

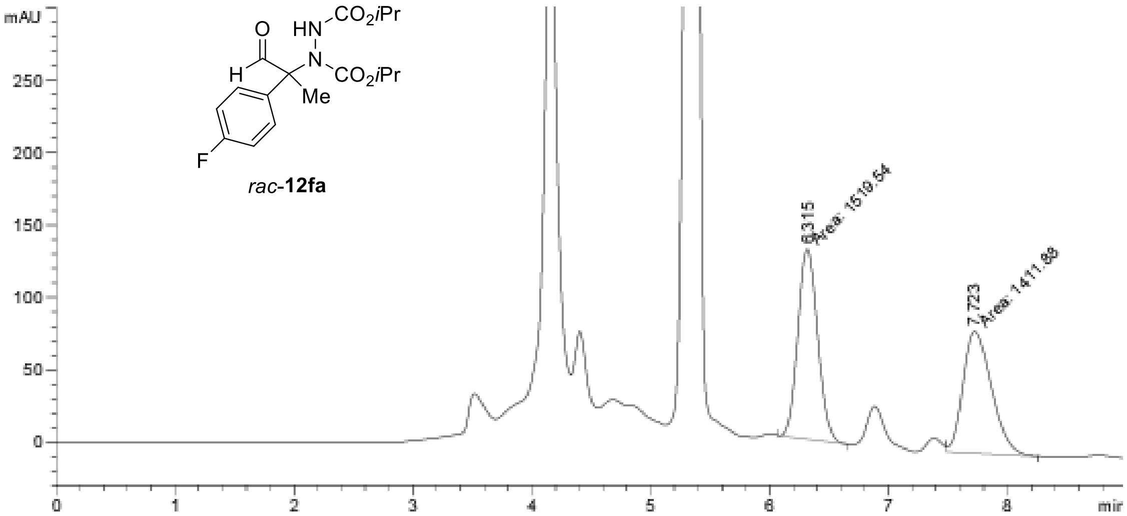


| Peak # | RetTime [min] | Type | Width [min] | Area [mAU*s] | Height [mAU] | Area % |
|---|---|---|---|---|---|---|
| 1 | 6.315 | MM | 0.1928 | 1519.53735 | 131.34573 | 51.8362 |
| 2 | 7.723 | MM | 0.2792 | 1411.88208 | 84.28263 | 48.1638 |

**Figure S74.** HPLC chromatogram of compound *rac*-**12fa**.

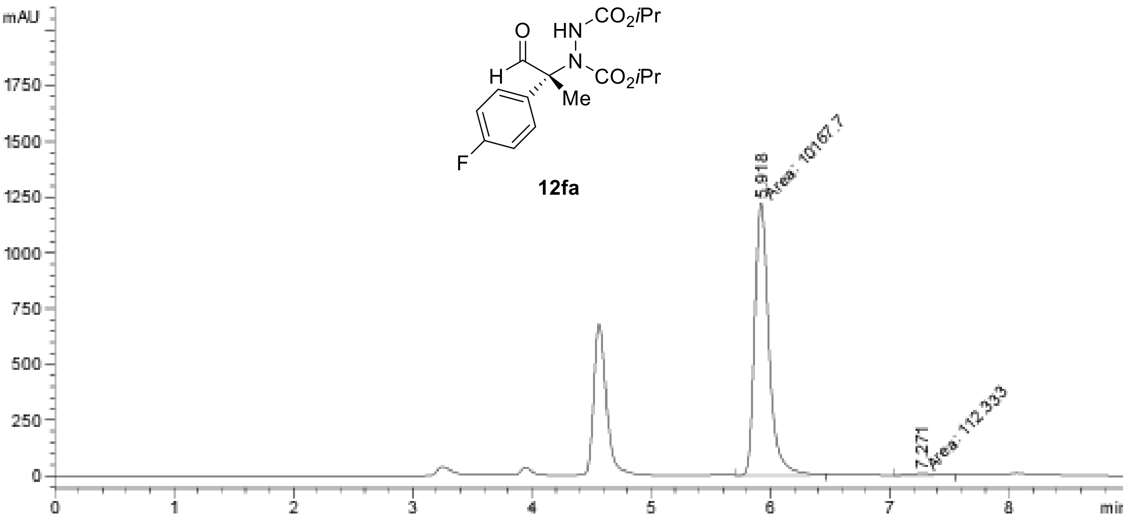


| Peak # | RetTime [min] | Type | Width [min] | Area [mAU*s] | Height [mAU] | Area % |
|---|---|---|---|---|---|---|
| 1 | 5.918 | MM | 0.1385 | 1.01677e4 | 1223.47705 | 98.9073 |
| 2 | 7.271 | MM | 0.2154 | 112.33310 | 8.69315 | 1.0927 |

**Figure S75.** HPLC chromatogram of compound **12fa**.

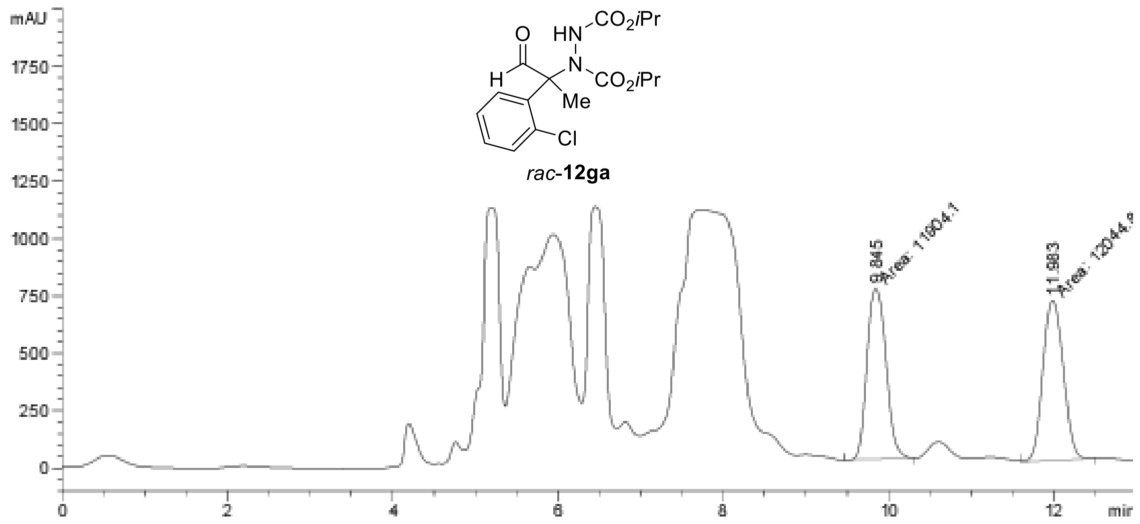


| Peak # | RetTime [min] | Type | Width [min] | Area [mAU*s] | Height [mAU] | Area % |
|---|---|---|---|---|---|---|
| 1 | 9.845 | MM | 0.2671 | 1.19041e4 | 742.86157 | 49.7062 |
| 2 | 11.983 | MM | 0.2875 | 1.20448e4 | 698.31116 | 50.2938 |

**Figure S76.** HPLC chromatogram of compound *rac*-**12ga**.

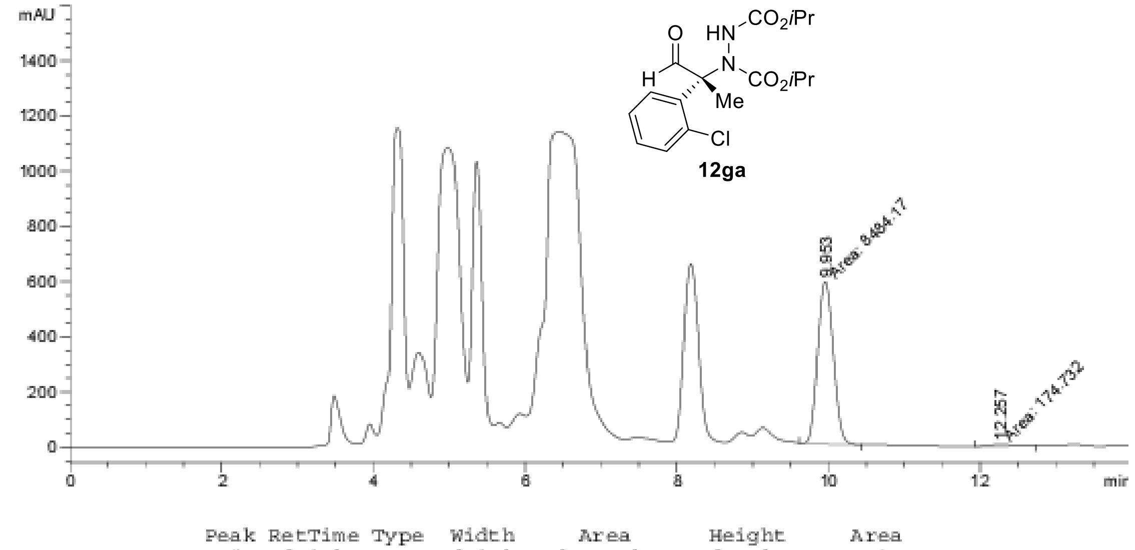


| Peak # | RetTime [min] | Type | Width [min] | Area [mAU*s] | Height [mAU] | Area % |
|---|---|---|---|---|---|---|
| 1 | 9.953 | MM | 0.2404 | 8484.17090 | 588.17108 | 97.9821 |
| 2 | 12.257 | MM | 0.4189 | 174.73192 | 6.95258 | 2.0179 |

**Figure S77.** HPLC chromatogram of compound **12ga**.

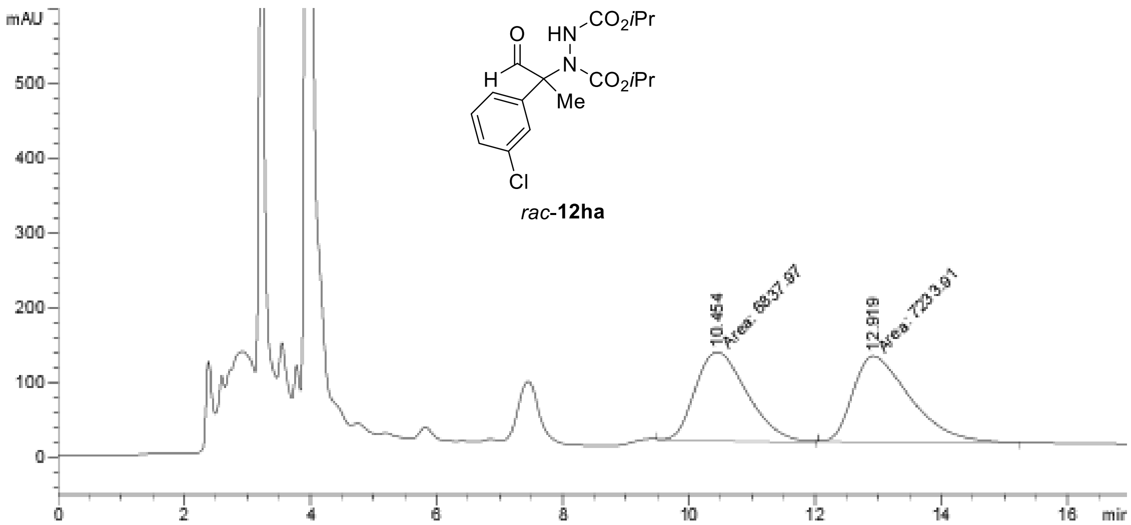


| Peak # | RetTime [min] | Type | Width [min] | Area [mAU*s] | Height [mAU] | Area % |
|---|---|---|---|---|---|---|
| 1 | 10.454 | MM | 0.9604 | 6837.96973 | 118.66920 | 48.5931 |
| 2 | 12.919 | MM | 1.0499 | 7233.91357 | 114.83564 | 51.4069 |

**Figure S78.** HPLC chromatogram of compound *rac*-**12ha**.

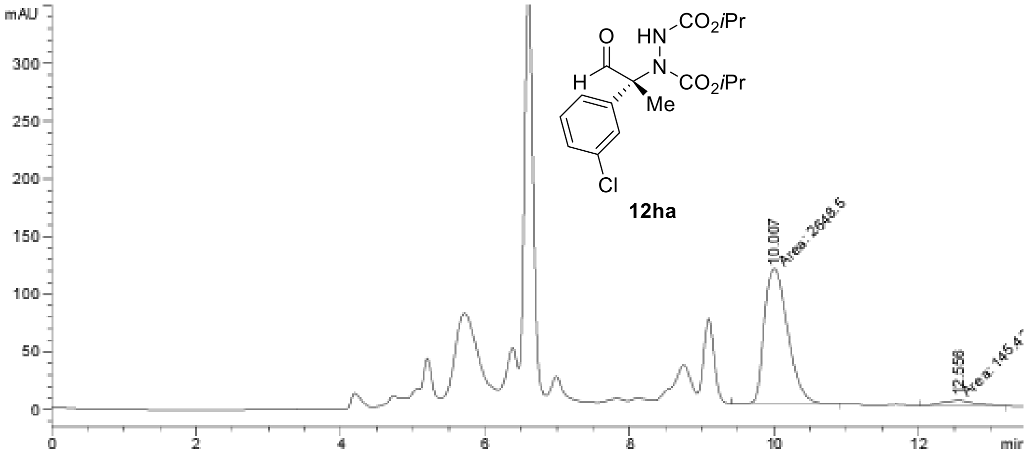


| Peak # | RetTime [min] | Type | Width [min] | Area [mAU*s] | Height [mAU] | Area % |
|---|---|---|---|---|---|---|
| 1 | 10.007 | MM | 0.3777 | 2648.50366 | 116.86100 | 94.7950 |
| 2 | 12.556 | MM | 0.5293 | 145.42538 | 4.57935 | 5.2050 |

**Figure S79.** HPLC chromatogram of compound **12ha**.

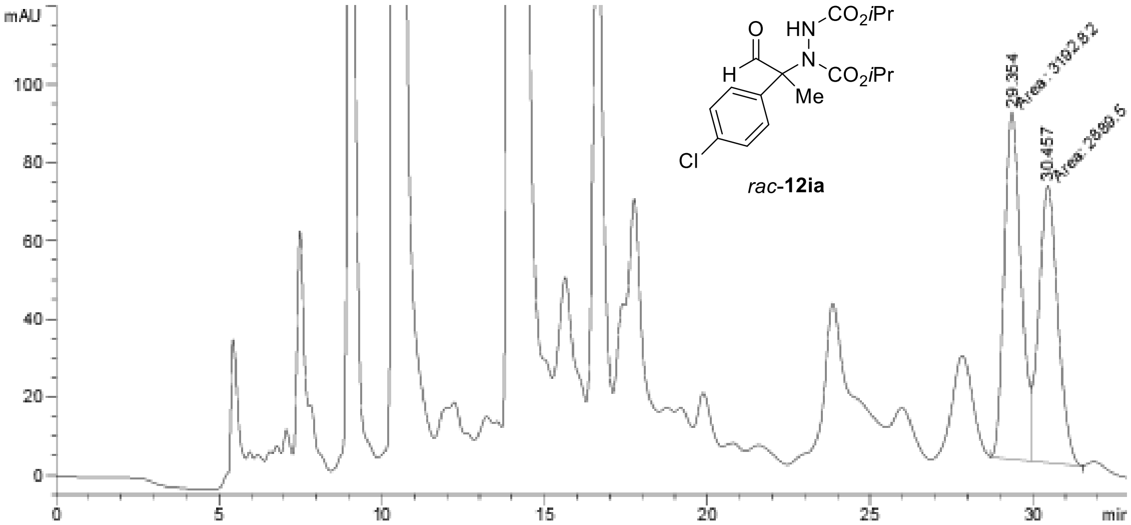


| Peak # | RetTime [min] | Type | Width [min] | Area [mAU*s] | Height [mAU] | Area % |
|---|---|---|---|---|---|---|
| 1 | 29.354 | MF | 0.5995 | 3192.82056 | 88.76774 | 52.4935 |
| 2 | 30.457 | FM | 0.6805 | 2889.49976 | 70.76472 | 47.5065 |

**Figure S80.** HPLC chromatogram of compound *rac*-**12ia**.

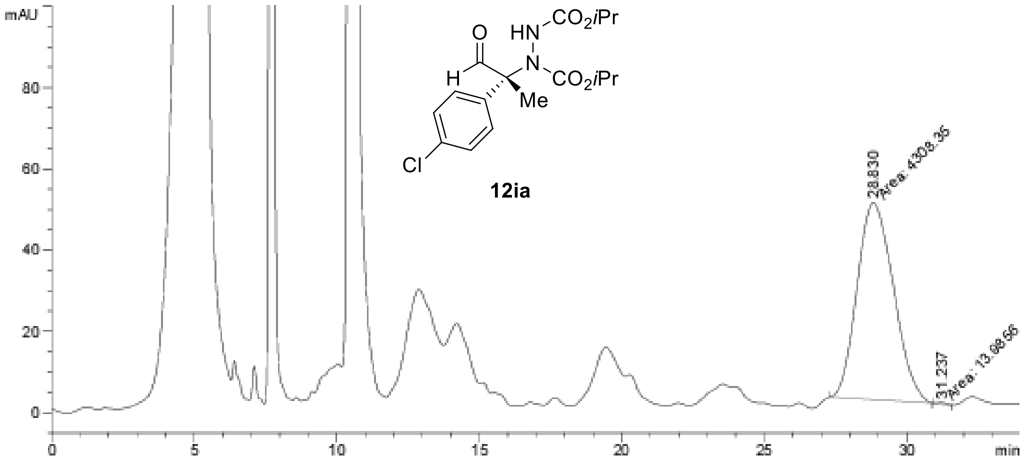


| Peak # | RetTime [min] | Type | Width [min] | Area [mAU*s] | Height [mAU] | Area % |
|---|---|---|---|---|---|---|
| 1 | 28.830 | MM | 1.4839 | 4308.35400 | 48.38976 | 99.6764 |
| 2 | 31.237 | MM | 0.4608 | 13.98563 | 5.05837e-1 | 0.3236 |

**Figure S81.** HPLC chromatogram of compound **12ia**.

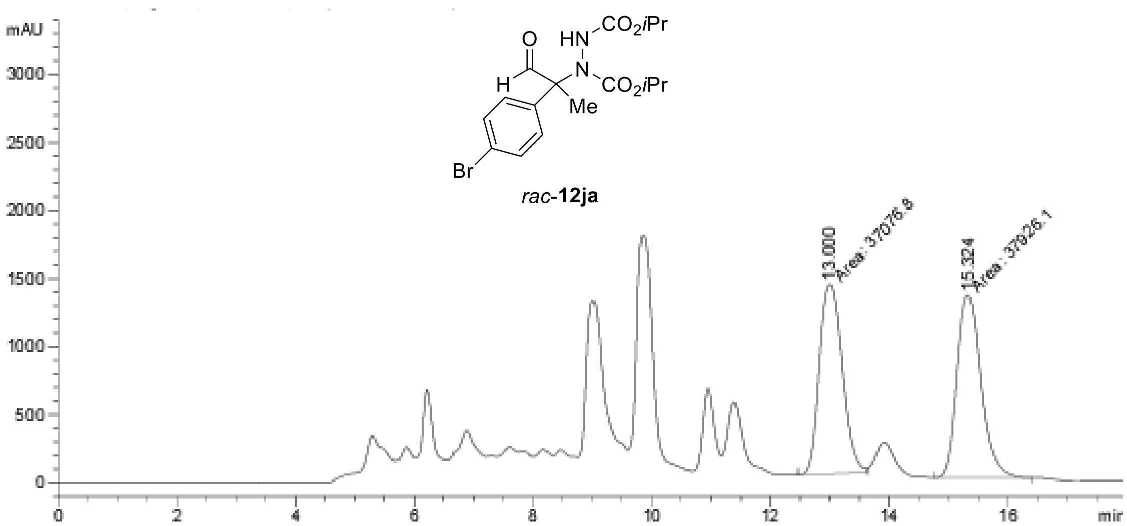


| Peak # | RetTime [min] | Type | Width [min] | Area [mAU*s] | Height [mAU] | Area % |
|---|---|---|---|---|---|---|
| 1 | 13.000 | MM | 0.4446 | 3.70768e4 | 1389.97766 | 49.4339 |
| 2 | 15.324 | MM | 0.4710 | 3.79261e4 | 1342.11658 | 50.5661 |

**Figure S82.** HPLC chromatogram of compound *rac*-**12ja**.

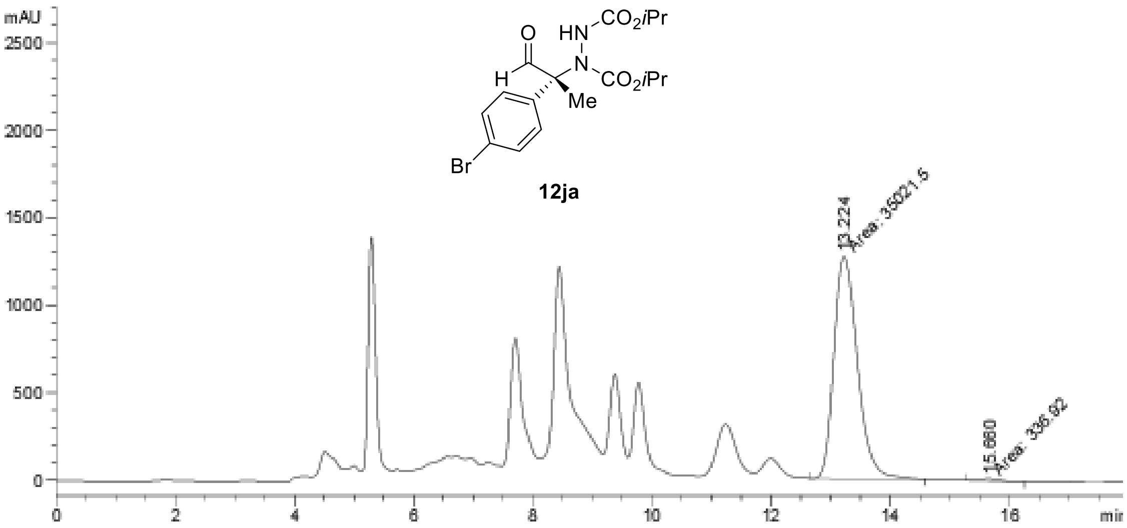


| Peak # | RetTime [min] | Type | Width [min] | Area [mAU*s] | Height [mAU] | Area % |
|---|---|---|---|---|---|---|
| 1 | 13.224 | MM | 0.4591 | 3.50215e4 | 1271.42664 | 99.0471 |
| 2 | 15.660 | MM | 0.4159 | 336.91998 | 13.50015 | 0.9529 |

**Figure S83.** HPLC chromatogram of compound **12ja**.

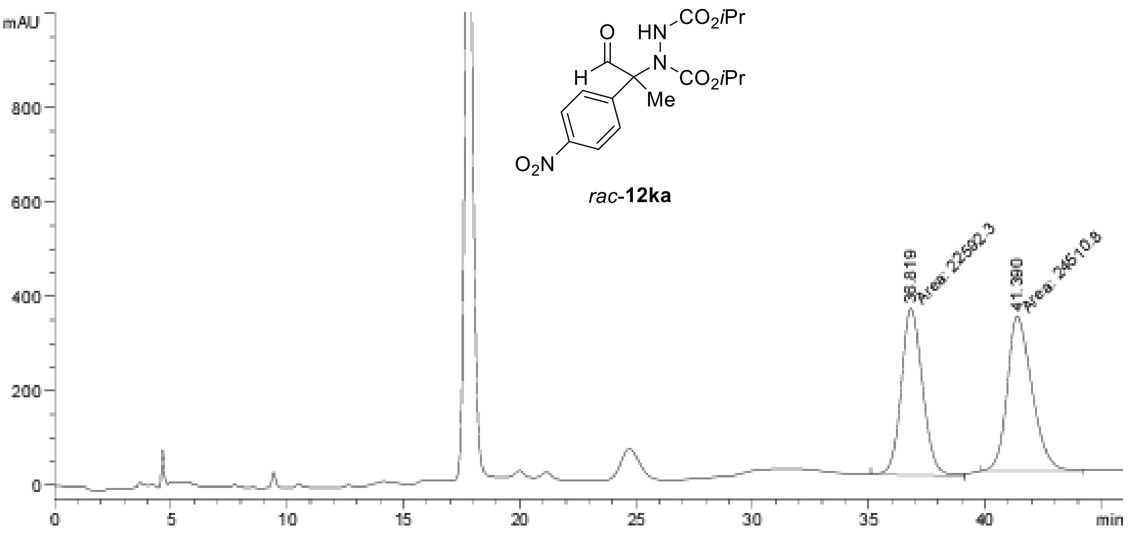


| Peak # | RetTime [min] | Type | Width [min] | Area [mAU*s] | Height [mAU] | Area % |
|---|---|---|---|---|---|---|
| 1 | 36.819 | MM | 1.0650 | 2.25922e4 | 353.54507 | 47.9635 |
| 2 | 41.390 | MM | 1.2456 | 2.45108e4 | 327.97543 | 52.0365 |

**Figure S84.** HPLC chromatogram of compound *rac*-**12ka**.

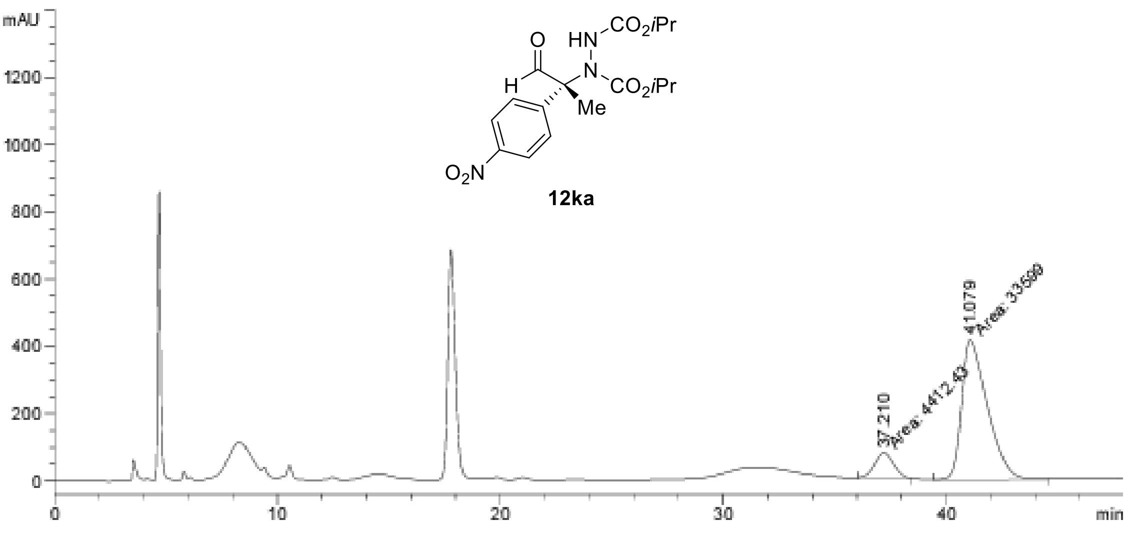


| Peak # | RetTime [min] | Type | Width [min] | Area [mAU*s] | Height [mAU] | Area % |
|---|---|---|---|---|---|---|
| 1 | 37.210 | MM | 0.9812 | 4412.43408 | 74.95010 | 11.6082 |
| 2 | 41.079 | MM | 1.3433 | 3.35990e4 | 416.87506 | 88.3918 |

**Figure S85.** HPLC chromatogram of compound **12ka**.

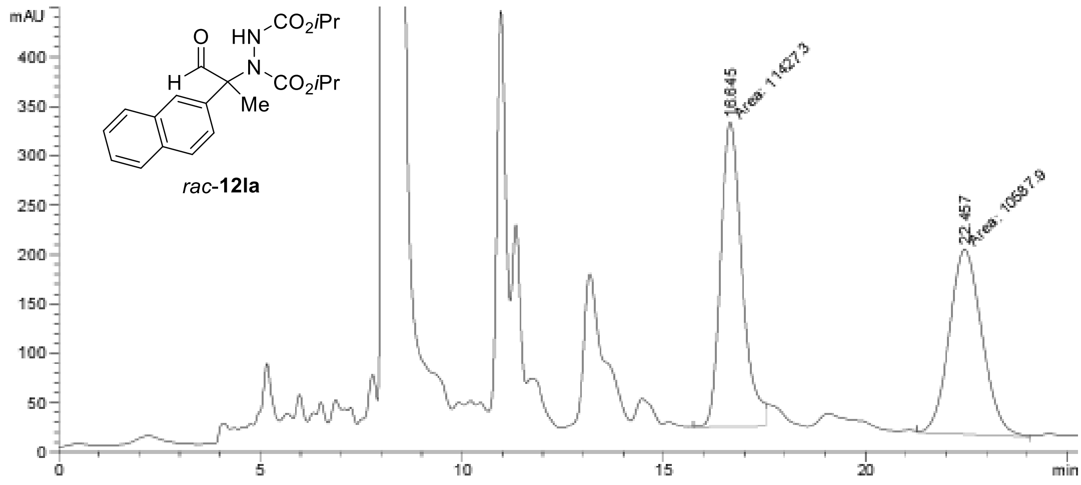

rac-**12la**

| Peak # | RetTime [min] | Type | Width [min] | Area [mAU*s] | Height [mAU] | Area % |
|---|---|---|---|---|---|---|
| 1 | 16.645 | MM | 0.6180 | 1.14273e4 | 308.16061 | 51.9065 |
| 2 | 22.457 | MM | 0.9406 | 1.05879e4 | 187.60022 | 48.0935 |

**Figure S86.** HPLC chromatogram of compound *rac*-**12la**.

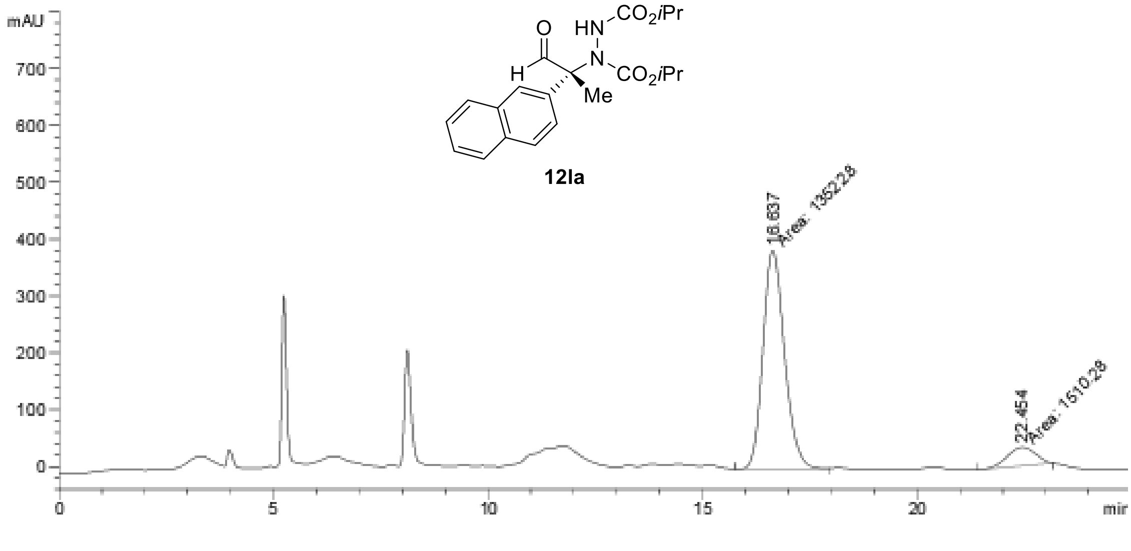

**12la**

| Peak # | RetTime [min] | Type | Width [min] | Area [mAU*s] | Height [mAU] | Area % |
|---|---|---|---|---|---|---|
| 1 | 16.637 | MM | 0.5853 | 1.35228e4 | 385.06741 | 89.9536 |
| 2 | 22.454 | MM | 0.7709 | 1510.27905 | 32.65319 | 10.0464 |

**Figure S87.** HPLC chromatogram of compound **12la**.

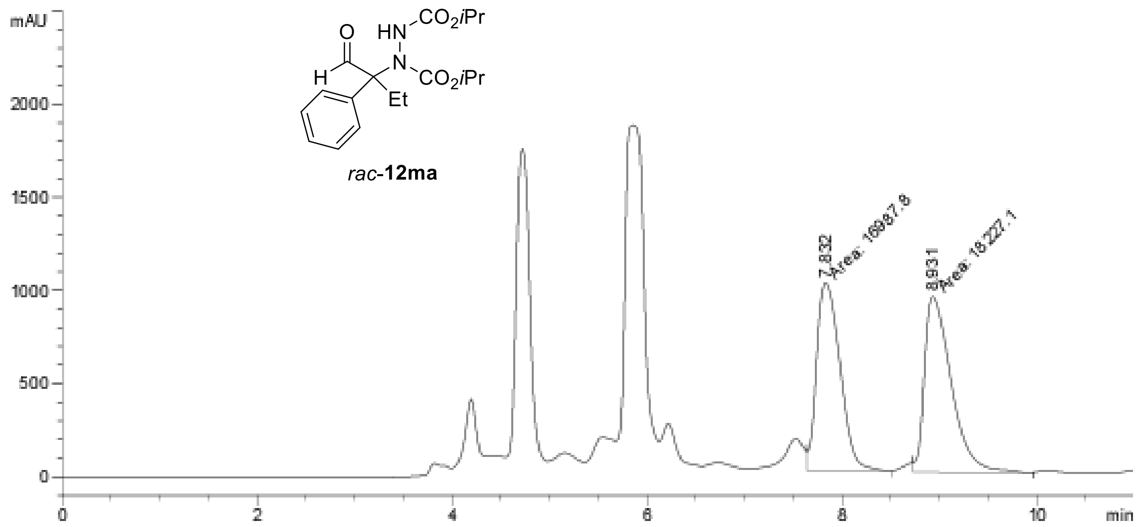


| Peak # | RetTime [min] | Type | Width [min] | Area [mAU*s] | Height [mAU] | Area % |
|---|---|---|---|---|---|---|
| 1 | 7.832 | MF | 0.2802 | 1.69878e4 | 1010.36151 | 48.2405 |
| 2 | 8.931 | FM | 0.3222 | 1.82271e4 | 942.85248 | 51.7595 |

**Figure S88.** HPLC chromatogram of compound *rac*-**12ma**.

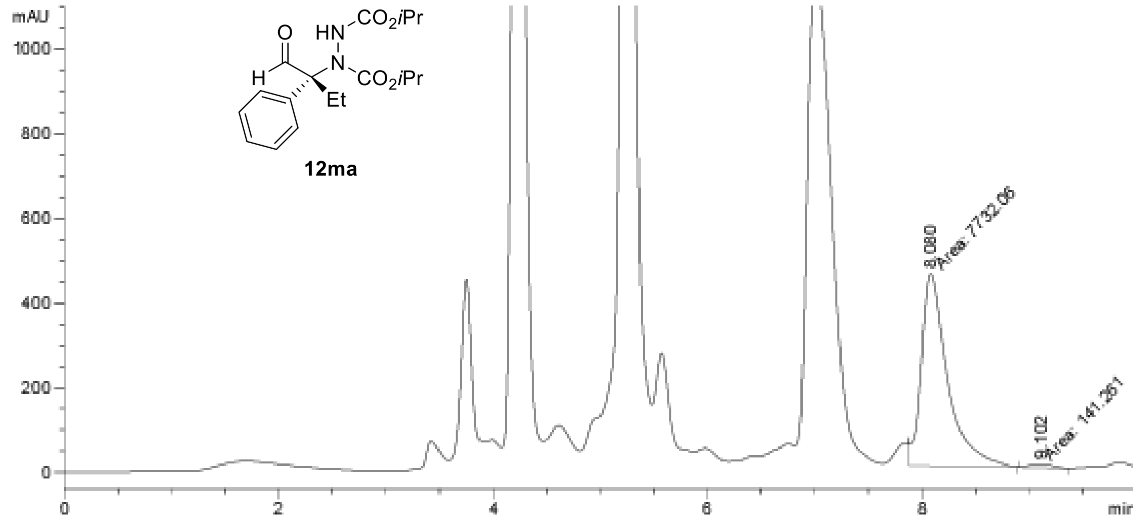


| Peak # | RetTime [min] | Type | Width [min] | Area [mAU*s] | Height [mAU] | Area % |
|---|---|---|---|---|---|---|
| 1 | 8.080 | MM | 0.2835 | 7732.05762 | 454.62033 | 98.2058 |
| 2 | 9.102 | MM | 0.2443 | 141.26071 | 9.63785 | 1.7942 |

**Figure S89.** HPLC chromatogram of compound **12ma**.

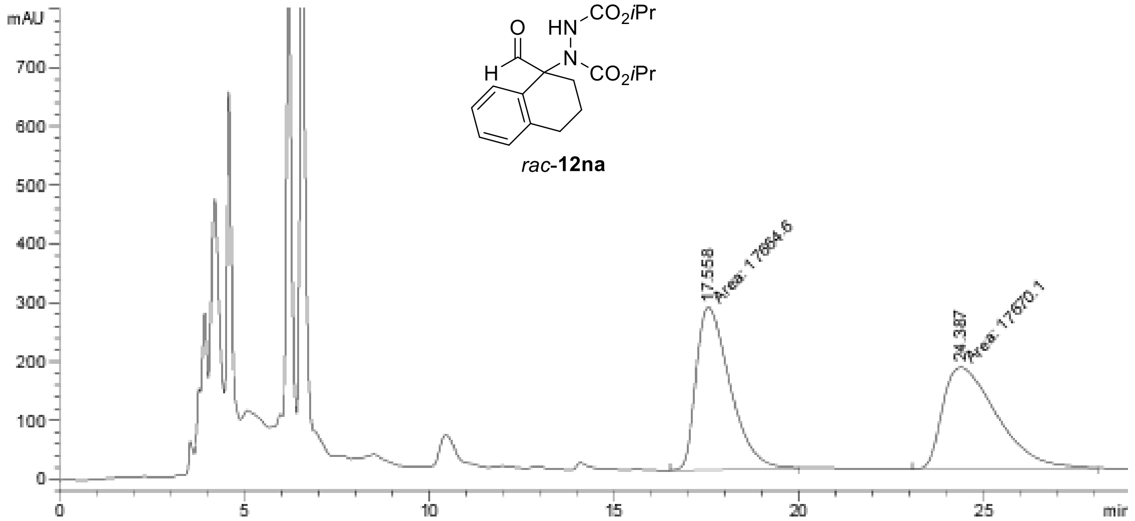


| Peak # | RetTime [min] | Type | Width [min] | Area [mAU*s] | Height [mAU] | Area % |
|---|---|---|---|---|---|---|
| 1 | 17.558 | MM | 1.0652 | 1.76646e4 | 276.38354 | 49.9923 |
| 2 | 24.387 | MM | 1.7061 | 1.76701e4 | 172.61725 | 50.0077 |

**Figure S90.** HPLC chromatogram of compound *rac*-**12na**.

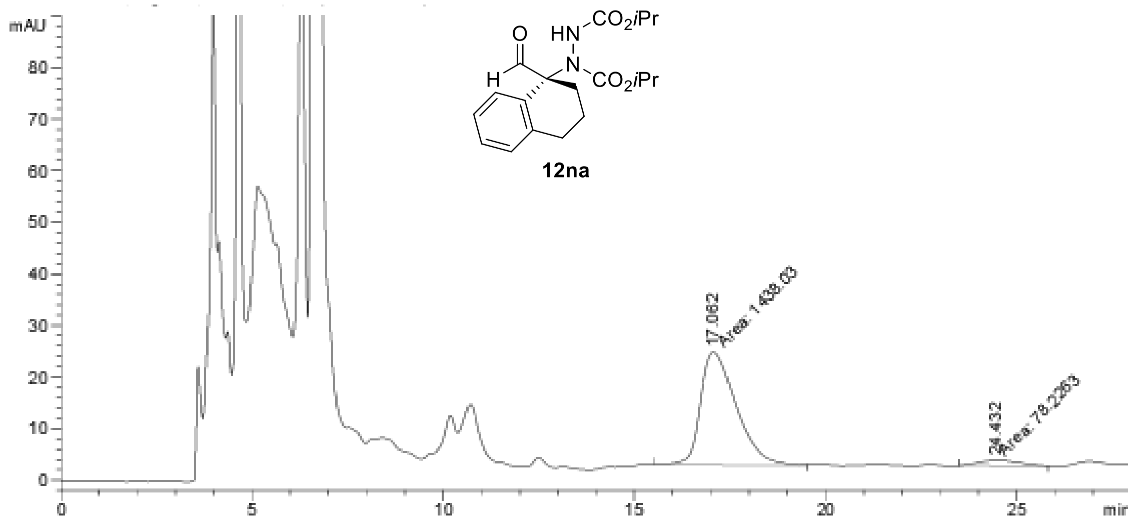


| Peak # | RetTime [min] | Type | Width [min] | Area [mAU*s] | Height [mAU] | Area % |
|---|---|---|---|---|---|---|
| 1 | 17.062 | MM | 1.0892 | 1438.03333 | 22.00508 | 94.8408 |
| 2 | 24.432 | MM | 1.1830 | 78.22626 | 1.10212 | 5.1592 |

**Figure S91.** HPLC chromatogram of compound **12na**.

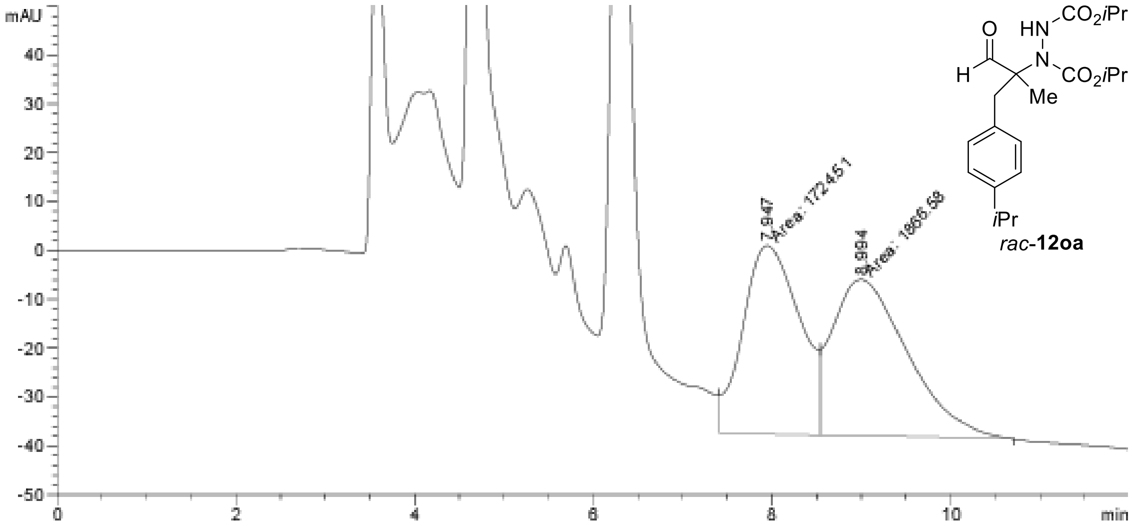


| Peak # | RetTime [min] | Type | Width [min] | Area [mAU*s] | Height [mAU] | Area % |
|---|---|---|---|---|---|---|
| 1 | 7.947 | MF | 0.7437 | 1724.51001 | 38.64548 | 48.0219 |
| 2 | 8.994 | FM | 0.9720 | 1866.58093 | 32.00672 | 51.9781 |

**Figure S92.** HPLC chromatogram of compound *rac*-**12oa**.

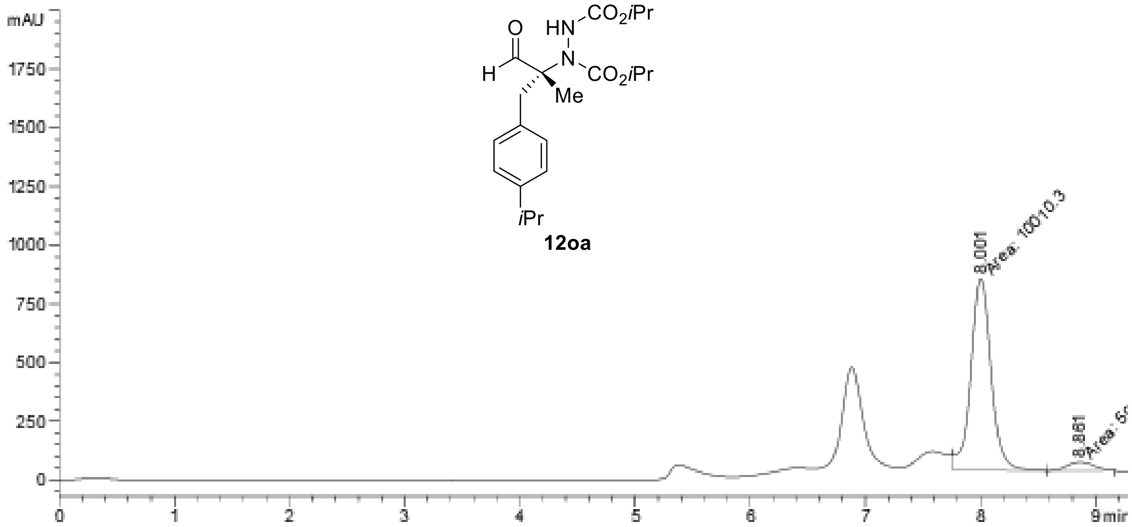


| Peak # | RetTime [min] | Type | Width [min] | Area [mAU*s] | Height [mAU] | Area % |
|---|---|---|---|---|---|---|
| 1 | 8.001 | MM | 0.2043 | 1.00103e4 | 816.51666 | 94.4158 |
| 2 | 8.861 | MM | 0.2717 | 592.05493 | 36.31840 | 5.5842 |

**Figure S93.** HPLC chromatogram of compound **12oa**.

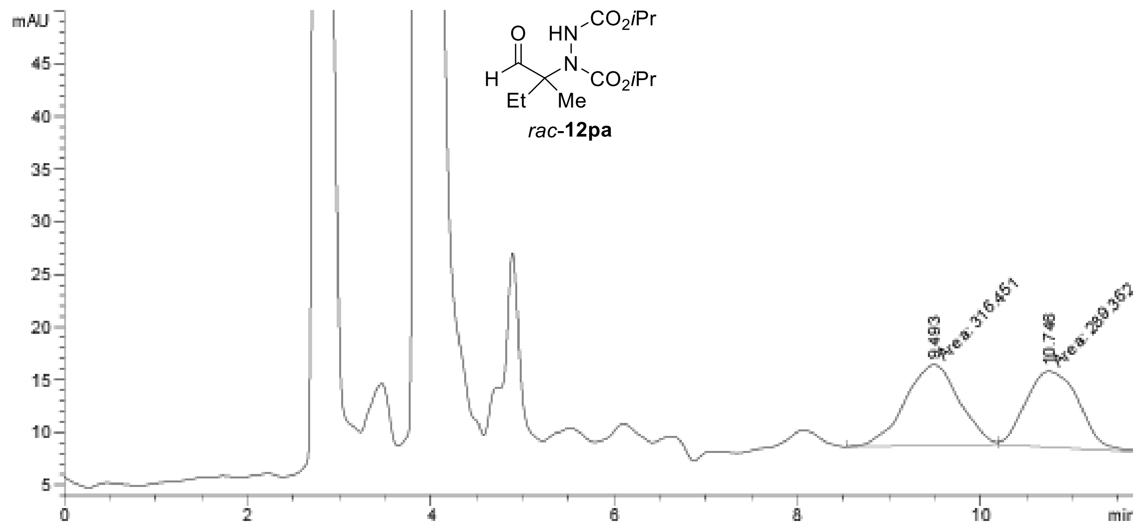


| Peak # | RetTime [min] | Type | Width [min] | Area [mAU*s] | Height [mAU] | Area % |
|---|---|---|---|---|---|---|
| 1 | 9.493 | MM | 0.6889 | 316.45108 | 7.65634 | 52.2358 |
| 2 | 10.746 | MM | 0.6698 | 289.36194 | 7.20048 | 47.7642 |

**Figure S94.** HPLC chromatogram of compound *rac*-**12pa**.

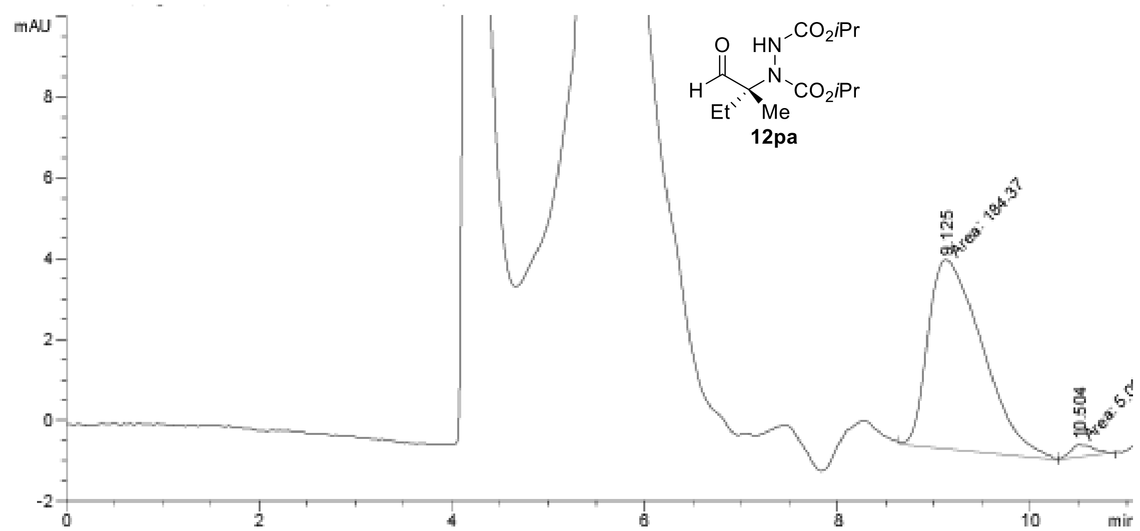


| Peak # | RetTime [min] | Type | Width [min] | Area [mAU*s] | Height [mAU] | Area % |
|---|---|---|---|---|---|---|
| 1 | 9.125 | MM | 0.6557 | 184.36987 | 4.68652 | 97.3279 |
| 2 | 10.504 | MM | 0.2829 | 5.06181 | 2.98217e-1 | 2.6721 |

**Figure S95.** HPLC chromatogram of compound **12pa**.

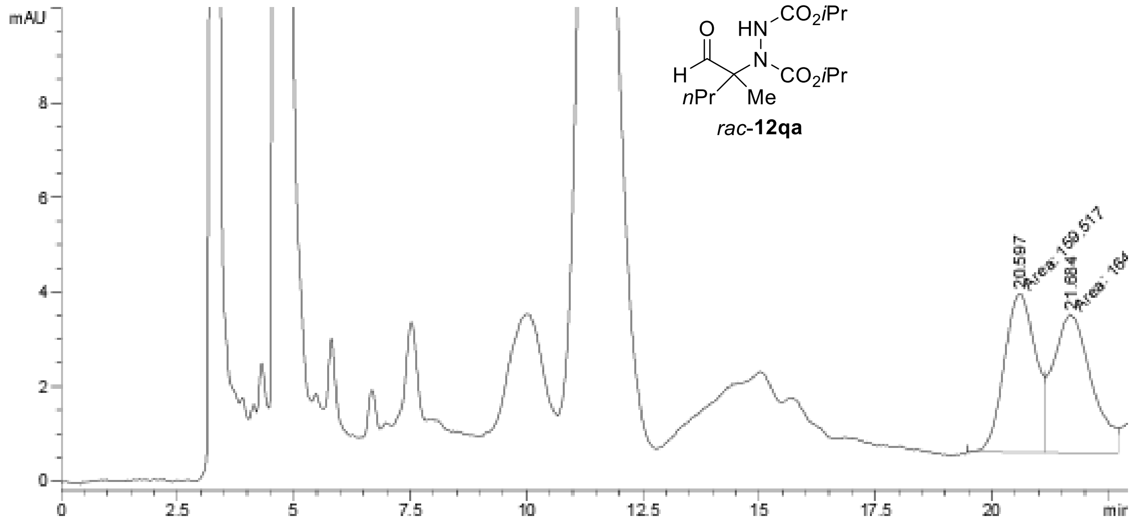


| Peak # | RetTime [min] | Type | Width [min] | Area [mAU*s] | Height [mAU] | Area % |
|---|---|---|---|---|---|---|
| 1 | 20.597 | MF | 0.7940 | 159.51707 | 3.34856 | 49.1774 |
| 2 | 21.684 | FM | 0.9395 | 164.85352 | 2.92448 | 50.8226 |

**Figure S96.** HPLC chromatogram of compound *rac*-**12qa**.

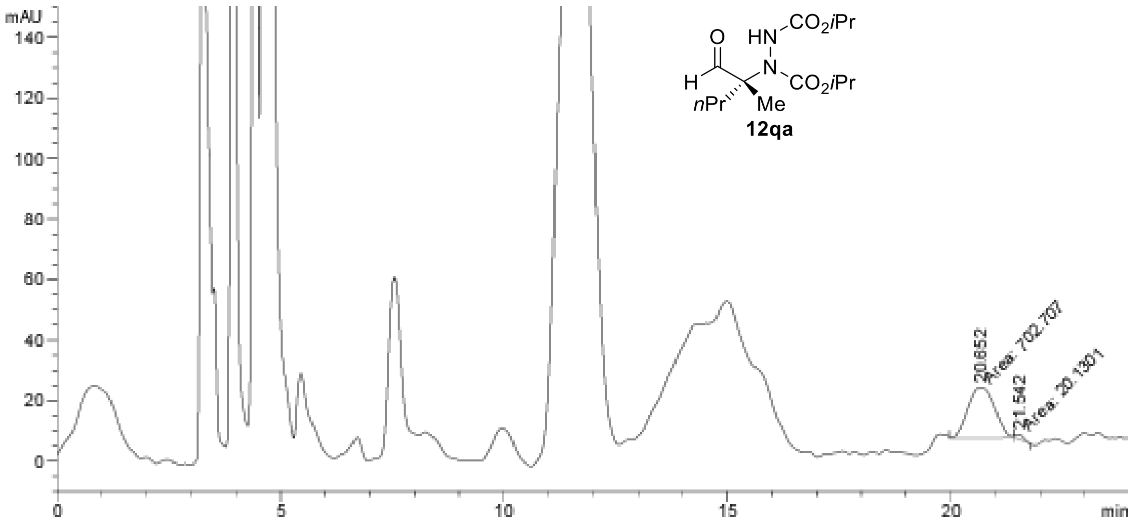


| Peak # | RetTime [min] | Type | Width [min] | Area [mAU*s] | Height [mAU] | Area % |
|---|---|---|---|---|---|---|
| 1 | 20.652 | MM | 0.7025 | 702.70715 | 16.67043 | 97.2151 |
| 2 | 21.542 | MM | 0.2056 | 20.13012 | 1.63219 | 2.7849 |

**Figure S97.** HPLC chromatogram of compound **12qa**.

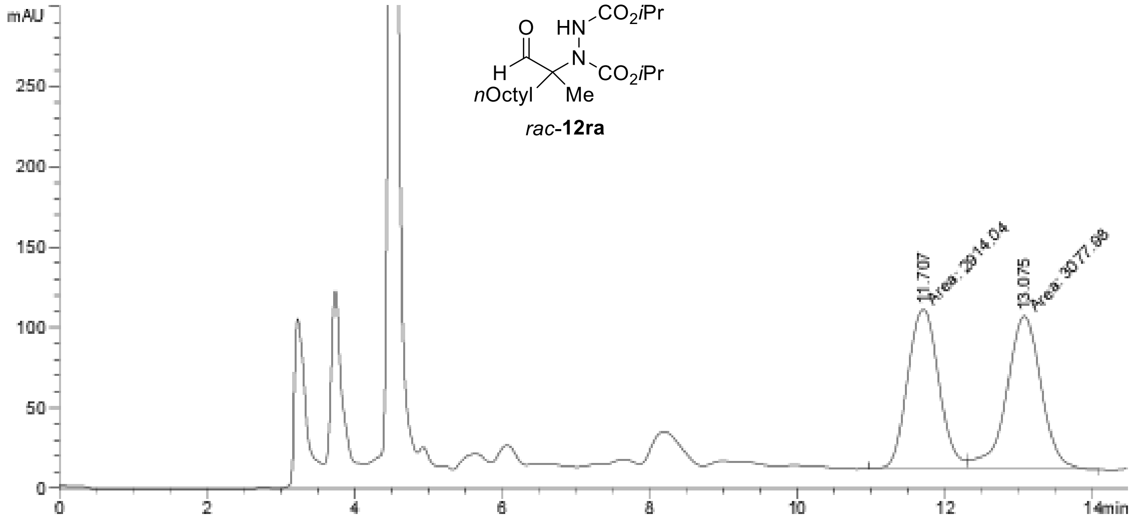


| Peak # | RetTime [min] | Type | Width [min] | Area [mAU*s] | Height [mAU] | Area % |
|---|---|---|---|---|---|---|
| 1 | 11.707 | MF | 0.4917 | 2914.04175 | 98.77592 | 48.6321 |
| 2 | 13.075 | FM | 0.5401 | 3077.97705 | 94.98752 | 51.3679 |

**Figure S98.** HPLC chromatogram of compound *rac*-**12ra**.

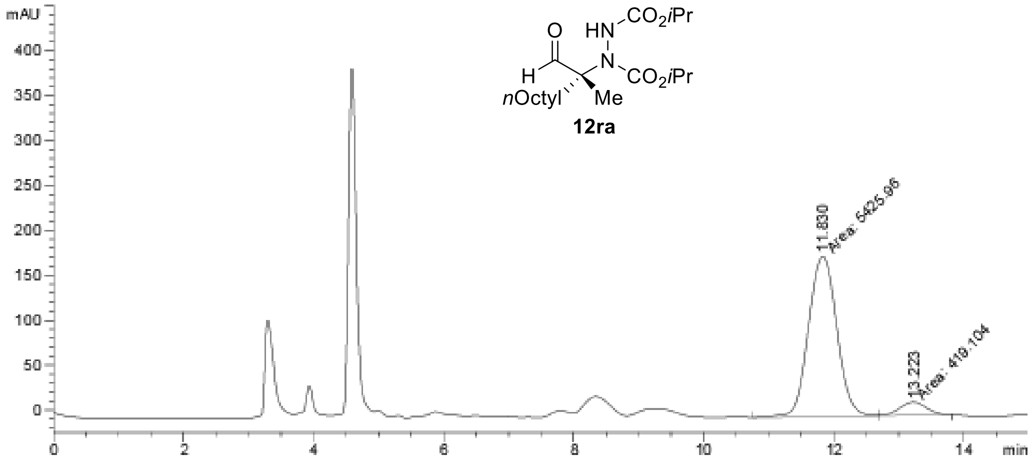


| Peak # | RetTime [min] | Type | Width [min] | Area [mAU*s] | Height [mAU] | Area % |
|---|---|---|---|---|---|---|
| 1 | 11.830 | MM | 0.5063 | 5425.95850 | 178.60724 | 92.8298 |
| 2 | 13.223 | MM | 0.4764 | 419.10379 | 14.66179 | 7.1702 |

**Figure S99.** HPLC chromatogram of compound **12ra**.

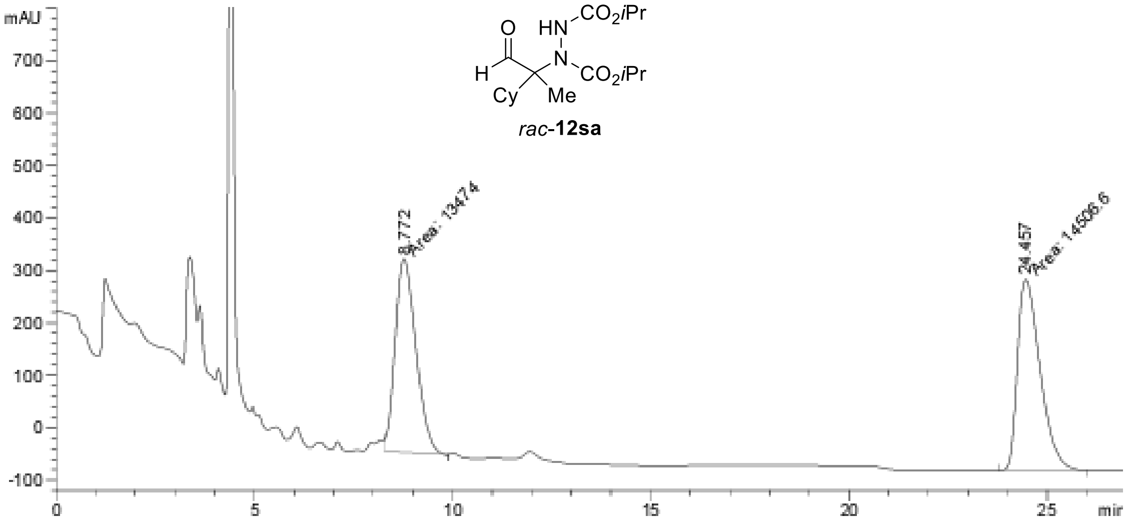


| Peak # | RetTime [min] | Type | Width [min] | Area [mAU*s] | Height [mAU] | Area % |
|---|---|---|---|---|---|---|
| 1 | 8.772 | FM | 0.6084 | 1.34740e4 | 369.12384 | 48.1547 |
| 2 | 24.457 | MM | 0.6638 | 1.45066e4 | 364.25745 | 51.8453 |

**Figure S100.** HPLC chromatogram of compound *rac*-**12sa**.

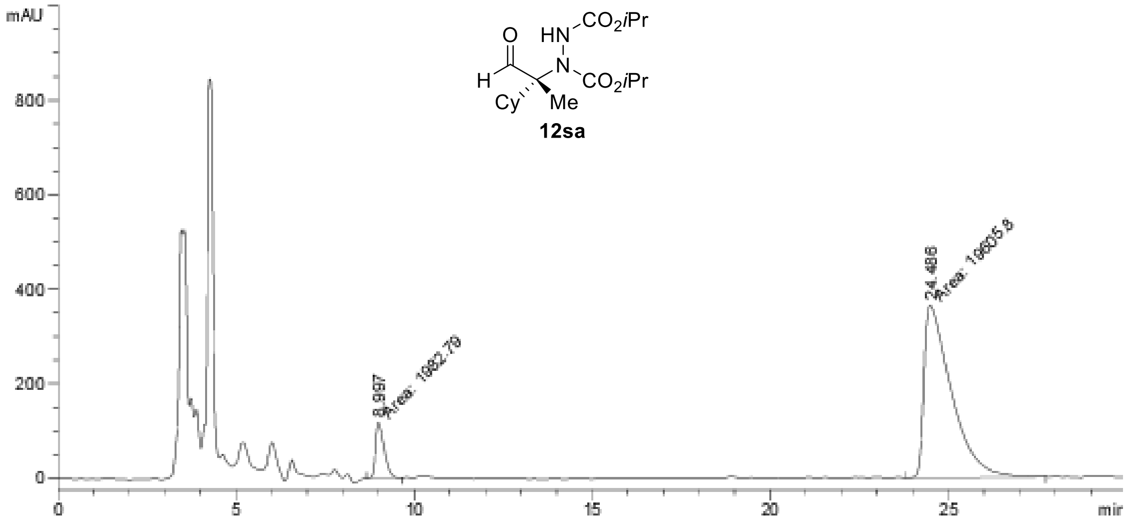


| Peak # | RetTime [min] | Type | Width [min] | Area [mAU*s] | Height [mAU] | Area % |
|---|---|---|---|---|---|---|
| 1 | 8.997 | MM | 0.2796 | 1982.79443 | 118.19604 | 9.1845 |
| 2 | 24.486 | MM | 0.8931 | 1.96058e4 | 365.88602 | 90.8155 |

**Figure S101.** HPLC chromatogram of compound **12sa**.

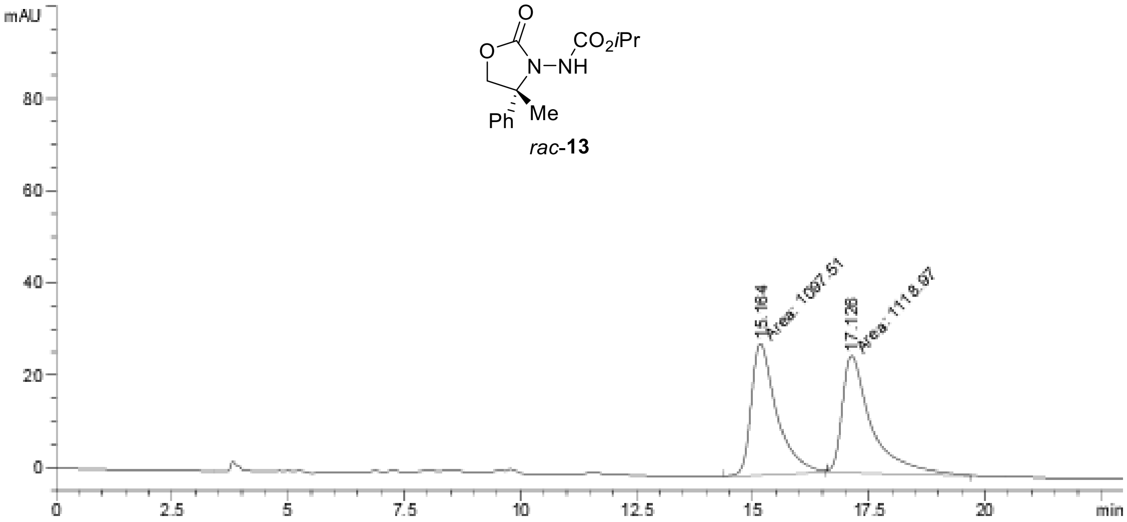


| Peak # | RetTime [min] | Type | Width [min] | Area [mAU*s] | Height [mAU] | Area % |
|---|---|---|---|---|---|---|
| 1 | 15.164 | MM | 0.6422 | 1097.50879 | 28.48422 | 49.5159 |
| 2 | 17.126 | MM | 0.7347 | 1118.96973 | 25.38483 | 50.4841 |

**Figure S102.** HPLC chromatogram of compound *rac*-**13**.

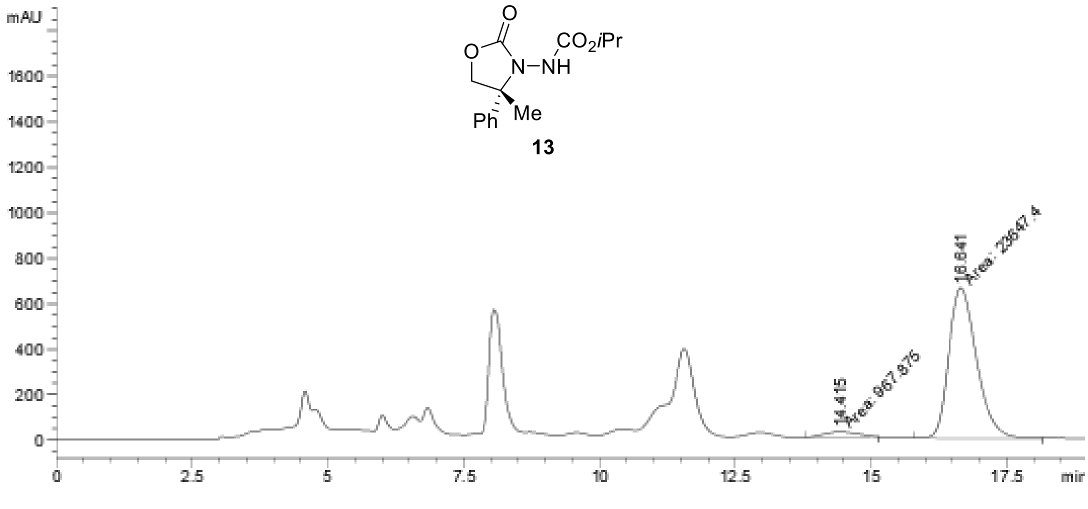


| Peak # | RetTime [min] | Type | Width [min] | Area [mAU*s] | Height [mAU] | Area % |
|---|---|---|---|---|---|---|
| 1 | 14.415 | MM | 0.7146 | 967.87537 | 22.57509 | 3.9320 |
| 2 | 16.641 | MM | 0.5946 | 2.36474e4 | 662.82599 | 96.0680 |

**Figure S103.** HPLC chromatogram of compound **13**.

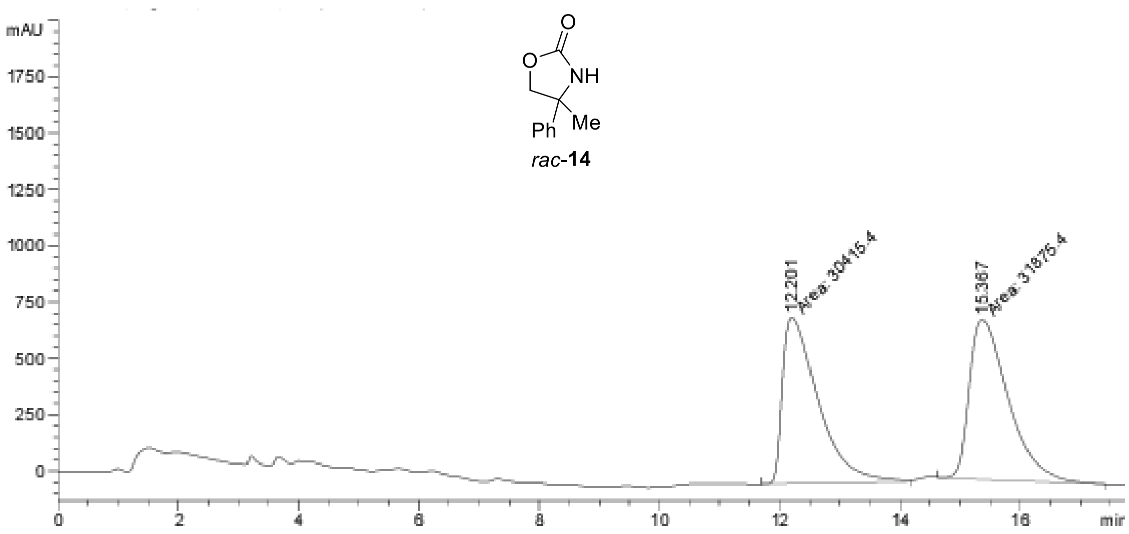


| Peak # | RetTime [min] | Type | Width [min] | Area [mAU*s] | Height [mAU] | Area % |
|---|---|---|---|---|---|---|
| 1 | 12.201 | MM | 0.6898 | 3.04154e4 | 734.85291 | 48.8281 |
| 2 | 15.367 | MM | 0.7508 | 3.18754e4 | 707.59320 | 51.1719 |

**Figure S104.** HPLC chromatogram of compound *rac*-**14**.

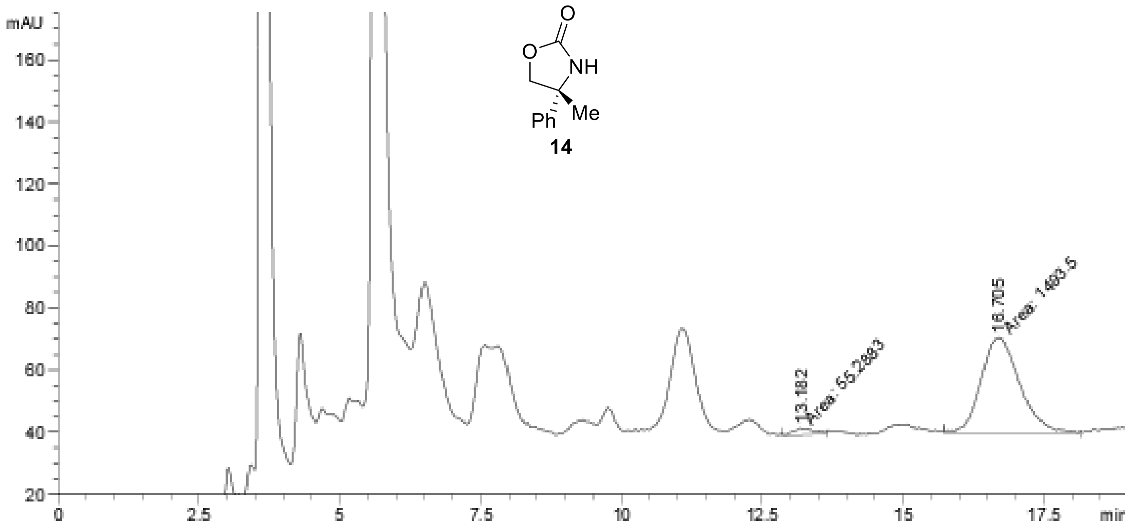


| Peak # | RetTime [min] | Type | Width [min] | Area [mAU*s] | Height [mAU] | Area % |
|---|---|---|---|---|---|---|
| 1 | 13.182 | MM | 0.4139 | 55.28831 | 2.22615 | 3.5698 |
| 2 | 16.705 | MM | 0.8064 | 1493.50476 | 30.86742 | 96.4302 |

**Figure S105.** HPLC chromatogram of compound **14**.

## VIII. Green chemistry metrics

**General remarks**. The E-factors for the each organocatalytic enantioselective α-nitrogenation of aldehydes were calculated using the equation 1.[7] In the term "Total imput mass" are included the mass of reagents and catalysts used, and the mass of silica and eluent employed in the purification by column chromatography. The E-factors for the reaction methodology excluding all the contributions coming from the purification were also calculated using the same equation.

$$\text{E-factor} = \frac{\textit{Total imput mass} - \textit{Mass of product}}{\textit{Mass of product}} \qquad (1)$$

EcoScale values for each organocatalytic enantioselective α-nitrogenation of aldehydes were calculated following the literature method,[8] starting with a value of 100 and subtracting to that value different penalty points assigned to the following six reaction parameters: 1. Yield; 2. Price of reaction components; 3. Safety; 4. Technical setup; 5. Temperature/time; 6. Workup and purification. The reaction conditions are ranked using the following scores: >75, excellent; >50, acceptable; <50 inadequate.

### E-Factor and EcoScale values

**Table S1.** E-Factor and EcoScale values for the different organocatalytic enantioselective α-nitrogenations of aldehydes and comparison with literature.

| Product | E-Factor (no purif.)[a] | E-Factor (purif.)[a] | EcoScale[a] | EcoScale (literature)[b] |
|---|---|---|---|---|
| **12aa** | 0.30 | 291 | 70 | 54,[6] 61,[4] 68,[9] 58,[10] 56,[11] 59[12] |
| **12ab** | 0.30 | 271 | 67 | 24,[6] 66,[9] 54,[10] 42,[11] 59,[12] 65[13] |
| **12ba** | 0.34 | 291 | 68 | – |
| **12ca** | 0.30 | 282 | 70 | 59,[6] 61[4] |
| **12da** | 0.27 | 266 | 70 | – |
| **12ea** | 0.28 | 244 | 69 | – |
| **12fa** | 0.37 | 294 | 67 | 45,[6] 65[4] |
| **12ga** | 0.35 | 280 | 67 | 22,[6] 27[4] |
| **12ha** | 0.38 | 287 | 66 | 49,[6] 63[4] |
| **12ia** | 0.40 | 290 | 66 | – |
| **12ja** | 0.29 | 245 | 68 | 52,[6] 64[4] |
| **12ka** | 0.47 | 298 | 63 | 59,[4] |
| **12la** | 0.33 | 266 | 68 | 56,[6] 63[4] |
| **12ma** | 0.29 | 279 | 70 | – |
| **12na** | 0.36 | 287 | 67 | – |
| **12oa** | 0.29 | 256 | 72 | – |
| **12pa** | 0.37 | 346 | 67 | – |
| **12qa** | 0.34 | 326 | 63 | 57,[6] 39[4] |
| **12ra** | 0.29 | 268 | 68 | – |
| **12sa** | 0.33 | 295 | 67 | – |

[a] Obtained from the experimental data reported in the Supporting Information (see Supporting Information for details in calculations). [b] Obtained from the experimental data reported in the respective publications (see Supporting Information for details in calculations).

### Calculations details and literature comparison of EcoScale values.

<u>– Compound **12aa**</u>

$$\text{E-factor (no purif.)} = \frac{0.0863 - 0.0665}{0.0665} = 0.30 \qquad \text{E-factor (purif.)} = \frac{19.3875 - 0.0665}{0.0665} = 291$$

EcoScale = 100 – total penalty points = 70 (acceptable)

| Reaction parameters | Penalty points |
|---|---|
| 1. Yield: 99% | 1 |
| 2. **9**: Very expensive<br>**10a**: Expensive<br>**11a**: Inexpensive<br>Acetic acid: Inexpensive | 5<br>3<br>0<br>0 |
| 3. **9**: –<br>**10a**: –<br>**11a**: Dangerous for the environment<br>Acetic acid: – | 0<br>0<br>5<br>0 |
| 4. Common setup<br>Inert gas atmosphere | 0<br>1 |
| 5. Cooling < 0 ºC | 5 |
| 6. Classical chromatography | 10 |
| Total penalty points: | 30 |

Literature: *J. Org. Chem.* **2011**, *76*, 4661-4664

EcoScale = 100 – total penalty points = 54 (acceptable)

| Reaction parameters | Penalty points |
|---|---|
| 1. Yield: 82% | 9 |
| 2. Catalyst: Very expensive<br>**10a**: Very expensive<br>**11a**: Inexpensive<br>Tetrahydrofuran: Expensive | 5<br>5<br>0<br>3 |
| 3. Catalyst: –<br>**10a**: –<br>**11a**: Dangerous for the environment<br>Tetrahydrofuran: Highly flammable | 0<br>0<br>5<br>5 |
| 4. Common setup | 0 |
| 5. Cooling to 0 ºC | 4 |
| 6. Classical chromatography | 10 |
| Total penalty points: | 46 |

Literature: *Org. Biomol. Chem.* **2010**, *8*, 4524-4526

EcoScale = 100 – total penalty points = 61 (acceptable)

| Reaction parameters | Penalty points |
|---|---|
| 1. Yield: 87% | 7 |
| 2. Catalyst: Very expensive<br>**10a**: Very expensive<br>**11a**: Inexpensive<br>Salicylic acid: Inexpensive<br>Dichloromethane: Expensive | 5<br>5<br>0<br>0<br>3 |
| 3. Catalyst: –<br>**10a**: –<br>**11a**: Dangerous for the environment<br>Salicylic acid: –<br>Dichloromethane: – | 0<br>0<br>5<br>0<br>0 |
| 4. Common setup | 0 |
| 5. Cooling to 0 ºC | 4 |
| 6. Classical chromatography | 10 |
| Total penalty points: | 39 |

Literature: *Tetrahedron Lett.* **2011**, *52*, 4430-4432

EcoScale = 100 – total penalty points = 68 (acceptable)

| Reaction parameters | Penalty points |
|---|---|
| 1. Yield: 95% | 3 |
| 2. Catalyst: Very expensive<br>**10a**: Expensive<br>**11a**: Inexpensive<br>Trifluoroacetic acid: Inexpensive<br>Chloroform: Very expensive | 5<br>3<br>0<br>0<br>5 |
| 3. Catalyst: –<br>**10a**: –<br>**11a**: Dangerous for the environment<br>Trifluoroacetic acid: –<br>Chloroform: – | 0<br>0<br>5<br>0<br>0 |
| 4. Common setup | 0 |
| 5. Room temperature <24 h | 1 |
| 6. Classical chromatography | 10 |
| Total penalty points: | 32 |

Literature: *Chirality* **2013**, *25*, 668-672

EcoScale = 100 – total penalty points = 58 (acceptable)

| Reaction parameters | Penalty points |
|---|---|
| 1. Yield: 94% | 3 |
| 2. Catalyst: Very expensive<br>**10a**: Very expensive<br>**11a**: Inexpensive<br>Trifluoroacetic acid: Inexpensive<br>1,2-Dichloroethane: Expensive | 5<br>5<br>0<br>0<br>3 |
| 3. Catalyst: –<br>**10a**: –<br>**11a**: Dangerous for the environment<br>Trifluoroacetic acid: –<br>1,2-Dichloroethane: Highly flammable and toxic | 0<br>0<br>5<br>0<br>10 |
| 4. Common setup | 0 |
| 5. Room temperature <24 h | 1 |
| 6. Classical chromatography | 10 |
| Total penalty points: | 42 |

Literature: *Eur. J. Org. Chem.* **2013**, *2013*, 2864-2868

EcoScale = 100 – total penalty points = 56 (acceptable)

| Reaction parameters | Penalty points |
|---|---|
| 1. Yield: 92% | 4 |
| 2. Catalyst: Very expensive<br>**10a**: Very expensive<br>**11a**: Inexpensive<br>Trifluoroacetic acid: Inexpensive<br>4-Chloro-2-nitrobenzoic acid: Inexpensive<br>Diethyl ether: Inexpensive | 5<br>5<br>0<br>0<br>0<br>0 |
| 3. Catalyst: –<br>**10a**: –<br>**11a**: Dangerous for the environment<br>Trifluoroacetic acid: –<br>4-Chloro-2-nitrobenzoic acid: –<br>Diethyl ether: Extremely flammable | 0<br>0<br>5<br>0<br>0<br>10 |
| 4. Common setup | 0 |
| 5. Cooling < 0 ºC | 5 |
| 6. Classical chromatography | 10 |
| Total penalty points: | 44 |

Literature: *Tetrahedron* **2013**, *69*, 5438-5443

EcoScale = 100 – total penalty points = 59 (acceptable)

| Reaction parameters | Penalty points |
|---|---|
| 1. Yield: 93% | 4 |
| 2. Catalyst: Very expensive<br>**10a**: Very expensive<br>**11a**: Inexpensive<br>Tetrahydrofuran: Expensive | 5<br>5<br>0<br>3 |
| 3. Catalyst: –<br>**10a**: –<br>**11a**: Dangerous for the environment<br>Tetrahydrofuran: Highly flammable | 0<br>0<br>5<br>5 |
| 4. Common setup | 0 |
| 5. Cooling to 0 ºC | 4 |
| 6. Classical chromatography | 10 |
| Total penalty points: | 41 |

<u>– Compound **12ab**</u>

$$\text{E-factor (no purif.)} = \frac{0.0929 - 0.0713}{0.0713} = 0.30 \qquad \text{E-factor (purif.)} = \frac{19.3942 - 0.0713}{0.0713} = 271$$

EcoScale = 100 – total penalty points = 67 (acceptable)

| Reaction parameters | Penalty points |
|---|---|
| 1. Yield: 98% | 1 |
| 2. **9**: Very expensive<br>**10a**: Expensive<br>**11b**: Expensive<br>Acetic acid: Inexpensive | 5<br>3<br>3<br>0 |
| 3. **9**: –<br>**10a**: –<br>**11b**: Higlhy flammable<br>Acetic acid: – | 0<br>0<br>5<br>0 |
| 4. Common setup<br>Inert gas atmosphere | 0<br>1 |
| 5. Cooling < 0 ºC | 5 |
| 6. Classical chromatography | 10 |
| Total penalty points: | 33 |

Literature: *J. Org. Chem.* **2011**, *76*, 4661-4664

EcoScale = 100 – total penalty points = 24 (inadequate)

| Reaction parameters | Penalty points |
|---|---|
| 1. Yield: 33% | 34 |
| 2. Catalyst: Very expensive<br>**10a**: Very expensive<br>**11b**: Expensive<br>Tetrahydrofuran: Very expensive | 5<br>5<br>3<br>5 |
| 3. Catalyst: –<br>**10a**: –<br>**11b**: Highly flammable<br>Tetrahydrofuran: Highly flammable | 0<br>0<br>5<br>5 |
| 4. Common setup | 0 |
| 5. Cooling to 0 ºC | 4 |
| 6. Classical chromatography | 10 |
| Total penalty points: | 76 |

Literature: *Tetrahedron Lett.* **2011**, *52*, 4430-4432

EcoScale = 100 – total penalty points = 66 (acceptable)

| Reaction parameters | Penalty points |
|---|---|
| 1. Yield: 96% | 2 |
| 2. Catalyst: Very expensive<br>**10a**: Expensive<br>**11b**: Expensive<br>Trifluoroacetic acid: Inexpensive<br>Chloroform: Very expensive | 5<br>3<br>3<br>0<br>5 |
| 3. Catalyst: –<br>**10a**: –<br>**11b**: Highly flammable<br>Trifluoroacetic acid: –<br>Chloroform: – | 0<br>0<br>5<br>0<br>0 |
| 4. Common setup | 0 |
| 5. Room temperature <24 h | 1 |
| 6. Classical chromatography | 10 |
| Total penalty points: | 34 |

Literature: *Chirality* **2013**, *25*, 668-672

EcoScale = 100 – total penalty points = 54 (acceptable)

| Reaction parameters | Penalty points |
|---|---|
| 1. Yield: 92% | 4 |
| 2. Catalyst: Very expensive<br>**10a**: Very expensive<br>**11b**: Expensive<br>Trifluoroacetic acid: Inexpensive<br>1,2-Dichloroethane: Expensive | 5<br>5<br>3<br>0<br>3 |
| 3. Catalyst: –<br>**10a**: –<br>**11b**: Highly flammable<br>Trifluoroacetic acid: –<br>1,2-Dichloroethane: Highly flammable and toxic | 0<br>0<br>5<br>0<br>10 |
| 4. Common setup | 0 |
| 5. Room temperature <24 h | 1 |
| 6. Classical chromatography | 10 |
| Total penalty points: | 46 |

Literature: *Eur. J. Org. Chem.* **2013**, *2013*, 2864-2868

EcoScale = 100 – total penalty points = 42 (inadequate)

| Reaction parameters | Penalty points |
|---|---|
| 1. Yield: 75% | 12 |
| 2. Catalyst: Very expensive<br>**10a**: Very expensive<br>**11b**: Expensive<br>Trifluoroacetic acid: Inexpensive<br>4-Chloro-2-nitrobenzoic acid: Inexpensive<br>Diethyl ether: Expensive | 5<br>5<br>3<br>0<br>0<br>3 |
| 3. Catalyst: –<br>**10a**: –<br>**11b**: Highly flammable<br>Trifluoroacetic acid: –<br>4-Chloro-2-nitrobenzoic acid: –<br>Diethyl ether: Extremely flammable | 0<br>0<br>5<br>0<br>0<br>10 |
| 4. Common setup | 0 |
| 5. Cooling < 0 ºC | 5 |
| 6. Classical chromatography | 10 |
| Total penalty points: | 58 |

Literature: *Tetrahedron* **2013**, *69*, 5438-5443

EcoScale = 100 – total penalty points = 59 (acceptable)

| Reaction parameters | Penalty points |
|---|---|
| 1. Yield: 98% | 1 |
| 2. Catalyst: Very expensive<br>**10a**: Very expensive<br>**11b**: Expensive<br>Tetrahydrofuran: Expensive | 5<br>5<br>3<br>3 |
| 3. Catalyst: –<br>**10a**: –<br>**11b**: Highly flammable<br>Tetrahydrofuran: Highly flammable | 0<br>0<br>5<br>5 |
| 4. Common setup | 0 |
| 5. Cooling to 0 ºC | 4 |
| 6. Classical chromatography | 10 |
| Total penalty points: | 41 |

Literature: *Org. Lett.* **2011**, *13*, 2638-2641

EcoScale = 100 – total penalty points = 65 (acceptable)

| Reaction parameters | Penalty points |
|---|---|
| 1. Yield: 99% | 1 |
| 2. Catalyst: Very expensive<br>**10a**: Very expensive<br>**11b**: Expensive<br>10-Camphorsulfonic acid: Inexpensive<br>Chloroform: Very expensive | 5<br>5<br>3<br>0<br>5 |
| 3. Catalyst: –<br>**10a**: –<br>**11b**: Highly flammable<br>10-Camphorsulfonic acid: –<br>Chloroform: – | 0<br>0<br>5<br>0<br>0 |
| 4. Common setup | 0 |
| 5. Room temperature <24 h | 1 |
| 6. Classical chromatography | 10 |
| Total penalty points: | 35 |

– Compound **12ba**

$$\text{E-factor (no purif.)} = \frac{0.0891 - 0.0665}{0.0665} = 0.34 \qquad \text{E-factor (purif.)} = \frac{19.3903 - 0.0665}{0.0665} = 291$$

EcoScale = 100 – total penalty points = 68 (acceptable)

| Reaction parameters | Penalty points |
|---|---|
| 1. Yield: 95% | 3 |
| 2. **9**: Very expensive<br>**10b**: Expensive<br>**11a**: Inexpensive<br>Acetic acid: Inexpensive | 5<br>3<br>0<br>0 |
| 3. **9**: –<br>**10b**: –<br>**11a**: Dangerous for the environment<br>Acetic acid: – | 0<br>0<br>5<br>0 |
| 4. Common setup<br>Inert gas atmosphere | 0<br>1 |
| 5. Cooling < 0 ºC | 5 |
| 6. Classical chromatography | 10 |
| Total penalty points: | 32 |

– Compound **12ca**

$$\text{E-factor (no purif.)} = \frac{0.0891 - 0.0686}{0.0686} = 0.30 \qquad \text{E-factor (purif.)} = \frac{19.3903 - 0.0686}{0.0686} = 282$$

EcoScale = 100 – total penalty points = 70 (acceptable)

| Reaction parameters | Penalty points |
|---|---|
| 1. Yield: 98% | 1 |
| 2. **9**: Very expensive<br>**10c**: Expensive<br>**11a**: Inexpensive<br>Acetic acid: Inexpensive | 5<br>3<br>0<br>0 |
| 3. **9**: –<br>**10c**: –<br>**11a**: Dangerous for the environment<br>Acetic acid: – | 0<br>0<br>5<br>0 |
| 4. Common setup<br>Inert gas atmosphere | 0<br>1 |
| 5. Cooling < 0 ºC | 5 |
| 6. Classical chromatography | 10 |
| Total penalty points: | 30 |

Literature: *J. Org. Chem.* **2011**, *76*, 4661-4664

EcoScale = 100 – total penalty points = 59 (acceptable)

| Reaction parameters | Penalty points |
|---|---|
| 1. Yield: 91% | 4 |
| 2. Catalyst: Very expensive<br>**10c**: Very expensive<br>**11a**: Inexpensive<br>Tetrahydrofuran: Expensive | 5<br>5<br>0<br>3 |
| 3. Catalyst: –<br>**10c**: –<br>**11a**: Dangerous for the environment<br>Tetrahydrofuran: Highly flammable | 0<br>0<br>5<br>5 |
| 4. Common setup | 0 |
| 5. Cooling to 0 ºC | 4 |
| 6. Classical chromatography | 10 |
| Total penalty points: | 41 |

Literature: *Org. Biomol. Chem.* **2010**, *8*, 4524-4526

EcoScale = 100 – total penalty points = 61 (acceptable)

| Reaction parameters | Penalty points |
|---|---|
| 1. Yield: 85% | 7 |
| 2. Catalyst: Very expensive<br>**10c**: Very expensive<br>**11a**: Inexpensive<br>Salicylic acid: Inexpensive<br>Dichloromethane: Expensive | 5<br>5<br>0<br>0<br>3 |
| 3. Catalyst: –<br>**10c**: –<br>**11a**: Dangerous for the environment<br>Salicylic acid: –<br>Dichloromethane: – | 0<br>0<br>5<br>0<br>0 |
| 4. Common setup | 0 |
| 5. Cooling to 0 ºC | 4 |
| 6. Classical chromatography | 10 |
| Total penalty points: | 39 |

– Compound **12da**

$$\text{E-factor (no purif.)} = \frac{0.0923 - 0.0725}{0.0725} = 0.27 \qquad \text{E-factor (purif.)} = \frac{19.3935 - 0.0725}{0.0725} = 267$$

EcoScale = 100 – total penalty points = 70 (acceptable)

| Reaction parameters | Penalty points |
|---|---|
| 1. Yield: 99% | 1 |
| 2. **9**: Very expensive<br>**10d**: Expensive<br>**11a**: Inexpensive<br>Acetic acid: Inexpensive | 5<br>3<br>0<br>0 |
| 3. **9**: –<br>**10d**: –<br>**11a**: Dangerous for the environment<br>Acetic acid: – | 0<br>0<br>5<br>0 |
| 4. Common setup<br>Inert gas atmosphere | 0<br>1 |
| 5. Cooling < 0 ºC | 5 |
| 6. Classical chromatography | 10 |
| Total penalty points: | 30 |

– Compound **12ea**

$$\text{E-factor (no purif.)} = \frac{0.1015 - 0.0791}{0.0791} = 0.28 \qquad \text{E-factor (purif.)} = \frac{19.4027 - 0.0791}{0.0791} = 244$$

EcoScale = 100 – total penalty points = 69 (acceptable)

| Reaction parameters | Penalty points |
|---|---|
| 1. Yield: 96% | 2 |
| 2. **9**: Very expensive<br>**10e**: Expensive<br>**11a**: Inexpensive<br>Acetic acid: Inexpensive | 5<br>3<br>0<br>0 |
| 3. **9**: –<br>**10e**: –<br>**11a**: Dangerous for the environment<br>Acetic acid: – | 0<br>0<br>5<br>0 |
| 4. Common setup<br>Inert gas atmosphere | 0<br>1 |
| 5. Cooling < 0 ºC | 5 |
| 6. Classical chromatography | 10 |
| Total penalty points: | 31 |

– Compound **12fa**

$$\text{E-factor (no purif.)} = \frac{0.0899 - 0.0658}{0.0658} = 0.37 \qquad \text{E-factor (purif.)} = \frac{19.3911 - 0.0658}{0.0658} = 294$$

EcoScale = 100 – total penalty points = 67 (acceptable)

| Reaction parameters | Penalty points |
|---|---|
| 1. Yield: 93% | 4 |
| 2. **9**: Very expensive<br>**10f**: Expensive<br>**11a**: Inexpensive<br>Acetic acid: Inexpensive | 5<br>3<br>0<br>0 |
| 3. **9**: –<br>**10f**: –<br>**11a**: Dangerous for the environment<br>Acetic acid: – | 0<br>0<br>5<br>0 |
| 4. Common setup<br>Inert gas atmosphere | 0<br>1 |
| 5. Cooling < 0 ºC | 5 |
| 6. Classical chromatography | 10 |
| Total penalty points: | 33 |

Literature: *J. Org. Chem.* **2011**, *76*, 4661-4664

EcoScale = 100 – total penalty points = 45 (inadequate)

| Reaction parameters | Penalty points |
|---|---|
| 1. Yield: 63% | 18 |
| 2. Catalyst: Very expensive<br>**10f**: Very expensive<br>**11a**: Inexpensive<br>Tetrahydrofuran: Expensive | 5<br>5<br>0<br>3 |
| 3. Catalyst: –<br>**10f**: –<br>**11a**: Dangerous for the environment<br>Tetrahydrofuran: Highly flammable | 0<br>0<br>5<br>5 |
| 4. Common setup | 0 |
| 5. Cooling to 0 ºC | 4 |
| 6. Classical chromatography | 10 |
| Total penalty points: | 55 |

Literature: *Org. Biomol. Chem.* **2010**, *8*, 4524-4526

EcoScale = 100 – total penalty points = 65 (acceptable)

| Reaction parameters | Penalty points |
|---|---|
| 1. Yield: 94% | 3 |
| 2. Catalyst: Very expensive<br>**10f**: Very expensive<br>**11a**: Inexpensive<br>Salicylic acid: Inexpensive<br>Dichloromethane: Expensive | 5<br>5<br>0<br>0<br>3 |
| 3. Catalyst: –<br>**10f**: –<br>**11a**: Dangerous for the environment<br>Salicylic acid: –<br>Dichloromethane: – | 0<br>0<br>5<br>0<br>0 |
| 4. Common setup | 0 |
| 5. Cooling to 0 ºC | 4 |
| 6. Classical chromatography | 10 |
| Total penalty points: | 35 |

– Compound **12ga**

$$\text{E-factor (no purif.)} = \frac{0.0932 - 0.0689}{0.0689} = 0.35 \qquad \text{E-factor (purif.)} = \frac{19.3944 - 0.0689}{0.0689} = 280$$

EcoScale = 100 – total penalty points = 67 (acceptable)

| Reaction parameters | Penalty points |
|---|---|
| 1. Yield: 93% | 4 |
| 2. **9**: Very expensive<br>**10g**: Expensive<br>**11a**: Inexpensive<br>Acetic acid: Inexpensive | 5<br>3<br>0<br>0 |
| 3. **9**: –<br>**10g**: –<br>**11a**: Dangerous for the environment<br>Acetic acid: – | 0<br>0<br>5<br>0 |
| 4. Common setup<br>Inert gas atmosphere | 0<br>1 |
| 5. Cooling < 0 ºC | 5 |
| 6. Classical chromatography | 10 |
| Total penalty points: | 33 |

Literature: *J. Org. Chem.* **2011**, *76*, 4661-4664

EcoScale = 100 – total penalty points = 22 (inadequate)

| Reaction parameters | Penalty points |
|---|---|
| 1. Yield: 29% | 36 |
| 2. Catalyst: Very expensive<br>**10f**: Very expensive<br>**11a**: Expensive<br>Tetrahydrofuran: Very expensive | 5<br>5<br>3<br>5 |
| 3. Catalyst: –<br>**10f**: –<br>**11a**: Dangerous for the environment<br>Tetrahydrofuran: Highly flammable | 0<br>0<br>5<br>5 |
| 4. Common setup | 0 |
| 5. Cooling to 0 ºC | 4 |
| 6. Classical chromatography | 10 |
| Total penalty points: | 78 |

Literature: *Org. Biomol. Chem.* **2010**, *8*, 4524-4526

EcoScale = 100 – total penalty points = 27 (inadequate)

| Reaction parameters | Penalty points |
|---|---|
| 1. Yield: 30% | 35 |
| 2. Catalyst: Very expensive<br>**10g**: Very expensive<br>**11a**: Expensive<br>Salicylic acid: Expensive<br>Dichloromethane: Expensive | 5<br>5<br>3<br>3<br>3 |
| 3. Catalyst: –<br>**10g**: –<br>**11a**: Dangerous for the environment<br>Salicylic acid: –<br>Dichloromethane: – | 0<br>0<br>5<br>0<br>0 |
| 4. Common setup | 0 |
| 5. Cooling to 0 ºC | 4 |
| 6. Classical chromatography | 10 |
| Total penalty points: | 73 |

– Compound **12ha**

$$\text{E-factor (no purif.)} = \frac{0.0932 - 0.0674}{0.0674} = 0.38 \qquad \text{E-factor (purif.)} = \frac{19.3944 - 0.0674}{0.0674} = 287$$

EcoScale = 100 – total penalty points = 66 (acceptable)

| Reaction parameters | Penalty points |
|---|---|
| 1. Yield: 91% | 5 |
| 2. **9**: Very expensive<br>**10h**: Expensive<br>**11a**: Inexpensive<br>Acetic acid: Inexpensive | 5<br>3<br>0<br>0 |
| 3. **9**: –<br>**10h**: –<br>**11a**: Dangerous for the environment<br>Acetic acid: – | 0<br>0<br>5<br>0 |
| 4. Common setup<br>Inert gas atmosphere | 0<br>1 |
| 5. Cooling < 0 ºC | 5 |
| 6. Classical chromatography | 10 |
| Total penalty points: | 34 |

Literature: *J. Org. Chem.* **2011**, *76*, 4661-4664

EcoScale = 100 – total penalty points = 49 (inadequate)

| Reaction parameters | Penalty points |
|---|---|
| 1. Yield: 72% | 14 |
| 2. Catalyst: Very expensive<br>**10h**: Very expensive<br>**11a**: Inexpensive<br>Tetrahydrofuran: Expensive | 5<br>5<br>0<br>3 |
| 3. Catalyst: –<br>**10h**: –<br>**11a**: Dangerous for the environment<br>Tetrahydrofuran: Highly flammable | 0<br>0<br>5<br>5 |
| 4. Common setup | 0 |
| 5. Cooling to 0 ºC | 4 |
| 6. Classical chromatography | 10 |
| Total penalty points: | 51 |

Literature: *Org. Biomol. Chem.* **2010**, *8*, 4524-4526

EcoScale = 100 – total penalty points = 63 (acceptable)

| Reaction parameters | Penalty points |
|---|---|
| 1. Yield: 90% | 5 |
| 2. Catalyst: Very expensive<br>**10g**: Very expensive<br>**11a**: Inexpensive<br>Salicylic acid: Inexpensive<br>Dichloromethane: Expensive | 5<br>5<br>0<br>0<br>3 |
| 3. Catalyst: –<br>**10g**: –<br>**11a**: Dangerous for the environment<br>Salicylic acid: –<br>Dichloromethane: – | 0<br>0<br>5<br>0<br>0 |
| 4. Common setup | 0 |
| 5. Cooling to 0 ºC | 4 |
| 6. Classical chromatography | 10 |
| Total penalty points: | 37 |

– Compound **12ia**

$$\text{E-factor (no purif.)} = \frac{0.0932 - 0.0667}{0.0667} = 0.40 \qquad \text{E-factor (purif.)} = \frac{19.3944 - 0.0667}{0.0667} = 290$$

EcoScale = 100 – total penalty points = 66 (acceptable)

| Reaction parameters | Penalty points |
|---|---|
| 1. Yield: 90% | 5 |
| 2. **9**: Very expensive<br>**10i**: Expensive<br>**11a**: Inexpensive<br>Acetic acid: Inexpensive | 5<br>3<br>0<br>0 |
| 3. **9**: –<br>**10i**: –<br>**11a**: Dangerous for the environment<br>Acetic acid: – | 0<br>0<br>5<br>0 |
| 4. Common setup<br>Inert gas atmosphere | 0<br>1 |
| 5. Cooling < 0 ºC | 5 |
| 6. Classical chromatography | 10 |
| Total penalty points: | 34 |

– Compound **12ja**

$$\text{E-factor (no purif.)} = \frac{0.1021 - 0.0789}{0.0789} = 0.29 \qquad \text{E-factor (purif.)} = \frac{19.4033 - 0.0789}{0.0789} = 245$$

EcoScale = 100 – total penalty points = 68 (acceptable)

| Reaction parameters | Penalty points |
|---|---|
| 1. Yield: 95% | 3 |
| 2. **9**: Very expensive<br>**10j**: Expensive<br>**11a**: Inexpensive<br>Acetic acid: Inexpensive | 5<br>3<br>0<br>0 |
| 3. **9**: –<br>**10j**: –<br>**11a**: Dangerous for the environment<br>Acetic acid: – | 0<br>0<br>5<br>0 |
| 4. Common setup<br>Inert gas atmosphere | 0<br>1 |
| 5. Cooling < 0 ºC | 5 |
| 6. Classical chromatography | 10 |
| Total penalty points: | 32 |

Literature: *J. Org. Chem.* **2011**, *76*, 4661-4664

EcoScale = 100 – total penalty points = 52 (acceptable)

| Reaction parameters | Penalty points |
|---|---|
| 1. Yield: 77% | 11 |
| 2. Catalyst: Very expensive<br>**10j**: Very expensive<br>**11a**: Inexpensive<br>Tetrahydrofuran: Expensive | 5<br>5<br>0<br>3 |
| 3. Catalyst: –<br>**10j**: –<br>**11a**: Dangerous for the environment<br>Tetrahydrofuran: Highly flammable | 0<br>0<br>5<br>5 |
| 4. Common setup | 0 |
| 5. Cooling to 0 ºC | 4 |
| 6. Classical chromatography | 10 |
| Total penalty points: | 48 |

Literature: *Org. Biomol. Chem.* **2010**, *8*, 4524-4526

EcoScale = 100 – total penalty points = 64 (acceptable)

| Reaction parameters | Penalty points |
|---|---|
| 1. Yield: 92% | 4 |
| 2. Catalyst: Very expensive<br>**10j**: Very expensive<br>**11a**: Inexpensive<br>Salicylic acid: Inexpensive<br>Dichloromethane: Expensive | 5<br>5<br>0<br>0<br>3 |
| 3. Catalyst: –<br>**10j**: –<br>**11a**: Dangerous for the environment<br>Salicylic acid: –<br>Dichloromethane: – | 0<br>0<br>5<br>0<br>0 |
| 4. Common setup | 0 |
| 5. Cooling to 0 ºC | 4 |
| 6. Classical chromatography | 10 |
| Total penalty points: | 36 |

<u>– Compound **12ka**</u>

$$\text{E-factor (no purif.)} = \frac{0.0953 - 0.0648}{0.0648} = 0.47 \qquad \text{E-factor (purif.)} = \frac{19.3965 - 0.0648}{0.0648} = 298$$

EcoScale = 100 – total penalty points = 63 (acceptable)

| Reaction parameters | Penalty points |
|---|---|
| 1. Yield: 85% | 8 |
| 2. **9**: Very expensive<br>**10k**: Expensive<br>**11a**: Inexpensive<br>Acetic acid: Inexpensive | 5<br>3<br>0<br>0 |
| 3. **9**: –<br>**10k**: –<br>**11a**: Dangerous for the environment<br>Acetic acid: – | 0<br>0<br>5<br>0 |
| 4. Common setup<br>Inert gas atmosphere | 0<br>1 |
| 5. Cooling < 0 ºC | 5 |
| 6. Classical chromatography | 10 |
| Total penalty points: | 37 |

Literature: *Org. Biomol. Chem.* **2010**, *8*, 4524-4526

EcoScale = 100 – total penalty points = 59 (acceptable)

| Reaction parameters | Penalty points |
|---|---|
| 1. Yield: 81% | 9 |
| 2. Catalyst: Very expensive<br>**10k**: Very expensive<br>**11a**: Inexpensive<br>Salicylic acid: Inexpensive<br>Dichloromethane: Expensive | 5<br>5<br>0<br>0<br>3 |
| 3. Catalyst: –<br>**10k**: –<br>**11a**: Dangerous for the environment<br>Salicylic acid: –<br>Dichloromethane: – | 0<br>0<br>5<br>0<br>0 |
| 4. Common setup | 0 |
| 5. Cooling to 0 ºC | 4 |
| 6. Classical chromatography | 10 |
| Total penalty points: | 41 |

– Compound **12la**

$$\text{E-factor (no purif.)} = \frac{0.0963 - 0.0726}{0.0726} = 0.33 \qquad \text{E-factor (purif.)} = \frac{19.3975 - 0.0726}{0.0726} = 266$$

EcoScale = 100 – total penalty points = 68 (acceptable)

| Reaction parameters | Penalty points |
|---|---|
| 1. Yield: 94% | 3 |
| 2. **9**: Very expensive<br>**10l**: Expensive<br>**11a**: Inexpensive<br>Acetic acid: Inexpensive | 5<br>3<br>0<br>0 |
| 3. **9**: –<br>**10l**: –<br>**11a**: Dangerous for the environment<br>Acetic acid: – | 0<br>0<br>5<br>0 |
| 4. Common setup<br>Inert gas atmosphere | 0<br>1 |
| 5. Cooling < 0 ºC | 5 |
| 6. Classical chromatography | 10 |
| Total penalty points: | 32 |

Literature: *J. Org. Chem.* **2011**, *76*, 4661-4664

EcoScale = 100 – total penalty points = 56 (acceptable)

| Reaction parameters | Penalty points |
|---|---|
| 1. Yield: 86% | 7 |
| 2. Catalyst: Very expensive<br>**10l**: Very expensive<br>**11a**: Inexpensive<br>Tetrahydrofuran: Expensive | 5<br>5<br>0<br>3 |
| 3. Catalyst: –<br>**10l**: –<br>**11a**: Dangerous for the environment<br>Tetrahydrofuran: Highly flammable | 0<br>0<br>5<br>5 |
| 4. Common setup | 0 |
| 5. Cooling to 0 ºC | 4 |
| 6. Classical chromatography | 10 |
| Total penalty points: | 44 |

Literature: *Org. Biomol. Chem.* **2010**, *8*, 4524-4526

EcoScale = 100 – total penalty points = 63 (acceptable)

| Reaction parameters | Penalty points |
|---|---|
| 1. Yield: 90% | 5 |
| 2. Catalyst: Very expensive<br>**10l**: Very expensive<br>**11a**: Inexpensive<br>Salicylic acid: Inexpensive<br>Dichloromethane: Expensive | 5<br>5<br>0<br>0<br>3 |
| 3. Catalyst: –<br>**10l**: –<br>**11a**: Dangerous for the environment<br>Salicylic acid: –<br>Dichloromethane: – | 0<br>0<br>5<br>0<br>0 |
| 4. Common setup | 0 |
| 5. Cooling to 0 ºC | 4 |
| 6. Classical chromatography | 10 |
| Total penalty points: | 37 |

– Compound **12ma**

$$\text{E-factor (no purif.)} = \frac{0.0891 - 0.0693}{0.0693} = 0.29 \qquad \text{E-factor (purif.)} = \frac{19.3903 - 0.0693}{0.0693} = 279$$

EcoScale = 100 – total penalty points = 70 (acceptable)

| Reaction parameters | Penalty points |
|---|---|
| 1. Yield: 99% | 1 |
| 2. **9**: Very expensive<br>**10m**: Expensive<br>**11a**: Inexpensive<br>Acetic acid: Inexpensive | 5<br>3<br>0<br>0 |
| 3. **9**: –<br>**10m**: –<br>**11a**: Dangerous for the environment<br>Acetic acid: – | 0<br>0<br>5<br>0 |
| 4. Common setup<br>Inert gas atmosphere | 0<br>1 |
| 5. Cooling < 0 ºC | 5 |
| 6. Classical chromatography | 10 |
| Total penalty points: | 30 |

– Compound **12na**

$$\text{E-factor (no purif.)} = \frac{0.0915 - 0.0673}{0.0673} = 0.36 \qquad \text{E-factor (purif.)} = \frac{19.3927 - 0.0673}{0.0673} = 287$$

EcoScale = 100 – total penalty points = 67 (acceptable)

| Reaction parameters | Penalty points |
|---|---|
| 1. Yield: 93% | 4 |
| 2. **9**: Very expensive<br>**10n**: Expensive<br>**11a**: Inexpensive<br>Acetic acid: Inexpensive | 5<br>3<br>0<br>0 |
| 3. **9**: –<br>**10n**: –<br>**11a**: Dangerous for the environment<br>Acetic acid: – | 0<br>0<br>5<br>0 |
| 4. Common setup<br>Inert gas atmosphere | 0<br>1 |
| 5. Cooling < 0 ºC | 5 |
| 6. Classical chromatography | 10 |
| Total penalty points: | 33 |

– Compound **12oa**

$$\text{E-factor (no purif.)} = \frac{0.0976 - 0.0754}{0.0754} = 0.29 \qquad \text{E-factor (purif.)} = \frac{19.3988 - 0.0754}{0.0754} = 256$$

EcoScale = 100 – total penalty points = 72 (acceptable)

| Reaction parameters | Penalty points |
|---|---|
| 1. Yield: 96% | 2 |
| 2. **9**: Very expensive<br>**10o**: Inexpensive<br>**11a**: Inexpensive<br>Acetic acid: Inexpensive | 5<br>0<br>0<br>0 |
| 3. **9**: –<br>**10o**: –<br>**11a**: Dangerous for the environment<br>Acetic acid: – | 0<br>0<br>5<br>0 |
| 4. Common setup<br>Inert gas atmosphere | 0<br>1 |
| 5. Cooling < 0 ºC | 5 |
| 6. Classical chromatography | 10 |
| Total penalty points: | 28 |

– Compound **12pa**

$$\text{E-factor (no purif.)} = \frac{0.0767 - 0.0559}{0.0559} = 0.37 \qquad \text{E-factor (purif.)} = \frac{19.3779 - 0.0559}{0.0559} = 346$$

EcoScale = 100 – total penalty points = 67 (acceptable)

| Reaction parameters | Penalty points |
|---|---|
| 1. Yield: 97% | 2 |
| 2. **9**: Very expensive<br>**10p**: Inexpensive<br>**11a**: Inexpensive<br>Acetic acid: Inexpensive | 5<br>0<br>0<br>0 |
| 3. **9**: –<br>**10p**: Highly flammable<br>**11a**: Dangerous for the environment<br>Acetic acid: – | 0<br>5<br>5<br>0 |
| 4. Common setup<br>Inert gas atmosphere | 0<br>1 |
| 5. Cooling < 0 ºC | 5 |
| 6. Classical chromatography | 10 |
| Total penalty points: | 33 |

– Compound **12qa**

$$\text{E-factor (no purif.)} = \frac{0.0795 - 0.0592}{0.0592} = 0.34 \qquad \text{E-factor (purif.)} = \frac{19.3807 - 0.0592}{0.0592} = 326$$

EcoScale = 100 – total penalty points = 63 (acceptable)

| Reaction parameters | Penalty points |
|---|---|
| 1. Yield: 98% | 1 |
| 2. **9**: Very expensive<br>**10q**: Very expensive<br>**11a**: Inexpensive<br>Acetic acid: Inexpensive | 5<br>5<br>0<br>0 |
| 3. **9**: –<br>**10q**: Highly flammable<br>**11a**: Dangerous for the environment<br>Acetic acid: – | 0<br>5<br>5<br>0 |
| 4. Common setup<br>Inert gas atmosphere | 0<br>1 |
| 5. Cooling < 0 ºC | 5 |
| 6. Classical chromatography | 10 |
| Total penalty points: | 37 |

Literature: *J. Org. Chem.* **2011**, *76*, 4661-4664

EcoScale = 100 – total penalty points = 57 (acceptable)

| Reaction parameters | Penalty points |
|---|---|
| 1. Yield: 88% | 6 |
| 2. Catalyst: Very expensive<br>**10q**: Very expensive<br>**11a**: Inexpensive<br>Tetrahydrofuran: Expensive | 5<br>5<br>0<br>3 |
| 3. Catalyst: –<br>**10q**: –<br>**11a**: Dangerous for the environment<br>Tetrahydrofuran: Highly flammable | 0<br>0<br>5<br>5 |
| 4. Common setup | 0 |
| 5. Cooling to 0 ºC | 4 |
| 6. Classical chromatography | 10 |
| Total penalty points: | 43 |

Literature: *Org. Biomol. Chem.* **2010**, *8*, 4524-4526

EcoScale = 100 – total penalty points = 39 (inadequate)

| Reaction parameters | Penalty points |
|---|---|
| 1. Yield: 54% | 23 |
| 2. Catalyst: Very expensive<br>**10q**: Very expensive<br>**11a**: Expensive<br>Salicylic acid: Expensive<br>Dichloromethane: Expensive | 5<br>5<br>3<br>3<br>3 |
| 3. Catalyst: –<br>**10q**: –<br>**11a**: Dangerous for the environment<br>Salicylic acid: –<br>Dichloromethane: – | 0<br>0<br>5<br>0<br>0 |
| 4. Common setup | 0 |
| 5. Cooling to 0 ºC | 4 |
| 6. Classical chromatography | 10 |
| Total penalty points: | 61 |

– Compound **12ra**

$$\text{E-factor (no purif.)} = \frac{0.0936 - 0.0724}{0.0724} = 0.29 \qquad \text{E-factor (purif.)} = \frac{19.3948 - 0.0724}{0.0724} = 267$$

EcoScale = 100 – total penalty points = 68 (acceptable)

| Reaction parameters | Penalty points |
|---|---|
| 1. Yield: 97% | 1 |
| 2. **9**: Very expensive<br>**10r**: Inexpensive<br>**11a**: Inexpensive<br>Acetic acid: Inexpensive | 5<br>0<br>0<br>0 |
| 3. **9**: –<br>**10r**: Highly flammable<br>**11a**: Dangerous for the environment<br>Acetic acid: – | 0<br>5<br>5<br>0 |
| 4. Common setup<br>Inert gas atmosphere | 0<br>1 |
| 5. Cooling < 0 ºC | 5 |
| 6. Classical chromatography | 10 |
| Total penalty points: | 32 |

– Compound **12sa**

$$\text{E-factor (no purif.)} = \frac{0.0875 - 0.0656}{0.0656} = 0.33 \qquad \text{E-factor (purif.)} = \frac{19.3887 - 0.0656}{0.0656} = 295$$

EcoScale = 100 – total penalty points = 67 (acceptable)

| Reaction parameters | Penalty points |
|---|---|
| 1. Yield: 96% | 2 |
| 2. **9**: Very expensive<br>**10s**: Inexpensive<br>**11a**: Inexpensive<br>Acetic acid: Inexpensive | 5<br>0<br>0<br>0 |
| 3. **9**: –<br>**10s**: Highly flammable<br>**11a**: Dangerous for the environment<br>Acetic acid: – | 0<br>5<br>5<br>0 |
| 4. Common setup<br>Inert gas atmosphere | 0<br>1 |
| 5. Cooling < 0 ºC | 5 |
| 6. Classical chromatography | 10 |
| Total penalty points: | 33 |

– Scaled-up reaction

$$\text{E-factor (no purif.)} = \frac{1.2949 - 0.9586}{0.9586} = 0.35 \qquad \text{E-factor (purif.)} = \frac{39.8974 - 0.9586}{0.9586} = 41$$

EcoScale = 100 – total penalty points = 68 (acceptable)

| Reaction parameters | Penalty points |
|---|---|
| 1. Yield: 95% | 3 |
| 2. **9**: Very expensive<br>**10a**: Expensive<br>**11a**: Inexpensive<br>Acetic acid: Inexpensive | 5<br>3<br>0<br>0 |
| 3. **9**: –<br>**10a**: –<br>**11a**: Dangerous for the environment<br>Acetic acid: – | 0<br>0<br>5<br>0 |
| 4. Common setup<br>Inert gas atmosphere | 0<br>1 |
| 5. Cooling < 0 ºC | 5 |
| 6. Classical chromatography | 10 |
| Total penalty points: | 32 |

# IX. Computational details

All structures were optimized using density functional theory (DFT) as implemented in Gaussian,[14] with wB97XD[15] as functional and def2tzvpp[16] as basis set, introducing implicit solvation factors with the IEF-PCM method, and tetrahydrofuran as solvent.[17-19] The stationary points were characterized by frequency calculations in order to verify that they have the right number of imaginary frequencies.

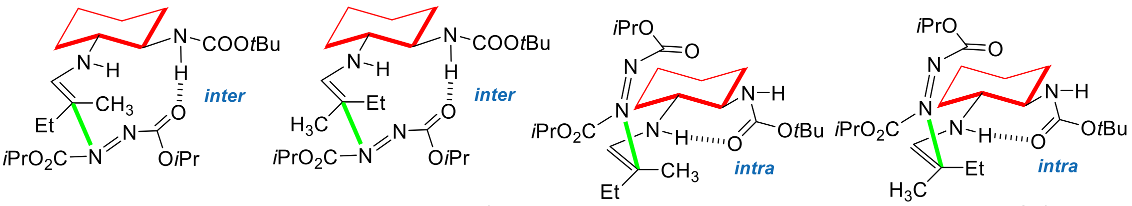


**Figure S106.** Computed transition states for the reaction between the enamine from **9** and **10p** and **11a**. Energies are given in kcal/mol.

**TS1** G (hartrees): -1763.360798 Frequency: -175.8 cm$^{-1}$

Standard orientation:

| Center Number | Atomic Number | Atomic Type | Coordinates (Angstroms) X | Y | Z |
|---|---|---|---|---|---|
| 1 | 6 | 0 | -0.278072 | -3.428120 | 0.514940 |
| 2 | 6 | 0 | -1.078392 | -4.604900 | -0.065110 |
| 3 | 6 | 0 | -1.171251 | -2.196950 | 0.680780 |
| 4 | 1 | 0 | 0.558058 | -3.187271 | -0.154720 |
| 5 | 1 | 0 | 0.157218 | -3.711860 | 1.480810 |
| 6 | 6 | 0 | -1.862631 | -1.804760 | -0.657080 |
| 7 | 1 | 0 | -1.966011 | -2.414480 | 1.405040 |
| 8 | 7 | 0 | -0.446551 | -1.013780 | 1.171360 |
| 9 | 6 | 0 | -1.743152 | -4.227660 | -1.396040 |
| 10 | 1 | 0 | -1.850082 | -4.910080 | 0.654210 |
| 11 | 1 | 0 | -0.413583 | -5.465840 | -0.196210 |
| 12 | 6 | 0 | -2.644381 | -2.995069 | -1.238690 |
| 13 | 1 | 0 | -1.061821 | -1.517020 | -1.345100 |
| 14 | 7 | 0 | -2.665670 | -0.592489 | -0.502780 |
| 15 | 1 | 0 | -3.061081 | -2.694319 | -2.206270 |
| 16 | 1 | 0 | -3.487652 | -3.225239 | -0.580540 |
| 17 | 1 | 0 | -0.963682 | -4.023550 | -2.143730 |
| 18 | 1 | 0 | -2.328272 | -5.071789 | -1.778170 |
| 19 | 6 | 0 | -3.811820 | -0.543069 | 0.235150 |
| 20 | 1 | 0 | -2.355430 | 0.251571 | -0.990500 |
| 21 | 8 | 0 | -4.247111 | -1.466449 | 0.916520 |
| 22 | 8 | 0 | -4.377810 | 0.683182 | 0.114590 |
| 23 | 6 | 0 | -5.618280 | 1.021652 | 0.823930 |
| 24 | 6 | 0 | -5.401490 | 0.938172 | 2.339040 |
| 25 | 1 | 0 | -6.293410 | 1.301602 | 2.859750 |
| 26 | 1 | 0 | -4.556800 | 1.568302 | 2.636010 |
| 27 | 1 | 0 | -5.205640 | -0.088438 | 2.649020 |
| 28 | 6 | 0 | -5.861809 | 2.469722 | 0.390770 |
| 29 | 1 | 0 | -6.774539 | 2.853873 | 0.856100 |
| 30 | 1 | 0 | -5.970809 | 2.532962 | -0.695460 |
| 31 | 1 | 0 | -5.024039 | 3.106822 | 0.688050 |

| | | | | | |
|---|---|---|---|---|---|
| 32 | 6 | 0 | -6.760070 | 0.114993 | 0.351580 |
| 33 | 1 | 0 | -6.579921 | -0.920838 | 0.639390 |
| 34 | 1 | 0 | -6.859550 | 0.169173 | -0.737020 |
| 35 | 1 | 0 | -7.703510 | 0.448513 | 0.796080 |
| 36 | 6 | 0 | 0.831969 | -0.897541 | 1.494690 |
| 37 | 6 | 0 | 1.480920 | 0.357449 | 1.584290 |
| 38 | 6 | 0 | 0.644930 | 1.599759 | 1.811670 |
| 39 | 1 | 0 | -1.000230 | -0.167750 | 1.103870 |
| 40 | 1 | 0 | 1.428799 | -1.798671 | 1.507530 |
| 41 | 1 | 0 | 5.299410 | 0.012967 | -2.532360 |
| 42 | 1 | 0 | 2.153902 | 5.001189 | -1.349380 |
| 43 | 6 | 0 | 5.102709 | -1.058703 | -2.435270 |
| 44 | 1 | 0 | 5.800449 | -1.592673 | -3.088910 |
| 45 | 1 | 0 | 5.284989 | -1.360243 | -1.402920 |
| 46 | 6 | 0 | 1.137032 | 5.076709 | -0.951480 |
| 47 | 1 | 0 | 0.764122 | 6.086459 | -1.149460 |
| 48 | 1 | 0 | 1.127862 | 4.091709 | -3.572310 |
| 49 | 1 | 0 | 1.179952 | 4.933479 | 0.132080 |
| 50 | 6 | 0 | 3.670719 | -1.376092 | -2.857390 |
| 51 | 8 | 0 | 2.705420 | -0.511482 | -2.180260 |
| 52 | 1 | 0 | 3.532179 | -1.062452 | -3.896810 |
| 53 | 7 | 0 | 1.685840 | 0.455599 | -0.433650 |
| 54 | 6 | 0 | 2.562140 | -0.590631 | -0.845580 |
| 55 | 6 | 0 | 0.136402 | 4.189870 | -3.118490 |
| 56 | 8 | 0 | 3.168169 | -1.357852 | -0.119870 |
| 57 | 6 | 0 | 0.226012 | 4.045110 | -1.602610 |
| 58 | 1 | 0 | -0.268428 | 5.172930 | -3.381030 |
| 59 | 8 | 0 | 0.791811 | 2.747009 | -1.269280 |
| 60 | 6 | 0 | -0.053089 | 1.688380 | -1.284450 |
| 61 | 7 | 0 | 0.522950 | 0.446529 | -1.062090 |
| 62 | 6 | 0 | 3.294649 | -2.851042 | -2.739780 |
| 63 | 1 | 0 | -0.522979 | 3.425060 | -3.533860 |
| 64 | 1 | 0 | 3.912488 | -3.437572 | -3.427900 |
| 65 | 1 | 0 | -0.779178 | 4.090550 | -1.172880 |
| 66 | 1 | 0 | 3.451928 | -3.220032 | -1.725510 |
| 67 | 8 | 0 | -1.251109 | 1.797360 | -1.551850 |
| 68 | 1 | 0 | 2.246548 | -2.999171 | -3.017040 |
| 69 | 1 | 0 | 0.317850 | 1.633329 | 2.857900 |
| 70 | 1 | 0 | -0.247840 | 1.642390 | 1.182740 |
| 71 | 1 | 0 | 1.228871 | 2.497409 | 1.611150 |
| 72 | 1 | 0 | 2.594689 | -1.228662 | 3.527790 |
| 73 | 6 | 0 | 3.273050 | -0.459292 | 3.170370 |
| 74 | 6 | 0 | 2.855140 | 0.424118 | 2.164690 |
| 75 | 6 | 0 | 3.740540 | 1.431868 | 1.748880 |
| 76 | 1 | 0 | 3.435701 | 2.107438 | 0.955970 |
| 77 | 6 | 0 | 5.010420 | 1.538147 | 2.310400 |
| 78 | 1 | 0 | 5.685741 | 2.316697 | 1.966970 |
| 79 | 6 | 0 | 5.418550 | 0.648017 | 3.306380 |
| 80 | 1 | 0 | 6.407650 | 0.734667 | 3.746450 |
| 81 | 6 | 0 | 4.544030 | -0.350562 | 3.734190 |
| 82 | 1 | 0 | 4.846499 | -1.043762 | 4.513890 |

---------------------------------------------------------------------

**TS2** G (hartrees): -1763.359310 Frequency: -268.2 $cm^{-1}$

Standard orientation:

| Center Number | Atomic Number | Atomic Type | Coordinates (Angstroms) X | Y | Z |
|---|---|---|---|---|---|
| 1 | 6 | 0 | -2.153140 | -3.380530 | -1.240320 |
| 2 | 6 | 0 | -3.302750 | -4.212490 | -0.653690 |
| 3 | 6 | 0 | -2.026460 | -2.038180 | -0.498440 |
| 4 | 1 | 0 | -2.334730 | -3.180490 | -2.304040 |
| 5 | 1 | 0 | -1.210760 | -3.934630 | -1.174850 |
| 6 | 6 | 0 | -3.346100 | -1.238280 | -0.578450 |
| 7 | 1 | 0 | -1.801490 | -2.227180 | 0.558610 |
| 8 | 7 | 0 | -0.925450 | -1.208990 | -1.029270 |
| 9 | 6 | 0 | -4.625580 | -3.434670 | -0.678270 |
| 10 | 1 | 0 | -3.058450 | -4.487840 | 0.381110 |
| 11 | 1 | 0 | -3.398030 | -5.148840 | -1.213410 |
| 12 | 6 | 0 | -4.492170 | -2.072410 | 0.017340 |
| 13 | 1 | 0 | -3.557950 | -1.046420 | -1.635630 |
| 14 | 7 | 0 | -3.275230 | 0.067740 | 0.063700 |
| 15 | 1 | 0 | -5.419620 | -1.498090 | -0.069420 |
| 16 | 1 | 0 | -4.307590 | -2.217910 | 1.090490 |
| 17 | 1 | 0 | -4.935500 | -3.278980 | -1.720320 |
| 18 | 1 | 0 | -5.418500 | -4.019030 | -0.199400 |
| 19 | 6 | 0 | -2.929100 | 1.183260 | -0.644860 |
| 20 | 1 | 0 | -3.067230 | 0.101880 | 1.059620 |
| 21 | 8 | 0 | -2.597470 | 1.163000 | -1.835510 |
| 22 | 8 | 0 | -3.038530 | 2.273340 | 0.125440 |
| 23 | 6 | 0 | -2.709300 | 3.627890 | -0.366740 |
| 24 | 6 | 0 | -3.708470 | 4.023420 | -1.457000 |
| 25 | 1 | 0 | -3.528280 | 5.059310 | -1.761070 |
| 26 | 1 | 0 | -4.734030 | 3.951940 | -1.081700 |
| 27 | 1 | 0 | -3.604580 | 3.379920 | -2.331720 |
| 28 | 6 | 0 | -2.900580 | 4.482180 | 0.888580 |
| 29 | 1 | 0 | -2.717160 | 5.534660 | 0.654590 |
| 30 | 1 | 0 | -2.204970 | 4.173790 | 1.673960 |
| 31 | 1 | 0 | -3.921000 | 4.384670 | 1.270200 |
| 32 | 6 | 0 | -1.258230 | 3.692310 | -0.849010 |
| 33 | 1 | 0 | -1.132030 | 3.163980 | -1.794400 |
| 34 | 1 | 0 | -0.591570 | 3.248570 | -0.105260 |
| 35 | 1 | 0 | -0.976550 | 4.740100 | -0.996580 |
| 36 | 6 | 0 | 0.339090 | -1.549470 | -1.021030 |
| 37 | 6 | 0 | 1.387510 | -0.648090 | -1.386070 |
| 38 | 6 | 0 | 1.038320 | 0.402530 | -2.427710 |
| 39 | 1 | 0 | -1.207750 | -0.320000 | -1.450980 |
| 40 | 1 | 0 | 0.582710 | -2.504690 | -0.569510 |
| 41 | 1 | 0 | 4.141430 | 2.462230 | 2.565360 |
| 42 | 1 | 0 | 1.897560 | -3.476810 | 2.784270 |
| 43 | 6 | 0 | 4.237430 | 3.097080 | 1.679520 |
| 44 | 1 | 0 | 4.834670 | 3.972890 | 1.952950 |
| 45 | 1 | 0 | 4.763110 | 2.542330 | 0.901130 |
| 46 | 6 | 0 | 0.807980 | -3.577780 | 2.814980 |
| 47 | 1 | 0 | 0.554020 | -4.290250 | 3.605660 |
| 48 | 1 | 0 | 1.675370 | -1.453120 | 4.414110 |
| 49 | 1 | 0 | 0.470580 | -3.993580 | 1.861050 |
| 50 | 6 | 0 | 2.862360 | 3.554720 | 1.200200 |
| 51 | 8 | 0 | 1.937440 | 2.428850 | 1.059090 |

| | | | | | |
|---|---|---|---|---|---|
| 52 | 1 | 0 | 2.381140 | 4.113860 | 2.008690 |
| 53 | 7 | 0 | 1.356560 | 0.359440 | 0.350990 |
| 54 | 6 | 0 | 2.196250 | 1.461580 | 0.147740 |
| 55 | 6 | 0 | 0.591810 | -1.609090 | 4.411360 |
| 56 | 8 | 0 | 3.126710 | 1.494850 | -0.645900 |
| 57 | 6 | 0 | 0.144160 | -2.237140 | 3.095350 |
| 58 | 1 | 0 | 0.334900 | -2.265700 | 5.248760 |
| 59 | 8 | 0 | 0.508290 | -1.356180 | 1.992790 |
| 60 | 6 | 0 | -0.320360 | -0.296300 | 1.740690 |
| 61 | 7 | 0 | 0.127770 | 0.624400 | 0.819990 |
| 62 | 6 | 0 | 2.894760 | 4.437490 | -0.044550 |
| 63 | 1 | 0 | 0.095530 | -0.648100 | 4.565220 |
| 64 | 1 | 0 | 3.431310 | 5.363650 | 0.186530 |
| 65 | 1 | 0 | -0.944060 | -2.342060 | 3.092870 |
| 66 | 1 | 0 | 3.396000 | 3.936850 | -0.873450 |
| 67 | 8 | 0 | -1.413060 | -0.187380 | 2.308070 |
| 68 | 1 | 0 | 1.878870 | 4.703170 | -0.351280 |
| 69 | 1 | 0 | 1.869320 | 1.094150 | -2.553570 |
| 70 | 1 | 0 | 0.161160 | 0.984550 | -2.133030 |
| 71 | 1 | 0 | 0.818380 | -0.066970 | -3.393530 |
| 72 | 6 | 0 | 3.512550 | -1.251420 | -2.621090 |
| 73 | 6 | 0 | 2.734610 | -1.300090 | -1.454290 |
| 74 | 6 | 0 | 3.220810 | -2.026320 | -0.353800 |
| 75 | 6 | 0 | 4.445000 | -2.689440 | -0.423170 |
| 76 | 1 | 0 | 4.808460 | -3.237590 | 0.441510 |
| 77 | 6 | 0 | 5.208240 | -2.640999 | -1.592330 |
| 78 | 1 | 0 | 6.162580 | -3.156529 | -1.645800 |
| 79 | 6 | 0 | 4.736510 | -1.917810 | -2.689190 |
| 80 | 1 | 0 | 5.321370 | -1.871009 | -3.603350 |
| 81 | 1 | 0 | 3.163250 | -0.696900 | -3.485340 |
| 82 | 1 | 0 | 2.646600 | -2.031510 | 0.566880 |

---------------------------------------------------------------------

**TS3** G (hartrees): -1763.356426 Frequency: -270.6 $cm^{-1}$

Standard orientation:

| Center Number | Atomic Number | Atomic Type | Coordinates (Angstroms) X | Y | Z |
|---|---|---|---|---|---|
| 1 | 6 | 0 | -0.983698 | -3.745160 | -0.971510 |
| 2 | 6 | 0 | -1.981878 | -4.765400 | -0.400020 |
| 3 | 6 | 0 | -1.225549 | -2.355100 | -0.366530 |
| 4 | 1 | 0 | -1.091858 | -3.681440 | -2.062090 |
| 5 | 1 | 0 | 0.040062 | -4.078780 | -0.769430 |
| 6 | 6 | 0 | -2.673539 | -1.885421 | -0.614440 |
| 7 | 1 | 0 | -1.071799 | -2.392960 | 0.721660 |
| 8 | 7 | 0 | -0.307519 | -1.331500 | -0.897460 |
| 9 | 6 | 0 | -3.434158 | -4.309141 | -0.598400 |
| 10 | 1 | 0 | -1.784728 | -4.899360 | 0.672270 |
| 11 | 1 | 0 | -1.814807 | -5.739740 | -0.871970 |
| 12 | 6 | 0 | -3.662109 | -2.905431 | -0.020660 |
| 13 | 1 | 0 | -2.847479 | -1.829871 | -1.695920 |
| 14 | 7 | 0 | -2.849240 | -0.536831 | -0.097820 |
| 15 | 1 | 0 | -4.677129 | -2.556842 | -0.232900 |
| 16 | 1 | 0 | -3.544379 | -2.919721 | 1.071160 |
| 17 | 1 | 0 | -3.669428 | -4.302281 | -1.671490 |
| 18 | 1 | 0 | -4.121528 | -5.024921 | -0.134260 |
| 19 | 6 | 0 | -3.803730 | 0.281919 | -0.641240 |

| | | | | | |
|---|---|---|---|---|---|
| 20 | 1 | 0 | -2.428970 | -0.289671 | 0.802210 |
| 21 | 8 | 0 | -4.440990 | 0.012999 | -1.651360 |
| 22 | 8 | 0 | -3.908631 | 1.408679 | 0.097130 |
| 23 | 6 | 0 | -4.820531 | 2.492778 | -0.294520 |
| 24 | 6 | 0 | -6.269631 | 1.994768 | -0.272610 |
| 25 | 1 | 0 | -6.949781 | 2.833967 | -0.451400 |
| 26 | 1 | 0 | -6.508011 | 1.567598 | 0.706560 |
| 27 | 1 | 0 | -6.433630 | 1.237258 | -1.039220 |
| 28 | 6 | 0 | -4.585781 | 3.533438 | 0.803100 |
| 29 | 1 | 0 | -5.212622 | 4.412988 | 0.627930 |
| 30 | 1 | 0 | -3.538392 | 3.847059 | 0.817580 |
| 31 | 1 | 0 | -4.832801 | 3.119708 | 1.784850 |
| 32 | 6 | 0 | -4.418351 | 3.047729 | -1.665080 |
| 33 | 1 | 0 | -4.562351 | 2.300668 | -2.446070 |
| 34 | 1 | 0 | -3.368021 | 3.355479 | -1.654670 |
| 35 | 1 | 0 | -5.027542 | 3.926738 | -1.899520 |
| 36 | 6 | 0 | 0.990821 | -1.435279 | -1.066290 |
| 37 | 6 | 0 | 1.794630 | -0.306709 | -1.425700 |
| 38 | 6 | 0 | 1.173340 | 0.638871 | -2.449290 |
| 39 | 1 | 0 | -0.719320 | -0.396730 | -0.951360 |
| 40 | 1 | 0 | 1.458101 | -2.375279 | -0.791430 |
| 41 | 1 | 0 | 3.903809 | 3.525362 | 2.282330 |
| 42 | 1 | 0 | 3.145861 | -2.306898 | 3.340490 |
| 43 | 6 | 0 | 3.766898 | 4.127312 | 1.379070 |
| 44 | 1 | 0 | 4.120768 | 5.142042 | 1.590310 |
| 45 | 1 | 0 | 4.370038 | 3.702362 | 0.575830 |
| 46 | 6 | 0 | 2.175131 | -2.808379 | 3.275140 |
| 47 | 1 | 0 | 2.091942 | -3.505489 | 4.114740 |
| 48 | 1 | 0 | 1.986010 | -0.388129 | 4.670360 |
| 49 | 1 | 0 | 2.148502 | -3.385769 | 2.346100 |
| 50 | 6 | 0 | 2.290408 | 4.175531 | 0.992610 |
| 51 | 8 | 0 | 1.699429 | 2.842401 | 0.938470 |
| 52 | 1 | 0 | 1.726488 | 4.616441 | 1.821100 |
| 53 | 7 | 0 | 1.592360 | 0.690121 | 0.271890 |
| 54 | 6 | 0 | 2.148659 | 1.957011 | 0.023410 |
| 55 | 6 | 0 | 1.045270 | -0.941279 | 4.585400 |
| 56 | 8 | 0 | 3.010939 | 2.191112 | -0.808350 |
| 57 | 6 | 0 | 1.039971 | -1.794629 | 3.320840 |
| 58 | 1 | 0 | 0.934691 | -1.575899 | 5.470960 |
| 59 | 8 | 0 | 1.216130 | -0.940589 | 2.158140 |
| 60 | 6 | 0 | 0.117690 | -0.265290 | 1.712320 |
| 61 | 7 | 0 | 0.333510 | 0.675941 | 0.729900 |
| 62 | 6 | 0 | 2.000958 | 4.979501 | -0.273000 |
| 63 | 1 | 0 | 0.215830 | -0.231119 | 4.566930 |
| 64 | 1 | 0 | 2.279118 | 6.026251 | -0.109910 |
| 65 | 1 | 0 | 0.072001 | -2.296210 | 3.228080 |
| 66 | 1 | 0 | 2.564478 | 4.592972 | -1.122940 |
| 67 | 8 | 0 | -1.009100 | -0.497080 | 2.167360 |
| 68 | 1 | 0 | 0.932898 | 4.945341 | -0.507890 |
| 69 | 1 | 0 | 1.837609 | 1.482221 | -2.627680 |
| 70 | 1 | 0 | 0.218300 | 1.039441 | -2.096120 |
| 71 | 1 | 0 | 0.988440 | 0.117731 | -3.395580 |
| 72 | 6 | 0 | 3.962230 | -0.416368 | -2.735500 |
| 73 | 6 | 0 | 3.250050 | -0.637758 | -1.548510 |
| 74 | 6 | 0 | 3.913611 | -1.240528 | -0.467530 |
| 75 | 6 | 0 | 5.248811 | -1.624377 | -0.578650 |
| 76 | 1 | 0 | 5.749771 | -2.079537 | 0.271180 |
| 77 | 6 | 0 | 5.945761 | -1.410917 | -1.769200 |
| 78 | 1 | 0 | 6.987131 | -1.707157 | -1.855120 |
| 79 | 6 | 0 | 5.297390 | -0.802497 | -2.844770 |

| | | | | | |
|---|---|---|---|---|---|
| 80 | 1 | 0 | 5.831990 | -0.625957 | -3.773750 |
| 81 | 1 | 0 | 3.475330 | 0.056242 | -3.581790 |
| 82 | 1 | 0 | 3.383361 | -1.357568 | 0.472610 |

**TS4** G (hartrees): -1763.354735 Frequency: -187.2 $cm^{-1}$

Standard orientation:

| Center Number | Atomic Number | Atomic Type | Coordinates (Angstroms) X | Y | Z |
|---|---|---|---|---|---|
| 1 | 6 | 0 | -0.212571 | -3.668111 | -0.603029 |
| 2 | 6 | 0 | 0.577599 | -4.944581 | -0.275919 |
| 3 | 6 | 0 | 0.691490 | -2.587091 | -1.212759 |
| 4 | 1 | 0 | -0.680041 | -3.271000 | 0.306481 |
| 5 | 1 | 0 | -1.024671 | -3.912960 | -1.296659 |
| 6 | 6 | 0 | 1.909560 | -2.291571 | -0.309239 |
| 7 | 1 | 0 | 1.076150 | -2.968361 | -2.174239 |
| 8 | 7 | 0 | -0.009250 | -1.324041 | -1.493309 |
| 9 | 6 | 0 | 1.775559 | -4.649661 | 0.634601 |
| 10 | 1 | 0 | 0.933959 | -5.402221 | -1.209429 |
| 11 | 1 | 0 | -0.094281 | -5.673611 | 0.189891 |
| 12 | 6 | 0 | 2.683059 | -3.582571 | 0.011251 |
| 13 | 1 | 0 | 1.546160 | -1.839911 | 0.619041 |
| 14 | 7 | 0 | 2.739350 | -1.271601 | -0.942649 |
| 15 | 1 | 0 | 3.505879 | -3.323691 | 0.683741 |
| 16 | 1 | 0 | 3.130149 | -3.972751 | -0.915189 |
| 17 | 1 | 0 | 1.415239 | -4.296081 | 1.609991 |
| 18 | 1 | 0 | 2.344659 | -5.566381 | 0.825151 |
| 19 | 6 | 0 | 3.627130 | -0.544321 | -0.165759 |
| 20 | 1 | 0 | 3.052720 | -1.437841 | -1.891109 |
| 21 | 8 | 0 | 3.649960 | -0.576691 | 1.050071 |
| 22 | 8 | 0 | 4.439660 | 0.163399 | -0.978099 |
| 23 | 6 | 0 | 5.480110 | 1.048928 | -0.423589 |
| 24 | 6 | 0 | 6.525200 | 0.206448 | 0.314971 |
| 25 | 1 | 0 | 7.347760 | 0.847978 | 0.647361 |
| 26 | 1 | 0 | 6.939360 | -0.560212 | -0.348149 |
| 27 | 1 | 0 | 6.085290 | -0.277392 | 1.187811 |
| 28 | 6 | 0 | 6.077740 | 1.679358 | -1.683839 |
| 29 | 1 | 0 | 6.894881 | 2.354858 | -1.414019 |
| 30 | 1 | 0 | 5.318120 | 2.252028 | -2.223239 |
| 31 | 1 | 0 | 6.472200 | 0.909268 | -2.353709 |
| 32 | 6 | 0 | 4.852460 | 2.118118 | 0.474831 |
| 33 | 1 | 0 | 4.466680 | 1.679589 | 1.394701 |
| 34 | 1 | 0 | 4.021981 | 2.608409 | -0.037509 |
| 35 | 1 | 0 | 5.611601 | 2.867038 | 0.725731 |
| 36 | 6 | 0 | -1.323780 | -1.131440 | -1.496029 |
| 37 | 6 | 0 | -1.915070 | 0.155400 | -1.525079 |
| 38 | 6 | 0 | -1.178160 | 1.285930 | -2.219089 |
| 39 | 1 | 0 | 0.601540 | -0.509991 | -1.463659 |
| 40 | 1 | 0 | -1.950090 | -1.988140 | -1.294029 |
| 41 | 1 | 0 | -4.131780 | 0.549730 | 3.595641 |
| 42 | 1 | 0 | -1.865239 | 5.131560 | 0.978711 |
| 43 | 6 | 0 | -3.957020 | -0.530050 | 3.606161 |
| 44 | 1 | 0 | -4.376030 | -0.933050 | 4.534281 |
| 45 | 1 | 0 | -4.475750 | -0.980890 | 2.759031 |
| 46 | 6 | 0 | -1.126009 | 5.176730 | 0.172601 |
| 47 | 1 | 0 | -0.786979 | 6.212930 | 0.074611 |

| | | | | | |
|---|---|---|---|---|---|
| 48 | 1 | 0 | 0.082861 | 4.563599 | 2.619781 |
| 49 | 1 | 0 | -1.617879 | 4.887270 | -0.760749 |
| 50 | 6 | 0 | -2.460340 | -0.825640 | 3.555681 |
| 51 | 8 | 0 | -1.806480 | -0.109910 | 2.463091 |
| 52 | 1 | 0 | -1.979820 | -0.360120 | 4.421821 |
| 53 | 7 | 0 | -1.463410 | 0.533710 | 0.346341 |
| 54 | 6 | 0 | -2.124120 | -0.401210 | 1.190161 |
| 55 | 6 | 0 | 0.773851 | 4.618179 | 1.772241 |
| 56 | 8 | 0 | -2.928810 | -1.249430 | 0.844601 |
| 57 | 6 | 0 | 0.054891 | 4.263289 | 0.473851 |
| 58 | 1 | 0 | 1.175951 | 5.635579 | 1.718441 |
| 59 | 8 | 0 | -0.489599 | 2.921759 | 0.569441 |
| 60 | 6 | 0 | 0.374390 | 1.894709 | 0.328141 |
| 61 | 7 | 0 | -0.158820 | 0.624589 | 0.539911 |
| 62 | 6 | 0 | -2.115140 | -2.312900 | 3.550291 |
| 63 | 1 | 0 | 1.602741 | 3.928869 | 1.945641 |
| 64 | 1 | 0 | -2.442690 | -2.763740 | 4.493061 |
| 65 | 1 | 0 | 0.772401 | 4.275249 | -0.352529 |
| 66 | 1 | 0 | -2.606480 | -2.829860 | 2.724911 |
| 67 | 8 | 0 | 1.545390 | 2.067199 | 0.016971 |
| 68 | 1 | 0 | -1.032660 | -2.449980 | 3.468081 |
| 69 | 1 | 0 | -1.316870 | 1.186390 | -3.301739 |
| 70 | 1 | 0 | -0.104340 | 1.296079 | -2.022519 |
| 71 | 1 | 0 | -1.582660 | 2.250680 | -1.912739 |
| 72 | 1 | 0 | -3.638130 | -1.524280 | -2.860849 |
| 73 | 6 | 0 | -4.144590 | -0.688150 | -2.386879 |
| 74 | 6 | 0 | -3.405210 | 0.248630 | -1.651529 |
| 75 | 6 | 0 | -4.082630 | 1.339690 | -1.084359 |
| 76 | 1 | 0 | -3.520440 | 2.060710 | -0.499669 |
| 77 | 6 | 0 | -5.460830 | 1.475540 | -1.233809 |
| 78 | 1 | 0 | -5.969099 | 2.320670 | -0.778119 |
| 79 | 6 | 0 | -6.189050 | 0.531021 | -1.960459 |
| 80 | 1 | 0 | -7.263220 | 0.639741 | -2.078369 |
| 81 | 6 | 0 | -5.524700 | -0.550890 | -2.537349 |
| 82 | 1 | 0 | -6.078120 | -1.287669 | -3.112669 |

---------------------------------------------------------------------

**TS1**-*E* G (hartrees): -1763.360798 Frequency: -175.8 $cm^{-1}$

Standard orientation:

| Center Number | Atomic Number | Atomic Type | Coordinates (Angstroms) X | Y | Z |
|---|---|---|---|---|---|
| 1 | 6 | 0 | -0.278072 | -3.428120 | 0.514940 |
| 2 | 6 | 0 | -1.078392 | -4.604900 | -0.065110 |
| 3 | 6 | 0 | -1.171251 | -2.196950 | 0.680780 |
| 4 | 1 | 0 | 0.558058 | -3.187271 | -0.154720 |
| 5 | 1 | 0 | 0.157218 | -3.711860 | 1.480810 |
| 6 | 6 | 0 | -1.862631 | -1.804760 | -0.657080 |
| 7 | 1 | 0 | -1.966011 | -2.414480 | 1.405040 |
| 8 | 7 | 0 | -0.446551 | -1.013780 | 1.171360 |
| 9 | 6 | 0 | -1.743152 | -4.227660 | -1.396040 |
| 10 | 1 | 0 | -1.850082 | -4.910080 | 0.654210 |
| 11 | 1 | 0 | -0.413583 | -5.465840 | -0.196210 |
| 12 | 6 | 0 | -2.644381 | -2.995069 | -1.238690 |
| 13 | 1 | 0 | -1.061821 | -1.517020 | -1.345100 |
| 14 | 7 | 0 | -2.665670 | -0.592489 | -0.502780 |
| 15 | 1 | 0 | -3.061081 | -2.694319 | -2.206270 |

| | | | | | |
|---|---|---|---|---|---|
| 16 | 1 | 0 | -3.487652 | -3.225239 | -0.580540 |
| 17 | 1 | 0 | -0.963682 | -4.023550 | -2.143730 |
| 18 | 1 | 0 | -2.328272 | -5.071789 | -1.778170 |
| 19 | 6 | 0 | -3.811820 | -0.543069 | 0.235150 |
| 20 | 1 | 0 | -2.355430 | 0.251571 | -0.990500 |
| 21 | 8 | 0 | -4.247111 | -1.466449 | 0.916520 |
| 22 | 8 | 0 | -4.377810 | 0.683182 | 0.114590 |
| 23 | 6 | 0 | -5.618280 | 1.021652 | 0.823930 |
| 24 | 6 | 0 | -5.401490 | 0.938172 | 2.339040 |
| 25 | 1 | 0 | -6.293410 | 1.301602 | 2.859750 |
| 26 | 1 | 0 | -4.556800 | 1.568302 | 2.636010 |
| 27 | 1 | 0 | -5.205640 | -0.088438 | 2.649020 |
| 28 | 6 | 0 | -5.861809 | 2.469722 | 0.390770 |
| 29 | 1 | 0 | -6.774539 | 2.853873 | 0.856100 |
| 30 | 1 | 0 | -5.970809 | 2.532962 | -0.695460 |
| 31 | 1 | 0 | -5.024039 | 3.106822 | 0.688050 |
| 32 | 6 | 0 | -6.760070 | 0.114993 | 0.351580 |
| 33 | 1 | 0 | -6.579921 | -0.920838 | 0.639390 |
| 34 | 1 | 0 | -6.859550 | 0.169173 | -0.737020 |
| 35 | 1 | 0 | -7.703510 | 0.448513 | 0.796080 |
| 36 | 6 | 0 | 0.831969 | -0.897541 | 1.494690 |
| 37 | 6 | 0 | 1.480920 | 0.357449 | 1.584290 |
| 38 | 6 | 0 | 0.644930 | 1.599759 | 1.811670 |
| 39 | 1 | 0 | -1.000230 | -0.167750 | 1.103870 |
| 40 | 1 | 0 | 1.428799 | -1.798671 | 1.507530 |
| 41 | 1 | 0 | 5.299410 | 0.012967 | -2.532360 |
| 42 | 1 | 0 | 2.153902 | 5.001189 | -1.349380 |
| 43 | 6 | 0 | 5.102709 | -1.058703 | -2.435270 |
| 44 | 1 | 0 | 5.800449 | -1.592673 | -3.088910 |
| 45 | 1 | 0 | 5.284989 | -1.360243 | -1.402920 |
| 46 | 6 | 0 | 1.137032 | 5.076709 | -0.951480 |
| 47 | 1 | 0 | 0.764122 | 6.086459 | -1.149460 |
| 48 | 1 | 0 | 1.127862 | 4.091709 | -3.572310 |
| 49 | 1 | 0 | 1.179952 | 4.933479 | 0.132080 |
| 50 | 6 | 0 | 3.670719 | -1.376092 | -2.857390 |
| 51 | 8 | 0 | 2.705420 | -0.511482 | -2.180260 |
| 52 | 1 | 0 | 3.532179 | -1.062452 | -3.896810 |
| 53 | 7 | 0 | 1.685840 | 0.455599 | -0.433650 |
| 54 | 6 | 0 | 2.562140 | -0.590631 | -0.845580 |
| 55 | 6 | 0 | 0.136402 | 4.189870 | -3.118490 |
| 56 | 8 | 0 | 3.168169 | -1.357852 | -0.119870 |
| 57 | 6 | 0 | 0.226012 | 4.045110 | -1.602610 |
| 58 | 1 | 0 | -0.268428 | 5.172930 | -3.381030 |
| 59 | 8 | 0 | 0.791811 | 2.747009 | -1.269280 |
| 60 | 6 | 0 | -0.053089 | 1.688380 | -1.284450 |
| 61 | 7 | 0 | 0.522950 | 0.446529 | -1.062090 |
| 62 | 6 | 0 | 3.294649 | -2.851042 | -2.739780 |
| 63 | 1 | 0 | -0.522979 | 3.425060 | -3.533860 |
| 64 | 1 | 0 | 3.912488 | -3.437572 | -3.427900 |
| 65 | 1 | 0 | -0.779178 | 4.090550 | -1.172880 |
| 66 | 1 | 0 | 3.451928 | -3.220032 | -1.725510 |
| 67 | 8 | 0 | -1.251109 | 1.797360 | -1.551850 |
| 68 | 1 | 0 | 2.246548 | -2.999171 | -3.017040 |
| 69 | 1 | 0 | 0.317850 | 1.633329 | 2.857900 |
| 70 | 1 | 0 | -0.247840 | 1.642390 | 1.182740 |
| 71 | 1 | 0 | 1.228871 | 2.497409 | 1.611150 |
| 72 | 1 | 0 | 2.594689 | -1.228662 | 3.527790 |
| 73 | 6 | 0 | 3.273050 | -0.459292 | 3.170370 |
| 74 | 6 | 0 | 2.855140 | 0.424118 | 2.164690 |
| 75 | 6 | 0 | 3.740540 | 1.431868 | 1.748880 |

| | | | | | |
|---|---|---|---|---|---|
| 76 | 1 | 0 | 3.435701 | 2.107438 | 0.955970 |
| 77 | 6 | 0 | 5.010420 | 1.538147 | 2.310400 |
| 78 | 1 | 0 | 5.685741 | 2.316697 | 1.966970 |
| 79 | 6 | 0 | 5.418550 | 0.648017 | 3.306380 |
| 80 | 1 | 0 | 6.407650 | 0.734667 | 3.746450 |
| 81 | 6 | 0 | 4.544030 | -0.350562 | 3.734190 |
| 82 | 1 | 0 | 4.846499 | -1.043762 | 4.513890 |

---------------------------------------------------------------------

**TS2**-*E* G (hartrees): -1763.359310 Frequency: -268.2 $cm^{-1}$

Standard orientation:

| Center Number | Atomic Number | Atomic Type | Coordinates (Angstroms) X | Y | Z |
|---|---|---|---|---|---|
| 1 | 6 | 0 | -2.153140 | -3.380530 | -1.240320 |
| 2 | 6 | 0 | -3.302750 | -4.212490 | -0.653690 |
| 3 | 6 | 0 | -2.026460 | -2.038180 | -0.498440 |
| 4 | 1 | 0 | -2.334730 | -3.180490 | -2.304040 |
| 5 | 1 | 0 | -1.210760 | -3.934630 | -1.174850 |
| 6 | 6 | 0 | -3.346100 | -1.238280 | -0.578450 |
| 7 | 1 | 0 | -1.801490 | -2.227180 | 0.558610 |
| 8 | 7 | 0 | -0.925450 | -1.208990 | -1.029270 |
| 9 | 6 | 0 | -4.625580 | -3.434670 | -0.678270 |
| 10 | 1 | 0 | -3.058450 | -4.487840 | 0.381110 |
| 11 | 1 | 0 | -3.398030 | -5.148840 | -1.213410 |
| 12 | 6 | 0 | -4.492170 | -2.072410 | 0.017340 |
| 13 | 1 | 0 | -3.557950 | -1.046420 | -1.635630 |
| 14 | 7 | 0 | -3.275230 | 0.067740 | 0.063700 |
| 15 | 1 | 0 | -5.419620 | -1.498090 | -0.069420 |
| 16 | 1 | 0 | -4.307590 | -2.217910 | 1.090490 |
| 17 | 1 | 0 | -4.935500 | -3.278980 | -1.720320 |
| 18 | 1 | 0 | -5.418500 | -4.019030 | -0.199400 |
| 19 | 6 | 0 | -2.929100 | 1.183260 | -0.644860 |
| 20 | 1 | 0 | -3.067230 | 0.101880 | 1.059620 |
| 21 | 8 | 0 | -2.597470 | 1.163000 | -1.835510 |
| 22 | 8 | 0 | -3.038530 | 2.273340 | 0.125440 |
| 23 | 6 | 0 | -2.709300 | 3.627890 | -0.366740 |
| 24 | 6 | 0 | -3.708470 | 4.023420 | -1.457000 |
| 25 | 1 | 0 | -3.528280 | 5.059310 | -1.761070 |
| 26 | 1 | 0 | -4.734030 | 3.951940 | -1.081700 |
| 27 | 1 | 0 | -3.604580 | 3.379920 | -2.331720 |
| 28 | 6 | 0 | -2.900580 | 4.482180 | 0.888580 |
| 29 | 1 | 0 | -2.717160 | 5.534660 | 0.654590 |
| 30 | 1 | 0 | -2.204970 | 4.173790 | 1.673960 |
| 31 | 1 | 0 | -3.921000 | 4.384670 | 1.270200 |
| 32 | 6 | 0 | -1.258230 | 3.692310 | -0.849010 |
| 33 | 1 | 0 | -1.132030 | 3.163980 | -1.794400 |
| 34 | 1 | 0 | -0.591570 | 3.248570 | -0.105260 |
| 35 | 1 | 0 | -0.976550 | 4.740100 | -0.996580 |
| 36 | 6 | 0 | 0.339090 | -1.549470 | -1.021030 |
| 37 | 6 | 0 | 1.387510 | -0.648090 | -1.386070 |
| 38 | 6 | 0 | 1.038320 | 0.402530 | -2.427710 |
| 39 | 1 | 0 | -1.207750 | -0.320000 | -1.450980 |
| 40 | 1 | 0 | 0.582710 | -2.504690 | -0.569510 |
| 41 | 1 | 0 | 4.141430 | 2.462230 | 2.565360 |
| 42 | 1 | 0 | 1.897560 | -3.476810 | 2.784270 |
| 43 | 6 | 0 | 4.237430 | 3.097080 | 1.679520 |

| | | | | | |
|---|---|---|---|---|---|
| 44 | 1 | 0 | 4.834670 | 3.972890 | 1.952950 |
| 45 | 1 | 0 | 4.763110 | 2.542330 | 0.901130 |
| 46 | 6 | 0 | 0.807980 | -3.577780 | 2.814980 |
| 47 | 1 | 0 | 0.554020 | -4.290250 | 3.605660 |
| 48 | 1 | 0 | 1.675370 | -1.453120 | 4.414110 |
| 49 | 1 | 0 | 0.470580 | -3.993580 | 1.861050 |
| 50 | 6 | 0 | 2.862360 | 3.554720 | 1.200200 |
| 51 | 8 | 0 | 1.937440 | 2.428850 | 1.059090 |
| 52 | 1 | 0 | 2.381140 | 4.113860 | 2.008690 |
| 53 | 7 | 0 | 1.356560 | 0.359440 | 0.350990 |
| 54 | 6 | 0 | 2.196250 | 1.461580 | 0.147740 |
| 55 | 6 | 0 | 0.591810 | -1.609090 | 4.411360 |
| 56 | 8 | 0 | 3.126710 | 1.494850 | -0.645900 |
| 57 | 6 | 0 | 0.144160 | -2.237140 | 3.095350 |
| 58 | 1 | 0 | 0.334900 | -2.265700 | 5.248760 |
| 59 | 8 | 0 | 0.508290 | -1.356180 | 1.992790 |
| 60 | 6 | 0 | -0.320360 | -0.296300 | 1.740690 |
| 61 | 7 | 0 | 0.127770 | 0.624400 | 0.819990 |
| 62 | 6 | 0 | 2.894760 | 4.437490 | -0.044550 |
| 63 | 1 | 0 | 0.095530 | -0.648100 | 4.565220 |
| 64 | 1 | 0 | 3.431310 | 5.363650 | 0.186530 |
| 65 | 1 | 0 | -0.944060 | -2.342060 | 3.092870 |
| 66 | 1 | 0 | 3.396000 | 3.936850 | -0.873450 |
| 67 | 8 | 0 | -1.413060 | -0.187380 | 2.308070 |
| 68 | 1 | 0 | 1.878870 | 4.703170 | -0.351280 |
| 69 | 1 | 0 | 1.869320 | 1.094150 | -2.553570 |
| 70 | 1 | 0 | 0.161160 | 0.984550 | -2.133030 |
| 71 | 1 | 0 | 0.818380 | -0.066970 | -3.393530 |
| 72 | 6 | 0 | 3.512550 | -1.251420 | -2.621090 |
| 73 | 6 | 0 | 2.734610 | -1.300090 | -1.454290 |
| 74 | 6 | 0 | 3.220810 | -2.026320 | -0.353800 |
| 75 | 6 | 0 | 4.445000 | -2.689440 | -0.423170 |
| 76 | 1 | 0 | 4.808460 | -3.237590 | 0.441510 |
| 77 | 6 | 0 | 5.208240 | -2.640999 | -1.592330 |
| 78 | 1 | 0 | 6.162580 | -3.156529 | -1.645800 |
| 79 | 6 | 0 | 4.736510 | -1.917810 | -2.689190 |
| 80 | 1 | 0 | 5.321370 | -1.871009 | -3.603350 |
| 81 | 1 | 0 | 3.163250 | -0.696900 | -3.485340 |
| 82 | 1 | 0 | 2.646600 | -2.031510 | 0.566880 |

---------------------------------------------------------------------

**TS3**-*E* G (hartrees): -1763.356426 Frequency: -270.6 $cm^{-1}$

Standard orientation:

| Center Number | Atomic Number | Atomic Type | Coordinates (Angstroms) X | Y | Z |
|---|---|---|---|---|---|
| 1 | 6 | 0 | -0.983698 | -3.745160 | -0.971510 |
| 2 | 6 | 0 | -1.981878 | -4.765400 | -0.400020 |
| 3 | 6 | 0 | -1.225549 | -2.355100 | -0.366530 |
| 4 | 1 | 0 | -1.091858 | -3.681440 | -2.062090 |
| 5 | 1 | 0 | 0.040062 | -4.078780 | -0.769430 |
| 6 | 6 | 0 | -2.673539 | -1.885421 | -0.614440 |
| 7 | 1 | 0 | -1.071799 | -2.392960 | 0.721660 |
| 8 | 7 | 0 | -0.307519 | -1.331500 | -0.897460 |
| 9 | 6 | 0 | -3.434158 | -4.309141 | -0.598400 |
| 10 | 1 | 0 | -1.784728 | -4.899360 | 0.672270 |
| 11 | 1 | 0 | -1.814807 | -5.739740 | -0.871970 |

| | | | | | |
|---|---|---|---|---|---|
| 12 | 6 | 0 | -3.662109 | -2.905431 | -0.020660 |
| 13 | 1 | 0 | -2.847479 | -1.829871 | -1.695920 |
| 14 | 7 | 0 | -2.849240 | -0.536831 | -0.097820 |
| 15 | 1 | 0 | -4.677129 | -2.556842 | -0.232900 |
| 16 | 1 | 0 | -3.544379 | -2.919721 | 1.071160 |
| 17 | 1 | 0 | -3.669428 | -4.302281 | -1.671490 |
| 18 | 1 | 0 | -4.121528 | -5.024921 | -0.134260 |
| 19 | 6 | 0 | -3.803730 | 0.281919 | -0.641240 |
| 20 | 1 | 0 | -2.428970 | -0.289671 | 0.802210 |
| 21 | 8 | 0 | -4.440990 | 0.012999 | -1.651360 |
| 22 | 8 | 0 | -3.908631 | 1.408679 | 0.097130 |
| 23 | 6 | 0 | -4.820531 | 2.492778 | -0.294520 |
| 24 | 6 | 0 | -6.269631 | 1.994768 | -0.272610 |
| 25 | 1 | 0 | -6.949781 | 2.833967 | -0.451400 |
| 26 | 1 | 0 | -6.508011 | 1.567598 | 0.706560 |
| 27 | 1 | 0 | -6.433630 | 1.237258 | -1.039220 |
| 28 | 6 | 0 | -4.585781 | 3.533438 | 0.803100 |
| 29 | 1 | 0 | -5.212622 | 4.412988 | 0.627930 |
| 30 | 1 | 0 | -3.538392 | 3.847059 | 0.817580 |
| 31 | 1 | 0 | -4.832801 | 3.119708 | 1.784850 |
| 32 | 6 | 0 | -4.418351 | 3.047729 | -1.665080 |
| 33 | 1 | 0 | -4.562351 | 2.300668 | -2.446070 |
| 34 | 1 | 0 | -3.368021 | 3.355479 | -1.654670 |
| 35 | 1 | 0 | -5.027542 | 3.926738 | -1.899520 |
| 36 | 6 | 0 | 0.990821 | -1.435279 | -1.066290 |
| 37 | 6 | 0 | 1.794630 | -0.306709 | -1.425700 |
| 38 | 6 | 0 | 1.173340 | 0.638871 | -2.449290 |
| 39 | 1 | 0 | -0.719320 | -0.396730 | -0.951360 |
| 40 | 1 | 0 | 1.458101 | -2.375279 | -0.791430 |
| 41 | 1 | 0 | 3.903809 | 3.525362 | 2.282330 |
| 42 | 1 | 0 | 3.145861 | -2.306898 | 3.340490 |
| 43 | 6 | 0 | 3.766898 | 4.127312 | 1.379070 |
| 44 | 1 | 0 | 4.120768 | 5.142042 | 1.590310 |
| 45 | 1 | 0 | 4.370038 | 3.702362 | 0.575830 |
| 46 | 6 | 0 | 2.175131 | -2.808379 | 3.275140 |
| 47 | 1 | 0 | 2.091942 | -3.505489 | 4.114740 |
| 48 | 1 | 0 | 1.986010 | -0.388129 | 4.670360 |
| 49 | 1 | 0 | 2.148502 | -3.385769 | 2.346100 |
| 50 | 6 | 0 | 2.290408 | 4.175531 | 0.992610 |
| 51 | 8 | 0 | 1.699429 | 2.842401 | 0.938470 |
| 52 | 1 | 0 | 1.726488 | 4.616441 | 1.821100 |
| 53 | 7 | 0 | 1.592360 | 0.690121 | 0.271890 |
| 54 | 6 | 0 | 2.148659 | 1.957011 | 0.023410 |
| 55 | 6 | 0 | 1.045270 | -0.941279 | 4.585400 |
| 56 | 8 | 0 | 3.010939 | 2.191112 | -0.808350 |
| 57 | 6 | 0 | 1.039971 | -1.794629 | 3.320840 |
| 58 | 1 | 0 | 0.934691 | -1.575899 | 5.470960 |
| 59 | 8 | 0 | 1.216130 | -0.940589 | 2.158140 |
| 60 | 6 | 0 | 0.117690 | -0.265290 | 1.712320 |
| 61 | 7 | 0 | 0.333510 | 0.675941 | 0.729900 |
| 62 | 6 | 0 | 2.000958 | 4.979501 | -0.273000 |
| 63 | 1 | 0 | 0.215830 | -0.231119 | 4.566930 |
| 64 | 1 | 0 | 2.279118 | 6.026251 | -0.109910 |
| 65 | 1 | 0 | 0.072001 | -2.296210 | 3.228080 |
| 66 | 1 | 0 | 2.564478 | 4.592972 | -1.122940 |
| 67 | 8 | 0 | -1.009100 | -0.497080 | 2.167360 |
| 68 | 1 | 0 | 0.932898 | 4.945341 | -0.507890 |
| 69 | 1 | 0 | 1.837609 | 1.482221 | -2.627680 |
| 70 | 1 | 0 | 0.218300 | 1.039441 | -2.096120 |
| 71 | 1 | 0 | 0.988440 | 0.117731 | -3.395580 |

| | | | | | |
|---|---|---|---|---|---|
| 72 | 6 | 0 | 3.962230 | -0.416368 | -2.735500 |
| 73 | 6 | 0 | 3.250050 | -0.637758 | -1.548510 |
| 74 | 6 | 0 | 3.913611 | -1.240528 | -0.467530 |
| 75 | 6 | 0 | 5.248811 | -1.624377 | -0.578650 |
| 76 | 1 | 0 | 5.749771 | -2.079537 | 0.271180 |
| 77 | 6 | 0 | 5.945761 | -1.410917 | -1.769200 |
| 78 | 1 | 0 | 6.987131 | -1.707157 | -1.855120 |
| 79 | 6 | 0 | 5.297390 | -0.802497 | -2.844770 |
| 80 | 1 | 0 | 5.831990 | -0.625957 | -3.773750 |
| 81 | 1 | 0 | 3.475330 | 0.056242 | -3.581790 |
| 82 | 1 | 0 | 3.383361 | -1.357568 | 0.472610 |

---------------------------------------------------------------------

**TS4**-*E* G (hartrees): -1763.354735 Frequency: -187.2 $cm^{-1}$

Standard orientation:

| Center Number | Atomic Number | Atomic Type | Coordinates (Angstroms) X | Y | Z |
|---|---|---|---|---|---|
| 1 | 6 | 0 | -0.212571 | -3.668111 | -0.603029 |
| 2 | 6 | 0 | 0.577599 | -4.944581 | -0.275919 |
| 3 | 6 | 0 | 0.691490 | -2.587091 | -1.212759 |
| 4 | 1 | 0 | -0.680041 | -3.271000 | 0.306481 |
| 5 | 1 | 0 | -1.024671 | -3.912960 | -1.296659 |
| 6 | 6 | 0 | 1.909560 | -2.291571 | -0.309239 |
| 7 | 1 | 0 | 1.076150 | -2.968361 | -2.174239 |
| 8 | 7 | 0 | -0.009250 | -1.324041 | -1.493309 |
| 9 | 6 | 0 | 1.775559 | -4.649661 | 0.634601 |
| 10 | 1 | 0 | 0.933959 | -5.402221 | -1.209429 |
| 11 | 1 | 0 | -0.094281 | -5.673611 | 0.189891 |
| 12 | 6 | 0 | 2.683059 | -3.582571 | 0.011251 |
| 13 | 1 | 0 | 1.546160 | -1.839911 | 0.619041 |
| 14 | 7 | 0 | 2.739350 | -1.271601 | -0.942649 |
| 15 | 1 | 0 | 3.505879 | -3.323691 | 0.683741 |
| 16 | 1 | 0 | 3.130149 | -3.972751 | -0.915189 |
| 17 | 1 | 0 | 1.415239 | -4.296081 | 1.609991 |
| 18 | 1 | 0 | 2.344659 | -5.566381 | 0.825151 |
| 19 | 6 | 0 | 3.627130 | -0.544321 | -0.165759 |
| 20 | 1 | 0 | 3.052720 | -1.437841 | -1.891109 |
| 21 | 8 | 0 | 3.649960 | -0.576691 | 1.050071 |
| 22 | 8 | 0 | 4.439660 | 0.163399 | -0.978099 |
| 23 | 6 | 0 | 5.480110 | 1.048928 | -0.423589 |
| 24 | 6 | 0 | 6.525200 | 0.206448 | 0.314971 |
| 25 | 1 | 0 | 7.347760 | 0.847978 | 0.647361 |
| 26 | 1 | 0 | 6.939360 | -0.560212 | -0.348149 |
| 27 | 1 | 0 | 6.085290 | -0.277392 | 1.187811 |
| 28 | 6 | 0 | 6.077740 | 1.679358 | -1.683839 |
| 29 | 1 | 0 | 6.894881 | 2.354858 | -1.414019 |
| 30 | 1 | 0 | 5.318120 | 2.252028 | -2.223239 |
| 31 | 1 | 0 | 6.472200 | 0.909268 | -2.353709 |
| 32 | 6 | 0 | 4.852460 | 2.118118 | 0.474831 |
| 33 | 1 | 0 | 4.466680 | 1.679589 | 1.394701 |
| 34 | 1 | 0 | 4.021981 | 2.608409 | -0.037509 |
| 35 | 1 | 0 | 5.611601 | 2.867038 | 0.725731 |
| 36 | 6 | 0 | -1.323780 | -1.131440 | -1.496029 |
| 37 | 6 | 0 | -1.915070 | 0.155400 | -1.525079 |
| 38 | 6 | 0 | -1.178160 | 1.285930 | -2.219089 |
| 39 | 1 | 0 | 0.601540 | -0.509991 | -1.463659 |

| | | | | | |
|---|---|---|---|---|---|
| 40 | 1 | 0 | -1.950090 | -1.988140 | -1.294029 |
| 41 | 1 | 0 | -4.131780 | 0.549730 | 3.595641 |
| 42 | 1 | 0 | -1.865239 | 5.131560 | 0.978711 |
| 43 | 6 | 0 | -3.957020 | -0.530050 | 3.606161 |
| 44 | 1 | 0 | -4.376030 | -0.933050 | 4.534281 |
| 45 | 1 | 0 | -4.475750 | -0.980890 | 2.759031 |
| 46 | 6 | 0 | -1.126009 | 5.176730 | 0.172601 |
| 47 | 1 | 0 | -0.786979 | 6.212930 | 0.074611 |
| 48 | 1 | 0 | 0.082861 | 4.563599 | 2.619781 |
| 49 | 1 | 0 | -1.617879 | 4.887270 | -0.760749 |
| 50 | 6 | 0 | -2.460340 | -0.825640 | 3.555681 |
| 51 | 8 | 0 | -1.806480 | -0.109910 | 2.463091 |
| 52 | 1 | 0 | -1.979820 | -0.360120 | 4.421821 |
| 53 | 7 | 0 | -1.463410 | 0.533710 | 0.346341 |
| 54 | 6 | 0 | -2.124120 | -0.401210 | 1.190161 |
| 55 | 6 | 0 | 0.773851 | 4.618179 | 1.772241 |
| 56 | 8 | 0 | -2.928810 | -1.249430 | 0.844601 |
| 57 | 6 | 0 | 0.054891 | 4.263289 | 0.473851 |
| 58 | 1 | 0 | 1.175951 | 5.635579 | 1.718441 |
| 59 | 8 | 0 | -0.489599 | 2.921759 | 0.569441 |
| 60 | 6 | 0 | 0.374390 | 1.894709 | 0.328141 |
| 61 | 7 | 0 | -0.158820 | 0.624589 | 0.539911 |
| 62 | 6 | 0 | -2.115140 | -2.312900 | 3.550291 |
| 63 | 1 | 0 | 1.602741 | 3.928869 | 1.945641 |
| 64 | 1 | 0 | -2.442690 | -2.763740 | 4.493061 |
| 65 | 1 | 0 | 0.772401 | 4.275249 | -0.352529 |
| 66 | 1 | 0 | -2.606480 | -2.829860 | 2.724911 |
| 67 | 8 | 0 | 1.545390 | 2.067199 | 0.016971 |
| 68 | 1 | 0 | -1.032660 | -2.449980 | 3.468081 |
| 69 | 1 | 0 | -1.316870 | 1.186390 | -3.301739 |
| 70 | 1 | 0 | -0.104340 | 1.296079 | -2.022519 |
| 71 | 1 | 0 | -1.582660 | 2.250680 | -1.912739 |
| 72 | 1 | 0 | -3.638130 | -1.524280 | -2.860849 |
| 73 | 6 | 0 | -4.144590 | -0.688150 | -2.386879 |
| 74 | 6 | 0 | -3.405210 | 0.248630 | -1.651529 |
| 75 | 6 | 0 | -4.082630 | 1.339690 | -1.084359 |
| 76 | 1 | 0 | -3.520440 | 2.060710 | -0.499669 |
| 77 | 6 | 0 | -5.460830 | 1.475540 | -1.233809 |
| 78 | 1 | 0 | -5.969099 | 2.320670 | -0.778119 |
| 79 | 6 | 0 | -6.189050 | 0.531021 | -1.960459 |
| 80 | 1 | 0 | -7.263220 | 0.639741 | -2.078369 |
| 81 | 6 | 0 | -5.524700 | -0.550890 | -2.537349 |
| 82 | 1 | 0 | -6.078120 | -1.287669 | -3.112669 |

---------------------------------------------------------------------

**TS1**-*Z* G (hartrees): -1763.357583 Frequency: -200.1 cm$^{-1}$

Standard orientation:

| Center Number | Atomic Number | Atomic Type | Coordinates (Angstroms) X | Y | Z |
|---|---|---|---|---|---|
| 1 | 6 | 0 | -0.593143 | 3.524982 | 1.151461 |
| 2 | 6 | 0 | -0.156486 | 4.887508 | 0.591004 |
| 3 | 6 | 0 | 0.469240 | 2.458555 | 0.874501 |
| 4 | 1 | 0 | -1.540680 | 3.225229 | 0.684427 |
| 5 | 1 | 0 | -0.774562 | 3.607399 | 2.229104 |
| 6 | 6 | 0 | 0.809428 | 2.366591 | -0.639312 |
| 7 | 1 | 0 | 1.394806 | 2.721110 | 1.402005 |

| | | | | | |
|---|---|---|---|---|---|
| 8 | 7 | 0 | 0.078815 | 1.115111 | 1.330585 |
| 9 | 6 | 0 | 0.154980 | 4.802303 | -0.908990 |
| 10 | 1 | 0 | 0.734786 | 5.234130 | 1.130758 |
| 11 | 1 | 0 | -0.944282 | 5.624536 | 0.779984 |
| 12 | 6 | 0 | 1.224675 | 3.739396 | -1.194255 |
| 13 | 1 | 0 | -0.104484 | 2.034246 | -1.140456 |
| 14 | 7 | 0 | 1.782521 | 1.308659 | -0.909561 |
| 15 | 1 | 0 | 1.393830 | 3.638424 | -2.271864 |
| 16 | 1 | 0 | 2.176807 | 4.034538 | -0.743337 |
| 17 | 1 | 0 | -0.765539 | 4.556784 | -1.456724 |
| 18 | 1 | 0 | 0.490433 | 5.776567 | -1.281470 |
| 19 | 6 | 0 | 3.080117 | 1.344773 | -0.497655 |
| 20 | 1 | 0 | 1.437617 | 0.444570 | -1.336691 |
| 21 | 8 | 0 | 3.590227 | 2.251603 | 0.156905 |
| 22 | 8 | 0 | 3.717034 | 0.224473 | -0.920050 |
| 23 | 6 | 0 | 5.120048 | -0.045350 | -0.568041 |
| 24 | 6 | 0 | 5.271208 | -0.148546 | 0.953153 |
| 25 | 1 | 0 | 6.285199 | -0.478638 | 1.199869 |
| 26 | 1 | 0 | 4.566456 | -0.884772 | 1.352762 |
| 27 | 1 | 0 | 5.088431 | 0.813604 | 1.432086 |
| 28 | 6 | 0 | 5.371188 | -1.403977 | -1.227365 |
| 29 | 1 | 0 | 6.399452 | -1.726771 | -1.040341 |
| 30 | 1 | 0 | 5.218129 | -1.342087 | -2.308575 |
| 31 | 1 | 0 | 4.690494 | -2.159528 | -0.824900 |
| 32 | 6 | 0 | 6.030762 | 1.026992 | -1.174298 |
| 33 | 1 | 0 | 5.839932 | 2.001588 | -0.724946 |
| 34 | 1 | 0 | 5.867542 | 1.096034 | -2.254327 |
| 35 | 1 | 0 | 7.078197 | 0.757725 | -1.005239 |
| 36 | 6 | 0 | -0.965823 | 0.754868 | 2.056090 |
| 37 | 6 | 0 | -1.351454 | -0.599425 | 2.213895 |
| 38 | 1 | 0 | 0.678073 | 0.375777 | 0.978121 |
| 39 | 1 | 0 | -1.631404 | 1.535978 | 2.399957 |
| 40 | 1 | 0 | -6.028589 | -0.537455 | -1.271401 |
| 41 | 1 | 0 | -2.647682 | -4.650942 | -2.059240 |
| 42 | 6 | 0 | -6.080691 | 0.478113 | -0.868262 |
| 43 | 1 | 0 | -6.975042 | 0.959462 | -1.276499 |
| 44 | 1 | 0 | -6.175004 | 0.425146 | 0.217148 |
| 45 | 6 | 0 | -1.576789 | -4.763316 | -1.861992 |
| 46 | 1 | 0 | -1.207298 | -5.610999 | -2.446860 |
| 47 | 1 | 0 | -2.053431 | -2.994033 | -3.959069 |
| 48 | 1 | 0 | -1.442578 | -4.992897 | -0.802001 |
| 49 | 6 | 0 | -4.849080 | 1.278728 | -1.280215 |
| 50 | 8 | 0 | -3.608755 | 0.553132 | -0.987160 |
| 51 | 1 | 0 | -4.807601 | 1.318091 | -2.372763 |
| 52 | 7 | 0 | -2.144446 | -0.582951 | 0.301602 |
| 53 | 6 | 0 | -3.273558 | 0.280196 | 0.287504 |
| 54 | 6 | 0 | -0.994063 | -3.134392 | -3.720620 |
| 55 | 8 | 0 | -3.922960 | 0.619915 | 1.263195 |
| 56 | 6 | 0 | -0.822172 | -3.499351 | -2.249081 |
| 57 | 1 | 0 | -0.602647 | -3.937483 | -4.353308 |
| 58 | 8 | 0 | -1.363390 | -2.436268 | -1.410411 |
| 59 | 6 | 0 | -0.602427 | -1.328546 | -1.243551 |
| 60 | 7 | 0 | -1.220640 | -0.278856 | -0.594754 |
| 61 | 6 | 0 | -4.814998 | 2.705711 | -0.741282 |
| 62 | 1 | 0 | -0.451748 | -2.216531 | -3.955316 |
| 63 | 1 | 0 | -5.658309 | 3.266077 | -1.157522 |
| 64 | 1 | 0 | 0.241208 | -3.598090 | -2.013721 |
| 65 | 1 | 0 | -4.885368 | 2.722828 | 0.346991 |
| 66 | 8 | 0 | 0.512308 | -1.205608 | -1.762492 |
| 67 | 1 | 0 | -3.892419 | 3.207698 | -1.048128 |

| | | | | | |
|---|---|---|---|---|---|
| 68 | 6 | 0 | -2.461533 | -0.926156 | 3.192523 |
| 69 | 1 | 0 | -3.059372 | -1.763266 | 2.821203 |
| 70 | 1 | 0 | -2.021302 | -1.238019 | 4.147369 |
| 71 | 1 | 0 | -3.126093 | -0.080561 | 3.354882 |
| 72 | 6 | 0 | -0.322778 | -1.688485 | 2.110077 |
| 73 | 6 | 0 | -0.671981 | -2.959363 | 1.625740 |
| 74 | 6 | 0 | 0.965519 | -1.510572 | 2.645900 |
| 75 | 6 | 0 | 0.245067 | -4.008612 | 1.653221 |
| 76 | 1 | 0 | -1.659070 | -3.108374 | 1.205284 |
| 77 | 6 | 0 | 1.882860 | -2.562982 | 2.673984 |
| 78 | 1 | 0 | 1.247835 | -0.553573 | 3.073638 |
| 79 | 6 | 0 | 1.527114 | -3.815116 | 2.173738 |
| 80 | 1 | 0 | -0.042587 | -4.983850 | 1.271720 |
| 81 | 1 | 0 | 2.868904 | -2.403085 | 3.099904 |
| 82 | 1 | 0 | 2.238116 | -4.635450 | 2.195263 |

**TS2**-*Z* G (hartrees): -1763.355090 Frequency: -274.4 $cm^{-1}$

Standard orientation:

| Center Number | Atomic Number | Atomic Type | Coordinates (Angstroms) X | Y | Z |
|---|---|---|---|---|---|
| 1 | 6 | 0 | 2.494708 | -2.648762 | 2.014736 |
| 2 | 6 | 0 | 3.909917 | -3.152115 | 1.691116 |
| 3 | 6 | 0 | 2.006011 | -1.672907 | 0.931059 |
| 4 | 1 | 0 | 2.488303 | -2.132341 | 2.982821 |
| 5 | 1 | 0 | 1.804190 | -3.494763 | 2.096244 |
| 6 | 6 | 0 | 2.971267 | -0.480726 | 0.787818 |
| 7 | 1 | 0 | 1.962561 | -2.192196 | -0.035320 |
| 8 | 7 | 0 | 0.649281 | -1.160309 | 1.204355 |
| 9 | 6 | 0 | 4.891861 | -1.987773 | 1.500800 |
| 10 | 1 | 0 | 3.877647 | -3.757341 | 0.775252 |
| 11 | 1 | 0 | 4.250060 | -3.815405 | 2.493327 |
| 12 | 6 | 0 | 4.382582 | -0.994586 | 0.446768 |
| 13 | 1 | 0 | 3.020533 | 0.047948 | 1.747332 |
| 14 | 7 | 0 | 2.450584 | 0.467268 | -0.187780 |
| 15 | 1 | 0 | 5.050640 | -0.131433 | 0.369883 |
| 16 | 1 | 0 | 4.355151 | -1.471438 | -0.541908 |
| 17 | 1 | 0 | 5.025604 | -1.464950 | 2.457478 |
| 18 | 1 | 0 | 5.877785 | -2.367111 | 1.211422 |
| 19 | 6 | 0 | 2.777334 | 1.789636 | -0.118630 |
| 20 | 1 | 0 | 1.992250 | 0.110671 | -1.030252 |
| 21 | 8 | 0 | 3.412259 | 2.297316 | 0.800989 |
| 22 | 8 | 0 | 2.282964 | 2.436996 | -1.199439 |
| 23 | 6 | 0 | 2.467965 | 3.886744 | -1.381073 |
| 24 | 6 | 0 | 3.959731 | 4.215925 | -1.495879 |
| 25 | 1 | 0 | 4.082953 | 5.273843 | -1.748145 |
| 26 | 1 | 0 | 4.418892 | 3.621207 | -2.291723 |
| 27 | 1 | 0 | 4.479409 | 4.014682 | -0.558984 |
| 28 | 6 | 0 | 1.752889 | 4.147754 | -2.708828 |
| 29 | 1 | 0 | 1.815023 | 5.209369 | -2.964878 |
| 30 | 1 | 0 | 0.698141 | 3.867334 | -2.640266 |
| 31 | 1 | 0 | 2.212592 | 3.568947 | -3.514881 |
| 32 | 6 | 0 | 1.787656 | 4.651348 | -0.241134 |
| 33 | 1 | 0 | 2.279693 | 4.454280 | 0.711502 |
| 34 | 1 | 0 | 0.734905 | 4.360725 | -0.166215 |
| 35 | 1 | 0 | 1.829155 | 5.726090 | -0.444409 |

| | | | | | |
|---|---|---|---|---|---|
| 36 | 6 | 0 | -0.427614 | -1.879719 | 1.409936 |
| 37 | 6 | 0 | -1.741647 | -1.328682 | 1.518302 |
| 38 | 1 | 0 | 0.559015 | -0.147421 | 1.231778 |
| 39 | 1 | 0 | -0.310733 | -2.955722 | 1.328673 |
| 40 | 1 | 0 | -5.021451 | 0.103923 | -3.123700 |
| 41 | 1 | 0 | -1.268285 | -5.239302 | -1.665186 |
| 42 | 6 | 0 | -5.324174 | 0.814758 | -2.349131 |
| 43 | 1 | 0 | -6.081314 | 1.479867 | -2.776667 |
| 44 | 1 | 0 | -5.768288 | 0.266012 | -1.517564 |
| 45 | 6 | 0 | -0.190378 | -5.056836 | -1.613087 |
| 46 | 1 | 0 | 0.322311 | -5.880464 | -2.118807 |
| 47 | 1 | 0 | -1.313884 | -3.831913 | -3.860201 |
| 48 | 1 | 0 | 0.111308 | -5.061412 | -0.561550 |
| 49 | 6 | 0 | -4.128387 | 1.644520 | -1.890272 |
| 50 | 8 | 0 | -2.983388 | 0.798451 | -1.544628 |
| 51 | 1 | 0 | -3.728481 | 2.185622 | -2.753257 |
| 52 | 7 | 0 | -1.958965 | -0.922158 | -0.456950 |
| 53 | 6 | 0 | -3.070580 | -0.051620 | -0.503513 |
| 54 | 6 | 0 | -0.235775 | -3.677251 | -3.750339 |
| 55 | 8 | 0 | -4.045011 | -0.155502 | 0.226019 |
| 56 | 6 | 0 | 0.166800 | -3.736370 | -2.280419 |
| 57 | 1 | 0 | 0.284570 | -4.458287 | -4.313720 |
| 58 | 8 | 0 | -0.549856 | -2.703710 | -1.541248 |
| 59 | 6 | 0 | -0.065731 | -1.434098 | -1.602206 |
| 60 | 7 | 0 | -0.841168 | -0.454518 | -1.027424 |
| 61 | 6 | 0 | -4.446734 | 2.652908 | -0.790085 |
| 62 | 1 | 0 | 0.029312 | -2.709523 | -4.182390 |
| 63 | 1 | 0 | -5.153121 | 3.394657 | -1.176847 |
| 64 | 1 | 0 | 1.237428 | -3.535310 | -2.186070 |
| 65 | 1 | 0 | -4.886765 | 2.165505 | 0.080459 |
| 66 | 8 | 0 | 1.025399 | -1.177562 | -2.131761 |
| 67 | 1 | 0 | -3.538724 | 3.178031 | -0.479747 |
| 68 | 6 | 0 | -1.870055 | -0.000015 | 2.218679 |
| 69 | 6 | 0 | -2.564658 | 0.070056 | 3.438045 |
| 70 | 6 | 0 | -1.278024 | 1.179811 | 1.727542 |
| 71 | 6 | 0 | -2.645779 | 1.266260 | 4.151114 |
| 72 | 1 | 0 | -3.042105 | -0.815804 | 3.841313 |
| 73 | 6 | 0 | -1.361435 | 2.374970 | 2.443525 |
| 74 | 1 | 0 | -0.802786 | 1.184158 | 0.749256 |
| 75 | 6 | 0 | -2.039139 | 2.424116 | 3.662161 |
| 76 | 1 | 0 | -3.185750 | 1.289916 | 5.093194 |
| 77 | 1 | 0 | -0.900913 | 3.271011 | 2.037879 |
| 78 | 1 | 0 | -2.102124 | 3.354138 | 4.218750 |
| 79 | 6 | 0 | -2.801779 | -2.382450 | 1.792794 |
| 80 | 1 | 0 | -2.720468 | -3.185402 | 1.056440 |
| 81 | 1 | 0 | -2.677102 | -2.822764 | 2.789380 |
| 82 | 1 | 0 | -3.795194 | -1.946900 | 1.716646 |

---------------------------------------------------------------------

**TS1p**-*E* G (hartrees): -1610.961829 Frequency: -144.2 $cm^{-1}$

Standard orientation:

| Center Number | Atomic Number | Atomic Type | Coordinates (Angstroms) X | Y | Z |
|---|---|---|---|---|---|
| 1 | 6 | 0 | 0.313918 | -3.375271 | 0.834556 |
| 2 | 6 | 0 | -0.278427 | -4.618934 | 0.153773 |
| 3 | 6 | 0 | -0.686049 | -2.216982 | 0.814805 |

| | | | | | |
|---|---|---|---|---|---|
| 4 | 1 | 0 | 1.229331 | -3.077102 | 0.306912 |
| 5 | 1 | 0 | 0.596773 | -3.611651 | 1.866618 |
| 6 | 6 | 0 | -1.159302 | -1.898558 | -0.631393 |
| 7 | 1 | 0 | -1.573768 | -2.496948 | 1.395849 |
| 8 | 7 | 0 | -0.156910 | -0.979509 | 1.408447 |
| 9 | 6 | 0 | -0.721130 | -4.311247 | -1.282816 |
| 10 | 1 | 0 | -1.139337 | -4.976714 | 0.734011 |
| 11 | 1 | 0 | 0.463773 | -5.424374 | 0.164113 |
| 12 | 6 | 0 | -1.727166 | -3.152785 | -1.317291 |
| 13 | 1 | 0 | -0.273924 | -1.556326 | -1.175073 |
| 14 | 7 | 0 | -2.072332 | -0.755006 | -0.659660 |
| 15 | 1 | 0 | -1.985399 | -2.894170 | -2.350135 |
| 16 | 1 | 0 | -2.654114 | -3.446405 | -0.815938 |
| 17 | 1 | 0 | 0.160622 | -4.053461 | -1.885924 |
| 18 | 1 | 0 | -1.164360 | -5.202235 | -1.741334 |
| 19 | 6 | 0 | -3.333288 | -0.772645 | -0.146057 |
| 20 | 1 | 0 | -1.716145 | 0.127419 | -1.032467 |
| 21 | 8 | 0 | -3.848476 | -1.727735 | 0.430856 |
| 22 | 8 | 0 | -3.925743 | 0.428700 | -0.362682 |
| 23 | 6 | 0 | -5.292769 | 0.710615 | 0.104971 |
| 24 | 6 | 0 | -5.357940 | 0.619398 | 1.633007 |
| 25 | 1 | 0 | -6.344050 | 0.945666 | 1.978172 |
| 26 | 1 | 0 | -4.606811 | 1.275292 | 2.084548 |
| 27 | 1 | 0 | -5.187028 | -0.402182 | 1.972846 |
| 28 | 6 | 0 | -5.510036 | 2.153304 | -0.358895 |
| 29 | 1 | 0 | -6.508444 | 2.493538 | -0.069394 |
| 30 | 1 | 0 | -5.420143 | 2.226544 | -1.446467 |
| 31 | 1 | 0 | -4.771061 | 2.820232 | 0.094478 |
| 32 | 6 | 0 | -6.288396 | -0.232012 | -0.578619 |
| 33 | 1 | 0 | -6.127849 | -1.264159 | -0.267004 |
| 34 | 1 | 0 | -6.183910 | -0.169084 | -1.666268 |
| 35 | 1 | 0 | -7.310060 | 0.061814 | -0.317801 |
| 36 | 6 | 0 | 1.021806 | -0.765492 | 1.982744 |
| 37 | 6 | 0 | 1.503277 | 0.517676 | 2.300734 |
| 38 | 6 | 0 | 0.541168 | 1.675890 | 2.355188 |
| 39 | 1 | 0 | -0.781475 | -0.189969 | 1.303830 |
| 40 | 1 | 0 | 1.674323 | -1.621073 | 2.105128 |
| 41 | 1 | 0 | 5.489262 | 0.272225 | -2.158659 |
| 42 | 1 | 0 | 2.311393 | 5.284721 | -0.507206 |
| 43 | 6 | 0 | 5.573972 | -0.727921 | -1.723706 |
| 44 | 1 | 0 | 6.331150 | -1.280244 | -2.289178 |
| 45 | 1 | 0 | 5.905136 | -0.638014 | -0.688370 |
| 46 | 6 | 0 | 1.239138 | 5.257805 | -0.289075 |
| 47 | 1 | 0 | 0.816001 | 6.239490 | -0.522239 |
| 48 | 1 | 0 | 1.769637 | 4.390532 | -2.893681 |
| 49 | 1 | 0 | 1.107614 | 5.071847 | 0.780462 |
| 50 | 6 | 0 | 4.241563 | -1.465255 | -1.820504 |
| 51 | 8 | 0 | 3.133768 | -0.649210 | -1.312489 |
| 52 | 1 | 0 | 3.965658 | -1.549180 | -2.875877 |
| 53 | 7 | 0 | 2.102252 | 0.701954 | 0.206481 |
| 54 | 6 | 0 | 3.108528 | -0.277078 | -0.018993 |
| 55 | 6 | 0 | 0.709597 | 4.381494 | -2.620426 |
| 56 | 8 | 0 | 3.930353 | -0.616727 | 0.817979 |
| 57 | 6 | 0 | 0.545774 | 4.185801 | -1.117177 |
| 58 | 1 | 0 | 0.267024 | 5.335275 | -2.924738 |
| 59 | 8 | 0 | 1.154436 | 2.918849 | -0.723445 |
| 60 | 6 | 0 | 0.416179 | 1.800065 | -0.878737 |
| 61 | 7 | 0 | 1.054443 | 0.602384 | -0.579816 |
| 62 | 6 | 0 | 4.248620 | -2.862067 | -1.207252 |
| 63 | 1 | 0 | 0.208658 | 3.580519 | -3.168699 |

| | | | | | |
|---|---|---|---|---|---|
| 64 | 1 | 0 | 4.972699 | -3.485194 | -1.741931 |
| 65 | 1 | 0 | -0.517023 | 4.133276 | -0.865791 |
| 66 | 1 | 0 | 4.523170 | -2.831562 | -0.152101 |
| 67 | 8 | 0 | -0.728910 | 1.811083 | -1.339964 |
| 68 | 1 | 0 | 3.264409 | -3.329473 | -1.308124 |
| 69 | 1 | 0 | -0.049456 | 1.621392 | 3.279172 |
| 70 | 1 | 0 | -0.166174 | 1.691009 | 1.522898 |
| 71 | 1 | 0 | 1.069573 | 2.629277 | 2.350081 |
| 72 | 6 | 0 | 2.715114 | 0.616682 | 3.206993 |
| 73 | 1 | 0 | 2.332326 | 0.681694 | 4.238281 |
| 74 | 1 | 0 | 3.290056 | -0.308305 | 3.140426 |
| 75 | 6 | 0 | 3.648536 | 1.807827 | 2.958762 |
| 76 | 1 | 0 | 3.138071 | 2.768704 | 3.073767 |
| 77 | 1 | 0 | 4.470740 | 1.787797 | 3.681076 |
| 78 | 1 | 0 | 4.071405 | 1.759258 | 1.953978 |

---------------------------------------------------------------------

**TS1p**-*Z* G (hartrees): -1610.960996 Frequency: -148.4 $cm^{-1}$

Standard orientation:

| Center Number | Atomic Number | Atomic Type | Coordinates (Angstroms) X | Y | Z |
|---|---|---|---|---|---|
| 1 | 6 | 0 | 0.357735 | -3.429653 | 0.789112 |
| 2 | 6 | 0 | -0.200098 | -4.653989 | 0.046812 |
| 3 | 6 | 0 | -0.648821 | -2.277108 | 0.768893 |
| 4 | 1 | 0 | 1.290100 | -3.108456 | 0.306347 |
| 5 | 1 | 0 | 0.603292 | -3.698550 | 1.822734 |
| 6 | 6 | 0 | -1.070622 | -1.914901 | -0.682503 |
| 7 | 1 | 0 | -1.555272 | -2.578806 | 1.308632 |
| 8 | 7 | 0 | -0.148943 | -1.054809 | 1.417390 |
| 9 | 6 | 0 | -0.591748 | -4.302584 | -1.394517 |
| 10 | 1 | 0 | -1.079166 | -5.035670 | 0.582876 |
| 11 | 1 | 0 | 0.547096 | -5.454752 | 0.058837 |
| 12 | 6 | 0 | -1.603905 | -3.149476 | -1.428966 |
| 13 | 1 | 0 | -0.167797 | -1.549955 | -1.181143 |
| 14 | 7 | 0 | -1.988844 | -0.775848 | -0.707313 |
| 15 | 1 | 0 | -1.825671 | -2.858945 | -2.461776 |
| 16 | 1 | 0 | -2.546613 | -3.464543 | -0.972057 |
| 17 | 1 | 0 | 0.309713 | -4.020333 | -1.956001 |
| 18 | 1 | 0 | -1.011589 | -5.180852 | -1.897411 |
| 19 | 6 | 0 | -3.267590 | -0.816169 | -0.241549 |
| 20 | 1 | 0 | -1.623089 | 0.119020 | -1.040697 |
| 21 | 8 | 0 | -3.797805 | -1.791373 | 0.286011 |
| 22 | 8 | 0 | -3.859019 | 0.388454 | -0.442440 |
| 23 | 6 | 0 | -5.248099 | 0.644628 | -0.027835 |
| 24 | 6 | 0 | -5.381491 | 0.502753 | 1.491912 |
| 25 | 1 | 0 | -6.384256 | 0.812718 | 1.802453 |
| 26 | 1 | 0 | -4.655769 | 1.147144 | 1.998232 |
| 27 | 1 | 0 | -5.219600 | -0.528599 | 1.805646 |
| 28 | 6 | 0 | -5.457207 | 2.100086 | -0.454032 |
| 29 | 1 | 0 | -6.470797 | 2.422974 | -0.199582 |
| 30 | 1 | 0 | -5.318692 | 2.209684 | -1.533422 |
| 31 | 1 | 0 | -4.745797 | 2.757436 | 0.054106 |
| 32 | 6 | 0 | -6.203613 | -0.282365 | -0.786132 |
| 33 | 1 | 0 | -6.048533 | -1.323106 | -0.501443 |
| 34 | 1 | 0 | -6.050876 | -0.182974 | -1.865313 |
| 35 | 1 | 0 | -7.238538 | -0.004799 | -0.562422 |

| | | | | | |
|---|---|---|---|---|---|
| 36 | 6 | 0 | 0.997527 | -0.860002 | 2.056135 |
| 37 | 6 | 0 | 1.462323 | 0.415392 | 2.432596 |
| 38 | 6 | 0 | 0.479241 | 1.565836 | 2.487581 |
| 39 | 1 | 0 | -0.765791 | -0.260956 | 1.297977 |
| 40 | 1 | 0 | 1.643828 | -1.719178 | 2.185469 |
| 41 | 1 | 0 | 5.678493 | 0.421397 | -1.793695 |
| 42 | 1 | 0 | 2.522045 | 5.210246 | -0.750104 |
| 43 | 6 | 0 | 5.756042 | -0.596223 | -1.399899 |
| 44 | 1 | 0 | 6.553964 | -1.110625 | -1.944775 |
| 45 | 1 | 0 | 6.025961 | -0.548920 | -0.344111 |
| 46 | 6 | 0 | 1.446629 | 5.253576 | -0.550519 |
| 47 | 1 | 0 | 1.069023 | 6.214254 | -0.913411 |
| 48 | 1 | 0 | 1.968547 | 4.075440 | -3.026287 |
| 49 | 1 | 0 | 1.291804 | 5.209036 | 0.530384 |
| 50 | 6 | 0 | 4.444371 | -1.348292 | -1.605683 |
| 51 | 8 | 0 | 3.296076 | -0.573387 | -1.125059 |
| 52 | 1 | 0 | 4.229505 | -1.387469 | -2.677778 |
| 53 | 7 | 0 | 2.148237 | 0.678380 | 0.391382 |
| 54 | 6 | 0 | 3.188872 | -0.268806 | 0.182499 |
| 55 | 6 | 0 | 0.906098 | 4.134382 | -2.768828 |
| 56 | 8 | 0 | 3.966667 | -0.638710 | 1.046962 |
| 57 | 6 | 0 | 0.720978 | 4.116578 | -1.254892 |
| 58 | 1 | 0 | 0.502222 | 5.063320 | -3.183754 |
| 59 | 8 | 0 | 1.278313 | 2.880826 | -0.710607 |
| 60 | 6 | 0 | 0.521811 | 1.765005 | -0.811562 |
| 61 | 7 | 0 | 1.140825 | 0.578464 | -0.448889 |
| 62 | 6 | 0 | 4.442932 | -2.772132 | -1.057649 |
| 63 | 1 | 0 | 0.381025 | 3.295810 | -3.231016 |
| 64 | 1 | 0 | 5.199031 | -3.360970 | -1.586888 |
| 65 | 1 | 0 | -0.345130 | 4.131745 | -1.012881 |
| 66 | 1 | 0 | 4.668906 | -2.788013 | 0.009208 |
| 67 | 8 | 0 | -0.612789 | 1.772098 | -1.300337 |
| 68 | 1 | 0 | 3.470779 | -3.245673 | -1.225027 |
| 69 | 1 | 0 | -0.252518 | 1.338003 | 3.277866 |
| 70 | 1 | 0 | -0.101846 | 1.604610 | 1.558891 |
| 71 | 6 | 0 | 2.643982 | 0.494153 | 3.364865 |
| 72 | 1 | 0 | 2.289098 | 0.678399 | 4.388487 |
| 73 | 1 | 0 | 3.232153 | -0.421291 | 3.354334 |
| 74 | 6 | 0 | 1.087657 | 2.947364 | 2.744213 |
| 75 | 1 | 0 | 1.573037 | 3.004352 | 3.722413 |
| 76 | 1 | 0 | 1.822951 | 3.192359 | 1.974846 |
| 77 | 1 | 0 | 0.302300 | 3.708813 | 2.721265 |
| 78 | 1 | 0 | 3.303984 | 1.321784 | 3.095452 |

---------------------------------------------------------------------

**TS2p**-*E* G (hartrees): -1610.958593 Frequency: -233.8 cm$^{-1}$

Standard orientation:

| Center Number | Atomic Number | Atomic Type | Coordinates (Angstroms) X | Y | Z |
|---|---|---|---|---|---|
| 1 | 6 | 0 | -3.889240 | -0.755786 | -1.324531 |
| 2 | 6 | 0 | -5.233040 | -0.568673 | -0.605533 |
| 3 | 6 | 0 | -2.742663 | -0.147538 | -0.496073 |
| 4 | 1 | 0 | -3.914078 | -0.266105 | -2.306545 |
| 5 | 1 | 0 | -3.698037 | -1.819491 | -1.502150 |
| 6 | 6 | 0 | -2.993501 | 1.354813 | -0.236399 |
| 7 | 1 | 0 | -2.686765 | -0.658836 | 0.473050 |

| | | | | | |
|---|---|---|---|---|---|
| 8 | 7 | 0 | -1.432066 | -0.316646 | -1.151327 |
| 9 | 6 | 0 | -5.497561 | 0.909787 | -0.289955 |
| 10 | 1 | 0 | -5.227324 | -1.147396 | 0.327953 |
| 11 | 1 | 0 | -6.038586 | -0.978836 | -1.223751 |
| 12 | 6 | 0 | -4.334811 | 1.536157 | 0.493311 |
| 13 | 1 | 0 | -3.046028 | 1.858644 | -1.207307 |
| 14 | 7 | 0 | -1.916711 | 2.005867 | 0.498438 |
| 15 | 1 | 0 | -4.502294 | 2.606307 | 0.649506 |
| 16 | 1 | 0 | -4.264776 | 1.076657 | 1.488746 |
| 17 | 1 | 0 | -5.638676 | 1.461455 | -1.229220 |
| 18 | 1 | 0 | -6.427690 | 1.016228 | 0.278548 |
| 19 | 6 | 0 | -0.893687 | 2.624504 | -0.162138 |
| 20 | 1 | 0 | -1.683317 | 1.641818 | 1.421004 |
| 21 | 8 | 0 | -0.781726 | 2.642779 | -1.392609 |
| 22 | 8 | 0 | -0.072741 | 3.222713 | 0.713081 |
| 23 | 6 | 0 | 1.139054 | 3.950940 | 0.283852 |
| 24 | 6 | 0 | 0.724942 | 5.194899 | -0.506675 |
| 25 | 1 | 0 | 1.612338 | 5.788044 | -0.749176 |
| 26 | 1 | 0 | 0.049565 | 5.819022 | 0.086718 |
| 27 | 1 | 0 | 0.226514 | 4.916677 | -1.436283 |
| 28 | 6 | 0 | 1.773876 | 4.337857 | 1.621403 |
| 29 | 1 | 0 | 2.683676 | 4.919642 | 1.448299 |
| 30 | 1 | 0 | 2.035055 | 3.444489 | 2.195137 |
| 31 | 1 | 0 | 1.083303 | 4.943002 | 2.215764 |
| 32 | 6 | 0 | 2.070863 | 3.033114 | -0.511478 |
| 33 | 1 | 0 | 1.671832 | 2.829881 | -1.505515 |
| 34 | 1 | 0 | 2.208951 | 2.085118 | 0.015206 |
| 35 | 1 | 0 | 3.044929 | 3.521182 | -0.621272 |
| 36 | 6 | 0 | -0.871338 | -1.476083 | -1.419450 |
| 37 | 6 | 0 | 0.455112 | -1.611846 | -1.901972 |
| 38 | 6 | 0 | 1.019626 | -0.491400 | -2.753794 |
| 39 | 1 | 0 | -0.974146 | 0.546126 | -1.452557 |
| 40 | 1 | 0 | -1.405907 | -2.356588 | -1.076652 |
| 41 | 1 | 0 | 4.838360 | -2.147214 | 1.895033 |
| 42 | 1 | 0 | -1.101588 | -4.683973 | 1.735268 |
| 43 | 6 | 0 | 5.333171 | -1.666835 | 1.045714 |
| 44 | 1 | 0 | 6.391934 | -1.543881 | 1.295349 |
| 45 | 1 | 0 | 5.251456 | -2.316661 | 0.173334 |
| 46 | 6 | 0 | -1.908066 | -3.981127 | 1.966997 |
| 47 | 1 | 0 | -2.575829 | -4.452347 | 2.694442 |
| 48 | 1 | 0 | 0.285816 | -3.570479 | 3.651737 |
| 49 | 1 | 0 | -2.478428 | -3.793857 | 1.052301 |
| 50 | 6 | 0 | 4.718013 | -0.296084 | 0.775948 |
| 51 | 8 | 0 | 3.257601 | -0.366616 | 0.692888 |
| 52 | 1 | 0 | 4.841275 | 0.321489 | 1.670982 |
| 53 | 7 | 0 | 1.310002 | -1.280599 | -0.038050 |
| 54 | 6 | 0 | 2.675943 | -1.092215 | -0.289318 |
| 55 | 6 | 0 | -0.552051 | -2.886853 | 3.822091 |
| 56 | 8 | 0 | 3.299235 | -1.634583 | -1.194529 |
| 57 | 6 | 0 | -1.349462 | -2.685271 | 2.537499 |
| 58 | 1 | 0 | -1.192990 | -3.312651 | 4.600703 |
| 59 | 8 | 0 | -0.476120 | -2.132307 | 1.511046 |
| 60 | 6 | 0 | -0.250342 | -0.786949 | 1.536647 |
| 61 | 7 | 0 | 0.709413 | -0.314695 | 0.666549 |
| 62 | 6 | 0 | 5.331746 | 0.443329 | -0.409714 |
| 63 | 1 | 0 | -0.162018 | -1.932576 | 4.183973 |
| 64 | 1 | 0 | 6.389160 | 0.637725 | -0.202020 |
| 65 | 1 | 0 | -2.153965 | -1.966834 | 2.716639 |
| 66 | 1 | 0 | 5.253741 | -0.142698 | -1.325818 |
| 67 | 8 | 0 | -0.881056 | -0.040097 | 2.294300 |

| | | | | | |
|---|---|---|---|---|---|
| 68 | 1 | 0 | 4.834553 | 1.405859 | -0.559835 |
| 69 | 1 | 0 | 2.083948 | -0.652454 | -2.923727 |
| 70 | 1 | 0 | 0.897516 | 0.491176 | -2.288450 |
| 71 | 1 | 0 | 0.515538 | -0.462132 | -3.727387 |
| 72 | 6 | 0 | 0.845967 | -3.034704 | -2.269389 |
| 73 | 1 | 0 | 1.932957 | -3.088107 | -2.320684 |
| 74 | 1 | 0 | 0.531643 | -3.703846 | -1.460447 |
| 75 | 6 | 0 | 0.231192 | -3.507527 | -3.599035 |
| 76 | 1 | 0 | 0.536310 | -4.536974 | -3.812513 |
| 77 | 1 | 0 | -0.864036 | -3.482782 | -3.570695 |
| 78 | 1 | 0 | 0.557637 | -2.885477 | -4.438430 |

---------------------------------------------------------------------

**TS2p**-*Z* G (hartrees): -1610.957787 Frequency: -240.2 $cm^{-1}$

Standard orientation:

| Center Number | Atomic Number | Atomic Type | Coordinates (Angstroms) X | Y | Z |
|---|---|---|---|---|---|
| 1 | 6 | 0 | 3.895112 | -0.578346 | 1.424098 |
| 2 | 6 | 0 | 5.239362 | -0.450224 | 0.693145 |
| 3 | 6 | 0 | 2.739889 | -0.123786 | 0.513645 |
| 4 | 1 | 0 | 3.895889 | 0.041610 | 2.329947 |
| 5 | 1 | 0 | 3.733828 | -1.613009 | 1.744533 |
| 6 | 6 | 0 | 2.947590 | 1.338135 | 0.058614 |
| 7 | 1 | 0 | 2.712359 | -0.760156 | -0.379874 |
| 8 | 7 | 0 | 1.427512 | -0.247645 | 1.175100 |
| 9 | 6 | 0 | 5.463648 | 0.978943 | 0.180493 |
| 10 | 1 | 0 | 5.258815 | -1.150708 | -0.152644 |
| 11 | 1 | 0 | 6.050949 | -0.747949 | 1.365463 |
| 12 | 6 | 0 | 4.289001 | 1.460430 | -0.683392 |
| 13 | 1 | 0 | 2.977604 | 1.966736 | 0.954815 |
| 14 | 7 | 0 | 1.859020 | 1.855258 | -0.760521 |
| 15 | 1 | 0 | 4.426778 | 2.504737 | -0.980197 |
| 16 | 1 | 0 | 4.239332 | 0.870754 | -1.608940 |
| 17 | 1 | 0 | 5.583166 | 1.656433 | 1.036646 |
| 18 | 1 | 0 | 6.394160 | 1.034256 | -0.394637 |
| 19 | 6 | 0 | 0.822342 | 2.540547 | -0.193486 |
| 20 | 1 | 0 | 1.640440 | 1.363666 | -1.625807 |
| 21 | 8 | 0 | 0.705159 | 2.724729 | 1.022486 |
| 22 | 8 | 0 | -0.005943 | 2.999308 | -1.143836 |
| 23 | 6 | 0 | -1.202710 | 3.803641 | -0.821041 |
| 24 | 6 | 0 | -0.763800 | 5.138787 | -0.213557 |
| 25 | 1 | 0 | -1.639134 | 5.778122 | -0.061861 |
| 26 | 1 | 0 | -0.075104 | 5.658172 | -0.887130 |
| 27 | 1 | 0 | -0.272436 | 4.985963 | 0.748291 |
| 28 | 6 | 0 | -1.835459 | 4.010971 | -2.198908 |
| 29 | 1 | 0 | -2.732610 | 4.629849 | -2.107805 |
| 30 | 1 | 0 | -2.118048 | 3.051479 | -2.640736 |
| 31 | 1 | 0 | -1.135798 | 4.512028 | -2.874020 |
| 32 | 6 | 0 | -2.149412 | 3.026075 | 0.096407 |
| 33 | 1 | 0 | -1.738407 | 2.933285 | 1.101837 |
| 34 | 1 | 0 | -2.330179 | 2.026239 | -0.305968 |
| 35 | 1 | 0 | -3.104900 | 3.557512 | 0.157539 |
| 36 | 6 | 0 | 0.906681 | -1.376700 | 1.606158 |
| 37 | 6 | 0 | -0.421749 | -1.485514 | 2.092293 |
| 38 | 6 | 0 | -1.017945 | -0.283521 | 2.814799 |
| 39 | 1 | 0 | 0.930223 | 0.629876 | 1.338112 |

| | | | | | |
|---|---|---|---|---|---|
| 40 | 1 | 0 | 1.477652 | -2.276280 | 1.398257 |
| 41 | 1 | 0 | -4.752592 | -2.462072 | -1.710587 |
| 42 | 1 | 0 | 1.158400 | -4.995264 | -1.105023 |
| 43 | 6 | 0 | -5.264795 | -1.922856 | -0.908231 |
| 44 | 1 | 0 | -6.322449 | -1.838283 | -1.177709 |
| 45 | 1 | 0 | -5.183096 | -2.498623 | 0.014730 |
| 46 | 6 | 0 | 1.968085 | -4.324250 | -1.408673 |
| 47 | 1 | 0 | 2.649283 | -4.880624 | -2.059461 |
| 48 | 1 | 0 | -0.201200 | -4.136864 | -3.160558 |
| 49 | 1 | 0 | 2.521273 | -4.022063 | -0.514480 |
| 50 | 6 | 0 | -4.673964 | -0.525109 | -0.743797 |
| 51 | 8 | 0 | -3.214216 | -0.568094 | -0.634606 |
| 52 | 1 | 0 | -4.791325 | 0.014403 | -1.688708 |
| 53 | 7 | 0 | -1.267516 | -1.398539 | 0.201737 |
| 54 | 6 | 0 | -2.637215 | -1.195522 | 0.415457 |
| 55 | 6 | 0 | 0.637583 | -3.477485 | -3.405675 |
| 56 | 8 | 0 | -3.266183 | -1.657213 | 1.361209 |
| 57 | 6 | 0 | 1.417849 | -3.112127 | -2.146840 |
| 58 | 1 | 0 | 1.289607 | -3.996843 | -4.115265 |
| 59 | 8 | 0 | 0.531971 | -2.434499 | -1.209798 |
| 60 | 6 | 0 | 0.298950 | -1.106173 | -1.414594 |
| 61 | 7 | 0 | -0.667839 | -0.529005 | -0.619612 |
| 62 | 6 | 0 | -5.317901 | 0.300776 | 0.366509 |
| 63 | 1 | 0 | 0.250823 | -2.578400 | -3.891260 |
| 64 | 1 | 0 | -6.372085 | 0.468185 | 0.122008 |
| 65 | 1 | 0 | 2.224849 | -2.421159 | -2.405616 |
| 66 | 1 | 0 | -5.254968 | -0.208649 | 1.328350 |
| 67 | 8 | 0 | 0.930640 | -0.461631 | -2.260376 |
| 68 | 1 | 0 | -4.831594 | 1.277017 | 0.448856 |
| 69 | 1 | 0 | -2.090644 | -0.452269 | 2.921848 |
| 70 | 1 | 0 | -0.907563 | 0.622354 | 2.206996 |
| 71 | 6 | 0 | -0.794045 | -2.857436 | 2.614828 |
| 72 | 1 | 0 | -1.876271 | -2.950867 | 2.679964 |
| 73 | 1 | 0 | -0.415648 | -3.641826 | 1.954053 |
| 74 | 6 | 0 | -0.394818 | -0.043170 | 4.202730 |
| 75 | 1 | 0 | -0.871206 | 0.816935 | 4.683377 |
| 76 | 1 | 0 | -0.531929 | -0.907939 | 4.858998 |
| 77 | 1 | 0 | 0.678461 | 0.162518 | 4.137111 |
| 78 | 1 | 0 | -0.368049 | -3.020091 | 3.613112 |

---------------------------------------------------------------------

## X. References


1. Lee, D. W.; Ha, H.-J.; Lee, W. K., Selective Mono-BOC Protection of Diamines. *Synth. Commun.* **2007**, *37*, 737-742.
2. Baumann, T.; Vogt, H.; Bräse, S., The Proline-Catalyzed Asymmetric Amination of Branched Aldehydes. *Eur. J. Org. Chem.* **2007**, 266-282.
3. Li, X.; Carter, R. G. Pummerer Cyclization Revised: Unraveling of Acyl Oxonium Ion and Vinyl Sulfide Pathways. *Org. Lett.* **2018**, *50*, 5541-5545.
4. Fu, J.-Y.; Xu, X.-Y.; Li, Y.-C.; Huang, Q.-C.; Wang, L.-X., Effective Construction of Quaternary Stereocenters by Highly Enantioselective α-Amination of Branched Aldehydes *Org. Biomol. Chem.* **2010**, *8*, 4524-4526.
5. Havare, N.; Plattner, D. A. Oxidative Cleavage of α-Aryl Aldehydes Using Iodosylbenzene. *Org. Lett.* **2012**, *14*, 5078-5081.
6. Fu, J.-Y.; Yang, Q.-C.; Wang, Q.-L.; Ming, J.-N.; Wang, F.-Y.; Xu, X.-Y.; Wang, L.-X., Enantioselective α-Amination of Branched Aldehydes Promoted by Simple Chiral Primary Amino Acids. *J. Org. Chem.* **2011**, *76*, 4661-4664.
7. Sheldon, R. A., The E Factor: Fifteen Years On. *Green Chem.* **2007**, *9*, 1273-1283.

8. Van Aken, K.; Strekowski, L.; Patiny, L., EcoScale, a Semi-Quantitative Tool to Select an Organic Preparation Based on Economical and Ecological Parameters. *Beilstein J. Org. Chem.* **2006**, *2*, 1–7.
9. Desmarchelier, A.; Yalgin, H.; Coeffard, V.; Moreau, X.; Greck, C., Primary Amine Catalyzed Electrophilic Amination of α,α-Disubstituted Aldehydes. *Tetrahedron Lett.* **2011**, *52*, 4430-4432.
10. Fu, J.-Y.; Wang, Q.-L.; Peng, L.; Gui, Y.-Y.; Wang, L.-X., Construction of Quaternary Stereocenters: Asymmetric Amination of Branched Aldehydes Catalyzed by Monoimide Substituted Cyclohexane-1,2-Diamines. *Chirality* **2013**, *25*, 668-672.
11. Fu, J.-Y.; Wang, Q.-L.; Peng, L.; Gui, Y.-Y.; Wang, F.; Tian, F.; Xu, X.-Y.; Wang, L.-X., Chiral α-Arylethanamines: An Organocatalyst for the Enantioselective α-Amination of Branched Aldehydes. *Eur. J. Org. Chem.* **2013**, *2013*, 2864-2868.
12. Theodorou, A.; Papadopoulos, G. N.; Kokotos, C. G., β-*tert*-Butyl Aspartate as an Organocatalyst for the Asymmetric α-Amination of α,α-Disubstituted Aldehydes. *Tetrahedron* **2013**, *69*, 5438-5443.
13. Liu, C.; Zhu, Q.; Huang, K.-W.; Lu, Y., Primary Amine/CSA Ion Pair: A Powerful Catalytic System for the Asymmetric Enamine Catalysis. *Org. Lett.* **2011**, *13*, 2638-2641.
14. Gaussian 16, Revision C.01, Frisch, M. J.; Trucks, G. W.; Schlegel, H. B.; Scuseria, G. E.; Robb, M. A.; Cheeseman, J. R.; Scalmani, G.; Barone, V.; Petersson, G. A.; Nakatsuji, H.; Li, X.; Caricato, M.; Marenich, A. V.; Bloino, J.; Janesko, B. G.; Gomperts, R.; Mennucci, B.; Hratchian, H. P.; Ortiz, J. V.; Izmaylov, A. F.; Sonnenberg, J. L.; Williams-Young, D.; Ding, F.; Lipparini, F.; Egidi, F.; Goings, J.; Peng, B.; Petrone, A.; Henderson, T.; Ranasinghe, D.; Zakrzewski, V. G.; Gao, J.; Rega, N.; Zheng, G.; Liang, W.; Hada, M.; Ehara, M.; Toyota, K.; Fukuda, R.; Hasegawa, J.; Ishida, M.; Nakajima, T.; Honda, Y.; Kitao, O.; Nakai, H.; Vreven, T.; Throssell, K.; Montgomery, J. A. N.; Peralta, J. E.; Ogliaro, F.; Bearpark, M. J.; Heyd, J. J.; Brothers, E. N.; Kudin, K. N.; Staroverov, V. N.; Keith, T. A.; Kobayashi, R.; Normand, J.; Raghavachari, K.; Rendell, A. J.; Burant, J. C.; Iyengar, S. S.; Tomasi, J.; Cossi, M.; Millam, J. M.; Klene, M.; Adamo, C.; Cammi, R.; Ochterski, J. W.; Martin, R. L.; Morokuma, K.; Farkas, O.; Foresman, J. B.; Fox, D. J., Gaussian, Inc., Wallingford CT, **2019**.
15. Chai, J.-D.; Head-Gordon, M., Long-Range Corrected Hybrid Density Functionals with Damped Atom-Atom Dispersion Corrections. *Phys. Chem. Chem. Phys.* **2008**, *10*, 6615-6620.
16. Weigend, F.; Ahlrichs, R., Balanced Basis Sets of Split Valence, Triple Zeta Valence and Quadruple Zeta Valence Quality for H to Rn: Design and Assessment of Accuracy. *Phys. Chem. Chem. Phys.* **2005**, *7,* 3297-305.
17. Cancés, E.; Mennucci, B.; Tomasi, J., A New Integral Equation Formalism for the Polarizable Continuum Model: Theoretical Background and Applications to Isotropic and Anisotropic Dielectrics. *J. Chem. Phys*. **1997**, *107*, 3032-3041.
18. Cossi, M.;Barone, V.; Mennucci, B.; Tomasi, J., Ab Initio Study of Ionic Solutions by a Polarizable Continuum Dielectric Model. *Chem. Phys. Lett.* **1998**, *286*, 253-260.
19. Tomasi, J.; Mennucci, B.; Cancès, E., The IEF Version of the PCM Solvation Method: An Overview of a New Method Addressed to Study Molecular Solutes at the QM ab Initio Level *J. Mol. Struc.: THEOCHEM* **1999**, *464*, 211-226.
